# Access to *N*‐Monofluoromethylated (Thio)Carbamates, Formamides, Alkynamides, and Related Derivatives

**DOI:** 10.1002/anie.202505478

**Published:** 2025-05-10

**Authors:** Filip G. Zivkovic, Fritz Bahns, Che‐Ming Hsu, Franziska Schoenebeck

**Affiliations:** ^1^ Institute of Organic Chemistry RWTH Aachen University Landoltweg 1 52074 Aachen Germany

**Keywords:** Building block, Fluorine, Monofluoromethyl

## Abstract

This work presents the first general synthetic access to *N*‐CH₂F and *N*‐CHRF carbamates, thiocarbamates, formamides, alkynamides, and related compounds. The synthetic approach employs *N*‐CH_2_F and *N*‐CHRF carbamoyl fluorides as versatile strategic building blocks, which can be efficiently synthesized in a single step directly from readily available amines or imines.

## Graphical abstract

The first general synthetic access to *N*‐CH₂F and *N*‐CHRF carbamates, thiocarbamates, formamides, alkynamides, and related compounds is disclosed. The method involves the direct synthesis of carbamoyl fluoride building blocks from readily available amines, followed by their derivatization.

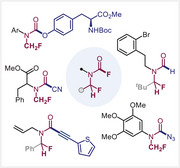

Thiocarbamates, ureas, formamides, and alkynamides—all members of the *N*‐carbonyl family—are defined by the amide bond (R_2_ *N─*C═O), a fundamental motif that underpins advancements across the physical and life sciences.^[^ ^1^ ^]^ These versatile compounds are ubiquitous, serving as essential components in pharmaceuticals (both as active drugs and prodrugs), cosmetics, advanced materials, and agrochemicals as potent pesticides.^[^ ^2^, ^3^, ^4^, ^5^, ^6^, ^7^, ^8^, ^9^ ^]^ The strategic modification of these core structures holds significant potential for expanding the boundaries of chemical space, offering a promising approach to engineering molecules with novel properties and enabling function.^[^ ^10^, ^11^, ^12^ ^]^ In this context, the fluorination of organic molecules has become a widely pursued strategy for modulating key physicochemical properties, including conformation, stability, pH, and lipophilicity.^[^ ^13^, ^14^, ^15^ ^]^ Likewise, *N*‐methylation of peptides or polymers has been recognized for its ability to enhance solubility, metabolic stability, and cellular permeability.^[^ ^8^, ^16^, ^17^, ^18^, ^19^ ^]^ In light of the synergistic potential of these modifications, the combined *N*‐CF_3_ and *N*‐CF_2_H carbamoyl motifs hold significant potential, and their synthetic access as analogues of amides,^[^ ^20^, ^21^, ^22^, ^23^, ^24^ ^]^ ureas,^[^ ^20^ ^]^ carbamates,^[^ ^20^ ^]^ formamides,^[^ ^25^ ^]^ hydrazines,^[^ ^26^, ^27^ ^]^ amines,^[^ ^28^ ^]^ indoles,^[^ ^26^ ^]^ and related derivatives,^[^ ^29^, ^30^, ^31^, ^32^ ^]^ has recently been unlocked. Our previous findings demonstrate a distinct correlation between the degree of fluorination and the molecular properties, with increasing fluorine content (*N*‐CH_3 _→ *N*‐CF_2_H → *N*‐CF_3_) leading to enhanced lipophilicity and greater conformational flexibility.^[^ ^33^ ^]^ The *N*‐monofluoromethyl motif is currently a missing link in the fine tuning of the properties of *N*‐carbonyl family.

The monofluoromethyl (─CH_2_F) group already has significance in medicinal chemistry, serving as a bioisostere for key functional moieties, including methyl, hydroxymethyl, and aminomethyl groups.^[^ ^34^, ^35^ ^]^ While significant progress has been made in monofluoromethylation of heteroatom nucleophiles, such as phenols, thiols, diverse heterocycles, lactams, and tertiary amines,^[^ ^36^, ^37^, ^38^, ^39^, ^40^ ^]^ the inherent instability and propensity for dehydrofluorination,^[^ ^39^ ^]^ of R(CH_2_F)*N*‐H amines renders conventional amide coupling^[^ ^41^ ^]^ strategies ineffective for direct access to *N*‐CH_2_F carbonyl derivatives.

There is currently no broadly applicable synthetic method for accessing the diverse *N*‐CH_2_F carbonyl family, including (thio)carbamates, alkynamides, and formamides. While a very recent report^[^ ^42^ ^]^ disclosed a strategy for synthesizing the corresponding amides,^[^ ^43^ ^]^ its approach inherently limits expansion to the broader *N*‐CH_2_F carbonyl family. We therefore envisioned the development of a versatile building block that would provide a streamlined and efficient pathway to multiple members of this compound class (Figure 1).

**Figure 1:**
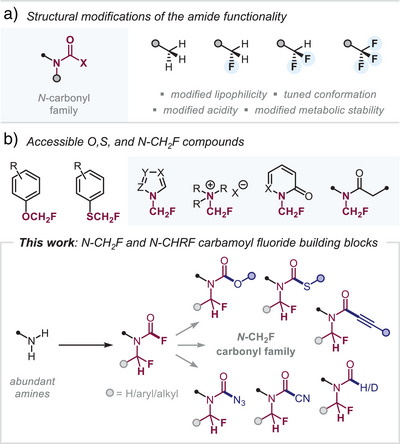
Accessible *O*‐, *S*‐, and *N*‐CH_2_F compounds and this work.

Our research group has previously demonstrated the efficacy of a building block approach for the synthesis of *N*‐CF_3_ and *N*‐CF_2_H amides, along with their broader *N*‐carbonyl derivatives,^[^ ^20^, ^33^ ^]^ through straightforward derivatizations of the corresponding carbamoyl fluorides (Scheme 1a). Building on this strategy, our objective was to further expand access to the broader *N*‐CH_2_F carbonyl family, extending beyond amides, by developing a robust and scalable methodology for synthesizing the *N*‐monofluoromethyl carbamoyl fluoride building block.

**Scheme 1:**
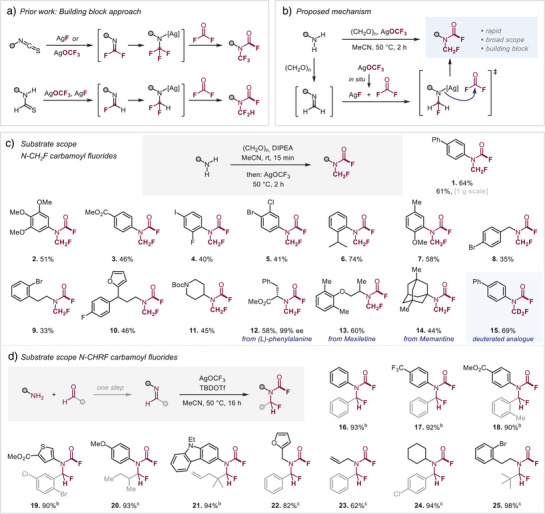
Scope of *N*‐CH_2_F and *N*‐CHRF carbamoyl fluorides. Reaction conditions: a) Amine (1 equiv), (CH_2_O)*_n_* (5 equiv), DIPEA (1 equiv), MeCN, rt, 15 min; then AgOCF_3_ (2.5 equiv), 50 °C, 2 h; b) Imine (1 equiv), TBDOTf (1.1 equiv), AgOCF_3_ (2.0 equiv), MeCN, 50 °C, 16 h; c) Imine (1 equiv), AgOCF_3_ (2.0 equiv), MeCN, 50 °C, 16 h; isolated yields are given.

The successful synthesis of *N*‐CF_3_ and *N*‐CF_2_H carbamoyl fluorides relied on the formation of fluoro‐imine species^[^ ^20^, ^33^, ^44^ ^]^ as key reactive intermediates. Applying this strategic design to the *N*‐monofluoromethyl analogue necessitates a reaction pathway involving the fluorination and carbamoylation of methanimine (Scheme 1b), which is ideally generated in situ directly from its parent amines. If successfully realized, this approach would enable the direct and efficient synthesis of the desired *N*‐CH_2_F carbamoyl fluoride building block in a single step, starting from readily available amines.

The challenge of this one‐pot strategy is to prevent undesired cross‐reactions of the various involved electrophiles and nucleophiles, i.e., enable a sequence of selective conversion of the nucleophilic amine to the imine with an appropriate electrophile, to then convert the species to a nucleophile upon fluoride attack and quench with yet another, different carbonyl electrophile (Scheme 1b). We envisioned that this is best realizable if reagents are employed that allow for delayed availability of reagents through in situ release of the key reactive species. Following extensive investigations (see Supporting Information for details), we ultimately identified that the convenient solid reagent paraformaldehyde (CH_2_O)*_n_* (which liberates formaldehyde upon heating in situ) allows for rapid imine formation^[^ ^45^ ^]^ with biphenyl aniline in the presence of AgF and AgOCF_3_. The formed imine then reacts with AgF, while AgOCF_3_ slowly releases difluorocarbonyl (O═CF_2_) in situ for trapping the formed Ag‐(R)*N*CH_2_F.^[^ ^46^ ^]^ While this initially gave 16% of the desired *N*‐CH_2_F carbamoyl fluoride (**1**, Scheme 1c), the addition of DIPEA as likely additional activator of AgOCF_3_ for more efficient in situ release of AgF and O = CF_2_^[^ ^44^ ^]^ was found to significantly improve the yield to 64%. Additionally, we observed that pre‐stirring of the amine with paraformaldehyde was critical for reproducibility, and additional AgF was no longer needed. The method proved to be equally effective at larger scale, giving 61% of product with 1 g of starting material.

With the optimized conditions established, we proceeded to investigate the substrate scope (Scheme 1c). Notably, our methodology demonstrated broad applicability to both anilines and alkyl amines. In the case of anilines, a wide range of functional groups were tolerated, including electron‐donating (**2**) and electron‐withdrawing (**3**) substituents, as well as halogens (**4** and **5**). Steric hindrance posed no significant challenge, as evidenced by the successful transformation of *ortho*‐substituted anilines bearing *^i^* Pr (**6**) and OMe (**7**) groups, yielding 74% and 58%, respectively. Similarly, alkyl amines exhibited good reactivity, with benzylic (**8**) and extended alkyl chains (**9** and **10**) delivering the desired products efficiently, alongside heterocyclic substrates (**10** and **11**).^[^ ^47^ ^]^ The robustness of this strategy was further demonstrated by the successful synthesis of an amino acid‐derived *N*‐CH_2_F carbamoyl fluoride with full conservation of stereochemistry (**12**) and the more structurally complex Mexiletine and Memantine derivatives (**13** and **14**). Furthermore, this approach facilitates the straightforward incorporation of deuterium with high efficiency by employing deuterated paraformaldehyde, enabling isotopic labeling and the first reported synthesis of the *N*‐CD_2_F motif (**15**).

Building upon this approach, we envisioned that expanding beyond paraformaldehyde to other aldehydes could grant access to alternative building blocks featuring the *N*‐CHRF motif, thereby unlocking new opportunities for molecular tuning (Scheme 1d). However, when benzaldehyde was used in place of paraformaldehyde, with aniline as the amine source under otherwise identical conditions, the desired product was not obtained. We hypothesized that this outcome was due to the inherently slower imine formation and the reduced reactivity of the resulting intermediates.

To overcome this limitation, we shifted from a one‐pot approach to a stepwise strategy, opting to pre‐form and isolate the imine intermediate, given that substituted aldimines are significantly more stable than methanimines. Notably, a patent procedure describes the conversion of aldimines to *N*‐CH(Ph)F carbamoyl fluorides using fluorophosgene gas under high pressure in an autoclave, though the reported scope remains limited to methyl, ethyl, and butyl amine under these conditions.^[^ ^48^ ^]^ Through systematic optimization of concentration and reaction time, we identified that treating the corresponding imine with AgOCF_3_ in the presence of TBDOTf as a Lewis acid enabled the formation of the *N*‐CHRF carbamoyl fluoride (**16**) in 93% yield following a simple filtration over celite and silica. While the Lewis acid proved beneficial for aromatic amines, it had an adverse effect on alkyl amines—likely due to the preferential formation of enamines under these conditions—and was therefore omitted in those cases.

This approach allowed us to systematically explore the extent of variability associated with the *N*‐CHRF motif by modifying substituents on both the amine and aldehyde (Scheme 1d). Encouragingly, a broad range of combinations was successfully achieved, including aryl–aryl (**16**–**18**), heteroaryl–aryl (**19**), (hetero)aryl–alkyl (**20** and **21**), alkyl–aryl (**22**–**24**), and alkyl–alkyl (**25**) systems, all with excellent yields (62%–98%). This methodology demonstrated remarkable functional group tolerance, accommodating trifluoromethyl (**17**), esters (**18** and **19**), methoxy (**20**), halogens (**17**, **19**, and **25**), terminal alkenes (**21**), allyl functionalities (**23**), and sterically demanding *tert*‐butyl (**25**) groups.

Having successfully established the synthesis of diverse *N*‐CH_2_F and *N*‐CHRF carbamoyl fluorides, we next investigated their potential for further derivatization. By making slight modifications to our previously developed protocols for the functionalization of *N*‐CF_3_ and *N*‐CF_2_H carbamoyl fluorides (i.e., omitting DMAP and using DBU instead as well as elevated temperature), we gained access to the corresponding *N*‐CH_2_F carbonyl family (Scheme 2). A range of natural products containing hydroxyl functionalities, including a flavone derivative (**26**), tyrosine (**27**), and galactose (**28**), were compatible with this approach, affording the corresponding *N*‐CH_2_F carbamates in high yields via direct nucleophilic addition. Similarly, *N*‐CH_2_F thiocarbamates were readily accessible through the nucleophilic addition of both aliphatic and aromatic thiolates (**29**–**31**).

**Scheme 2:**
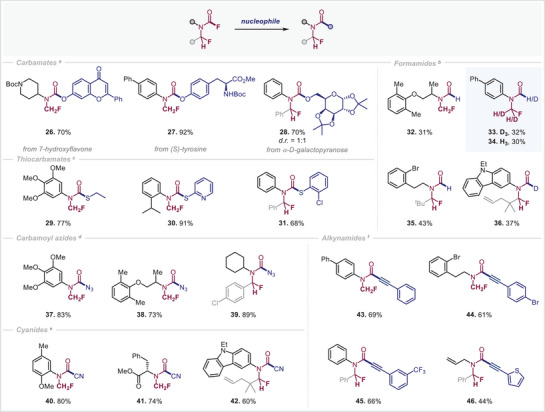
Derivatizations of *N*‐CH_2_F and *N*‐CHRF carbamoyl fluorides. Reaction conditions: a) For *N*‐CH_2_F carbamoyl fluoride (1 equiv): alcohol (2 equiv) DBU (2 equiv), MeCN, 80 °C, 3 h; for *N*‐CHRF carbamoyl fluoride (1 equiv): TMS‐OR (1.2 equiv), BTMG (20 mol%), TBAF (10 mol%) 60 °C, 2 h; b) for *N*‐CH_2_F carbamoyl fluoride (1 equiv): NaBH_4_ (2 equiv), DCM/*^t^* AmOH, rt, 3.5–15 h; for *N*‐CHRF carbamoyl fluoride (1 equiv): NaBH_4_ (2 equiv), MeCN, 50 °C, 2 h; c) carbamoyl fluoride (1 equiv), sodium‐thiolate (1.2 equiv), THF, rt, 16 h; d) for *N*‐CH_2_F carbamoyl fluoride (1 equiv): NaN_3_ (1.2 equiv), THF rt, 16 h; for *N*‐CHRF carbamoyl fluoride (1 equiv): TMSN_3_ (1.2 equiv), DBU (20 mol%), MeCN, rt, 1 h; e) for *N*‐CH_2_F carbamoyl fluoride (1 equiv): NaCN (1.2 equiv) THF, rt, 16 h; for *N*‐CHRF carbamoyl fluoride (1 equiv): TMSCN (1.5 equiv), DBU (5 mol%), MeCN, rt, 20 min; f) carbamoyl fluoride (1 equiv), TBAF (10 mol%), TMS‐alkyne (1.5 equiv), PhMe, 60 °C, 16 h; isolated yields are given.

Furthermore, applying our previously developed mild reduction strategy^[^ ^25^ ^]^ using NaBH_4_ or NaBD_4_, both *N*‐CH_2_F and *N*‐CHRF carbamoyl fluorides were efficiently converted into their respective formamide analogues (**32**–**36**).^[^ ^49^ ^]^ Formamides are key structural motifs in various pharmaceuticals and biologically active compounds, including the asthma and COPD drug arformoterol,^[^ ^50^ ^]^ the potent antitumor agent aplyronine A,^[^ ^51^ ^]^ the antiobesity drugs lipstatin^[^ ^52^ ^]^ and orlistat,^[^ ^53^, ^54^ ^]^ as well as the naturally occurring antimycins.^[^ ^55^ ^]^ Notably, this transformation offers a unique opportunity for isotope labeling at two distinct positions (**33**).

Further diversification was achieved through the introduction of simple nucleophiles, yielding carbamoyl azides (**37**–**39**) and carbamoyl cyanides (**40**–**42**) in good yields. These compounds serve as valuable synthetic intermediates for further transformations.

While our newly synthesized *N*‐CH_2_F carbamoyl fluorides remained unreactive under our previously established conditions for Ni‐catalyzed coupling with TMS‐alkynes,^[^ ^30^ ^]^ we successfully leveraged the TBAF‐catalyzed protocol developed by Le and coworkers^[^ ^56^ ^]^ to achieve the coupling. This strategy enabled the incorporation of an alkyne moiety, furnishing *N*‐CH_2_F and *N*‐CHRF alkynamides (**43**–**46**). Alkynamides are highly relevant bioactive scaffolds, playing a crucial role in the development of potent kinase inhibitors used in anticancer therapies, such as acalabrutinib and branebrutinib.^[^ ^7^, ^57^ ^]^

The corresponding ureas and amides remain a challenge to access through our building block approach. While ureas were successfully formed and detected in the crude reaction mixture (according to ^1^H and ^19^F NMR), their isolation was prevented by the direct hydrolysis of the *N*‐CH_2_F unit to *N*‐H (see Supporting Information). On the other hand, *N*‐CH_2_F amides could not be detected at all due to the high reactivity of our building block toward Grignard reagents. Instead, exclusive formation of tertiary amide with both fluorine atoms having been substituted was observed (see Supporting Information).^[^ ^58^ ^]^ Similar reactivity was observed in the case of *N*‐CHRF carbamoyl fluorides.

With synthetic access to *N*‐monofluoromethyl (thio)carbamates, formamides, alkynamides, and their derivatives successfully established, we next investigated their physical properties. To this end, we conducted a comparative analysis against the corresponding *N‐*CF_2_H, *N‐*CF_3_, and *N‐*Me analogues. Our log*P* calculations,^[^ ^59^ ^]^ which estimate a compound's lipophilicity—a key determinant of membrane permeability—revealed that the *N‐*CH_2_F analogue (−0.48) exhibited lower lipophilicity than both the *N‐*CF_3_ (0.82) and *N‐*CF_2_H (0.27) analogues, yet a modest increase compared to the *N‐*Me counterpart (−0.61).

Additionally, variable‐temperature ^1^H‐NMR spectroscopic studies examining the rotational barrier of the amide bond indicated that the *N*‐CH_2_F derivative, with a measured coalescence point of 60 °C, follows the previously observed trend of incrementally increased conformational flexibility upon fluorination. For comparison, for the nonfluorinated counterpart, coalescence was measured at 75 °C, while the difluoromethyl analogue drops to 40 °C (see Figure 2). This trend was further supported by IR carbonyl stretching frequencies (see Figure 2), reinforcing the unique electronic and steric effects introduced by fluorine substitution.

**Figure 2:**
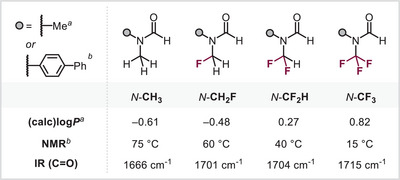
Physical properties and stability of *N*‐CH_2_F carbonyl derivatives.

In conclusion, we have developed a robust and efficient strategy for accessing a diverse chemical space of *N*‐CH_2_F and *N*‐CHRF carbonyl compounds in just two steps from readily available amines. This work marks the first successful synthesis of *N*‐monofluoromethyl derivatives of alkynamides, formamides, carbamates, and thiocarbamates—scaffolds of immense significance across fields ranging from oncology to agrochemistry. By leveraging *N*‐CH_2_F and *N*‐CHRF carbamoyl fluorides as strategic and versatile building blocks, our approach significantly expands the fluorinated molecular design toolkit, offering new opportunities for chemical and pharmaceutical exploration. Preliminary investigations into their physicochemical properties confirm that the *N*‐CH_2_F motif aligns with the established trends of *N*‐CF_3_ and *N*‐CF_2_H analogues, further highlighting the profound influence of fluorination on lipophilicity and conformational flexibility.

## Conflict of Interests

The authors declare no conflict of interest.

## Supporting information



Supporting Information

## Data Availability

The data that support the findings of this study are available in the Supporting Information of this article.
