# Supplementary material for: Access to N‐Monofluoromethylated (Thio)Carbamates, Formamides, Alkynamides, and Related Derivatives
Source: Angew Chem Int Ed Engl. 2025 May 10;64(28):e202505478. doi: 10.1002/anie.202505478 (PMC12232887; doi:10.1002/anie.202505478)
Supplement: Supplementary file 1 — Supporting Information [file ANIE-64-e202505478-s001.pdf]

Table of contents

1. General information 3

2. *N*-CH_2F carbamoyl fluorides 4

2.1. General procedure (GP-1) for the formation of *N*-CH_2F carbamoyl fluorides 4

2.2. Reaction optimization 5

2.2.1. Solvent and additives 5

2.2.2. Equivalents of reagents 6

3. *N*-CHRF carbamoyl fluorides 7

3.1. General procedure (GP-2) for the formation of *N*-CHRF carbamoyl fluorides 7

3.2. Reaction optimization 8

4. Preparation of $AgOCF_3$ stock solution 10

5. Characterization data of *N*-CH_2F carbamoyl fluorides 11

5.1. Enantioretention study 16

6. Characterization data of *N*-CHRF carbamoyl fluorides 18

7. Derivatizations of *N*-CH_2F and *N*-CHRF carbamoyl fluorides 22

7.1. General procedures 22

7.1.1. General procedure (GP-3) for *N*-CH_2F carbamate synthesis 22

7.1.2. General procedure (GP-4) for *N*-CHRF carbamate synthesis 22

7.1.3. General procedure (GP-5) for *N*-CH_2F and *N*-CHRF thiocarbamate synthesis[1] 22

7.1.4. General procedure (GP-6) for *N*-CH_2F formamide synthesis[3] 23

7.1.5. General procedure (GP-7) for *N*-CH_2F and *N*-CHRF formamide synthesis 23

7.1.6. General procedure (GP-8) for *N*-CH_2F carbamoyl azide and cyanide synthesis 23

7.1.7. General procedure (GP-9) for *N*-CHRF carbamoyl azide and cyanide synthesis 24

7.1.8. General procedure (GP-10) for *N*-CH_2F and *N*-CHRF alkynamide synthesis[4] 24

7.2. Characterization data of *N*-CH_2F and *N*-CHRF carbamates 25

7.3. Characterization data of *N*-CH_2F and *N*-CHRF thiocarbamates 26

7.4. Characterization data of *N*-CH_2F and *N*-CHRF formamides 27

7.5. Characterization data of *N*-CH_2F and *N*-CHRF carbamoyl azides 29

7.6. Characterization data of *N*-CH_2F and *N*-CHRF carbamoyl cyanides 30

7.7. Characterization data of *N*-CH_2F and *N*-CHRF alkynamides 31

7.7.1. Attempted synthesis of *N*-CH_2F amide[1] 34

7.7.2. Attempted synthesis of *N*-CH_2F urea[1] 36

8. Synthesis of starting materials 38

8.1. General procedure (GP-11) for the synthesis of aldimines 38

8.2. General procedure (GP-12) for the synthesis of aldimines 39

8.3. General procedure (GP-13) for the synthesis of TMS-protected alcohols 39

8.4. Characterization data of aldimines 40

8.5. Characterization data of TMS-protected alcohol 42

9. Property investigation 44

9.1. Computed log*P* values 44

9.1.1. XYZ coordinates of computed structure 44

9.2. NMR conformational studies 46

10. NMR spectra 48

10.1. *N*-CH_2F and *N*-CHRF carbamoyl fluorides 48

10.2. *N*-CH_2F and *N*-CHRF derivatives 86

10.3. Aldimines 120

10.4. TMS-Protected alcohol 131

11. References 132

1. General information

All reagents and starting materials were commercially available and used as received unless otherwise stated. AgF was purchased from Fluorochem (ChemPUR), BTC was purchased from TCI, TBDOTf was purchased from abcr, MeCN was purchased from Sigma Aldrich. Anhydrous solvents were dried using an Innovative Technology PS-MD-5 solvent purification system. Solvents used in work up and purification were distilled prior to use. Thin layer chromatography (TLC) was performed on Merck Kieselgel 60 F_{254} aluminium plates with unmodified silica and visualized either under UV light (λ = 254 nm) or stained with potassium permanganate or ceric ammonium molybdate (CAM) or phosphomolybdic acid (PMA). Flash column chromatography was performed with Merck silica gel 60 (35 – 70 mesh) under elevated pressure (0.3 – 0.5 bar) by compressed air.

All ^{1}H, ^{13}C and ^{19}F NMR spectra were recorded on Bruker Avance Neo 600, Varian VNMRS 600 or Varian VNMRS 400 spectrometers at ambient temperature. Chemical shifts (δ) are reported in parts per million (ppm) and were referenced to residual solvent peak (for ^{1}H and ^{13}C) or by the instrument internally after locking and shimming to the deuterated solvent (for ^{19}F). Coupling constants (J) are given in Hertz (Hz).
In some compounds rotamers were observed in the ^{19}F NMR, but not in the respective ^{1}H or ^{13}C NMR spectra. This is due to the larger spectral window for the ^{19}F nuclei. Because of this, the frequency separation between the exchanging sites in ^{19}F is larger than for the corresponding proton sites. This in turn can cause dynamic processes to be visible by ^{19}F NMR analysis but not ^{1}H NMR even though both belong to a single exchange process.[1]

Gas chromatography coupled with mass spectrometry (GC-MS) was performed on an Agilent Technologies 5975 series MSD mass spectrometer under electrospray ionization (EI) mode coupled with an Agilent Technologies 7820A gas chromatograph employing an Agilent 19091s-433 HP-5MS column (30 m x 0.25 μm x 0.25 μm).
High-resolution mass spectrometry (HRMS) was performed using a Thermo Scientific LTQ Orbitrap XL spectrometer (ESI), Finnigan MAT 95 (EI) or Bruker Maxis II LC-MS-System (APCI). Low-resolution masses of known compounds were extracted from their GC-MS chromatograms. All mass signals are given as mass-to-charge ratio [m/z], with the highest signal set to 100 % as the reference point for the intensities. The molecular ion fragment is indicated by an "M".

IR spectra were recorded on a PerkinElmar Spectrum 100 spectrometer with an UATR Diamond/KRS-5 crystal with attenuated total reflectance (ATR).

Melting points were measured with a Coesfeld melting point meter (MPM-H2) with visual detection and temperature increase of 1 °C/min.

2. *N*-CH_2F carbamoyl fluorides

2.1. General procedure (GP-1) for the formation of *N*-CH_2F carbamoyl fluorides

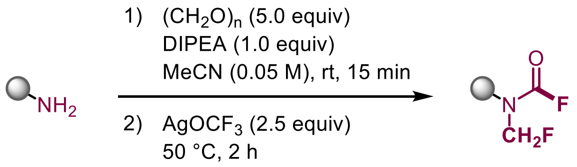

In an argon-filled glovebox, a 20 mL amber glass vial was loaded with the corresponding amine* (if solid) (0.50 mmol, 1.0 equiv), paraformaldehyde (75 mg, 2.5 mmol, 5.0 equiv), and MeCN (10 mL, 0.05 M). Subsequently, DIPEA (65 mg, 0.50 mmol, 1.0 equiv) was added, and the reaction mixture was stirred for 15 min at rt. After the indicated time, a stock solution of $AgOCF_3$ (1.0 M in MeCN, 1.25 mL, 1.25 mmol, 2.5 equiv) was added quickly and the vial was immediately secured tightly with a cap. The resulting solution was stirred for 2 h at 50 °C. After cooling down to room temperature, the reaction mixture was filtered through a short pad of Celite®, and the filtrate was concentrated *in vacuo*. The crude product was purified by flash column chromatography, eluting with 5 – 30 % Et_2O or 5 – 50 % EtOAc/pentane. To prevent decomposition, the obtained products were stored in a glass vial, under argon, at −30 °C in the freezer.

*In case of liquid amines, the starting material was added as a solution in MeCN (5 mL). If amines are commercially available as salts (i.e. R-NH_2•HCl), they were neutralized prior to the reaction. Extraction using DCM and saturated aqueous solution of $NaHCO_3$ was performed for neutralization. Organic phase was separated and dried over anhydrous $MgSO_4$. Solvents were removed under reduced pressure to obtain the free amine (R-NH_2).

2.2. Reaction optimization

2.2.1. Solvent and additives

Optimization of solvents and additives was carried out according to a modified general procedure GP-1 for the formation of *N*-CH_2F carbamoyl fluorides without pre-stirring.

In an argon-filled glovebox, a 2 mL amber glass vial was loaded with [1,1'-biphenyl]-4-amine (17 mg, 0.10 mmol, 1.0 equiv) and paraformaldehyde (15 mg, 0.50 mmol, 5.0 equiv). Then solvent (2 mL) was added followed by the corresponding additive A (0.10 mmol, 1.0 equiv) and additive B (0.10 mmol, 1.0 equiv). Subsequently, $AgOCF_3$ (0.20 mL, 0.20 mmol, 2.0 equiv, 1 M in MeCN) was added quickly and the vial was sealed. The reaction mixture was stirred for 2 h at 50 °C. After the indicated time, the mixture was cooled down to rt and 1-methoxy-4-(trifluoromethyl)benzene (2.5 μL 0.01767 mmol) was added as an internal standard. The crude mixture was analyzed by ^{19}F qNMR (Table S1).

Table S1. Screening of solvents and additives, and effect on yield.

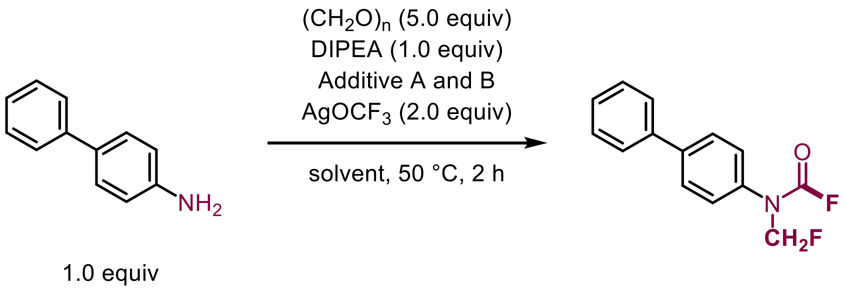

Entry	Solvent	Additive A	Additive B	Temperature / °C	NMR Yield / %[a]
1	MeCN	AgF	/	50	16%
2	DCM	AgF	/	50	0%
3	PhMe	AgF	/	50	0%
4	DMF	AgF	/	50	0%
5	MeCN	DIPEA	/	50	50%
6	MeCN	Et_3N	/	50	28%
7	MeCN	Pyridine	/	50	36%
8	MeCN	K_2CO_3	/	50	40%
9	MeCN	$BF_3{\cdot}OEt_2$	/	50	0%
10	MeCN	/	/	50	42%
11	MeCN	DIPEA	/	rt	33%
12	MeCN	DIPEA	/	50	43%
13	MeCN	DIPEA	AgF	50	36%
14	MeCN	DIPEA	$MgSO_4$	50	46%
15	MeCN	DIPEA	MS 4Å (1g/mmol)	50	40%

[a]Determined with 1-methoxy-4-(trifluoromethyl)benzene (2.5 μL, 0.01767 mmol) as internal standard (^{19}F qNMR, 25 s delay).

2.2.2. Equivalents of reagents

Carried out according to a modified general procedure GP-1 for the formation of *N*-CH_2F carbamoyl fluorides without pre-stirring.

In an argon-filled glovebox, a 2 mL amber glass vial was loaded with [1,1'-biphenyl]-4-amine (17mg, 0.10 mmol, 1.0 equiv) and paraformaldehyde. Then MeCN (2 mL) was added followed by DIPEA. Subsequently, $AgOCF_3$ (1 M stock solution in MeCN) was added quickly and the vial was sealed. The reaction mixture was stirred for 2 h at 50 °C. After cooling down to rt, 1-methoxy-4-(trifluoromethyl)benzene (2.5 μL 0.01767 mmol) was added as an internal standard and the crude mixture was analyzed by ^{19}F qNMR (Table S2).

Table S2. Screening of equivalents for paraformaldehyde, DIPEA and $AgOCF_3$, and effect on yield.[a]

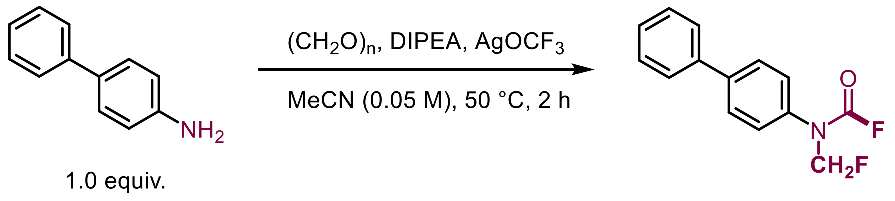

Entry	$(CH_2O)_n$ / equiv	DIPEA /equiv	$AgOCF_3$ / equiv	NMR Yield / %[a]
1	1	1	2	29%
2	3	1	2	47%
3	5	1	2	50%
4	8	1	2	57%
5	5	1	1.0	13%
6	5	1	1.5	24%
7	5	1	2.5	64%
8	5	1	5	58%
9	5	2	2	42%
10	5	2.5	2	44%
11	5	5	2.5	57%

[a]Determined with 1-methoxy-4-(trifluoromethyl)benzene (2.5 μL, 0.01767 mmol) as the internal standard (^{19}F qNMR, 25 s delay).

Note on importance of pre-stirring:

- It was observed that the yield of *N*-CH_2F carbamoyl fluoride was inconsistent and irreproducible when all the reagents were mixed from the start.
- Pre-stirring longer than 15 min resulted in significantly reduced yield (i.e. in case of 30 min the corresponding yield was 44% and in the case of 60 min 33%).

3. *N*-CHRF carbamoyl fluorides

3.1. General procedure (GP-2) for the formation of *N*-CHRF carbamoyl fluorides

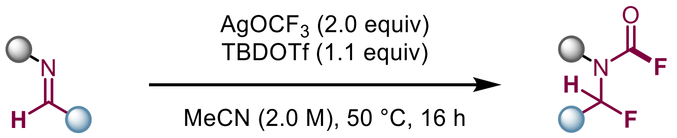

In an argon-filled glovebox, the corresponding imine (0.400 mmol, 1.00 equiv) was placed into an oven-dried 4 mL glass vial equipped with a magnetic stir bar and dissolved in anhydrous acetonitrile (0.20 mL, 2.0 M). After addition of *tert*-butyldimethylsilyl trifluoromethanesulfonate (TBDOTf, 0.440 mmol, 1.10 equiv, unless otherwise stated), a stock solution of $AgOCF_3$ (1.0 M in MeCN, 0.80 mmol, 2.0 equiv) was added, and the vial was immediately secured tightly with a cap. The resulting solution was stirred for 16 h at 50 °C. After the heterogeneous reaction mixture was cooled to room temperature, diethyl ether (Et_2O, ~2.5 mL) was added. The mixture was filtered through a short pad of Celite®, washed with Et_2O (~2.5 mL), and the filtrate was concentrated *in vacuo*. The residue was redissolved in a small amount of Et_2O (~1.0 mL), filtered through a short pad of silica, and washed with Et_2O (~2.0 mL). The solvent was evaporated under reduced pressure to obtain the desired product. To prevent decomposition, the obtained products were stored in a glass vial, under argon, at −30 °C in the freezer. *Note: The products were obtained in technical grade purity.*

3.2. Reaction optimization

DIPEA as an additive is not affecting the yield of the desired *N*-CHRF carbamoyl fluoride. However, the addition of Lewis acids is leading to a significant increase in yield at constant reaction conditions (Table S3).

Table S3. Additive screening for the reaction with aldimine **S1** and effect on yield.[a]

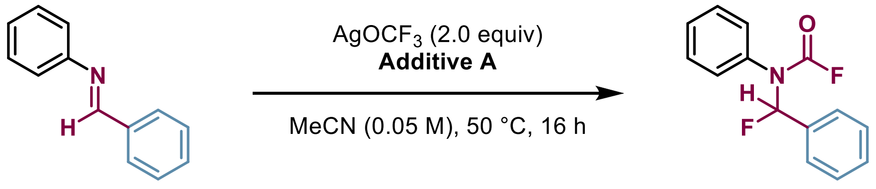

Entry	Additive **A**	**A** /equiv	Temperature / °C	Time / h	Conv. Imine/ %[b]	NMR Yield / %[c]
1	--	--	50	16	58	35
2	DIPEA	1.0	50	16	49	36
3	CSA	1.1	50	16	100	27
4	TFA	1.1	50	16	53	32
5	$Cu(OTf)_2$	1.1	50	16	100	53
6	TiF_4	1.1	50	16	100	61
7	TMSOTf	1.1	50	16	92	47
8	TBDOTf	1.1	50	16	84	64
9	$BF_3{\cdot}OEt_2$	1.1	50	16	80	60

Abbreviations: DIPEA = *N*,*N*-diisopropylethylamine, CSA = camphorsulfonic acid, TFA = trifluoroacetic acid, TMSOTf = trimethylsilyl trifluoromethanesulfonate, TBDOTf = *tert*-butyldimethylsilyl trifluoromethanesulfonate. [a]Conditions: 0.1 mmol of **S1** 0.2 mmol of $AgOCF_3$ (1.0 M stock solution), 2.0 mL of acetonitrile. [b]Conversion of aldimine **S1**; determined with 1-methoxy-4-(trifluoromethyl)benzene (2.5 µL) as internal standard (^1H qNMR). [c]Determined with 1-methoxy-4-(trifluoromethyl)benzene (2.5 µL) as internal standard (^{19}F qNMR).

Lower and higher loadings of $AgOCF_3$ are effective albeit they result in no change in yield of desired *N*-CHRF carbamoyl fluoride. The conversion of the imine starting material is increased by higher amounts of TBDOTf. The screening experiment revealed a bell curve for the product yield, with a maximum between 1.1 equiv and 1.8 equiv (Table S4). Higher loadings of TBDOTf resulted in formation of side species in higher amounts.

Table S4. Screening of equivalents for $AgOCF_3$ and TBDOTf, and effect on yield.[a]

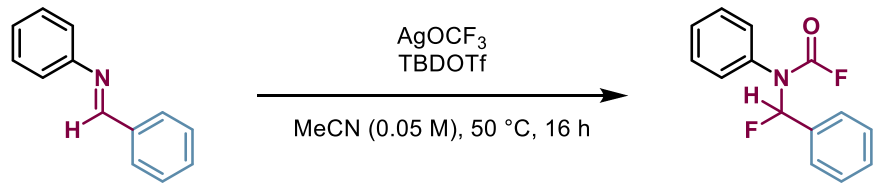

1.0 equiv

Entry	$AgOCF_3$ / equiv	TBDOTf /equiv	Conv. Imine/ %[b]	NMR Yield / %[c]
1	1.5	1.1	92	64
2	2.0	1.1	84	64
3	2.5	1.1	84	58
4	5.0	1.1	100	65
5	2.0	0.5	79	48
6	2.0	1.5	100	68
7	2.0	1.8	100	51
8	2.0	2.0	100	18
9	2.0	3.0	100	0

[a]Conditions: 0.1 mmol of **S1,** 2.0 mL of acetonitrile. [b]Conversion of aldimine **S1** determined with 1-methoxy-4-(trifluoromethyl)benzene (2.5 µL) as internal standard (^1H qNMR). [c]Determined with 1-methoxy-4-(trifluoromethyl)benzene (2.5 µL) as internal standard (^{19}F qNMR).

Higher concentration promotes the conversion of the imine to the product.

Table S5. Screening of solvents and effect of the concentration on yield.[a]

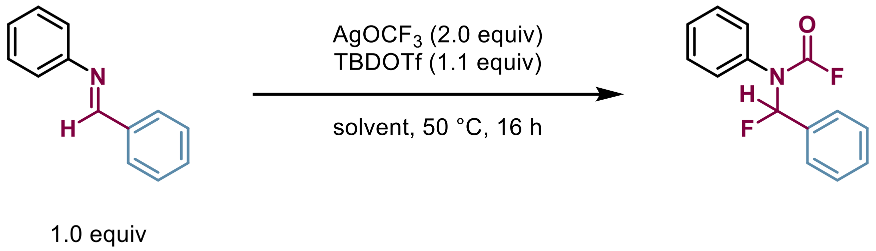

Entry	$AgOCF_3$ / equiv	Solvent	Molarity / M	Conv. Imine/ %[b]	NMR Yield / %[c]
1	2.0	MeCN	0.05	84	64
2	2.0	CH_2Cl_2	0.05	72	50
3	2.0	EtOAc	0.05	97	61
4	2.0	THF	0.05	51	23
5	2.0	1,4-dioxane	0.05	94	61
6	2.0	toluene	0.05	63	38
7	2.0	HFIP	0.05	54	0
8	2.0	MeCN	0.10[d]	95	67
9	2.0	MeCN	0.20[d]	98	76
10	2.0	MeCN	0.30[d]	>99	75[e]
11	2.0	MeCN	0.40[d]	>99	85[e]
12	2.0	MeCN	0.40[d]	98	85[e,f]
13	2.0	MeCN	0.50[d]	75	61[e,g]

Abbreviations: MeCN = acetonitrile, EtOAc = ethyl acetate, HFIP = hexafluoroisopropanol. [a]Conditions: 0.1 mmol of **S1**, 0.2 mmol of $AgOCF_3$ (1.0 M stock solution), 0.11 mmol of TBDOTf. [b]Conversion of aldimine **S1**; determined with 1-methoxy-4-(trifluoromethyl)benzene (2.5 µL) as internal standard (^1H qNMR). [c]Determined with 1-methoxy-4-(trifluoromethyl)benzene (2.5 µL) as internal standard (^{19}F qNMR). [d]The values for the molarity were determined by including the solvent amount of the $AgOCF_3$ stock solution. [e]The reaction was performed on a 0.4 mmol scale. [f]Without TBDOTf. [g]No additional MeCN (solvent) was added. The solvent amount resulted from the added $AgOCF_3$ stock solution.

4. Preparation of $AgOCF_3$ stock solution

In an argon-filled glovebox, a 20 mL amber vial was charged with silver fluoride (AgF, 2.28 g, 18 mmol, 9 equiv) and triphosgene (593.5 mg, 2 mmol, 1 equiv). Then, 6 mL of anhydrous acetonitrile was added quickly and the vial was immediately tightly secured with a cap. At this stage, bubbling and increase in temperature were observed. The reaction mixture was stirred vigorously overnight at room temperature and was then filtered through a syringe filter (PTFE, 0.45 µm) to obtain the $AgOCF_3$ as stock solution (~1.0 M, 5.5 mL) in acetonitrile (colorless or light-yellow). This solution was stored at -30 °C in darkness until further use.
Spectra were in agreement with previously reported data.[2]

5. Characterization data of *N*-CH_2F carbamoyl fluorides

[1,1'-Biphenyl]-4-yl(fluoromethyl)carbamic fluoride (1)

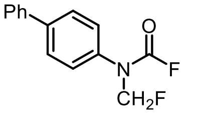

Prepared according to general procedure GP-1 and isolated as a pale-yellow solid (79.1 mg, 0.320 mmol, 64 %). ***R**$_f$* = 0.58 (10 % EtOAc/pentane). **^{1}H NMR** (600 MHz, Acetonitrile-*d$_3$*) δ 7.74 (d, *J* = 8.5 Hz, 2H), 7.67 (d, *J* = 7.7 Hz, 2H), 7.49 (t, *J* = 7.8 Hz, 4H), 7.41 (t, *J* = 7.4 Hz, 1H), 5.69 (d, *J* = 53.1 Hz, 2H). **^{19}F NMR** (564 MHz, Acetonitrile-*d$_3$*) δ -12.99 (s, 1F, rotamer A), -19.89 (s, 1F, rotamer B), -167.39 (t, *J* = 53.4 Hz, 1F, rotamer B), -172.67 (t, *J* = 55.8 Hz, 1F, rotamer A). **^{13}C NMR** (151 MHz, Acetonitrile-*d$_3$*) δ 146.8 (d, *J* = 291.6 Hz, rotamer A), 146.4 (d, *J* = 291.7 Hz, rotamer B), 142.4 (rotamer A), 142.1 (rotamer B), 140.5 (rotamer A), 139.7 (rotamer A or B), 138.6 (rotamer A or B), 130.0, 129.0 (rotamer A), 128.9 (rotamer B), 128.1 (rotamer B), 128.0 (rotamer A), 127.5, 90.9 (d, *J* = 200.5 Hz, rotamer A), 90.4 (d, *J* = 199.9 Hz, rotamer B). **HRMS** (EI): *m/z* [M]$^+$ calculated for $C_{14}H_{11}F_2NO$: 247.0803, found 247.0801. **IR** (neat, cm^{-1}): 3026, 2923, 1814, 1607, 1513, 1482, 1378, 1287, 975, 732, 685.

Gram-scale experiment: In an argon-filled glovebox, a 250 mL flask was loaded with [1,1'-biphenyl]-4-amine (1.014 g, 6.0 mmol, 1.0 equiv), paraformaldehyde (900 mg, 30.0 mmol, 5.0 equiv), and MeCN (120 mL, 0.05 M). Subsequently, DIPEA (775 mg, 1.0 mL, 6.0 mmol, 1.0 equiv) was added, and the reaction mixture was stirred for 15 min at rt. After the indicated time, a stock solution of $AgOCF_3$ (1.0 M in MeCN, 15 mL, 15 mmol, 2.5 equiv) was added quickly and the flask was immediately secured tightly with a septum. The resulting solution was stirred for 2 h at 50 °C. After cooling down to room temperature, the reaction mixture was filtered through over Celite®, and the filtrate was concentrated *in vacuo*. The product was isolated as pale yellow solid in 61% yield (904.3 mg, 3.66 mmol) after flash column chromatography, eluting with 10% EtOAc/pentane.

(Fluoromethyl)(3,4,5-trimethoxyphenyl)carbamic fluoride (2)

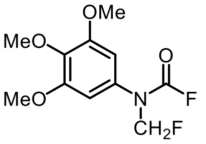

Prepared according to general procedure GP-1 and isolated as a pale-yellow solid (66.4 mg, 0.255 mmol, 51 %). ***R**$_f$* = 0.64 (20 % EtOAc/pentane, stain: $KMnO_4$). **^{1}H NMR** (600 MHz, Acetonitrile-*d$_3$*) δ 6.70 (s, 2H), 5.63 (d, *J* = 53.2 Hz, 2H), 3.80 (s, 6H), 3.74 (s, 3H). **^{19}F NMR** (564 MHz, Acetonitrile-*d$_3$*) δ -13.04 (s, 1F, rotamer A), -20.23 (s, 1F, rotamer B), -167.53 (t, *J* = 53.3 Hz, 1F, rotamer B), -172.78 (t, *J* = 53.3 Hz, 1F, rotamer A). **^{13}C NMR** (151 MHz, Acetonitrile-*d$_3$*) δ 154.6, 147.0 (d, *J* = 291.6 Hz, rotamer A), 146.4 (d, *J* = 300.9 Hz, rotamer B), 139.1, 136.0, 134.9, 105.7 (rotamer A), 105.2 (rotamer B), 90.7 (d, *J* = 199.9 Hz, rotamer A), 90.4 (d, *J* = 199.9 Hz, rotamer B). **HRMS** (EI): *m/z* [M]$^+$ calculated for $C_{11}H_{13}F_2NO_4$: 261.0807, found 261.0806. **IR** (neat, cm^{-1}): 2946, 2321, 1810, 1594, 1504, 1459, 1229, 1121, 965, 750, 693.

Methyl 4-((fluorocarbonyl)(fluoromethyl)amino)benzoate (3)

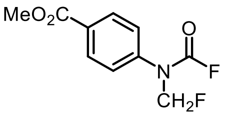

Prepared according to general procedure GP-1 and isolated as a pale-yellow oil (52.6 mg, 0.230 mmol, 46 %). ***R**$_f$* = 0.27 (10 % EtOAc/pentane). **^{1}H NMR** (600 MHz, Acetonitrile-*d$_3$*) δ 8.09 (d, *J* = 8.2 Hz, 2H), 7.52 (brs, 2H), 5.68 (d, *J* = 53.1 Hz, 2H), 3.89 (s, 3H). **^{19}F NMR** (564 MHz, Acetonitrile-*d$_3$*) δ -12.64 (s, 1F, rotamer A), -18.87 (s, 1F, rotamer B), -167.52 (t, *J* = 49.6 Hz, 1F, rotamer B), -172.56 (t, *J* = 54.8 Hz, 1F, rotamer A). **^{13}C NMR** (151 MHz, Acetonitrile-*d$_3$*) δ 166.7, 146.2 (d, *J* = 295.3 Hz), 131.5, 127.6, 126.8, 90.6 (d, *J* = 199.9 Hz, rotamer A), 90.1 (d, *J* = 209.1 Hz, rotamer B), 52.9. **HRMS** (EI): *m/z* [M]$^+$ calculated for

$C_{10}H_9F_2NO_3$: 229.0545, found 229.0544. **IR** (neat, cm^{-1}): 2956, 1808, 1720, 1608, 1375, 1278, 1109, 973, 763, 704.

(2-Fluoro-4-iodophenyl)(fluoromethyl)carbamic fluoride (4)

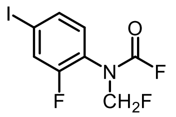

Prepared according to general procedure GP-1 and isolated as a yellow oil (62.5 mg, 0.200 mmol, 40 %). ***R**$_f$* = 0.53 (5 % EtOAc/pentane).
^{1}H NMR (600 MHz, Acetonitrile-*d$_3$*) δ 7.73 (dd, *J* = 9.3, 1.9 Hz, 1H), 7.68 (dt, *J* = 8.3, 1.4 Hz, 1H), 7.27 – 7.21 (m, 1H), 5.63 (d, *J* = 52.8 Hz, 2H, rotamer A), 5.60 (d, *J* = 52.9 Hz, 2H, rotamer B). **^{19}F NMR** (564 MHz, Acetonitrile-*d$_3$*) δ -14.37 (dd, *J* = 8.7, 6.1 Hz, 1F, rotamer A), -21.14 (d, *J* = 4.2 Hz, 1F, rotamer B), -119.79 (td, *J* = 9.0, 3.5 Hz, 1F, rotamer B), -121.01 (qd, *J* = 8.5, 3.5 Hz, 1F, rotamer A), -168.66 – -169.11 (m, 1F, rotamer B), -173.94 (tt, *J* = 52.8, 5.0 Hz, rotamer A). **^{13}C NMR** (151 MHz, Acetonitrile-*d$_3$*) δ 158.1 (d, *J* = 255.7 Hz, rotamer B), 158.0 (d, *J* = 255.3 Hz, rotamer A), 146.1 (d, *J* = 292.2 Hz, rotamer A), 145.5 (d, *J* = 295.6 Hz, rotamer B), 135.7 (d, *J* = 3.9 Hz, rotamer A+B), 131.2 (rotamer A+B), 127.0 (d, *J* = 22.4 Hz, rotamer B), 126.9 (d, *J* = 22.4 Hz, rotamer A), 124.5 (d, *J* = 21.5 Hz, rotamer A+B), 95.0 (d, *J* = 7.7 Hz, rotamer A), 94.9 (d, *J* = 7.8 Hz, rotamer B), 90.4 (d, *J* = 202.1 Hz, rotamer A), 89.8 (d, *J* = 202.4 Hz, rotamer B). **HRMS** (EI): *m/z* [M]$^+$ calculated for $C_8H_5ONF_3I$: 314.93624, found 314.93648. **IR** (neat, cm^{-1}): 1811, 1493, 1377, 1295, 1257, 984, 857, 751.

(4-Bromo-3-chlorophenyl)(fluoromethyl)carbamic fluoride (5)

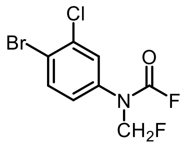

Prepared according to general procedure GP-1 and isolated as a pale-yellow oil (58.0 mg, 0.205 mmol, 41 %). ***R**$_f$* = 0.72 (10 % EtOAc/pentane, stain: $KMnO_4$).
^{1}H NMR (600 MHz, Acetonitrile-*d$_3$*) δ 7.79 (d, *J* = 8.6 Hz, 1H), 7.61 (s, 1H), 7.28 (brs, 1H), 5.63 (d, *J* = 53.0Hz, 2H). **^{19}F NMR** (564 MHz, Acetonitrile-*d$_3$*) δ -12.38 (s, 1F, rotamer A), -19.59 (s, 1F, rotamer B), -167.92 (t, *J* = 53.1 Hz, 1F, rotamer B), -173.03 (t, *J* = 53.0 Hz, 1F, rotamer A). **^{13}C NMR** (151 MHz, Acetonitrile-*d$_3$*) δ 146.1 (d, *J* = 291.0 Hz), 140.6, 139.5, 135.6, 129.8 (rotamer A), 129.0 (rotamer B), 127.9 (rotamer A), 127.2 (rotamer B), 123.3 (rotamer A), 122.7 (rotamer B), 90.4 (d, *J* = 201.1 Hz, rotamer A), 89.9 (d, *J* = 200.5 Hz, rotamer B). **HRMS** (EI): *m/z* [M]$^+$ calculated for $C_8H_5F_2NOBrCl$: 282.9205, found 282.9203. **IR** (neat, cm^{-1}): 2327, 2085, 1809, 1587, 1467, 1371, 1288, 1251, 1150, 980, 819, 754, 709.

(Fluoromethyl)(2-isopropylphenyl)carbamic fluoride (6)

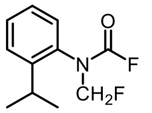

Prepared according to general procedure GP-1 and isolated as a pale-yellow oil (78.8 mg, 0.370 mmol, 74 %). ***R**$_f$* = 0.55 (5 % EtOAc/pentane, stain: $KMnO_4$).
^{1}H NMR (600 MHz, Acetonitrile-*d$_3$*) δ 7.49 – 7.43 (m, 2H), 7.29 (brs, 2H), 5.69 (dd, *J* = 53.1, 8.4 Hz, 1H, rotamer A H_a), 5.65 (dd, *J* = 53.7, 9.0 Hz, 1H, rotamer B, H_a), 5.51 (dd, *J* = 53.1, 8.4 Hz, 1H, rotamer A, H_b), 5.47 (dd, *J* = 53.1, 9.0 Hz, 1H, rotamer B, H_b), 3.09 – 3.05 (m, 1H), 1.19 (s, 3H), 1.18 (s, 3H). **^{19}F NMR** (564 MHz, Acetonitrile-*d$_3$*) δ -12.11 (s, 1F, rotamer A), -21.04 (s, 1F, rotamer B), -169.12 – -169.39 (m,1F, rotamer B), -174.57 – -174.82 (m, 1F, rotamer A). **^{13}C NMR** (151 MHz, Acetonitrile-*d$_3$*) δ 147.3 (d, *J* = 290.4 Hz), 147.4, 147.2, 146.6 (d, *J* = 297.2 Hz), 137.6, 136.4, 131.0 (rotamer A), 130.9 (rotamer B), 129.0 (rotamer A), 129.0 (rotamer B), 128.2 (rotamer A), 128.2 (rotamer B), 128.1 (rotamer B), 128.1 (rotamer A), 91.1 (d, *J* = 200.5 Hz, rotamer A), 90.6 (dd, *J* = 201.1, 3.1 Hz, rotamer B), 28.7 (rotamer A), 28.7 (rotamer B), 24.0, 23.8 (rotamer B), 23.7 (rotamer A). **HRMS** (EI): *m/z* [M]$^+$ calculated for $C_{11}H_{13}F_2NO$: 213.0959, found 213.0960. **IR** (neat, cm^{-1}): 2970, 1807, 1736, 1492, 1375, 1250, 979, 759, 720.

(Fluoromethyl)(2-methoxy-5-methylphenyl)carbamic fluoride (7)

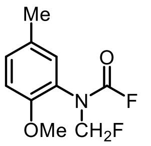

Prepared according to general procedure GP-1 and isolated as an orange oil (62.4 mg, 0.290 mmol, 58 %). R_f = 0.40 (5 % EtOAc/pentane).

^{1}H NMR (600 MHz, Acetonitrile-d_3) δ 7.26 – 7.22 (m, 1H), 7.20 – 7.14 (m, 1H), 7.04 – 6.98 (m, 1H), 5.88 – 5.25 (m, 2H), 3.82 (s, 3H, rotamer A), 3.81 (s, 3H, rotamer B), 2.29 (s, 3H). **^{19}F NMR** (564 MHz, Acetonitrile-d_3) δ -15.83 (d, J = 5.5 Hz, 1F, rotamer A), -21.54 (d, J = 4.1 Hz, 1F, rotamer B), -169.38 (td, J = 53.5, 4.1 Hz, 1F, rotamer B), -174.29 (td, J = 53.2, 5.5 Hz, rotamer A, 1F). **^{13}C NMR** (151 MHz, Acetonitrile-d_3) δ 153.5, 153.4, 147.5 (d, J = 290.2 Hz), 146.1 (d, J = 297.8 Hz), 131.8, 131.8, 131.6, 130.1, 129.7, 128.1, 127.3, 113.5, 113.2, 90.7 (d, J = 200.0 Hz), 90.1 (dd, J = 200.7, 3.2 Hz), 56.7, 56.6, 20.2. **HRMS** (EI): m/z [M]$^+$ calculated for $C_{10}H_{11}O_2NF_2$: 215.0752, found 215.0752. **IR** (neat, cm^{-1}): 1804, 1512, 1371, 1260, 975, 756.

(4-Bromobenzyl)(fluoromethyl)carbamic fluoride (8)

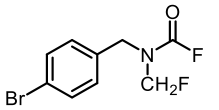

Prepared according to general procedure GP-1 and isolated as a yellow oil (46.1 mg, 0.175 mmol, 35 %). R_f = 0.67 (20 % EtOAc/pentane).

^{1}H NMR (600 MHz, Acetonitrile-d_3) mixture of rotamers δ 7.57 – 7.52 (m, 2H), 7.29 (d, J = 8.2 Hz, 2H, rotamer A), 7.27 (d, J = 8.1 Hz, 2H, rotamer B), 5.47 (d, J = 53.4 Hz, 2H, rotamer B), 5.42 (d, J = 53.6 Hz, 2H, rotamer A), 4.58 (s, 2H). **^{19}F NMR** (564 MHz, Acetonitrile-d_3) δ -17.89 (d, J = 6.2 Hz, 1F, rotamer B), -21.99 (d, J = 4.6 Hz, 1F, rotamer A), -170.36 (td, J = 53.6, 4.7 Hz, 1F, rotamer A), -175.18 (td, J = 53.3, 6.2 Hz, 1F, rotamer B). **^{13}C NMR** (151 MHz, Acetonitrile-d_3) δ 148.2 (d, J = 293.4 Hz, rotamer B), 147.4 (d, J = 298.2 Hz, rotamer A), 136.5 (rotamer B), 136.3 (rotamer A), 132.7 (rotamer B), 132.6 (rotamer A), 131.0 (rotamer A), 130.6 (rotamer B), 122.4 (rotamer A), 122.4 (rotamer B), 89.9 (d, J = 197.1 Hz, rotamer B), 88.8 (dd, J = 198.0, 4.1 Hz, rotamer A), 52.5 (rotamer A), 51.9 (rotamer B). **HRMS** (EI): m/z [M]$^+$ calculated for $C_9H_8ONBrF_2$: 262.9752, found 262.9751. **IR** (neat, cm^{-1}): 1799, 1487, 1401, 1251, 1108, 1010, 965, 824, 792, 757.

(2-Bromophenethyl)(fluoromethyl)carbamic fluoride (9)

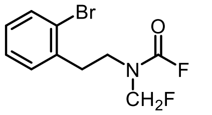

Prepared according to general procedure GP-1 and isolated as a colorless oil (45.6 mg, 0.165 mmol, 33 %). R_f = 0.34 (3 % EtOAc/pentane, stain: $KMnO_4$).

^{1}H NMR (600 MHz, Acetonitrile-d_3) δ 7.61 – 7.58 (m, 1H), 7.35 – 7.31 (m, 2H), 7.19 – 7.16 (m, 1H), 5.40 (d, J = 53.5 Hz, 2H, rotamer B), 5.36 (d, J = 53.8 Hz, 2H, rotamer A), 3.68 – 3.64 (m, 2H), 3.11 – 3.07 (m, 2H). **^{19}F NMR** (564 MHz, Acetonitrile-d_3) δ -19.79 (s, 1F, rotamer B), -21.80 (s, 1F, rotamer A), -169.28 (t, J = 48.6 Hz, 1F, rotamer A), -174.44 (t, J = 53.2 Hz, 1F, rotamer B) **^{13}C NMR** (151 MHz, Acetonitrile-d_3) δ 148.2 (d, J = 289.7 Hz, rotamer A), 146.9 (d, J = 298.4 Hz, rotamer B), 138.3 (rotamer A), 138.1 (rotamer B), 133.8 (rotamer B), 133.8 (rotamer A), 132.4 (rotamer B), 132.3 (rotamer A), 129.8 (rotamer B), 129.7 (rotamer A), 128.9 (rotamer B), 128.9 (rotamer A), 125.0 (rotamer A), 125.0 (rotamer B), 90.0 (d, J = 196.5 Hz, rotamer B), 89.0 (dd, J = 196.9, 4.2 Hz, rotamer A) 49.6 (rotamer A), 48.9 (rotamer B), 36.4 (rotamer B), 35.3 (rotamer A). **HRMS** (EI): m/z [M]$^+$ calculated for $C_{10}H_{10}F_2NOBr$: 276.9908, found 276.9906. **IR** (neat, cm^{-1}): 2944, 2323, 1800, 1417, 1249, 1177, 1099, 1026, 968, 752, 660.

(Fluoromethyl)(3-(4-fluorophenyl)-3-(furan-2-yl)propyl)carbamic fluoride (10)

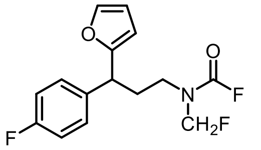

Prepared according to general procedure GP-1 and isolated as a pale-yellow oil (68.2 mg, 0.230 mmol, 46 %). R_f = 0.39 (8 % EtOAc/pentane, stain: $KMnO_4$).

^{1}H NMR (600 MHz, Acetonitrile-d_3) δ 7.39 (brs, 1H), 7.30 – 7.28 (m, 2H), 7.07 (t, J = 8.8 Hz, 2H), 6.36 – 6.35 (m, 1H), 6.21 – 6.19 (m, 1H), 5.38 (d, J = 53.5 Hz, 2H, rotamer B), 5.34 (d, J = 53.8 Hz, 2H, rotamer A), 4.08 (m, 1H), 3.42 – 3.33 (m, 2H), 2.44 – 2.41 (m, 1H), 2.24

– 2.21 (m, 1H). **^{19}F NMR** (564 MHz, Acetonitrile-*d$_3$*) δ -19.65 – -19.79 (m, 1F, rotamer B), -21.59 – -21.72 (m, 1F, rotamer A), -117.65 (s, 1F), -169.17 – -169.33 (m, 1F, rotamer A), -174.26 – -174.45 (m, 1F, rotamer B). **^{13}C NMR** (151 MHz, Acetonitrile-*d$_3$*) δ 162.6 (d, *J* = 243.0 Hz, rotamer A+B), 157.6 (rotamer A), 157.5 (rotamer B), 148.2 (d, *J* = 292.2 Hz, rotamer B), 146.9 (dd, *J* = 294.7, 4.3 Hz, rotamer A), 142.9 (rotamer B), 142.8 (rotamer A), 139.0 (d, *J* = 3.6 Hz, rotamer A), 138.9 (d, *J* = 3.0 Hz, rotamer B), 130.4 (d, *J* = 6.1 Hz, rotamer A), 130.3 (d, *J* = 6.2 Hz, rotamer B), 116.3 (d, *J* = 21.5 Hz, rotamer B), 116.2 (d, *J* = 21.5 Hz, rotamer A), 111.3 (rotamer A+B), 106.6 (rotamer A+B), 89.9 (d, *J* = 196.2 Hz), 88.9 (dd, *J* = 196.8, 4.3 Hz) 48.2 (rotamer A), 47.6 (rotamer B), 42.2 (rotamer A+B), 34.5 (rotamer B), 33.5 (rotamer A). **HRMS** (EI): *m/z* $[M]^+$ calculated for $C_{15}H_{14}F_3NO_2$: 297.0971, found 297.0970. **IR** (neat, cm^{-1}): 2948, 2326, 1800, 1507, 1418, 1226, 1170, 967, 818, 736.

***tert*-Butyl 4-((fluorocarbonyl)(fluoromethyl)amino)piperidine-1-carboxylate (11)**

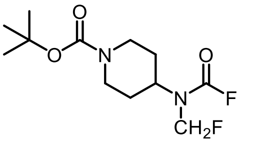

Prepared according to general procedure GP-1 and isolated as a white solid (62.6 mg, 0.225 mmol, 45 %). ***R$_f$*** = 0.45 (20 % EtOAc/pentane, stain: $KMnO_4$). **M.p.**: 83 – 85 °C.

^{1}H NMR (600 MHz, Acetonitrile-*d$_3$*) δ 5.45 (d, *J* = 54.5 Hz, 2H, rotamer B), 5.39 (d, *J* = 53.7 Hz, 2H, rotamer A), 4.16 – 4.13 (m, 2H), 3.92 – 3.86 (m, 1H), 2.76 (brs, 2H), 1.82 – 1.80 (m, 2H), 1.67 – 1.58 (m, 2H), 1.43 (s, 9H). **^{19}F NMR** (564 MHz, Acetonitrile-*d$_3$*) δ -19.26 (s, 1F, rotamer A), -19.60 (s, 1F, rotamer B), -166.78 (t, *J* = 63.2 Hz, 1F, rotamer A), -171.41 (t, *J* = 54.7 Hz, 1F, rotamer B). **^{13}C NMR** (151 MHz, Acetonitrile-*d$_3$*) δ 155.2, 146.7 (d, *J* = 294.1 Hz), 86.3 (d, *J* = 195.6 Hz, rotamer B), 85.4 (d, *J* = 194.4 Hz, rotamer A), 80.0, 57.2, 56.7, 44.1, 31.5, 30.5, 28.4. **HRMS** (ESI): *m/z* $[M + Na]^+$ calculated for $C_{12}H_{20}F_2N_2O_3Na$: 301.1334, found 301.1339. **IR** (neat, cm^{-1}): 2956, 2227, 1796, 1678, 1426, 1366, 1257, 1218, 1166, 1140, 1066, 1021, 794, 672.

Methyl *N*-(fluorocarbonyl)-*N*-(fluoromethyl)- *L*-phenylalaninate (12)

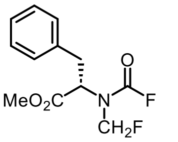

Prepared according to general procedure GP-1 and isolated as a green-yellow oil (74.5 mg, 0.290 mmol, 58 %). ***R$_f$*** = 0.33 (13 % EtOAc/pentane, stain: $KMnO_4$).

^{1}H NMR (600 MHz, Acetonitrile-*d$_3$*) δ 7.34 – 7.27 (m, 5H), 5.43 (dd, *J* = 53.0, 8.9 Hz, 1H, rotamer B, H_a), 5.39 (dd, *J* = 53.8, 9.6 Hz, 1H, rotamer A, H_a), 5.14 (dd, *J* = 52.7, 9.5 Hz, 1H, rotamer A, H_b), 5.11 (dd, *J* = 53.0, 11.7 Hz, 1H, rotamer B, H_b), 4.75 – 4.72 (m, 1H, rotamer A), 4.70 – 4.67 (m, 1H, rotamer B), 3.75 (s, 3H, rotamer B), 3.73 (s, 3H, rotamer A), 3.46 – 3.40 (m, 1H), 3.19 – 3.15 (m, 1H ,rotamer A), 3.12 – 3.07 (m, 1H, rotamer B). **^{19}F NMR** (564 MHz, Acetonitrile-*d$_3$*) δ -161.2 (brs, 1F, rotamer B), -19.66 – -19.68 (m, 1F, rotamer A), - 169.81 – 170.04 (m, 1F, rotamer A), -175.17 – -175.39 (m, 1F, rotamer B). **^{13}C NMR** (151 MHz, Acetonitrile-*d$_3$*) δ 170.5 (rotamer B), 170.3 (rotamer A), 147.7 (d, *J* = 294.2 Hz, rotamer B), 146.8 (dd, *J* = 296.1, 3.9 Hz, rotamer A), 137.5 (rotamer A), 137.4 (rotamer B), 130.1 (rotamer B), 130.1 (rotamer A), 129.6 (rotamer B), 129.5 (rotamer A), 128.0 (rotamer B), 127.9 (rotamer A), 89.5 (d, *J* = 199.9 Hz, rotamer A), 87.9 (dd, *J* = 200.4, 4.2 Hz, rotamer B), 63.8 (rotamer B), 63.5 (rotamer A), 53.6 (rotamer A), 53.4 (rotamer B), 36.8 (rotamer A), 35.7 (rotamer B). **HRMS** (ESI): *m/z* $[M + Na]^+$ calculated for $C_{12}H_{13}F_2NO_3Na$: 280.0755, found 280.0760. **IR** (neat, cm^{-1}): 2956, 2327, 2113, 1805, 1745, 1423, 1340, 1249, 1178, 1113, 974, 753.

(1-(2,6-Dimethylphenoxy)propan-2-yl)(fluoromethyl)carbamic fluoride (13)

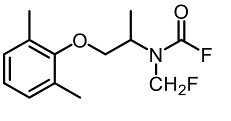

Prepared according to general procedure GP-1 and isolated as a yellow oil (76.7 mg, 0.300 mmol, 60 %). ***R$_f$*** = 0.50 (5 % EtOAc/pentane).

^{1}H NMR (400 MHz, Acetonitrile-*d$_3$*) δ 7.02 (d, *J* = 7.5 Hz, 2H), 6.96 – 6.90 (m, 1H), 5.61 (d, *J* = 53.4 Hz, 2H, rotamer B), 5.55 (d, *J* = 53.5 Hz, 2H, rotamer A), 4.41 (h, *J* = 6.9 Hz, 1H), 3.96 – 3.73 (m,

2H), 2.22 (s, 6H, rotamer A), 2.22 (s, 6H, rotamer B), 1.43 (d, *J* = 7.2 Hz, 3H, rotamer A), 1.40 (d, *J* = 6.9 Hz, 3H, rotamer B). **^{19}F NMR** (376 MHz, Acetonitrile-d_3) δ -18.54 (1F, rotamer A), -18.87 (1F, rotamer B), -167.14 (td, *J* = 53.6, 5.2 Hz, 1F, rotamer A), -172.12 (td, *J* = 53.5, 6.0 Hz, 1F, rotamer B). **^{13}C NMR** (151 MHz, Acetonitrile-d_3) δ 156.0 (rotamer A) , 155.9 (rotamer B), 148.8 (d, *J* = 292.8 Hz, rotamer B), 147.0 (dd, *J* = 295.1, 4.2 Hz, rotamer A), 131.7 (rotamer A+B), 129.9 (rotamer A+B), 125.2 (rotamer B), 125.2 (rotamer A), 86.7 (d, *J* = 196.5 Hz, rotamer B), 86.1 (dd, *J* = 197.4, 4.7 Hz, rotamer A), 73.8 (rotamer B), 73.1 (rotamer A), 55.5 (rotamer A), 54.7 (rotamer B), 16.3 (rotamer A+B), 15.9 (rotamer B), 15.4 (rotamer A). **HRMS** (ESI): *m/z* [M + Na]$^+$ calculated for $C_{13}H_{17}O_2NF_2Na$: 280.1120, found 280.1117. **IR** (neat, cm^{-1}): 1800, 1474, 1414, 1250, 1198, 1088, 1028, 968, 765.

((*1R,2R,3R,5S,7S*)-2,5-dimethyladamantan-1-yl)(fluoromethyl)carbamic fluoride (14)

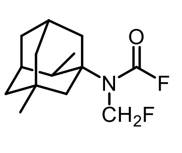

Prepared according to general procedure GP-1 and isolated as a pale-yellow oil (56.2 mg, 0.218 mmol, 44 %). ***R_f*** = 0.66 (10 % Et_2O/pentane).
^{1}H NMR (600 MHz, Acetonitrile-d_3) δ 5.49 (d, *J* = 53.7 Hz, 1H), 2.21 (h, *J* = 3.2 Hz, 1H), 1.96 – 1.94 (m, 2H), 1.76 (d, *J* = 11.6 Hz, 2H), 1.72 (d, *J* = 11.8 Hz, 2H), 1.42 – 1.37 (m, 2H), 1.35 – 1.30 (m, 2H), 1.20 – 1.17 (m, 2H), 0.88 (s, 6H). **^{19}F NMR** (564 MHz, Acetonitrile-d_3) δ -6.22 (s, 1F), -162.42 (brs, 1F). **^{13}C NMR** (151 MHz, Acetonitrile-d_3) δ 85.5 (d, *J* = 196.2 Hz), 61.4, 50.6, 46.5, 42.8, 39.0, 33.6, 31.4, 30.4. **MS** (70eV, EI): *m/z* (%): 257.100 (8.27), 242.063 (7.30), 205.084 (4.80), 190.084 (15.87), 166.000 (16.42), 164.100 (43.35), 163.175 (100.00), 147.085 (14.24), 134.000 (9.82), 121.064 (10.05), 107.085 (74.67), 93.055 (13.78), 79.000 (10.10). **IR** (neat, cm^{-1}): 2905, 2862, 2249, 1798, 1455, 1367, 1340, 1234, 1204, 1090, 1051, 955, 763.
Note: Due to low intensity, carbon signal of COF was not observed.

[1,1'-Biphenyl]-4-yl(fluoromethyl-*d*2)carbamic fluoride (15)

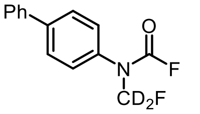

Prepared according to general procedure GP-1 with 60 minutes pre-stirring of imine solution before adding $AgOCF_3$. The desired product **14** was isolated as a pale-yellow solid (85.9 mg, 0.345 mmol, 69 %). ***R_f*** = 0.64 (10 % EtOAc/pentane).
^{1}H NMR (600 MHz, Acetonitrile-d_3) δ 7.75 (d, *J* = 8.5 Hz, 2H), 7.67 (d, *J* = 7.9 Hz, 2H), 7.49 (t, *J* = 7.5 Hz, 4H), 7.41 (t, *J* = 7.5 Hz, 1H). **^{19}F NMR** (564 MHz, Acetonitrile-d_3) δ -13.06 (s, 1F, rotamer A), -19.95 (s, 1F, rotamer B), -168.65 (s, 1F, rotamer B), -173.90 (s, 1F, rotamer A). **^{13}C NMR** (151 MHz, Acetonitrile-d_3) δ 148.4 (d, *J* = 228.2 Hz, rotamer A), 146.6 (d, *J* = 293.4 Hz, rotamer B), 142.4, 140.5, 138.5, 130.3, 129.0 (rotamer A), 128.9 (rotamer B), 128.1 (rotamer B), 128.0 (rotamer A), 127.5. **HRMS** (EI): *m/z* [M]$^+$ calculated for $C_{14}H_9F_2NOD_2$: 249.0928, found 249.0926. **IR** (neat, cm^{-1}): 2924, 2303, 1811, 1485, 1358, 1290, 1174, 1051, 933, 758, 726, 696.
Note: Due to low intensity, carbon signal of CD_2F was not observed.

5.1. Enantioretention study

Compound **12** was synthesized in racemic form using the same method as for the enantiopure compound described in the experimental section and spectroscopic data match the corresponding enantiopure compound.

Methyl *N*-(fluorocarbonyl)-*N*-(fluoromethyl)- *L*-phenylalaninate (12):

Column: Chiralpak IA (250x4,6) mm; *Mobile phase*: n-Hexane/iPrOH 8:2;
Temperature in °C: 30.0; Flow in ml/min: 1.00; Pressure in bar: 39.5

Racemic HPLC trace of 12

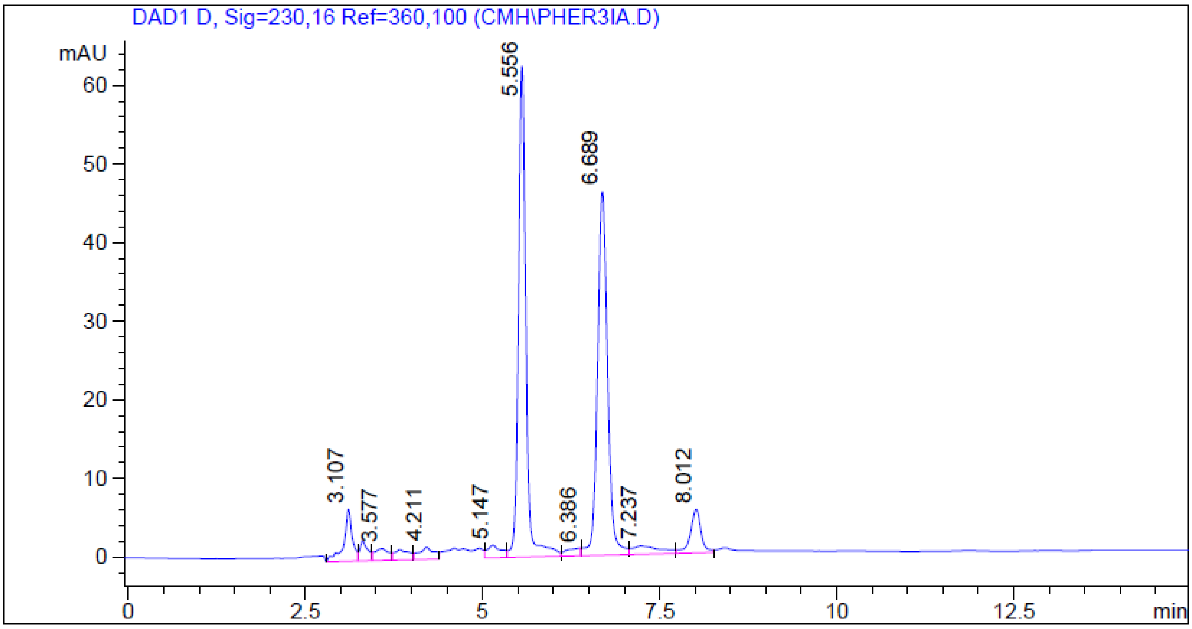

Enantiopure HPLC trace of 12

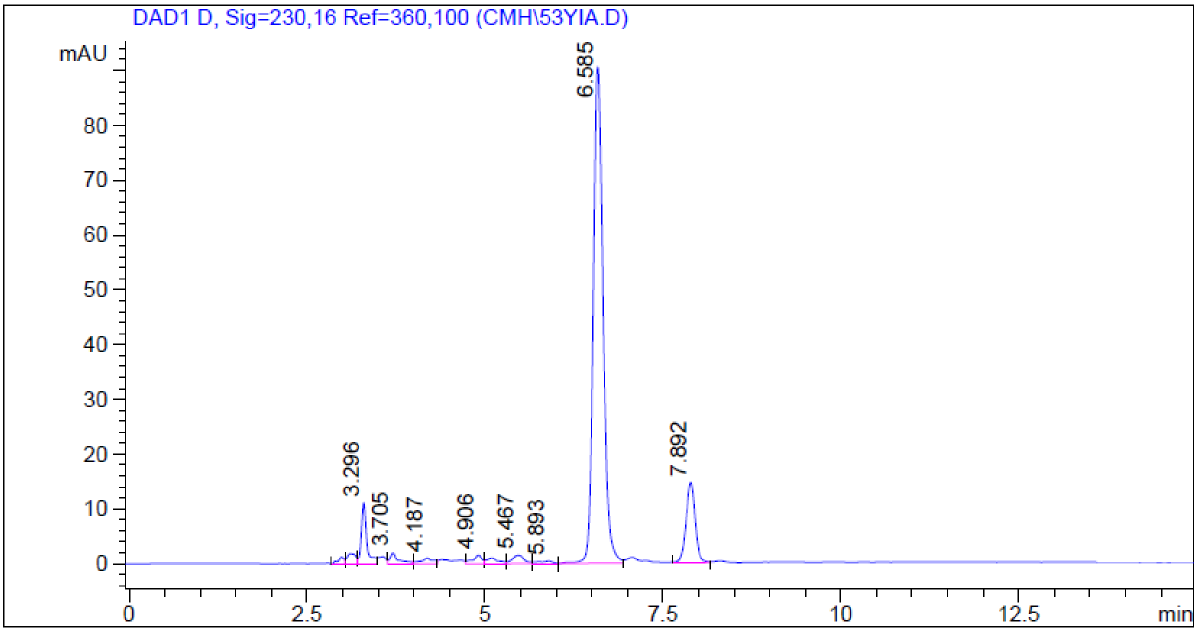

Overlay of HPLC traces of 12

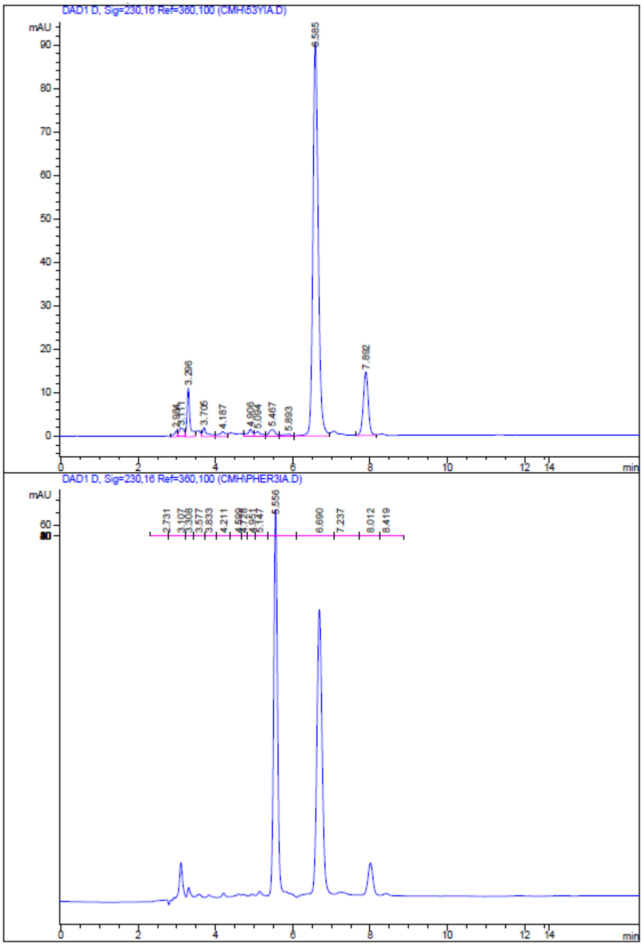

6. Characterization data of *N*-CHRF carbamoyl fluorides

(Fluoro(phenyl)methyl)(phenyl)carbamic fluoride (16)

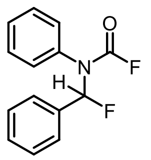

Prepared according to general procedure GP-2 from *N*,1-diphenylmethanimine (**S1**, 72.5 mg, 0.400 mmol, 1.00 equiv). The title compound **16** was obtained as a white solid (92.0 mg, 0.372 mmol, 93 %). R_f = 0.38 (pentane/EtOAc 19:1 *v/v*). **M.p.**: 58 – 59 °C.

^{1}H NMR (600 MHz, Acetonitrile-d_3) δ 7.39 (d, *J* = 49.2 Hz, 1H), 7.30 – 7.24 (m, 8H), 7.16 – 7.09 (m, 2H). **^{19}F NMR** (564 MHz, Acetonitrile-d_3) δ -10.38 (s, 1F, rotamer A), -20.17 (s, 1F, rotamer B), -149.20 (d, *J* = 49.5 Hz, 1F, rotamer A), -143.91 (s, 1F, rotamer B). **^{13}C NMR** (151 MHz, Acetonitrile-d_3) δ 147.2 (d, *J* = 293.1 Hz), 135.7, 135.0 (d, *J* = 27.1 Hz), 130.1, 130.0, 129.9, 129.8, 129.2 (d, *J* = 1.3 Hz), 126.9 (d, *J* = 7.5 Hz), 97.6 (d, *J* = 205.3 Hz). **MS** (70 eV, EI): *m/z* (%): 247 (16) [M$^+$], 180 (11), 119 (6), 109 (100), 83 (7), 77 (7). **HRMS** (ESI): *m/z* [M + Na]$^+$ calculated for $C_{14}H_{11}F_2NNaO$ 270.0701, found: 270.0704. **IR** (neat, cm^{-1}): 3036, 1799, 1593, 1491, 1396, 1296, 1256, 975, 850, 740, 692.

(Fluoro(phenyl)methyl)(4-(trifluoromethyl)phenyl)carbamic fluoride (17)

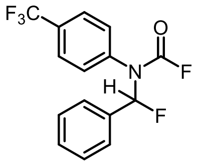

Prepared according to general procedure GP-2 from 1-phenyl-*N*-(4-(trifluoromethyl)phenyl)methanimine (**S2**, 99.7 mg, 0.400 mmol, 1.00 equiv). The title compound **17** was obtained as a white solid (116 mg, 0.367 mmol, 92 %). R_f = 0.47 (pentane/EtOAc 19:1 *v/v*). **M.p.**: 83 – 85 °C.

^{1}H NMR (600 MHz, Acetonitrile-d_3) δ 7.59 (d, *J* = 8.3 Hz, 2H), 7.43 (d, *J* = 49.6 Hz, 1H), 7.34 (d, *J* = 8.3 Hz, 2H), 7.28 (s, 5H). **^{19}F NMR** (564 MHz, Acetonitrile-d_3) δ -9.71 (s, 1F, rotamer A), -19.79 (s, 1F, rotamer B), -149.50 (s, 1F, rotamer A), -144.37 (s, 1F, rotamer B). **^{13}C NMR** (151 MHz, Acetonitrile-d_3) δ 146.6 (d, *J* = 294.2 Hz), 139.5, 134.6 (d, *J* = 26.9 Hz), 131.1 (q, *J* = 33.2 Hz), 131.0, 130.3, 129.3 (d, *J* = 1.7 Hz), 127.0 (q, *J* = 3.7 Hz), 126.9 (d, *J* = 7.5 Hz), 124.7 (q, *J* = 271.3 Hz), 97.5 (d, *J* = 206.4 Hz). **MS** (70 eV, EI): *m/z* (%): 315 (7) [M$^+$], 248 (15), 145 (8), 109 (100), 83 (10). **IR** (neat, cm^{-1}): 3072, 1804, 1614, 1517, 1401, 1316, 1260, 1169, 1117, 1063, 989, 853, 799, 748, 725, 695, 656.

Methyl 4-((fluoro(*o*-tolyl)methyl)(fluorocarbonyl)amino)benzoate (18)

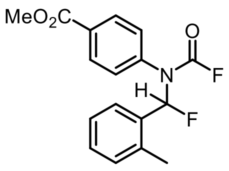

Prepared according to general procedure GP-2 from methyl 4-((2-methylbenzylidene)amino)benzoate (**S3**, 50.7 mg, 0.200 mmol, 1.00 equiv). The title compound **18** was obtained as a light-yellow oil (57.7 mg, 0.181 mmol, 90 %). R_f = 0.29 (pentane/EtOAc 19:1 *v/v*).

^{1}H NMR (600 MHz, Acetonitrile-d_3) δ 7.88 – 7.82 (m, 2H), 7.49 (d, *J* = 48.6 Hz, 1H), 7.19 (d, *J* = 8.1 Hz, 2H), 7.18 – 7.15 (m, 2H), 7.04 (d, *J* = 7.8 Hz, 1H), 7.00 – 6.93 (m, 1H), 3.83 (s, 3H), 2.38 (s, 3H). **^{19}F NMR** (564 MHz, Acetonitrile-d_3) δ -9.73 (s, 1F, rotamer A), -20.87 (s, 1F, rotamer B), -144.65 (s, 1F, rotamer B), -150.01 (s, 1F, rotamer A). **^{13}C NMR** (151 MHz, Acetonitrile-d_3) δ 166.6, 146.6 (d, *J* = 293.2 Hz), 139.5, 135.8 (d, *J* = 7.2 Hz), 132.7 (d, *J* = 25.7 Hz), 131.7, 131.4 (d, *J* = 2.5 Hz), 130.9, 130.4, 129.8, 126.9 (d, *J* = 9.8 Hz), 126.5, 95.9 (d, *J* = 205.0 Hz), 52.9, 18.8. **MS** (70 eV, EI): *m/z* (%): 319 (10) [M$^+$], 253 (5), 146 (7), 123 (100), 103 (12), 77 (9). **HRMS** (ESI): *m/z* [M + Na]$^+$ calculated for $C_{17}H_{15}F_2NNaO_3$ 342.0912, found: 342.0919. **IR** (neat, cm^{-1}): 2955, 1804, 1723, 1608, 1511, 1437, 1394, 1277, 1180, 1109, 986, 854, 748, 709, 660.

Methyl 4-(((2-bromo-5-chlorophenyl)fluoromethyl)(fluorocarbonyl)amino)thiophene-2-carboxylate (19)

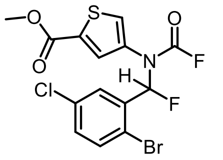

Prepared according to general procedure GP-2 from methyl 4-((2-bromo-5-chlorobenzylidene)amino)thiophene-2-carboxylate (**S4**, 179 mg, 0.500 mmol, 1.00 equiv). The title compound **19** was obtained as an orange syrup (191 mg, 0.449 mmol, 90 %). $\boldsymbol{R_f}$ = 0.18 (pentane/Et_2O 19:1 *v/v*).

^{1}H NMR (600 MHz, Acetonitrile-*d_3*) δ 7.61 – 7.57 (m, 1H), 7.56 (d, *J* = 1.6 Hz, 1H), 7.43 (s, 1H), 7.31 (d, *J* = 45.1 Hz, 1H), 7.31 – 7.24 (m, 2H), 3.80 (s, 3H). **^{19}F NMR** (564 MHz, Acetonitrile-*d_3*) δ -11.64 (s, 1F, rotamer A), -20.53 (s, 1F, rotamer B), -147.07 (d, *J* = 49.0 Hz, 1F, rotamer B), -151.21 (d, *J* = 48.7 Hz, 1F, rotamer A). **^{13}C NMR** (151 MHz, Acetonitrile-*d_3*) δ 162.2, 146.3 (d, *J* = 292.0 Hz), 135.9, 135.7, 135.5 (d, *J* = 2.1 Hz), 134.4 (d, *J* = 17.7 Hz), 133.8, 132.4, 132.2, 132.1, 129.1 (d, *J* = 11.2 Hz), 119.5, 96.4 (d, *J* = 208.0 Hz), 53.1. **MS** (70 eV, EI): *m/z* (%): 425 (2) [M^+], 423 (1) [M^+], 344 (22), 266 (18), 225 (28), 223 (100), 221 (82) 183 (9), 152 (14), 142 (9), 107 (18). **HRMS** (ESI): *m/z* [M + Na]$^+$ calculated for $C_{14}H_9BrClF_2NNaO_3S$ 447.9015, found: 447.9017. **IR** (neat, cm^{-1}): 3103, 2954, 1811, 1716, 1453, 1395, 1362, 1288, 1254, 1189, 1097, 1074, 1036, 996, 894, 862, 818, 791, 750, 722, 693.

(1-Fluoro-2-methylbutyl)(4-methoxyphenyl)carbamic fluoride (20)

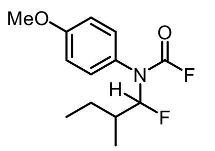

Prepared according to general procedure GP-2 from *N*-(4-methoxyphenyl)-2-methylbutan-1-imine (**S5**, 76.5 mg, 0.400 mmol, 1.00 equiv), without the addition of TBDOTf. The title compound **20** was obtained as a light-yellow oil (95.4 mg, 0.371 mmol, 93 %). *Note: The title compound was obtained as a mixture of two diastereomers (I/II = ~1.2:1), with each of the diastereomers were present as two rotamers and distinguishable in the NMR spectra.* $\boldsymbol{R_f}$ = 0.53 (pentane/EtOAc 19:1 *v/v*).

^{1}H NMR (600 MHz, Acetonitrile-*d_3*) δ 7.29 (dd, *J* = 11.5, 8.8 Hz, 4H, *diastereomer I + II*), 7.01 – 6.97 (m, 4H, *diastereomer I + II*), 5.89 (d, *J* = 49.1 Hz, 1H, *diastereomer II*), 5.87 (d, *J* = 49.6 Hz, 1H, *diastereomer I*), 3.82 (s, 3H, *diastereomer I*), 3.81 (s, 3H, *diastereomer II*), 1.66 – 1.44 (m, 4H, *diastereomer I + II*), 1.31 – 1.23 (m, 1H, *diastereomer II*), 1.19 – 1.08 (m, 1H, *diastereomer I*), 0.95 (dd, *J* = 6.7, 1.6 Hz, 3H, *diastereomer I*), 0.89 (d, *J* = 6.7 Hz, 3H, *diastereomer II*), 0.84 (t, *J* = 7.5 Hz, 3H, *diastereomer I*), 0.81 (t, *J* = 7.5 Hz, 3H, *diastereomer II*). **^{19}F NMR** (564 MHz, Acetonitrile-*d_3*) δ (rotamer A and A', 75 %) -9.82 (s, 1F, *diastereomer I*), -9.97 (s, 1F, *diastereomer II*), -145.38 (d, *J* = 49.3 Hz, 1F, *diastereomer II*), -146.60 (d, *J* = 49.2 Hz, 1F, *diastereomer I*); (rotamer B and B', 25 %) −20.92 (s, 1F, *diastereomer II*), −21.15 (s, 1F, *diastereomer I*), -140.15 (d, *J* = 51.2 Hz, 1F, *diastereomer II*), -141.19 (d, *J* = 50.3 Hz, 1F, *diastereomer I*). **^{13}C NMR** (151 MHz, Acetonitrile-*d_3*) δ 160.9 (*diastereomer I + II*), 147.7 (d, *J* = 287.7 Hz, *diastereomer I + II*), 131.2 (*diastereomer II*), 131.2 (*diastereomer I*), 130.7 (*diastereomer I + II*), 115.3 (*diastereomer II*), 115.3 (*diastereomer I*), 102.2 (d, *J* = 205.5 Hz, *diastereomer II*), 102.1 (d, *J* = 204.9 Hz, *diastereomer I*), 56.2, 36.9 (d, *J* = 22.1 Hz, *diastereomer I*), 36.6 (d, *J* = 23.0 Hz, *diastereomer II*), 24.9 (d, *J* = 3.6 Hz, *diastereomer I or II*), 24.4 (d, *J* = 6.1 Hz, *diastereomer I or II*), 14.1 (d, *J* = 4.7 Hz, *diastereomer I or II*), 13.8 (d, *J* = 7.1 Hz, *diastereomer I or II*), 10.7 (*diastereomer I or II*), 10.7 (*diastereomer I or II*). **MS** (70 eV, EI): *m/z* (%): 257 (44) [M^+], 237 (11), 200 (7), 169 (100), 154 (18), 149 (42), 134 (15). **HRMS** (EI): *m/z* [M^+] calculated for $C_{13}H_{17}F_2NO$ 257.1222, found: 257.1221. **IR** (neat, cm^{-1}): 2969, 2937, 2881, 2841, 1793, 1609, 1511, 1462, 1406, 1287, 1249, 1176, 1130, 1030, 980, 911, 837, 810, 748, 680.

(9-Ethyl-9*H*-carbazol-3-yl)(1-fluoro-2,2-dimethylpent-4-en-1-yl)carbamic fluoride (21)

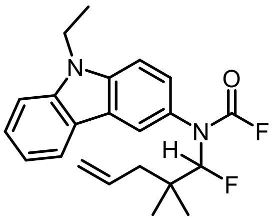

Prepared according to general procedure GP-2 from *N*-(9-ethyl-9*H*-carbazol-3-yl)-2,2-dimethylpent-4-en-1-imine (**S6**, 199 mg, 0.800 mmol, 1.00 equiv). The title compound **21** was obtained as a dark yellow syrup (278.5 mg, 0.752 mmol, 94 %). R_f = 0.28 (pentane/Et_2O 19:1 *v/v*).

^{1}H NMR (600 MHz, Acetonitrile-*d$_3$*) δ 8.19 (s, 1H), 8.14 (d, *J* = 7.8 Hz, 1H), 7.58 – 7.47 (m, 4H), 7.25 (ddd, *J* = 7.9, 6.8, 1.2 Hz, 1H), 6.18 (d, *J* = 44.9 Hz, 1H), 5.82 (ddt, *J* = 17.4, 10.2, 7.5 Hz, 1H), 5.11 – 5.02 (m, 2H), 4.42 (q, *J* = 7.2 Hz, 2H), 2.17 – 2.08 (m, 1H), 2.07 – 2.00 (m, 1H), 1.38 (t, *J* = 7.2 Hz, 3H), 0.79 (s, 3H), 0.71 (s, 3H). **^{19}F NMR** (564 MHz, Acetonitrile-*d$_3$*) δ -10.32 (s, 1F, rotamer A1), -10.77 (s, 1F, rotamer A2), -20.51 (s, 1F, rotamer B), -151.48 (brs, 1F, rotamer B), -155.12 (brs, 1F, rotamer A1), -155.64 (brs, 1F, rotamer A2). **^{13}C NMR** (151 MHz, Acetonitrile-*d$_3$*) δ 148.4 (d, *J* = 287.5 Hz), 141.6, 140.4, 134.6, 128.0, 127.6, 127.5, 123.6, 123.2, 122.1, 121.5, 120.3, 119.0, 110.3, 110.0, 102.6 (d, *J* = 209.6 Hz), 43.9 (d, *J* = 2.3 Hz), 39.8 (d, *J* = 23.7 Hz), 38.5, 23.0, 21.9, 14.0. **MS** (70 eV, EI): *m/z* (%): 371 (25) [M$^+$], 370 (100) [M$^+$], 350 (5), 305 (15), 304 (68), 289 (16), 263 (55), 256 (58), 241 (40), 236 (59), 234 (47), 222 (16), 221 (87), 209 (23), 205 (11), 198 (12), 196 (22), 180 (13), 179 (28), 166 (6), 152 (9). **HRMS** (ESI): *m/z* [M + Na]$^+$ calculated for $C_{22}H_{24}F_2N_2NaO$ 393.1749, found: 393.1746. **IR** (neat, cm^{-1}): 3071, 2976, 2936, 2876, 1793, 1484, 1305, 1230, 1150, 1120, 990, 923, 887, 810, 749, 714.

(Fluoro(phenyl)methyl)(furan-2-ylmethyl)carbamic fluoride (22)

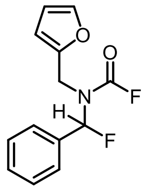

Prepared according to general procedure GP-2 from *N*-(furan-2-ylmethyl)-1-phenyl-methanimine (**S7**, 92.6 mg, 0.500 mmol, 1.00 equiv), without the addition of TBDOTf. The title compound **22** was obtained as a orange oil (103 mg, 0.409 mmol, 82 %). R_f = 0.42 (pentane/Et_2O 19:1 *v/v*).

^{1}H NMR (600 MHz, Acetonitrile-*d$_3$*) δ 7.48 – 7.40 (m, 5H), 7.37 (dd, *J* = 2.0, 0.8 Hz, 1H), 7.12 (dd, *J* = 65.2, 49.8 Hz, 1H), 6.30 (dd, *J* = 3.3, 1.9 Hz, 1H), 6.11 – 5.99 (m, 1H), 4.52 – 4.40 (m, 1H), 4.20 (m, 1H). **^{19}F NMR** (564 MHz, Acetonitrile-*d$_3$*) δ -15.21 (s, 1F, rotamer A), -19.41 (s, 1F, rotamer B), -149.48 (d, *J* = 49.6 Hz, 1F, rotamer B), -155.19 (d, *J* = 48.6 Hz, 1F, rotamer A). **^{13}C NMR** (151 MHz, Acetonitrile-*d$_3$*) δ 149.8 (rotamer A or B), 149.6 (rotamer A or B), 147.8 (d, *J* = 197.3 Hz, rotamer A), 145.9 (d, *J* = 196.6 Hz, rotamer B), 142.6 (rotamer A + B), 134.2 (d, *J* = 27.3 Hz, rotamer A + B), 129.5 (rotamer A + B), 128.8 (rotamer A), 128.7 (rotamer B), 125.7 (rotamer B), 125.7 (rotamer A), 110.5 (rotamer A + B), 108.7 (rotamer B), 108.3 (rotamer A), 96.5 (d, *J* = 203.5 Hz, rotamer A), 96.0 (d, *J* = 207.7 Hz, rotamer B), 41.3 (rotamer B), 40.1 (rotamer A). **MS** (70 eV, EI): *m/z* (%): 251 (17) [M$^+$], 231 (6), 185 (14), 142 (100), 122 (10), 109 (46), 99 (10), 81 (49), 77 (6), 53 (9). **HRMS** (EI): *m/z* [M]$^+$ calculated for $C_{13}H_{11}F_2NO_2$ 251.0752, found: 251.0751. **IR** (neat, cm^{-1}): 3066, 1798, 1600, 1449, 1410, 1332, 1282, 1207, 1163, 1077, 1045, 989, 948, 828, 741, 698.

Allyl(fluoro(phenyl)methyl)carbamic fluoride (23)

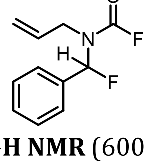

Prepared according to general procedure GP-2 from *N*-allyl-1-phenylmethanimine (**S8**, 58.1 mg, 0.400 mmol, 1.00 equiv), without the addition of TBDOTf. The title compound **23** was obtained as a light-yellow oil (52.7 mg, 0.250 mmol, 62 %). R_f = 0.56 (pentane/EtOAc 19:1 *v/v*).

^{1}H NMR (600 MHz, Acetonitrile-*d$_3$*) δ 7.52 – 7.41 (m, 5H), 7.12 (dd, *J* = 66.8, 49.6 Hz, 1H), 5.65 (ddt, *J* = 16.6, 11.1, 5.8 Hz, 1H), 5.06 (q, *J* = 1.5 Hz, 1H), 5.03 (dq, *J* = 9.6, 1.5 Hz, 1H), 3.92 – 3.77 (m, 1H), 3.65 (dd, *J* = 16.6, 5.2 Hz, 1H). **^{19}F NMR** (564 MHz, Acetonitrile-*d$_3$*) δ -16.00 (s, 1F, rotamer A), −20.34 (s, 1F, rotamer B), -154.31 (d, *J* = 48.6 Hz, 1F, rotamer A) -148.43 (d, *J* = 49.6 Hz, 1F, rotamer B). **^{13}C NMR** (151 MHz, Acetonitrile-*d$_3$*) δ 148.8 (d, *J* = 284.6 Hz, rotamer A+B), 135.4 (d, *J* = 27.8 Hz, rotamer A+B), 134.0 (rotamer

A+B), 133.2 (rotamer A+B), 130.5 (rotamer A+B), 129.7 (d, *J* = 1.3 Hz, rotamer A+B), 126.7 (d, *J* = 7.6 Hz, rotamer A+B), 97.5 (d, *J* = 201.5 Hz, rotamer A), 97.0 (d, *J* = 207.3 Hz, rotamer B), 47.9 (rotamer B), 46.9 (rotamer A). **MS** (70 eV, EI): *m/z* (%): 211 (2) [M+], 170 (22), 150 (15), 109 (100), 102 (7), 83 (8). **HRMS** (ESI): *m/z* [M + Na]+ calculated for $C_{11}H_{11}F_2NNaO$ 234.0701, found: 234.0704. **IR** (neat, cm^{-1}): 3072, 1797, 1452, 1409, 1328, 1279, 1252, 1217, 1182, 1131, 1035, 986, 930, 829, 753, 699.

((4-Chlorophenyl)fluoromethyl)(cyclohexyl)carbamic fluoride (24)

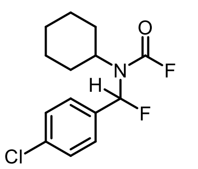

Prepared according to general procedure GP-2 from 1-(4-chlorophenyl)-*N*-cyclohexylmethanimine (**S9**, 177 mg, 0.800 mmol, 1.00 equiv), without the addition of TBDOTf. The title compound **24** was obtained as a yellow solid (217 mg, 0.752 mmol, 94 %). $\boldsymbol{R_f}$ = 0.36 (pentane/Et_2O 19:1 *v/v*). **M.p.**: 62 – 64 °C.

^{1}H NMR (600 MHz, Acetonitrile-*d3*) *δ* 7.49 – 7.45 (m, 2H), 7.45 – 7.40 (m, 2H), 6.91 (d, *J* = 32.4 Hz, 1H), 3.32 (brs, 1H), 1.90 – 1.62 (m, 5H), 1.59 – 1.48 (m, 2H), 1.28 (dddt, *J* = 16.7, 13.0, 9.8, 3.7 Hz, 1H), 1.16 – 0.99 (m, 2H). **^{19}F NMR** (564 MHz, Acetonitrile-*d3*) *δ* -9.93 (s, 1F, rotamer A), -10.85 (s, 1F, rotamer B), -150.44 (s, 1F, rotamer A), -156.84 (s, 1F, rotamer B). **^{13}C NMR** (151 MHz, Acetonitrile-*d3*) *δ* 145.8 (d, *J* = 117.9 Hz), 135.6 (d, *J* = 21.3 Hz), 135.4, 129.7, 128.0 (d, *J* = 7.2 Hz), 96.1 (d, *J* = 198.1 Hz), 57.9, 30.9, 26.5, 26.5, 25.7. **MS** (70 eV, EI): *m/z* (%): 289 (5) [M+], 287 (13) [M+], 221 (5), 206 (10), 204 (27), 192 (7), 145 (36), 143 (100), 125 (7), 82 (9), 55 (5). **HRMS** (EI): *m/z* [M]+ calculated for $C_{14}H_{16}ClF_2NO$ 287.0883, found: 287.0886. **IR** (neat, cm^{-1}): 2940, 2860, 1796, 1489, 1435, 1401, 1323, 1288, 1241, 1217, 1176, 1145, 1082, 1053, 982, 884, 798, 754, 730, 693, 658.

(2-Bromophenethyl)(1-fluoro-2,2-dimethylpropyl)carbamic fluoride (25)

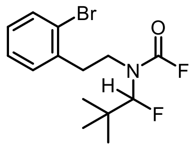

Prepared according to general procedure GP-2 from *N*-(2-bromophenethyl)-2,2-dimethylpropan-1-imine (**S10**, 107 mg, 0.400 mmol, 1.00 equiv), without the addition of TBDOTf. The title compound **25** was obtained as a orange oil (131 mg, 0.391 mmol, 98 %). $\boldsymbol{R_f}$ = 0.65 (pentane/Et_2O 19:1 *v/v*).

^{1}H NMR (600 MHz, Acetonitrile-*d3*) *δ* 7.60 – 7.55 (m, 1H), 7.35 – 7.28 (m, 2H), 7.16 (ddd, *J* = 8.0, 6.3, 2.8 Hz, 1H), 5.75 (d, *J* = 42.1 Hz, 1H, *rotamer A*), 5.60 (d, *J* = 42.1 Hz, 1H, *rotamer B*), 3.64 – 3.56 (m, 1H), 3.52 (s, 1H), 3.09 (s, 2H), 1.02 (s, 9H). **^{19}F NMR** (564 MHz, Acetonitrile-*d3*) *δ* -15.97(s, 1F, rotamer A), -15.55 (s, 1F, rotamer B), -168.74 (d, *J* = 42.2 Hz, 1F, rotamer A), -166.47 (s, 1F, rotamer B). **^{13}C NMR** (151 MHz, Acetonitrile-*d3*) *δ* 148.2 (d, *J* = 245.7 Hz, rotamer A), 146.2 (d, *J* = 239.9 Hz, rotamer B), 139.0 (rotamer B), 138.7 (rotamer A), 133.8 (rotamer A + B), 132.3 (rotamer A + B), 129.7 (rotamer A + B), 129.0 (rotamer A + B), 124.9 (rotamer A + B), 103.4 (d, *J* = 207.6 Hz, rotamer A), 103.0 (d, *J* = 208.8 Hz, rotamer B), 45.8 (rotamer B), 45.0 (rotamer A), 38.0 (d, *J* = 23.9 Hz, rotamer A + B), 37.5 (rotamer A), 35.3 (rotamer B), 25.3 (d, *J* = 3.2 Hz, rotamer A + B). **MS** (70 eV, EI): *m/z* (%): 335 (12) [M+], 333 (12) [M+], 258 (17), 266 (17), 254 (33), 185 (46), 184 (100), 183 (46), 182 (98), 171 (18), 169 (18), 164 (28), 146 (7), 104 (27), 89 (64), 69 (34), 57 (25). **HRMS** (EI): *m/z* [M]+ calculated for $C_{14}H_{18}BrF_2NO$ 333.0534, found: 233.0533. **IR** (neat, cm^{-1}): 2968, 2879, 1791, 1470, 1420, 1357, 1312, 1162, 1022, 896, 751, 665.

7. Derivatizations of *N*-CH_2F and *N*-CHRF carbamoyl fluorides

7.1. General procedures

7.1.1. General procedure (GP-3) for *N*-CH_2F carbamate synthesis

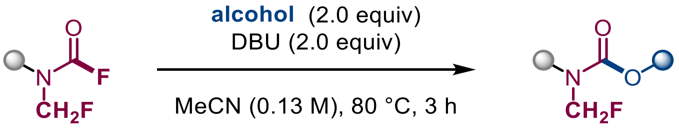

A 4 mL vial was charged with the *N*-CH_2F fluoride (1.0 equiv) and MeCN (0.13 M). Subsequently, the corresponding alcohol (2.0 equiv) and DBU (2.0 equiv) were added to the solution. The reaction mixture was stirred for 3 h at 80 °C. After the indicated time, the reaction mixture was cooled down to rt, concentrated under reduced pressure. The crude residue was then purified by flash column chromatography on neutralized silica gel with 2-3% Et_3N.

7.1.2. General procedure (GP-4) for *N*-CHRF carbamate synthesis

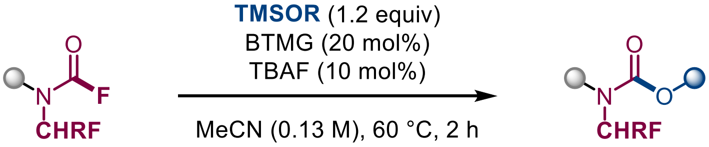

A 4 mL vial was charged with the *N*-CHRF carbamoyl fluoride (0.10 mmol, 1.0 equiv) and MeCN (0.75 mL, 0.13 M). Subsequently, the corresponding TMS-protected alcohol (0.12 mmol, 1.2 equiv), BTMG (4.0 μL, 0.020 mmol, 0.20 equiv) and TBAF (1M solution in THF, 10 μL, 0.010 mmol, 0.10 equiv) were added to the solution. The reaction mixture was stirred for 2 h at 60 °C. After completion, the solvent was evaporated under reduced pressure and the crude residue was purified by flash column chromatography on neutralized silica gel with 2-3% Et_3N.

7.1.3. General procedure (GP-5) for *N*-CH_2F and *N*-CHRF thiocarbamate synthesis[1]

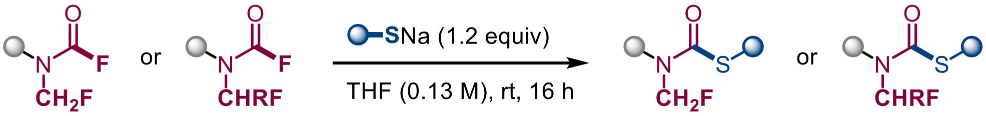

A 4 mL vial was charged with the *N*-CH_2F or *N*-CHRF carbamoyl fluoride (1.0 equiv) and THF (0.13 M). Subsequently, the corresponding sodium thiolate (1.2 equiv) was added. The reaction mixture was stirred for 16 h at room temperature. After completion, hexane (1 mL) was added, and the reaction mixture was filtered through a pad of celite before evaporation under reduced pressure. The crude residue was then purified by flash column chromatography on neutralized silica gel using 2-3% Et_3N.

7.1.4. General procedure (GP-6) for *N*-CH_2F formamide synthesis[3]

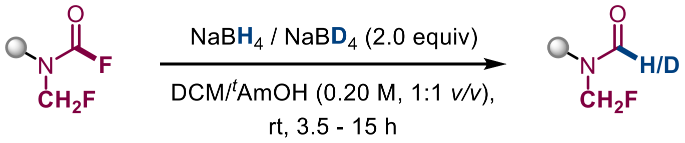

A solution of *N*-CH_2F carbamoyl fluoride (1.0 equiv) in DCM was added to a mixture of $NaBH_4$ or $NaBD_4$ (2.0 equiv) in ^{t}AmOH and the reaction mixture was stirred for the indicated time at room temperature. The reaction mixture was then quenched by slow addition of water and stirred for additional 5 min. The organic phase was separated, and the aqueous phase was extracted two times with DCM. The combined organic phases were dried over $MgSO_4$, concentrated *in vacuo* and the crude product purified by flash column chromatography on neutralized silica gel using 2-3% Et_3N.

7.1.5. General procedure (GP-7) for *N*-CH_2F and *N*-CHRF formamide synthesis

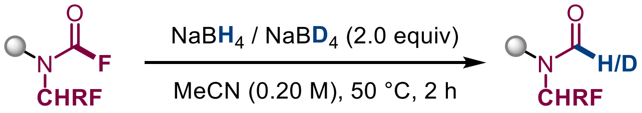

A solution of *N*-CHRF carbamoyl fluoride (1.0 equiv) in MeCN (0.20 M) was added to $NaBH_4$ or $NaBD_4$ (2.0 equiv), and the reaction mixture was stirred for 2 h at 50 °C. The reaction mixture was then quenched by slow addition of water and stirred for 5 min. The organic phase was separated, and the aqueous phase was extracted with DCM (3 x 20 mL). The combined organic phases were dried over $MgSO_4$, concentrated *in vacuo* and the crude product purified by flash column chromatography on neutralized silica gel with 2-3% Et_3N.

7.1.6. General procedure (GP-8) for *N*-CH_2F carbamoyl azide and cyanide synthesis

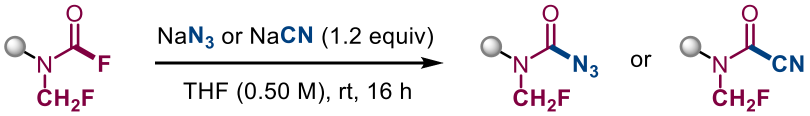

To the solution of *N*-CH_2F carbamoyl fluoride (1.0 equiv) in THF (0.50 M) was added NaCN or NaN_3 (1.2 equiv) and the reaction mixture was stirred for 16 h at room temperature. The reaction mixture was then filtered over celite and concentrated *in vacuo*. The crude product was purified by flash column chromatography on silica gel.

Note: All *N*-CH_2F and *N*-CHRF carbonyl derivatives were stored in a glass vial, under argon, at −30 °C in the freezer to prevent decomposition.

7.1.7. General procedure (GP-9) for *N*-CHRF carbamoyl azide and cyanide synthesis

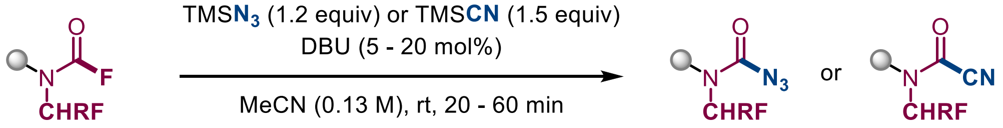

A 4 mL vial was charged with the *N*-CHRF carbamoyl fluoride (0.10 mmol, 1.0 equiv) and MeCN (0.75 mL, 0.13 M). After addition of $TMSN_3$ (16 µL, 0.12 mmol, 1.2 equiv) and DBU (3.0 µL, 0.020 mmol, 0.20 equiv), or TMSCN (19 µL, 0.15 mmol, 1.5 equiv) and DBU (0.8 µL, 0.005 mmol, 0.05 equiv), respectively, the reaction mixture was stirred at room temperature. After completion, the solvent was evaporated under reduced pressure and the crude residue was purified by flash column chromatography on neutralized silica gel with 2-3% Et_3N.

7.1.8. General procedure (GP-10) for *N*-CH_2F and *N*-CHRF alkynamide synthesis[4]

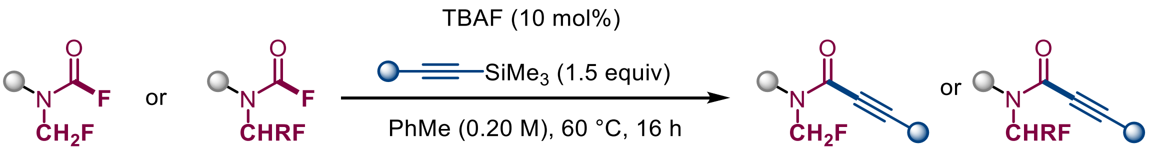

A 4 mL vial was charged with the corresponding *N*-CH_2F or *N*-CHRF carbamoyl fluoride (1.0 equiv) and toluene (0.20 M). Subsequently, the respective trimetylsilyl-alkyne (1.5 equiv) was added, followed by TBAF (10 mol%, as 1.0 M solution in THF). The reaction mixture was stirred at 60 °C for 16 h. The reaction mixture was then cooled to room temperature and filtered through a short plug of Celite® and concentrated *in vacuo*. The crude product was purified by flash column chromatography on neutralized silica gel using 2-3% Et_3N.

7.2. Characterization data of *N*-CH_2F and *N*-CHRF carbamates

***tert*-Butyl 4-((fluoromethyl)(((4-oxo-2-phenyl-4H-chromen-7-yl)oxy)carbonyl)amino)piperidine-1-carboxylate (26)**

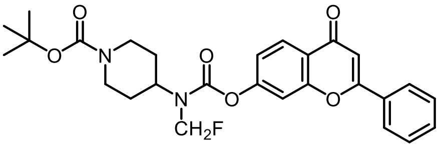

Prepared according to general procedure GP-3 on a 0.10 mmol scale, and isolated as yellow oil (35 mg, 0.070 mmol, 70 %). ***Rf*** = 0.27 (30 % EtOAc/pentane + 2 % Et_3N). **^{1}H NMR** (600 MHz, Acetonitrile-*d3*) *δ* 8.11 (d, *J* = 8.7 Hz, 1H), 8.03 – 7.97 (m, 2H), 7.60 – 7.53 (m, 3H), 7.52 (brs, 1H), 7.25 (d, *J* = 8.1 Hz, 1H), 6.80 (s, 1H), 5.63 (d, *J* = 53.8 Hz, 2H), 4.27 – 4.05 (m, 3H), 2.80 (brs, 2H), 1.89 – 1.80 (m, 1H), 1.73 – 1.62 (m, 2H), 1.44 (s, 9H). **^{19}F NMR** (564 MHz, Acetonitrile-*d3*) *δ* -164.58 (t, *J* = 54.3 Hz, 1F, rotamer A), -167.40 (t, *J* = 54.6 Hz, 1F, rotamer B). **^{13}C NMR** (151 MHz, Acetonitrile-*d3*) *δ* 177.9, 164.5, 157.6, 155.9, 155.3, 132.7, 132.5, 130.0, 127.3, 122.5, 120.5, 112.3, 108.1, 85.3 (d, *J* = 194.5 Hz), 80.0, 55.8, 44.4 (rotamer A), 43.7 (rotamer B), 31.8 (rotamer A), 31.1 (rotamer B), 28.5. **HRMS** (ESI): *m/z* $[M + Na]^+$ calculated for $C_{27}H_{29}O_6N_2FNa$: 519.1902, found 519.1918. **IR** (neat, cm^{-1}): 1735, 1688, 1645, 1418, 1362, 1235, 1148, 1026, 945, 768, 691.

Methyl (*S*)-3-(4-(([1,1'-biphenyl]-4-yl(fluoromethyl)carbamoyl)oxy)phenyl)-2-((*tert*-butoxy-carbonyl)amino)propanoate (27)

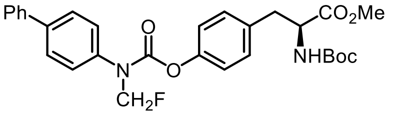

Prepared according to general procedure GP-3 on a 0.10 mmol scale, and isolated as a white solid (48 mg, 0.092 mmol, 92 %). ***Rf*** = 0.16 (20 % EtOAc/pentane + 3 % Et_3N). **M.p.**: 85 – 87 °C.

^{1}H NMR (600 MHz, Acetonitrile-*d3*) *δ* 7.72 (d, *J* = 8.5 Hz, 2H), 7.67 (d, *J* = 7.7 Hz, 2H), 7.54 (d, *J* = 8.4 Hz, 2H), 7.47 (t, *J* = 7.6 Hz, 2H), 7.39 (d, *J* = 7.4 Hz, 1H), 7.23 (d, *J* = 8.4 Hz, 2H), 7.11 (d, *J* = 8.3 Hz, 2H), 5.80 (d, *J* = 54.5 Hz, 2H), 5.56 (brs, 1H). 7.37 – 7.28 (m, 1H), 3.65 (s, 3H), 3.12 – 2.90 (m, 2H), 1.36 (s, 9H). **^{19}F NMR** (564 MHz, Acetonitrile-*d3*) *δ* -165.1 (brs, 1F, rotamer B), -167.8 (brs, 1F, rotamer A). **^{13}C NMR** (151 MHz, Acetonitrile-*d3*) *δ* 173.3, 155.4 (d, *J* = 263.3 Hz). 150.7, 141.4, 140.9, 140.8, 136.0, 131.3, 129.9, 128.7, 128.4, 127.9, 122.5, 90.4 (d, *J* = 197.5 Hz), 80.0, 55.8, 52.7, 37.5, 28.4. **HRMS** (ESI): *m/z* $[M + Na]^+$ calculated for $C_{29}H_{31}F_1N_2O_6Na$: 545.2058, found 545.2051. **IR** (neat, cm^{-1}): 2956, 2227, 1796, 1678, 1426, 1366, 1257, 1218, 1166, 1140, 1066, 1021, 794, 672.

((3a*R*,5*R*,5a*S*,8a*S*,8b*R*)-2,2,7,7-Tetramethyltetrahydro-5*H*-bis([1,3]dioxolo)[4,5-*b*:4',5'-*d*]pyran-5-yl)methyl (fluoro(phenyl)methyl)(phenyl)carbamate (28)

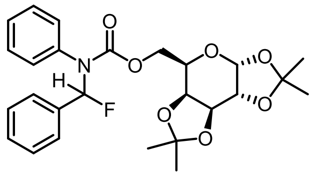

Prepared according to general procedure GP-4 from (fluoro(phenyl)methyl)(phenyl)carbamic fluoride (**16**, 24.7 mg, 0.100 mmol, 1.00 equiv) and 1,2:3,4-bis-*O*-(1-methylethylidene)-6-*O*-(trimethylsilyl)-α-D-galactopyranose (**S11**, 39.9 mg, 0.120 mmol, 1.20 equiv). The title compound **28** was obtained as a colorless syrup (34.1 mg, 0.0699 mmol, 70 %). *Note: 2 % Et_3N was added to the eluent mixture for the column chromatography; the title compound was obtained as a mixture of two diastereomers (d. r. = 1:1).* ***Rf*** = 0.62 (pentane/Et_2O/Et_3N 75:20:5 *v/v*).

^{1}H NMR (600 MHz, Acetonitrile-*d3*) *δ* 7.57 (d, *J* = 50.5 Hz, 1H, *diastereomer I or II*), 7.57 (d, *J* = 50.7 Hz, 1H, *diastereomer I or II*), 7.26 – 7.16 (m, 16H, *diastereomer I + II*), 7.08 – 7.04 (m, 4H, *diastereomer I + II*), 5.46 (d, *J* = 4.9 Hz, 1H, *diastereomer I or II*), 5.44 (d, *J* = 4.9 Hz, 1H, *diastereomer I or II*), 4.61 (t, *J* = 2.8 Hz, 1H, *diastereomer I or II*), 4.59 (t, *J* = 2.8 Hz, 1H, *diastereomer I or II*), 4.35 – 4.33 (m, 2H, *diastereomer I + II*), 4.33 – 4.28 (m, 2H, *diastereomer I + II*), 4.23 – 4.12 (m, 4H, *diastereomer I + II*), 4.01 – 3.97 (m, 1H, *diastereomer I or II*), 3.97 – 3.94 (m, 1H, *diastereomer I or II*), 1.43 (s, 3H, *diastereomer I or II*), 1.42 (s, 3H, *diastereomer I or II*), 1.39 (s, 3H, *diastereomer I or II*), 1.38 (s, 3H, *diastereomer I or II*), 1.31 (s, 3H,

diastereomer I or II), 1.31 (s, 3H, *diastereomer I or II*), 1.30 (s, 3H, *diastereomer I or II*), 1.30 (s, 3H, *diastereomer I or II*). **^{19}F NMR** (564 MHz, Acetonitrile-*d*$_3$) δ -145.02 (s, 1F, *diastereomer I*) -145.10 (s, 1F, *diastereomer II*). **^{13}C NMR** (151 MHz, Acetonitrile-*d$_3$*) δ 156.3 (*diastereomer I or II*), 156.3 (*diastereomer I or II*), 137.8 (m, *diastereomer I + II*), 136.6 (d, *J* = 28.8 Hz, *diastereomer I + II*), 130.6 (d, *J* = 7.5 Hz, *diastereomer I or II*), 130.6 (d, *J* = 7.4 Hz, *diastereomer I or II*), 129.5 (*diastereomer I or II*), 129.5 (*diastereomer I or II*), 129.4 (*diastereomer I + II*), 129.0 (*diastereomer I + II*), 128.6 (*diastereomer I or II*), 128.6 (*diastereomer II or I*), 126.9 (d, *J* = 5.7 Hz, *diastereomer I or II*), 126.8 (d, *J* = 5.7 Hz, *diastereomer I or II*), 110.1 (*diastereomer I or II*), 110.1 (*diastereomer I or II*), 109.5 (*diastereomer I + II*), 97.1 (*diastereomer I or II*), 97.1 (*diastereomer I or II*), 96.8 (d, *J* = 202.0 Hz, *diastereomer I or II*), 96.8 (d, *J* = 201.8 Hz, *diastereomer I or II*), 71.7 (*diastereomer I or II*), 71.7 (*diastereomer I or II*), 71.5 (*diastereomer I or II*), 71.5 (*diastereomer I or II*), 71.4 (*diastereomer I or II*), 71.3 (*diastereomer I or II*), 67.0 (*diastereomer I + II*), 67.0 (*diastereomer I + II*), 66.3 (*diastereomer I or II*), 66.2 (*diastereomer I or II*), 26.4 (*diastereomer I/II*), 26.3 (*diastereomer I/ II*), 26.2 (*diastereomer I /II*), 26.2 (*diastereomer I/II*), 25.2 (*diastereomer I /II*), 24.6 (*diastereomer I /II*). **HRMS** (ESI): *m/z* [M + Na]$^+$ calculated for $C_{26}H_{30}FNNaO_7$ 510.1899, found: 510.1882. **IR** (neat, cm^{-1}): 2979, 2934, 2804, 1717, 1597, 1496, 1452, 1381, 1294, 1256, 1210, 1168, 1114, 1066, 1003, 964, 897, 859, 766, 713.

7.3. Characterization data of *N*-CH$_2$F and *N*-CHRF thiocarbamates

S-Ethyl (fluoromethyl)(3,4,5-trimethoxyphenyl)carbamothioate (29)

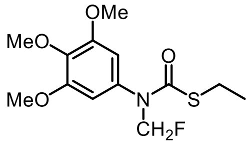

Prepared according to general procedure GP-5 on a 0.10 mmol scale, and isolated as a yellow oil (23 mg, 0.077 mmol, 77 %). ***R$_f$*** = 0.67 (20 % EtOAc/pentane + 2 % Et_3N).

^{1}H NMR (600 MHz, Acetonitrile-*d*$_3$) δ 6.67 (s, 2H, rotamer A), 6.65 (s, 2H, rotamer B), 5.61 (d, *J* = 54.6 Hz, 2H), 3.79 (s, 6H), 3.75 (s, 3H), 2.84 (q, *J* = 7.4 Hz, 2H, rotamer A), 2.79 (q, *J* = 7.4 Hz, 2H, rotamber B), 1.22 (t, *J* = 7.4 Hz, 3H, rotamer A), 1.19 (t, *J* = 7.3 Hz, 3H, rotamer B). **^{19}F NMR** (564 MHz, Acetonitrile-*d*$_3$) δ -166.61 (t, *J* = 52.1 Hz, 1F). **^{13}C NMR** (151 MHz, Acetonitrile-*d*$_3$) δ 171.9 (d, *J* = 3.1 Hz), 154.5, 139.6, 136.0 (d, *J* = 3.0 Hz), 107.9, 87.9 (d, *J* = 197.6 Hz), 60.9, 56.9, 26.0, 15.2. **HRMS** (ESI): *m/z* [M + Na]$^+$ calculated for $C_{13}H_{18}O_4NFSNa$: 326.0833, found 326.0834. **IR** (neat, cm^{-1}): 1669, 1591, 1500, 1455, 1416, 1314, 1229, 1124, 1053, 1004, 947, 687.

S-(Pyridin-2-yl) (fluoromethyl)(2-isopropylphenyl)carbamothioate (30)

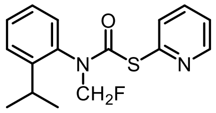

Prepared according to general procedure GP-5 on a 0.10 mmol scale, and isolated as a colorless oil (28 mg, 0.091 mmol, 91 %). ***R$_f$*** = 0.22 (3 % EtOAc/pentane + 3 % Et_3N).

^{1}H NMR (600 MHz, Acetonitrile-*d$_3$*) δ 8.54 (d, *J* = 2.9 Hz, 1H), 7.79 (td, *J* = 7.7, 1.9 Hz, 1H), 7.64 (d, *J* = 7.9 Hz, 1H), 7.54 – 7.52 (m, 2H), 7.38 – 7.33 (m, 3H), 5.77 (dd, *J* = 53.8, 8.1 Hz, 2H, rotamer A), 5.46 (dd, *J* = 54.2, 8.2 Hz, 2H, rotamer B), 3.20 – 3.16 (m, 1H), 1.37 (d, *J* = 6.9 Hz, 6H, rotamer B), 1.21 (d, *J* = 6.9 Hz, 6H, rotamer A). **^{19}F NMR** (564 MHz, Acetonitrile-*d$_3$*) δ -169.64 (t, *J* = 56.2 Hz, 1F). **^{13}C NMR** (151 MHz, Acetonitrile-*d$_3$*) δ 170.0, 152.8, 151.1, 149.2, 138.3, 136.9, 131.8, 131.4, 128.4, 128.1, 124.8, 88.2 (d, *J* = 198.7 Hz), 28.8, 24.6, 23.6. **HRMS** (ESI): *m/z* [M + H]$^+$ calculated for $C_{16}H_{17}FN_2OS$: 305.1118, found 305.1121. **IR** (neat, cm^{-1}): 2967, 2323, 2100, 1933, 1682, 1570, 1450, 1229, 1070, 956, 759, 676.

S-(2-Chlorophenyl) (fluoro(phenyl)methyl)(phenyl)carbamothioate (31)

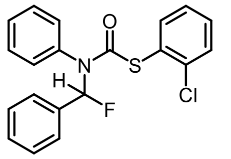

Prepared according to general procedure GP-5 from (fluoro(phenyl)methyl)-(phenyl)carbamic fluoride (**16**, 24.7 mg, 0.100 mmol, 1.00 equiv) and sodium 2-chlorobenzenethiolate (20.0 mg, 0.120 mmol, 1.20 equiv). The title compound **31** was obtained as a white solid (25.4 mg, 0.0683 mmol, 68 %). ***R**$_f$* = 0.35 (pentane/Et_2O/Et_3N 90:5:5 *v/v*). **M.p.**: 127 – 129 °C.

^{1}H NMR (600 MHz, Acetonitrile-*d_3*) δ 7.63 (d, *J* = 50.5 Hz, 1H), 7.62 – 7.57 (m, 2H), 7.46 (td, *J*= 7.9, 1.7 Hz, 1H), 7.41 – 7.18 (m, 11H). **^{19}F NMR** (564 MHz, Acetonitrile-*d_3*) δ -146.08 (d, *J* = 49.8 Hz, 1F). **^{13}C NMR** (151 MHz, Acetonitrile-*d_3*) δ 169.3 (d, *J* = 2.4 Hz), 140.0, 139.0, 136.1 (d, *J* = 2.4 Hz), 135.9 (d, *J* = 28.1 Hz), 132.7, 132.6, 131.0, 130.8, 130.0, 129.8, 129.1, 129.1 (d, *J* = 1.1 Hz), 128.6, 126.9 (d, *J* = 7.2 Hz), 94.5 (d, *J* = 203.9 Hz). **HRMS** (APCI): *m/z* $[M + H]^+$ calculated for $C_{20}H_{16}ClFNOS$ 372.0620, found: 372.0629. **IR** (neat, cm^{-1}): 3059, 3005, 2923, 1677, 1595, 1492, 1451, 1382, 1353, 1219, 1120, 1059, 1029, 960, 742, 707.

7.4. Characterization data of *N*-CH_2F and *N*-CHRF formamides

***N*-(1-(2,6-Dimethylphenoxy)propan-2-yl)-*N*-(fluoromethyl)formamide (32)**

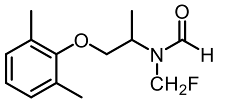

Prepared according to general procedure GP-6 (stirred for 15 h at rt) on a 0.20 mmol scale, and isolated as a yellow oil (15 mg, 0.063 mmol, 31 %). ***R**$_f$* = 0.47 (20 % EtOAc/pentane + 2 % Et_3N).

^{1}H NMR (400 MHz, Acetonitrile-*d_3*) δ 8.38 (s, 1H, rotamer A), 8.34 (s, 1H, rotamer B), 7.07 – 6.98 (m, 2H, A+B), 6.95 – 6.87 (m, 1H, rotamer A+B), 5.77 – 5.42 (m, 2H, A+B), 4.65 (h, *J* = 6.5 Hz, 1H, rotamer A), 4.14 (h, *J* = 6.9 Hz, 1H, rotamer B), 3.90 – 3.71 (m, 2H, rotamer A+B), 2.22 (s, 6H, rotamer A), 2.20 (s, 6H, rotamer B), 1.40 (d, *J* = 7.1 Hz, 3H, rotamer A+B). **^{19}F NMR** (376 MHz, Acetonitrile-*d_3*) δ -159.24 (t, *J* = 55.6 Hz, 1F rotamer A), -170.51 (t, *J* = 54.1 Hz, 1F, rotamer B). **^{13}C NMR** (151 MHz, Acetonitrile-*d_3*) δ 165.7 (d, *J* = 3.3 Hz, rotamer B), 165.6 (d, *J* = 2.7 Hz, rotamer A), 156.2 (rotamer A), 156.0 (rotamer B), 131.7 (rotamer A), 131.7 (rotamer B), 129.8 (rotamer A+B), 125.1 (rotamer B), 125.0 (rotamer A), 88.0 (d, *J* = 193.4 Hz, rotamer A), 81.2 (d, *J* = 190.7 Hz, rotamer B), 74.5 (rotamer B), 73.8 (d, *J* = 2.3 Hz, rotamer A), 54.3 (d, *J* = 2.7 Hz, rotamer B), 49.2 (d, *J* = 3.2 Hz, rotamer A), 16.3 (rotamer A+B), 16.2 (rotamer B), 15.8 (rotamer A). **HRMS** (EI): found $[C_5H_9FNO]^+$; $[C_8H_9O]^+$. **IR** (neat, cm^{-1}): 1692, 1471, 1393, 1198, 1021, 948, 770, 714.
Note: All our efforts (EI, ESI, APCI) to measure molecular ion failed.

***N*-([1,1'-Biphenyl]-4-yl)-*N*-(fluoromethyl-*d*2)formamide-*d* (33)**

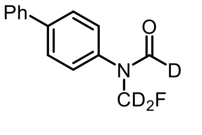

Prepared according to the general procedure GP-6 (stirred for 3.5 h at rt) on a 0.10 mmol scale, and isolated as a white solid (7.4 mg, 0.032 mmol, 32 %). ***R**$_f$* = 0.21 (10 % EtOAc/pentane + 2 % Et_3N). **M.p.**: 84 – 88 °C.

^{1}H NMR (600 MHz, Acetonitrile-*d_3*) δ 8.58 (s, 1H, NC(O)*H*), 7.75 (d, *J* = 8.5 Hz, 2H, rotamer A), 7.72 (d, *J* = 8.5 Hz, 2H, rotamer B), 7.67 (d, *J* = 9.3 Hz, 2H), 7.48 (t, *J* = 7.5 Hz, 2H), 7.45 – 7.38 (m, 3H). **^{19}F NMR** (564 MHz, Acetonitrile-*d_3*) δ -162.32 (p, *J* = 8.5 Hz, 1F, rotamer B), -172.19 (p, *J* = 8.3 Hz, 1F, rotamer A). **^{13}C NMR** (151 MHz, Acetonitrile-*d_3*) δ 164.7 (rotamer B), 163.9 (rotamer A), 141.0, 140.8, 140.8, 140.6, 140.3, 138.5, 129.9, 129.9, 129.1, 128.7, 128.7, 128.6, 127.9, 127.8, 127.0, 124.9. **IR** (neat, cm^{-1}): 3358, 3035, 2195, 1723, 1665, 1606, 1483, 1319, 1250, 1137, 1064, 912, 875, 832, 763, 695. **HRMS** (ESI): *m/z* $[M]^+$ calculated for $C_{14}H_9FNOD_3$: 232.1085, found 232.1084. **IR** (neat, cm^{-1}): 3358, 3035, 2195, 1723, 1665, 1606, 1483, 1319, 1250, 1137, 1064, 912, 875, 832, 763, 695.
Note: Reported as the mixture of rotamers CDO is not triplet and CD_2F is not visible (probably intensity lowers because of the coupling).

_N_-([1,1'-Biphenyl]-4-yl)-_N_-(fluoromethyl)formamide (34)

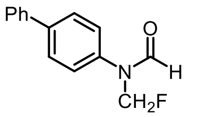

Prepared according to general procedure GP-6 (stirred for 3.5 h at rt) on a 0.20 mmol scale, and isolated as a pale yellow solid (14 mg, 0.060 mmol, 30 %). R_f = 0.23 (10 % EtOAc/pentane + 2 % Et_3N).

^{1}H NMR (600 MHz, Methylene chloride-d_2) δ 8.59 (s, 1H, rotamer B), 8.57 (s, 1H, rotamer A), 7.73 – 7.65 (m, 2H), 7.64 – 7.59 (m, 2H), 7.51 – 7.42 (m, 3H), 7.41 – 7.34 (m, 2H), 5.77 (d, J = 53.1 Hz, 2H, rotamer A), 5.68 (d, J = 53.4 Hz, 2H, rotamer B). **^{19}F NMR** (564 MHz, Methylene Chloride-d_2) δ -158.96 (t, J = 55.0 Hz, 1F, rotamer B), -170.30 (td, J = 53.5, 3.7 Hz, 1F, rotamer A). **^{13}C NMR** (151 MHz, Methylene chloride-d_2) δ 163.4 (rotamer B), 163.0 (d, J = 3.2 Hz, rotamer A), 141.0 (rotamer B), 140.9 (rotamer A), 140.5 (rotamer B), 140.1 (rotamer A), 139.5 (rotamer A or B), 139.5 (rotamer B or A), 129.3 (rotamer A), 129.3 (rotamer B), 128.8 (rotamer A), 128.2 (rotamer B or A), 128.2 (rotamer A or B), 128.0 (rotamer B or A), 127.5 (rotamer B), 127.4 (rotamer A), 126.3 (rotamer A+B), 124.6 (rotamer A or B), 124.6 (rotamer B or A), 90.1 (d, J = 200.6 Hz, rotamer B), 83.8 (d, J = 196.8 Hz, rotamer A). **HRMS** (EI): m/z $[M]^+$ calculated for $C_{14}H_{12}ONF$: 229.0897, found 229.0897. **IR** (neat, cm^{-1}): 1701, 1607, 1483, 1343, 1318, 1287, 1236, 1096, 998, 933, 834, 764, 727, 696.

Note: Similar yield (32%) was obtained using general procedure GP-7.

_N_-(2-Bromophenethyl)-_N_-(1-fluoro-2,2-dimethylpropyl)formamide (35)

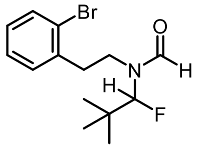

Prepared according to general procedure GP-7 from (2-bromophenethyl)(1-fluoro-2,2-dimethylpropyl)carbamic fluoride (**25**, 134 mg, 0.400 mmol, 1.00 equiv) and sodium borohydride (30.3 mg, 0.800 mmol, 2.00 equiv). The title compound **35** was obtained as a colorless oil (54.7 mg, 0.173 mmol, 43 %). R_f = 0.36 (pentane/Et_2O/Et_3N 85:10:5 *v/v*).

^{1}H NMR (600 MHz, Acetonitrile-d_3) δ 8.27 (s, 1H, rotamer A), 8.04 (d, J = 2.6 Hz, 1H, rotamer B), 7.61 – 7.58 (m, 1H, rotamer B), 7.57 (dd, J = 8.0, 1.1 Hz, 1H, rotamer A), 7.35 – 7.28 (m, 2H, rotamer A + B), 7.19 – 7.16 (m, 1H, rotamer B), 7.14 (ddd, J = 8.0, 6.7, 2.3 Hz, 1H, rotamer A), 6.04 (d, J = 43.4 Hz, 1H, rotamer B), 5.29 (d, J = 42.8 Hz, 1H, rotamer A), 3.69 (dddd, J = 14.6, 9.2, 5.2, 2.1 Hz, 2H, rotamer B), 3.63 – 3.53 (m, 2H, rotamer A), 3.14 (ddd, J = 13.9, 9.1, 5.2 Hz, 1H, rotamer B), 3.05 (ddd, J = 8.8, 6.9, 1.6 Hz, 2H, rotamer A), 3.10 – 2.97 (m, 1H, rotamer B), 1.04 (d, J = 1.0 Hz, 9H, rotamer A), 1.01 (d, J = 1.1 Hz, 9H, rotamer B). **^{19}F NMR** (564 MHz, Acetonitrile-d_3) δ −153.38 (d, J = 42.9 Hz, 1F, rotamer A), −165.52 (d, J = 43.4 Hz, 1F, rotamer B). **^{13}C NMR** (151 MHz, Acetonitrile-d_3) δ 165.2 (d, J = 31.7 Hz, rotamer B), 164.4 (d, J = 36.0 Hz, rotamer A), 139.5 (rotamer A), 138.7 (rotamer A), 133.8 (rotamer B), 133.7 (rotamer A), 132.6 (rotamer B), 132.2 (rotamer A), 129.7 (rotamer B), 129.4 (rotamer A), 128.9 (rotamer B), 128.8 (rotamer A), 125.0 (rotamer B), 124.9 (rotamer A), 105.9 (d, J = 206.4 Hz, rotamer A), 98.9 (d, J = 201.9 Hz, rotamer B), 44.9 (rotamer B), 42.7 (rotamer A), 39.3 (rotamer B), 37.4 (d, J = 27.0 Hz, rotamer A), 37.4 (d, J = 25.2 Hz, rotamer B), 35.6 (rotamer A), 25.8 (d, J = 2.9 Hz, rotamer A), 25.7 (d, J = 3.2 Hz, rotamer B). **HRMS** (APCI): m/z $[M + H]^+$ calculated for $C_{14}H_{20}BrFNO$ 316.0707, found: 316.0755. **IR** (neat, cm^{-1}): 2965, 2877, 1684, 1471, 1440, 1368, 1304, 1231, 1194, 1149, 1114, 1028, 991, 789, 751.

_N_-(9-Ethyl-9_H_-carbazol-3-yl)-_N_-(1-fluoro-2,2-dimethylpent-4-en-1-yl)formamide-_d_ (36)

Prepared according to general procedure GP-7 from (9-ethyl-9*H*-carbazol-3-yl)(1-fluoro-2,2-dimethylpent-4-en-1-yl)carbamic fluoride (**21**, 74.1 mg, 0.200 mmol, 1.00 equiv) and sodium borodeuteride (16.7 mg, 0.400 mmol, 2.00 equiv). The title compound **36** was obtained as a light-yellow oil (25.9 mg, 0.0738 mmol, 37 %). *Note: The title compound was obtained as a mixture of two diastereomers (d. r. = 1:1.8).* R_f = 0.23 (pentane/Et_2O/Et_3N 75:20:5 *v/v*). **^{1}H NMR** (600 MHz, Acetonitrile-d_3) δ 8.21 – 7.97

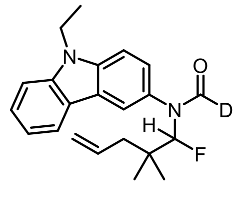

(m, 2H, rotamer A+B), 7.59 – 7.46 (m, 4H, rotamer A+B), 7.28 – 7.18 (m, 1H, rotamer A+B), 6.46 (d, *J* = 45.1 Hz, 1H, rotamer A), 5.82 (ddt, *J* = 17.4, 9.8, 7.5 Hz, 1H, rotamer A+B), 5.67 (d, *J* = 45.5 Hz, 1H, rotamer B), 5.09 – 4.99 (m, 1H, rotamer A+B), 4.42 (q, *J* = 7.2 Hz, 2H, *rotamer A*), 4.42 (q, *J* = 7.2 Hz, 2H, *rotamer B*), 2.14 – 2.07 (m, 1H, *rotamer A + B*), 2.06 – 1.96 (m, 1H, *rotamer A + B*), 1.38 (t, *J* = 7.2 Hz, 3H, *rotamer B*) 1.38 (t, *J* = 7.2 Hz, 3H, *rotamer A*), 0.78 (s, 3H, *rotamer B*), 0.77 (s, 3H, *rotamer A*), 0.76 (s, 3H, *rotamer B*), 0.72 (s, 3H, *rotamer A*). **^{19}F NMR** (564 MHz, CD_3CN) δ −147.45 (d, *J* = 45.5 Hz, 1F, *rotamer B*), −158.02 (d, *J* = 45.1 Hz, 1F, *rotamer* A). **^{13}C NMR** (151 MHz, CD_3CN) *δ* 165.9 (t, *J* = 30.1 Hz, rotamer B), 165.8 (t, *J* = 32.2 Hz, rotamer A), 141.5 (rotamer A), 141.4 (rotamer B), 140.2 (rotamer A), 140.1 (rotamer B), 134.9 (rotamer A), 134.8 (rotamer B), 130.8 (d, *J* = 2.3 Hz, rotamer A), 128.7 (d, *J* = 2.7 Hz, rotamer B), 127.7 (d, *J* = 2.3 Hz, rotamer B), 127.4 (rotamer A), 127.3 (d, *J* = 2.5 Hz, rotamer A), 127.2 (rotamer B), 123.8 (rotamer A), 123.6 (rotamer B), 123.3 (rotamer B), 123.2 (rotamer A), 121.9 (d, *J* = 2.2 Hz, rotamer B), 121.7 (d, *J* = 2.5 Hz, rotamer A), 121.5 (rotamer A), 121.5 (rotamer B), 120.2 (rotamer A), 120.1 (rotamer B), 118.8 (rotamer B), 118.8 (rotamer A), 110.2 (rotamer A), 110.1 (rotamer A), 110.1 (rotamer B), 109.9 (rotamer B), 105.5 (d, *J* = 207.6 Hz, rotamer B), 96.9 (d, *J* = 203.5 Hz, rotamer A), 44.1 (d, *J* = 2.1 Hz, rotamer A), 44.0 (d, *J* = 2.3 Hz, rotamer B), 39.9 (d, *J* = 25.2 Hz, rotamer B), 39.6 (d, *J* = 24.7 Hz, rotamer A), 38.4 (rotamer A), 38.4 (rotamer B), 23.6 (d, *J* = 1.2 Hz, rotamer B), 23.4 (d, *J* = 1.8 Hz, rotamer A), 22.6 (d, *J* = 2.5 Hz, rotamer B), 22.3 (d, *J* = 3.1 Hz, rotamer A), 14.02 (rotamer A), 13.99 (rotamer B). **HRMS** (ESI): *m/z* $[M + Na]^+$ calculated for $C_{22}H_{24}DFN_2NaO$ 376.1906, found: 376.1905. **IR** (neat, cm^{-1}): 3069, 2974, 2935, 1711, 1600, 1663, 1483, 1382, 1343, 1304, 1230, 1151, 1086, 997, 970, 918, 809, 776, 747, 674.

7.5. Characterization data of *N*-CH_2F and *N*-CHRF carbamoyl azides

(Fluoromethyl)(3,4,5-trimethoxyphenyl)carbamoyl azide (37)

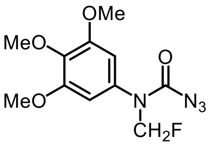

Prepared according to general procedure GP-8 for *N*-CH_2F carbamoyl azide on a 0.10 mmol scale, and isolated as a colorless oil (24 mg, 0.083 mmol, 83 %). ***R$_f$*** = 0.17 (12 % EtOAc/pentane + 3 % Et_3N).

^{1}H NMR (600 MHz, Acetonitrile-*d$_3$*) *δ* 6.61 (s, 2H), 5.62 (d, *J* = 54.0 Hz, 2H), 3.80 (s, 6H), 3.73 (s, 3H). **^{19}F NMR** (564 MHz, Acetonitrile-*d$_3$*) *δ* -165.2 (brs, 1F, rotamer B), -169.8 (s, 1F, rotamer A). **^{13}C NMR** (151 MHz, Acetonitrile-*d$_3$*) *δ* 154.9, 154.9, 139.3, 137.0, 106.7, 89.9 (d, *J* = 200.5 Hz), 61.3, 57.3. **HRMS** (ESI): *m/z* $[M + Na]^+$ calculated for $C_{11}H_{13}FN_4O_4Na$: 307.0813, found 307.0811. **IR** (neat, cm^{-1}): 2943, 2146, 1689, 1594, 1502, 1456, 1220, 1120, 1004, 726, 691.

(1-(2,6-Dimethylphenoxy)propan-2-yl)(fluoromethyl)carbamoyl azide (38)

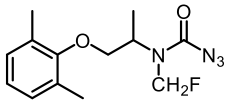

Prepared according to general procedure GP-8 for *N*-CH_2F carbamoyl azide on a 0.20 mmol scale, and isolated as a yellow oil (21 mg, 0.15 mmol, 73 %). ***R$_f$***=0.43 (5 % EtOAc/pentane).

^{1}H NMR (600 MHz, Acetonitrile-*d$_3$*) *δ* 7.01 (d, *J* = 7.5 Hz, 2H), 6.92 (t, *J* = 7.5 Hz, 1H), 5.55 (d, *J* = 54.3 Hz, 2H), 4.63 (h, *J* = 6.7 Hz, 1H, rotamer A), 4.43 (h, *J* = 6.8 Hz, 1H, rotamer B), 3.90 – 3.70 (m, 2H), 2.22 (s, 6H), 1.40 (d, *J* = 7.1 Hz, 3H). **^{19}F NMR** (564 MHz, Acetonitrile-*d$_3$*) *δ* -164.17 (t, *J* = 54.5 Hz, 1F, rotamer A), -168.38 (t, *J* = 54.0 Hz, 1F, rotamer B). **^{13}C NMR** (151 MHz, Acetonitrile-*d$_3$*) *δ* 159.3 (rotamer B), 158.3 (rotamer A), 156.0 (rotamer A+B), 131.7 (rotamer A+B), 129.8 (rotamer A+B), 125.1 (rotamer A+B), 85.8 (d, *J* = 195.6 Hz, rotamer A), 84.4 (d, *J* = 194.1 Hz, rotamer B), 74.1 (rotamer B), 73.7 (rotamer A), 53.9 (rotamer B), 53.3 (rotamer A), 16.3 (rotamer A+B), 15.8 (rotamer A+B). **HRMS** (APCI): $[M - F]^+$ calculated for $C_{13}H_{17}O_2N_4$: 261.1346, found 261.1352. **IR** (neat, cm^{-1}): 2147, 1685, 1473, 1410, 1199, 1084, 1018, 769, 737.

Note: All our efforts (EI, ESI, APCI) to measure molecular ion failed.

((4-Chlorophenyl)fluoromethyl)(cyclohexyl)carbamoyl azide (39)

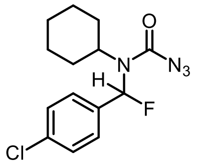

Prepared according to general procedure GP-9 for *N*-CHRF carbamoyl azides from ((4-chloro-phenyl)fluoromethyl)(cyclohexyl)carbamic fluoride (**24**, 28.7 mg, 0.100 mmol, 1.00 equiv), with a reaction time of 1 h. The title compound **39** was obtained as a colorless oil (27.6 mg, 0.0888 mmol, 89 %). $\boldsymbol{R_f}$ = 0.25 (pentane/Et_2O/Et_3N 93:2:5 *v/v*).
^{1}H NMR (600 MHz, Acetonitrile-*d_3*) δ 7.45 (d, *J* = 8.5 Hz, 2H), 7.38 (d, *J* = 8.5 Hz, 2H), 7.02 (d, *J* = 47.4 Hz, 1H), 3.23 (s, 1H), 1.95 (d, *J* = 45.3 Hz, 2H), 1.79 – 1.70 (m, 2H), 1.63 (d, *J* = 11.3 Hz, 1H), 1.53 (d, *J* = 12.1 Hz, 1H), 1.36 (s, 1H), 1.22 (qt, *J* = 13.2, 3.9 Hz, 1H), 1.07 (qt, *J* = 12.8, 3.4 Hz, 1H), 1.02 – 0.94 (m, 1H). **^{19}F NMR** (564 MHz, Acetonitrile-*d_3*) δ -152.62 (s, 1F, rotamer A), -154.83 (s, 1F, rotamer B). **^{13}C NMR** (151 MHz, Acetonitrile-*d_3*) δ 157.2, 136.4 (d, *J* = 31.4 Hz), 135.1, 129.5, 128.0 (d, *J* = 7.0 Hz), 96.4 (d, *J* = 203.8 Hz), 57.7, 31.6, 31.0, 26.8 (d, *J* = 11.9 Hz), 25.9. **HRMS** (APCI): *m/z* $[M - F]^+$ calculated for $C_{14}H_{16}ClN_4O$ 291.1008, found: 291.1075. **IR** (neat, cm^{-1}): 2933, 2857, 2149, 1697, 1491, 1428, 1380, 1338, 1282, 1224, 1179, 1134, 1091, 982, 955, 904, 876, 807, 740, 705, 664.
Note: All our efforts (EI, ESI, APCI) to measure molecular ion failed.

7.6. Characterization data of *N*-CH_2F and *N*-CHRF carbamoyl cyanides

(Fluoromethyl)(2-methoxy-5-methylphenyl)carbamoyl cyanide (40)

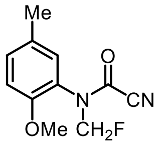

Prepared according to general procedure GP-8 for *N*-CH_2F carbamoyl cyanides on a 0.20 mmol scale, and isolated as a yellow oil (35.3 mg, 0.160 mmol, 80 %). $\boldsymbol{R_f}$ =0.20 (5 % EtOAc/pentane).
^{1}H NMR (600 MHz, Acetonitrile-*d_3*) δ 7.36 (ddd, *J* = 8.5, 2.2, 0.9 Hz, 1H), 7.26 (d, *J* = 2.2 Hz, 1H), 7.09 (d, *J* = 8.5 Hz, 1H), 5.79 (dd, *J* = 51.9, 8.2 Hz, 2H, rotamer A), 5.47 (dd, *J* = 52.4, 8.2 Hz, 2H, rotamer B), 3.85 (s, 3H, rotamer A), 3.79 (s, 3H, rotamer B), 2.33 (s, 3H, rotamer A), 2.29 (s, 3H, rotamer B). **^{19}F NMR** (564 MHz, Acetonitrile-*d_3*) δ -168.55 (t, *J* = 53.5 Hz, 1F rotamer B), -177.65 (t, *J* = 52.1 Hz, 1F, rotamer A). **^{13}C NMR** (151 MHz, Acetonitrile-*d_3*) δ 154.4 (rotamer A), 153.2 (rotamer B), 147.5 (d, *J* = 2.7 Hz, rotamer A), 146.0 (rotamer B), 133.4 (rotamer A+B), 132.3 (rotamer B), 132.1 (rotamer A), 130.9 (rotamer A+B), 129.8 (rotamer B), 126.3 (d, *J* = 1.9 Hz, rotamer A), 113.6 (rotamer A), 113.5 (rotamer B), 111.7 (rotamer A), 111.2 (rotamer B), 90.55 (d, *J* = 202.7 Hz, rotamer B), 86.25 (d, *J* = 200.1 Hz, rotamer A), 56.8 (rotamer A), 56.7 (rotamer B), 20.2 (rotamer A+B). **HRMS** (EI): *m/z* $[M]^+$ calculated for $C_{11}H_{11}O_2N_2F$: 222.0799, found 222.0799. **IR** (neat, cm^{-1}): 1700, 1510, 1460, 1371, 1264, 988, 814, 741.

Methyl *N*-(cyanocarbonyl)-*N*-(fluoromethyl)- *L*-phenylalaninate (41)

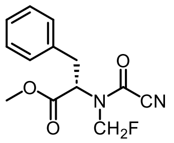

Prepared according to general procedure GP-8 for *N*-CH_2F carbamoyl cyanides on a 0.10 mmol scale, and isolated as a colorless oil (20 mg, 0.074 mmol, 74 %). $\boldsymbol{R_f}$ = 0.26 (10 % EtOAc/pentane).
^{1}H NMR (600 MHz, Acetonitrile-*d_3*) δ 7.37 – 7.23 (m, 5H), 5.71 (dd, *J* = 53.9, 10.6 Hz, 1H, rotamer A, H_a), 5.67 (dd, *J* = 52.2, 9.0 Hz, 1H, rotamer B, H_a), 5.50 (dd, *J* = 52.7, 10.7 Hz, 1H, rotamer A, H_b), 5.47 (dd, *J* = 51.1, 8.9 Hz, 1H, rotamer B, H_b).5.06 – 5.04 (m, 1H, rotamer B), 5.01 – 4.99 (m, 1H, rotamer A), 3.76 (s, 3H, rotamer B), 3.73 (s, 3H, rotamer A), 3.50 (dd, *J* = 14.5 ,5.2 Hz, 1H, rotamer B), 3.43 (dd, *J* = 14.5, 5.9 Hz, 1H, rotamer A), 3.19 – 3.11 (m, 1H). **^{19}F NMR** (564 MHz, Acetonitrile-*d_3*) δ -168.3 (t, *J* = 53.3 Hz, 1F, rotamer A), -178.3 (t, *J* = 51.8 Hz, 1F, rotamer B). **^{13}C NMR** (151 MHz, Acetonitrile-*d_3*) δ 169.8 (rotamer B), 169.6 (rotamer A), 147.5 (rotamer B), 147.0 (rotamer A), 137.2 (rotamer A), 136.5 (rotamer B), 130.4 (rotamer B), 130.0 (rotamer A), 129.8 (rotamer B), 129.6 (rotamer A), 128.3 (rotamer B), 128.0 (rotamer A), 110.5, 88.1 (d, *J* = 201.8 Hz, rotamer A), 83.1 (d, *J* = 198.7 Hz, rotamer B), 63.6 (rotamer B), 60.8 (rotamer

A), 53.8 (rotamer B), 53.5 (rotamer A), 36.4 (rotamer B), 35.5 (rotamer A). **IR** (neat, cm^{-1}): 3031, 2956, 2236, 1745, 1693, 1419, 1342, 1247, 1213, 987, 751, 700. *Note: All our efforts (EI, ESI, APCI) to measure molecular ion failed.*

(9-Ethyl-9*H*-carbazol-3-yl)(1-fluoro-2,2-dimethylpent-4-en-1-yl)carbamoyl cyanide (42)

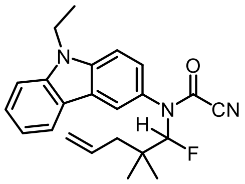

Prepared according to general procedure GP-9 for *N*-CHRF carbamoyl cyanides from (9-ethyl-9*H*-carbazol-3-yl)(1-fluoro-2,2-dimethylpent-4-en-1-yl)carbamic fluoride (**21**, 37.0 mg, 0.100 mmol, 1.00 equiv), with a reaction time of 20 min. The title compound **42** was obtained as an off-white solid (23 mg, 0.060 mmol, 60 %). ***R_f*** = 0.25 (pentane/Et_2O/Et_3N 75:20:5 *v/v*). **M.p.**: 122 – 126 °C.

^{1}H NMR (600 MHz, Acetonitrile-*d_3*) δ 8.38 (d, *J* = 2.1 Hz, 1H, rotamer A), 8.26 (dt, *J* = 2.6, 1.4 Hz, 1H, rotamer B), 8.17 (ddt, *J* = 14.4, 7.8, 1.0 Hz, 2H, rotamer A + B), 7.66 – 7.63 (m, 3H, rotamer A + B), 7.61 – 7.53 (m, 5H, rotamer A + B), 7.29 (dddd, *J* = 9.0, 7.9, 6.9, 1.1 Hz, 2H, rotamer A + B), 6.38 (d, *J* = 44.5 Hz, 1H, rotamer B), 6.37 (d, *J* = 44.4 Hz, 1H, rotamer A), 5.81 (ddtd, *J* = 17.2, 9.6, 7.5, 1.8 Hz, 2H, rotamer A + B), 5.11 – 5.02 (m, 4H, rotamer A + B), 4.46 (q, *J* = 7.2 Hz, 4H, rotamer A + B), 2.14 (td, *J* = 14.6, 7.8 Hz, 2H, rotamer A + B), 2.08 – 1.99 (m, 2H, rotamer A + B), 1.41 (t, *J* = 7.2 Hz, 6H, rotamer A + B), 0.84 (s, 3H, rotamer A or B), 0.82 (s, 3H, rotamer B or A), 0.75 (s, 3H, rotamer A or B), 0.73 (s, 3H, rotamer B or A). **^{19}F NMR** (564 MHz, Acetonitrile-*d_3*) δ −159.45 (d, *J* = 44.4 Hz, 1F, rotamer A), −160.08 (d, *J* = 44.4 Hz, 1F, rotamer B). **^{13}C NMR** (151 MHz, Acetonitrile-*d_3*) δ 148.6 (rotamer A or B), 148.6 (rotamer B or A), 141.8 (rotamer A or B), 141.6 (rotamer B or A), 141.3 (rotamer A or B), 141.2 (rotamer B or A), 134.5 (rotamer A+B), 128.9 (rotamer A or B), 128.5 (d, *J* = 2.5 Hz, rotamer B or A), 127.8 (rotamer A or B), 127.8 (rotamer B or A), 127.5 (rotamer A or B), 127.2 (rotamer B or A), 124.1 (rotamer B or A), 124.1 (rotamer A or B), 123.7 (d, *J* = 2.1 Hz, rotamer A or B), 123.6 (rotamer A+B), 123.0 (d, *J* = 2.9 Hz, rotamer B or A), 121.6 (rotamer A+B), 120.7 (rotamer A or B), 120.7 (rotamer B or A), 119.2 (rotamer A or B), 119.1 (rotamer B or A), 111.7 (rotamer A or B), 111.7 (rotamer B or A), 110.6 (rotamer A or B), 110.5 (rotamer B or A), 110.4 (rotamer A or B), 110.1 (rotamer B or A), 98.2 (d, *J* = 209.2 Hz, rotamer A or B), 98.2 (d, *J* = 209.5 Hz, rotamer B or A), 43.8 (rotamer A or B), 43.8 (rotamer B or A), 39.8 (d, *J* = 23.2 Hz, rotamer A or B), 39.7 (d, *J* = 23.1 Hz, rotamer B or A), 38.6 (rotamer A or B), 38.6 (rotamer B or A), 22.9 (d, *J* = 2.8 Hz, rotamer A or B) 22.8 (d, *J* = 2.4 Hz, rotamer B or A), 21.9 (d, *J* = 3.6 Hz, rotamer A or B), 21.8 (d, *J* = 3.6 Hz, rotamer B or A), 14.0 (rotamer A or B), 14.0 (rotamer B or A). **HRMS** (APCI): *m/z* $[M]^+$ calculated for $C_{23}H_{24}F_1N_3O$ 377.1903, found: 377.1958. **IR** (neat, cm^{-1}): 3073, 2980, 2935, 1687, 1597, 1471, 1309, 1247, 1226, 1147, 1126, 1089, 1056, 990, 917, 897, 865, 814, 751, 715.

7.7. Characterization data of *N*-CH_2F and *N*-CHRF alkynamides

***N*-([1,1'-Biphenyl]-4-yl)-*N*-(fluoromethyl)-3-phenylpropiolamide (43)**

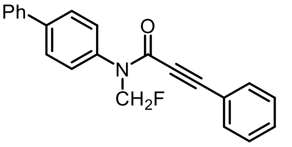

Prepared according to general procedure GP-10 on a 0.50 mmol scale, and isolated as a red-orange oil (114 mg, 0.348 mmol, 69 %). ***R_f*** = 0.33 (10 % EtOAc/pentane + 3 % Et_3N).

^{1}H NMR (600 MHz, Acetonitrile-*d_3*) δ 7.77 – 7.69 (m, 4H), 7.56 – 7.41 (m, 7H), 7.29 – 7.18 (m, 3H), 6.07 (d, *J* = 53.6 Hz, 2H, rotamer A), 5.79 (d, *J* = 53.8 Hz, 2H rotamer B). **^{19}F NMR** (564 MHz, Acetonitrile-*d_3*) δ -163.05 – -163.35 (m, 1F, rotamer A), - 170.86 – 171.15 (m, 1F, rotamer B). **^{13}C NMR** (151 MHz, Acetonitrile-*d_3*) δ 155.6 (rotamer B), 155.4 (rotamer A), 142.5 (rotamer A+B), 140.8 (rotamer B), 140.7 (rotamer A), 133.7 (rotamer B), 133.3 (rotamer A), 132.0 (rotamer B), 131.8 (rotamer A), 130.0 (rotamer A+B), 129.9 (rotamer A+B), 129.7 (rotamer A+B), 128.9 (rotamer A+B), 128.8 (rotamer A+B), 128.2 (rotamer A+B), 128.0 (rotamer A+B), 120.2 (rotamer A+B), 93.4 (rotamer A or B), 92.3 (rotamer

A+B), 91.0 (rotamer B or A), 86.5 (d, *J* = 195.7 Hz, rotamer A+B). **HRMS** (ESI): *m/z* $[M + Na]^+$ calculated for $C_{22}H_{16}FNONa$: 352.1108, found 352.1097. **IR** (neat, cm^{-1}): 3033, 2924, 2216, 1660, 1486, 1367, 1309, 1223, 1074, 955, 842, 756, 692.

N-(2-Bromophenethyl)-3-(4-bromophenyl)-N-(fluoromethyl)propiolamide (44)

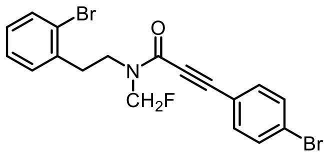

Prepared according to general procedure GP-10 on a 0.10 mmol scale, and isolated as a yellow solid (26.5 mg, 0.0607 mmol, 61 %). ***Rf*** = 0.42 (10 % EtOAc/pentane + 3 % Et_3N). **M.p.**: 91 – 93 °C.

^{1}H NMR (600 MHz, Acetonitrile-*d3*) δ 7.63 – 7.44 (m, 5H), 7.34 – 7.26 (m, 2H), 7.18 – 7.11 (m, 1H), 5.73 (d, *J* = 54.9 Hz, 2H, rotamer A), 5.57 (d, *J* = 53.7 Hz, 2H, rotamer B), 4.01 (t, *J* = 6.9 Hz, 2H, rotamer B), 3.76 (t, *J* = 7.7 Hz, 2H, rotamer A), 3.13 (t, *J* = 6.9 Hz, 2H, rotamer B), 3.05 (t, *J* = 7.4 Hz, 2H, rotamer A). **^{19}F NMR** (564 MHz, Acetonitrile-*d3*) δ -166.19 (t, *J* = 54.8 Hz, 1F, rotamer A), -173.99 (t, *J* = 53.7 Hz, 1F, rotamer B). **^{13}C NMR** (151 MHz, Acetonitrile-*d3*) δ 155.6, 138.9 (rotamer A), 138.4 (rotamer B), 135.1 (rotamer A), 135.1 (rotamer B), 133.8 (rotamer B), 133.7 (rotamer A), 133.0 (rotamer A), 132.9 (rotamer B), 132.7 (rotamer A or B), 132.2 (rotamer B or A), 129.7 (rotamer B), 129.6 (rotamer A), 128.9 (rotamer A), 128.8 (rotamer B), 125.8, 125.6, 125.4, 125.0, 119.8 (rotamer B), 119.7 (rotamer A), 90.6 (d, *J* = 195.6 Hz, rotamer A), 90.2 (rotamer B), 89.7 (rotamer A), 85.6 (d, *J* = 193.2 Hz, rotamer B), 82.3 (rotamer B), 82.0 (rotamer A), 49.6 (rotamer B), 46.3 (rotamer A), 36.9 (rotamer B), 35.4 (rotamer A). **HRMS** (ESI): *m/z* $[M + Na]^+$ calculated for $C_{18}H_{14}FNOBr_2Na$: 459.9318 $[M+Na]^+$, found 459.9323. **IR** (neat, cm^{-1}): 2928, 2856, 2218, 1906, 1734, 1638, 1585, 1478, 1441, 1411, 1385, 1330, 1271, 1234, 1159, 1064, 934, 817, 745, 659.

N-(Fluoro(phenyl)methyl)-N-phenyl-3-(3-(trifluoromethyl)phenyl)propiolamide (45)

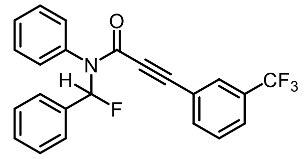

Prepared according to general procedure GP-10 from (fluoro(phenyl)methyl)(phenyl)carbamic fluoride (**16**, 49.7 mg, 0.200 mmol, 1.00 equiv) and trimethyl((3-(trifluoromethyl)phenyl)-ethynyl)silane (70.2 µL, 72.7 mg, 0.300 mmol, 1.50 equiv). The title compound **45** was obtained as a yellow oil (52.8 mg, 0.133 mmol, 66 %). ***Rf*** = 0.39 (pentane/Et_2O/Et_3N 85:10:5 *v/v*).

^{1}H NMR (600 MHz, CD_3CN) δ 7.79 (d, *J* = 49.2 Hz, 1H), 7.70 (d, *J* = 8.1 Hz, 1H), 7.49 (t, *J* = 7.9 Hz, 1H), 7.38 – 7.14 (m, 12H). **^{19}F NMR** (564 MHz, CD_3CN) δ -63.50 (s, 3F, rotamer B), -63.73 (s, 3F, rotamer A), -142.08 (d, *J* = 50.0 Hz, 1F, rotamer B), -149.23 (d, *J* = 49.3 Hz, 1F, rotamer A). **^{13}C NMR** (151 MHz, CD_3CN) δ 155.6, 137.9, 136.8, 135.9 (d, *J* = 27.8 Hz), 131.8, 131.3 (q, *J* = 32.4 Hz), 130.7, 130.4, 130.0, 129.8, 129.7, 129.1, 128.2 (d, *J* = 3.6 Hz), 127.0 (d, *J* = 7.2 Hz), 124.5 (q, *J* = 270.1 Hz), 121.4, 92.8 (d, *J* = 201.7 Hz), 91.5, 83.7. **HRMS** (EI): *m/z* $[M]^+$ calculated for $C_{23}H_{15}F_4NO$ 397.1084, found: 397.1080. **IR** (neat, cm^{-1}): 3067, 2974, 2222, 1659, 1595, 1491, 1434, 1386, 1333, 1269, 1210, 1170, 1129, 1071, 974, 907, 883, 804, 695, 665.

N-Allyl-N-(fluoro(phenyl)methyl)-3-(thiophen-2-yl)propiolamide (46)

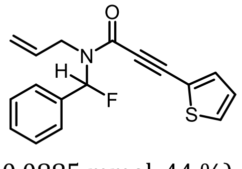

Prepared according to general procedure GP-10 from allyl(fluoro(phenyl)methyl)carbamic fluoride (**23**, 42.2 mg, 0.200 mmol, 1.00 equiv) and trimethyl(thiophen-2-ylethynyl)silane (54.1 mg, 0.300 mmol, 1.50 equiv). The title compound **46** was obtained as a yellow oil (26.5 mg, 0.0885 mmol, 44 %). ***Rf*** = 0.49 (pentane/Et_2O/Et_3N 75:20:5 *v/v*).

^{1}H NMR (600 MHz, Acetonitrile-*d3*) δ 7.65 (d, *J* = 1.2 Hz, 1H, rotamer B), 7.65 (d, *J* = 1.2 Hz, 1H, rotamer A), 7.60 (d, *J* = 48.5 Hz, 1H, rotamer A), 7.59 (dd, *J* = 3.6, 1.2 Hz, 1H, rotamer A), 7.58 (d, *J* = 48.5 Hz, 1H, rotamer B), 7.55 (d, *J* = 3.9 Hz, 1H, rotamer B), 7.48 – 7.40 (m, 10H, rotamer A + B), 7.14 (d, *J* = 3.7 Hz, 1H, rotamer

A), 7.13 (d, J = 3.8 Hz, 1H, rotamer B), 5.69 (ddt, J = 16.3, 10.8, 5.7 Hz, 1H, rotamer B), 5.60 (ddt, J = 16.4, 10.9, 5.7 Hz, 1H, rotamer A), 5.12 – 5.02 (m, 2H, rotamer B), 4.98 – 4.89 (m, 2H, rotamer A), 4.20 (dd, J = 17.3, 5.8 Hz, 2H, rotamer B), 3.99 (ddt, J = 15.9, 5.9, 1.6 Hz, 1H, rotamer A), 3.89 – 3.82 (m, 1H, rotamer B), 3.70 – 3.62 (m, 1H, rotamer A). **^{19}F NMR** (564 MHz, Acetonitrile-d_3) δ -147.36 (d, J = 49.0 Hz, 1F, rotamer A), -154.96 (d, J = 48.3 Hz, 1F, rotamer B). **^{13}C NMR** (151 MHz, Acetonitrile-d_3) δ 156.6 (rotamer B), 155.5 (rotamer A), 137.6 (rotamer A), 137.4 (rotamer B), 136.2 (d, J = 28.5 Hz, rotamer B), 136.1 (d, J = 29.0 Hz, rotamer A), 135.3 (rotamer B), 134.1 (rotamer A), 133.0 (rotamer B), 132.9 (rotamer A), 130.2 (rotamer A), 130.1 (rotamer B), 129.6 (d, J = 1.2 Hz, rotamer A and/or B), 129.0 (rotamer B), 128.9 (rotamer A), 126.7 (d, J = 7.3 Hz, rotamer A and/or B), 119.9 (rotamer B), 119.8 (rotamer A), 117.7 (rotamer B), 117.3 (rotamer A), 99.0 (d, J = 203.0 Hz, rotamer A), 93.0 (d, J = 199.9 Hz, rotamer B), 86.5 (rotamer B), 85.9 (rotamer A), 85.8 (rotamer B), 85.2 (rotamer A), 47.7 (rotamer B), 44.8 (rotamer A). **HRMS** (APCI): m/z [M + H]$^+$ calculated for $C_{17}H_{15}FNOS$ 300.0853, found: 300.0905. **IR** (neat, cm^{-1}): 3085, 2929, 2199, 1718, 1643, 1515, 1399, 1303, 1216, 1180, 1121, 973, 931, 852, 823, 701.

7.7.1. Attempted synthesis of *N*-CH_2F amide[1]

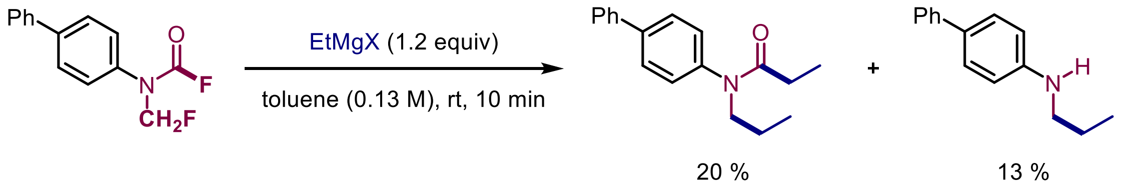

A 4 mL vial was charged with *N*-monofluoromethylcarbamoyl fluoride **1** (24.7 mg, 0.10 mmol, 1.0 equiv), dry toluene (0.75 mL, 0.13 M), and purged with argon. The corresponding Grignard reagent (0.12 mL, 0.12 mmol, 1.2 equiv) was subsequently added to the solution. The reaction mixture was stirred for 10 minutes at room temperature. The reaction mixture was quenched by the addition of saturated aqueous ammonium chloride solution (1.5 mL). The two phases were separated, and the aqueous phase was extracted with EtOAc (2x). Organic phase was dried over $MgSO_4$ and concentrated under reduced pressure. The crude material was then purified by column chromatography on silica gel. The corresponding tertiary amide and secondary amine were isolated in 20 % and 13 % respectively. In addition, 28 % of starting *N*-monofluoromethylcarbamoyl fluoride **1** was recovered.

Note: Similar result was observed when *N*-CHRF carbamoyl fluoride **16** was treated with corresponding Grignard reagent. The product of double-addition was the major species observed in the crude ^{1}H and ^{19}F NMR.

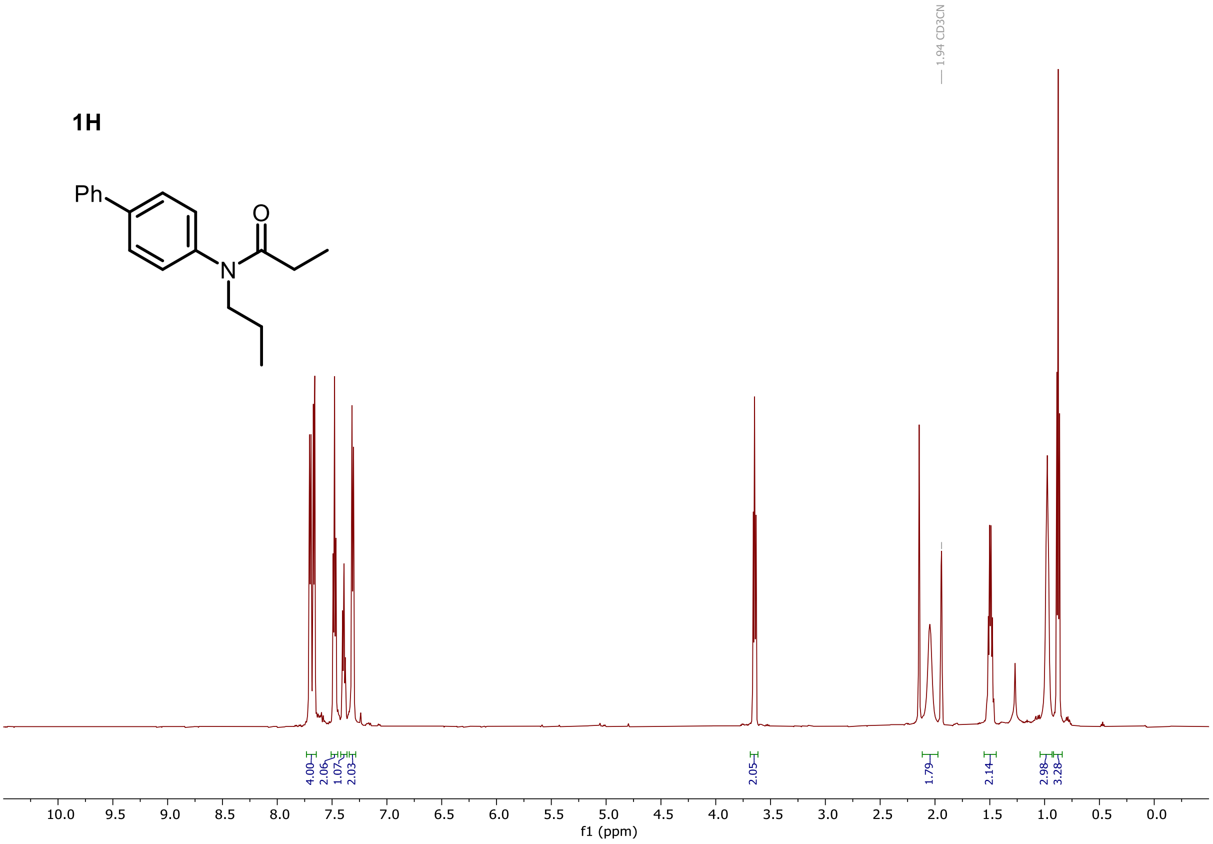

Figure S1: ^{1}H NMR of the double addition product.

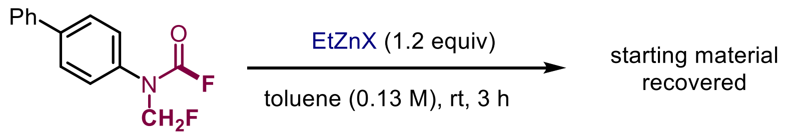

To a dry 4 mL vial under Ar atmosphere were added a solution of ethyl magnesium chloride (0.11 mL, 0.1 mmol, 1.1 equiv.), $ZnCl_2$ (1M in THF, 0.12 mL, 0.12 mmol, 1.2 equiv.) and stirred for 20 minutes. This mixture was subsequently added to another 4 mL vial charged with *N*-monofluoromethylcarbamoyl fluoride **1** (0.10 mmol, 1.0 equiv) in dry toluene (0.75 mL, 0.13 M), and purged with argon. The reaction mixture was stirred for 3 h at room temperature. The reaction mixture was quenched by the addition of saturated aqueous ammonium chloride solution (1.5 mL). The two phases were separated, and the aqueous phase was extracted with EtOAc (2x). Organic phase was dried over $MgSO_4$ and concentrated under reduced pressure. *N*-monofluoromethylcarbamoyl fluoride **1** was the only observed species in ^1H and ^{19}F NMR and recovered from the crude mixture.

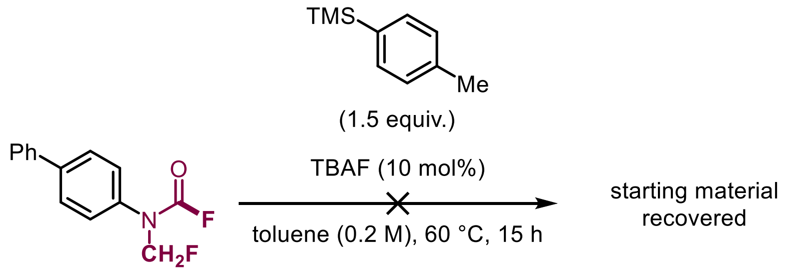

A 4 mL vial was charged with the corresponding *N*-monofluoromethylcarbamoyl carbamoyl fluoride **1** (24.7 mg, 0.1 mmol, 1.0 equiv) and toluene (0.5 mL, 0.20 M). Subsequently, the respective trimethyl(p-tolyl)silane (24.6 mg, 0.15 mmol, 1.5 equiv) was added, followed by TBAF (0.01 mL, 10 mol%, as 1.0 M solution in THF). The reaction mixture was stirred at 60 °C for 15 h. The reaction mixture was then cooled to room temperature and filtered through a short plug of Celite® and concentrated *in vacuo*. Starting material *N*-monofluoromethylcarbamoyl carbamoyl fluoride **1** was the only species observed in ^1H and ^{19}F NMR and recovered from the crude mixture.

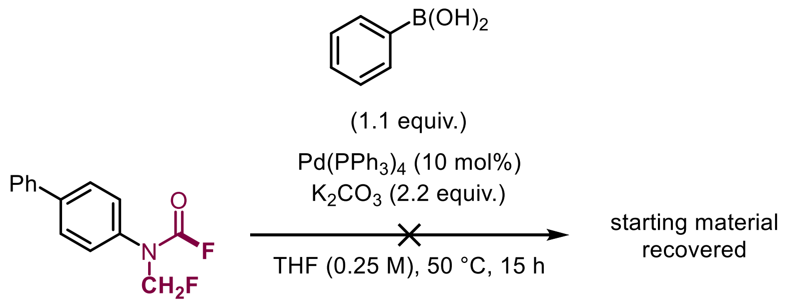

A 4 mL vial was charged with the corresponding *N*-monofluoromethylcarbamoyl carbamoyl fluoride **1** (24.7 mg, 0.1 mmol, 1.0 equiv), phenylboronic acid (13.4 mg, 0.11 mmol, 1.1 equiv.), K_2CO_3 (30.4 mg, 0.22 mmol, 2.2 equiv.), palladium-tetrakis(triphenylphosphine) (11.56 mg, 0.01 mmol, 10 mol%) and THF (0.4 mL, 0.25 M). The reaction mixture was stirred at 50 °C for 15 h. The reaction mixture was then cooled to room temperature and filtered through a short plug of Celite® and concentrated *in vacuo*. Starting material *N*-monofluoromethylcarbamoyl carbamoyl fluoride **1** was the only species observed in ^1H and ^{19}F NMR and recovered from the crude mixture.

Note: Same outcome was observed when the reaction was performed without Pd catalyst.

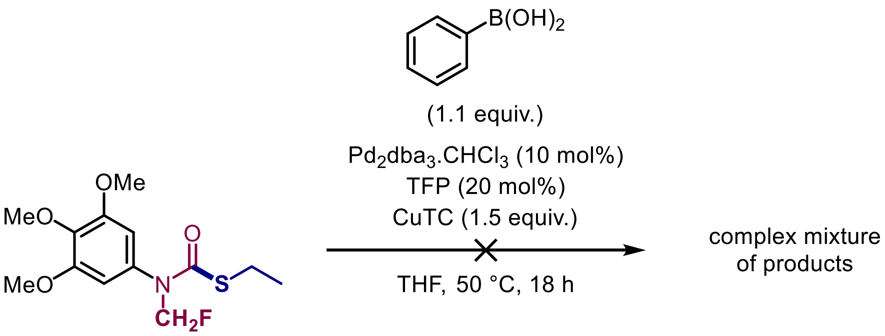

A 4 mL vial was charged with Cu(I)-thiophene-2-carboxylate CuTC (9.4 g, 0.05 mmol, 1.5 equiv.), phenylboronic acid (4.4 mg, 0.036 mmol, 1.1 equiv.), $Pd_2dba_3.CHCl_3$ (3.4 mg, 0.0033 mmol, 10 mol%) and tris-2-furylphosphine TFP (1.5 mg, 0.0066 mmol, 20 mol%). *N*-CH_2F thiocarbamate **29** (10.0 mg, 0.033 mmol, 1 equiv.) in THF (0.4 mL) was added subsequently, and the mixture was stirred for 18 h at 50 °C. The resulting mixture was filtered through pad of Celite® and concentrated *in vacuo*. The ^1H and ^{19}F NMR showed a complex picture of products without the significant peaks of the desired *N*-CH_2F amide.

7.7.2. Attempted synthesis of *N*-CH_2F urea[1]

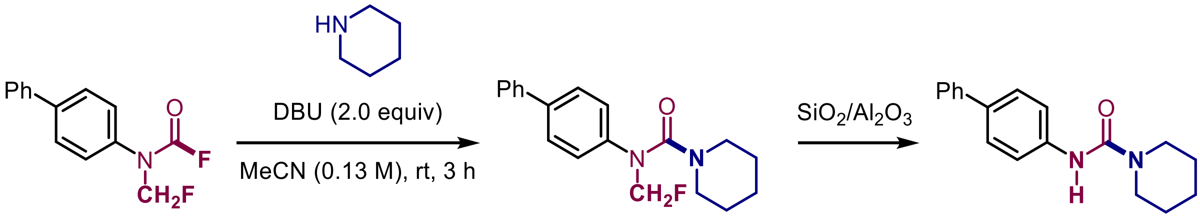

A 4 mL vial was charged with the *N*-CH_2F fluoride **1** (24.7 mg, 0.10 mmol, 1.0 equiv) and MeCN (0.75 mL, 0.13 M). Piperidine (17 mg, 0.20 mmol, 2.0 equiv), DBU (30 µl, 30 mg, 0.20 mmol, 2.0 equiv) were subsequently added to the solution. The reaction mixture was stirred for 3 h at rt. ^1H and ^{19}F NMR of the crude reaction mixture as well as upon column chromatography were recorded and spectra are provided below.

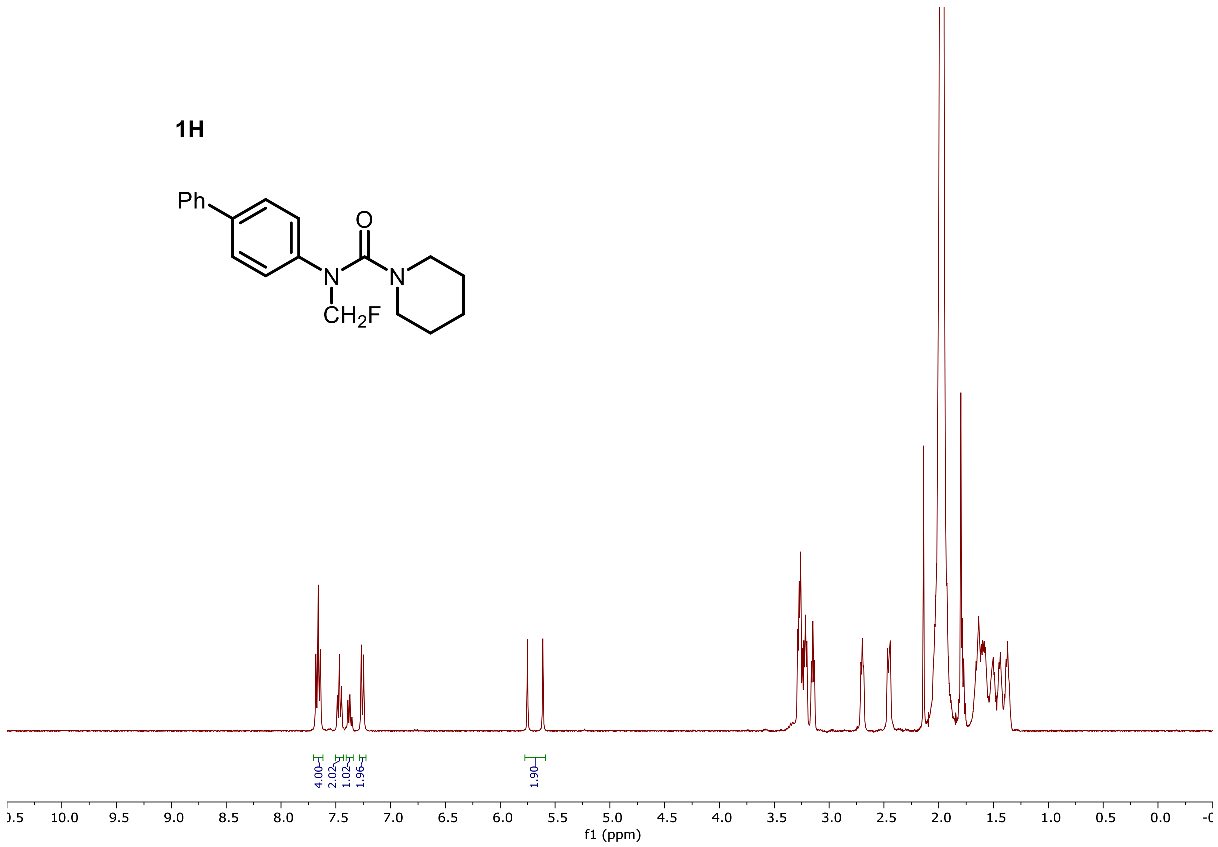

Figure S2: ^{1}H NMR of the crude reaction mixture.

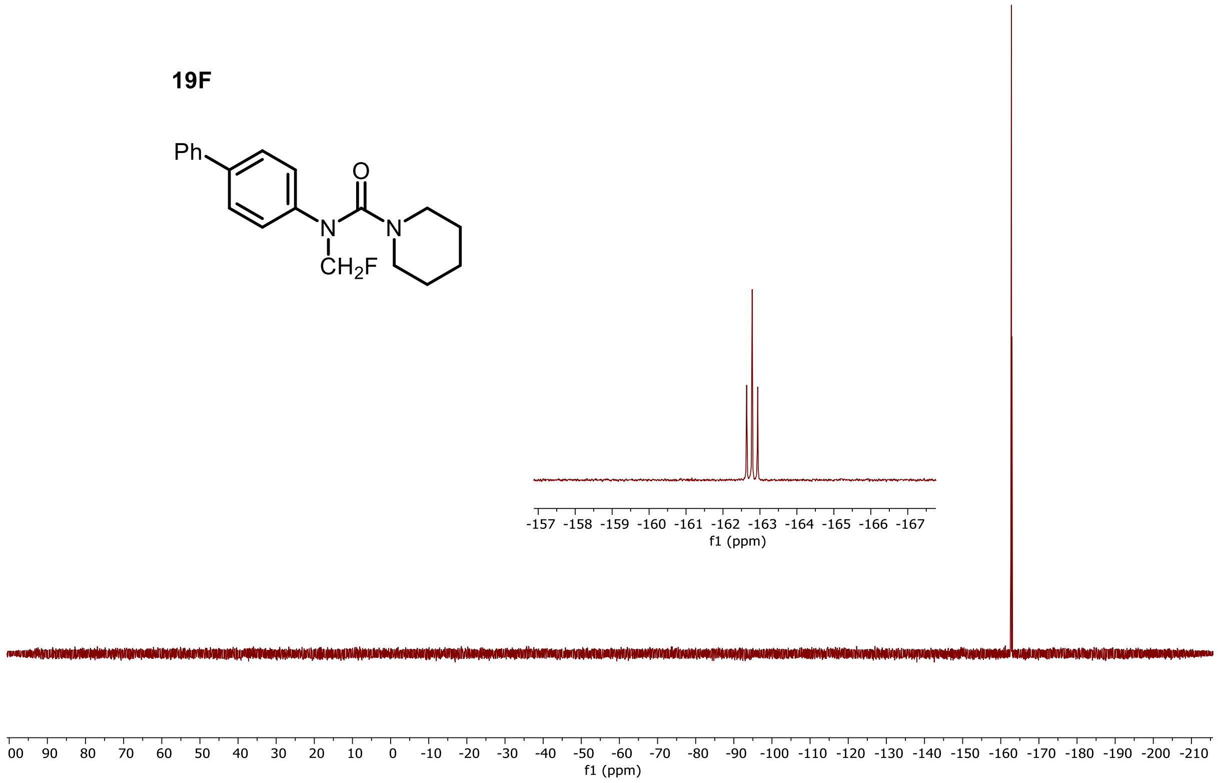

Figure S3: ^{19}F NMR of the crude reaction mixture.

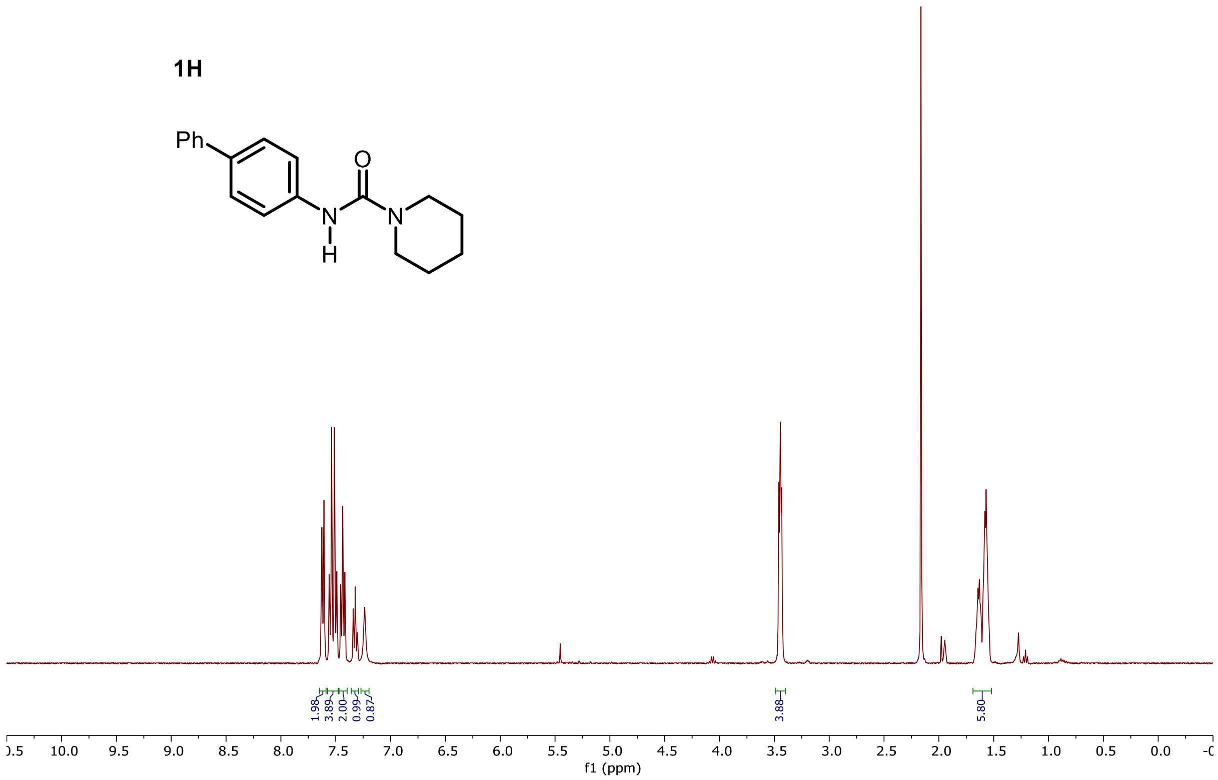

Figure S4: ^{1}H NMR of the isolated (hydrolyzed) product after column chromatography.

8. Synthesis of starting materials

8.1. General procedure (GP-11) for the synthesis of aldimines

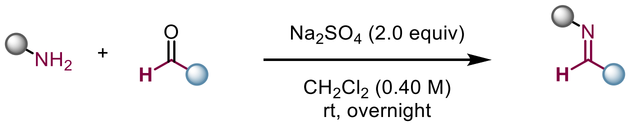

Synthesized according to a modified literature procedure by Li and coworkers.[5] Under ambient atmosphere, a 20 mL glass vial was charged with the amine (1.00 mmol, 1.00 equiv) and sodium sulfate (Na_2SO_4, 284 mg, 2.00 mmol, 2.00 equiv). Subsequently, dichloromethane (2.5 mL, 0.40 M) and the corresponding aldehyde (if liquid; 1.00 – 2.00 mmol, 1.00 – 2.00 equiv, equivalents indicated in each case) were added quickly, the vial was secured tightly with a cap, and the heterogeneous mixture was stirred overnight at room temperature. The resulting mixture was filtered through a short pad of Celite® and washed with dichloromethane (~2.5 mL) to remove the drying agent. The solvent was removed under reduced pressure to obtain the crude product in good purity. The imines were used without further purification. To prevent decomposition, some imines were stored under argon, at −30 °C in the freezer.

Note: In case of solid aldehydes, the order of addition was reversed, and the amine was added last. In case of highly volatile aldehydes or amines, the most volatile component was quickly added as the last reagent of the reaction, and the vial was immediately sealed tightly. The products were obtained in technical grade purity.

8.2. General procedure (GP-12) for the synthesis of aldimines

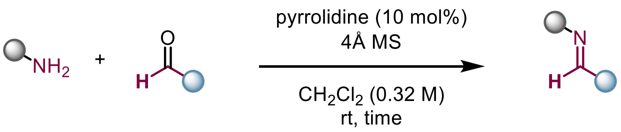

Synthesized according to the literature procedure by Morales and coworkers.[6] Under ambient atmosphere, a 20 mL glass vial was charged with the amine (1.00 mmol, 1.00 equiv), 4 Å molecular sieves (MS, 1 g/mmol) and dichloromethane (3.1 mL, 0.32 M). Subsequently, the corresponding aldehyde (1.00 – 1.20 mmol, 1.0 – 1.2 equiv, equivalents indicated in each case) and pyrrolidine (7.1 mg, 8.3 µL, 0.10 mmol, 10 mol%, or 0.10 mL of a 1.0 M stock solution in dichloromethane) were added quickly, the vial was secured tightly with a cap, and the heterogeneous mixture was stirred at room temperature for the time indicated in each case. The resulting mixture was filtered through a short pad of Celite® and washed with dichloromethane (~2.5 mL) to remove the molecular sieves. The solvent was removed under reduced pressure to obtain the crude product in good purity. The imines were used without further purification. To prevent decomposition some imines were stored under argon, at −30 °C in the freezer.

Note: In case of highly volatile aldehydes or amines, the most volatile component was quickly added as the last reagent, but prior to pyrrolidine and the vial was immediately sealed tightly. The products were obtained in technical grade purity.

8.3. General procedure (GP-13) for the synthesis of TMS-protected alcohols

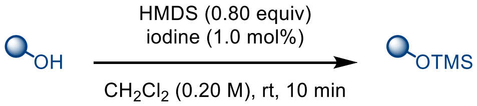

Synthesized according to the literature procedure by Karimi and coworkers.[7] Under ambient atmosphere, in a 20 mL glass vial, iodine (2.5 mg, 0.010 mmol, 0.010 equiv), and the alcohol (1.00 mmol, 1.00 equiv) were dissolved in dichloromethane (4.0 mL, 0.25 M). Subsequently, a solution of hexamethyldisilazane (HMDS, 167 µL, 129 mg, 0.800 mmol, 0.800 equiv) in DCM (1.0 mL, 0.80 M) was added dropwise over 5 min. This resulted in a color change from pink to orange. The reaction mixture was stirred for about 10 min, and the consumption was checked by TLC. After completion of the reaction, finely powdered sodium thiosulfate ($Na_2S_2O_3$, 310 mg, 2.0 mmol, 2.0 equiv) was added and the resulting heterogeneous solution was stirred for additional 30 min until discoloration of the solution occurred. The resulting mixture was filtered through a short pad of silica gel and washed with DCM (~4 mL). The solvent was removed under reduced pressure to obtain the crude product in good purity. The product was used in the next step without further purification.

8.4. Characterization data of aldimines

N-Diphenylmethanimine (S1)

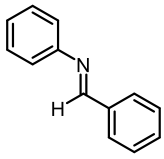

Synthesized according to a modified variant of general procedure GP-11 from aniline (93.1 mg, 1.00 mmol, 1.00 equiv) and benzaldehyde (117 mg, 1.10 mmol, 1.10 equiv), in acetonitrile (2.5 mL, 0.40 M) as a solvent. The title compound **S1** was obtained as yellow oil (185 mg, 1.02 mmol, quant. yield). R_f = 0.54 (pentane/EtOAc 19:1 *v/v*).

^{1}H NMR (400 MHz, $CDCl_3$) δ 8.46 (s, 1H), 7.95 – 7.88 (m, 2H), 7.53 – 7.45 (m, 3H), 7.44 – 7.36 (m, 2H), 7.25 – 7.19 (m, 3H). **^{13}C NMR** (101 MHz, $CDCl_3$) δ 160.5, 152.2, 136.4, 131.5, 129.3, 129.0, 128.9, 126.1, 121.0. **MS** (70 eV, EI): *m/z* (%): 181 (87) [M^+], 180 (100), 104 (10), 77 (41), 51 (12). These data are in agreement with those reported previously in the literature.[8]

1-Phenyl-*N*-(4-(trifluoromethyl)phenyl)methanimine (S2)

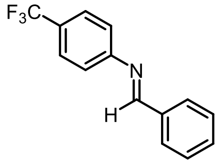

Synthesized according to general procedure GP-11 from 4-(trifluoromethyl)aniline (161 mg, 1.00 mmol, 1.00 equiv) and benzaldehyde (106 mg, 1.00 mmol, 1.00 equiv). The title compound **S2** was obtained as a yellow solid (238 mg, 0.954 mmol, 95 %). R_f = 0.56 (pentane/EtOAc 19:1 *v/v*).

^{1}H NMR (600 MHz, $CDCl_3$) δ 8.43 (s, 1H), 7.94 – 7.91 (m, 2H), 7.67 – 7.64 (m, 2H), 7.54 – 7.48 (m, 3H), 7.26 (d, *J* = 8.9 Hz, 2H). **^{19}F NMR** (564 MHz, $CDCl_3$) δ −62.02 (s, 3F). **^{13}C NMR** (151 MHz, $CDCl_3$) δ 162.1, 155.4, 135.9, 132.1, 129.2, 129.0, 127.9 (q, *J* = 32.6 Hz), 126.5 (q, *J* = 3.8 Hz), 126.2 (q, *J* = 271.2 Hz), 121.1. **MS** (70 eV, EI): *m/z* (%): 249 (89) [M^+], 248 (100), 230 (5), 172 (9), 145 (34), 95 (7), 78 (7). These data are in agreement with those reported previously in the literature.[9]

Methyl 4-((2-methylbenzylidene)amino)benzoate (S3)

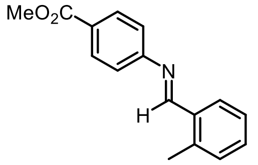

Synthesized according to general procedure GP-12 from methyl 4-aminobenzoate (151 mg, 1.00 mmol, 1.00 equiv) and 2-methylbenzaldehyde (120 mg, 1.00 mmol, 1.00 equiv). The title compound **S3** was obtained as a yellow solid (169 mg, 0.667 mmol, 67 %). R_f = 0.30 (pentane/EtOAc 19:1 *v/v*).

^{1}H NMR (600 MHz, $CDCl_3$) δ 8.72 (s, 1H), 8.11 – 8.04 (m, 3H), 7.39 (td, *J* = 7.5, 1.5 Hz, 1H), 7.32 (td, *J* = 7.5, 1.3 Hz, 1H), 7.25 (dd, *J* = 7.6, 1.1 Hz, 1H), 7.21 – 7.18 (m, 2H), 3.93 (s, 3H), 2.60 (s, 3H). **^{13}C NMR** (151 MHz, $CDCl_3$) δ 167.0, 160.5, 156.9, 139.1, 133.8, 131.6, 131.3, 131.0, 128.3, 127.4, 126.6, 120.8, 52.2, 19.6. **MS** (70 eV, EI): *m/z* (%): 253 (99.9) [M^+], 236 (100.0), 222 (26), 205 (27), 194 (98), 178 (22), 165 (18), 152 (9), 118 (30), 111 (11), 97 (18), 91 (14), 77 (12). These data are in agreement with those reported previously in the literature.[10]

Methyl 4-((2-bromo-5-chlorobenzylidene)amino)thiophene-2-carboxylate (S4)

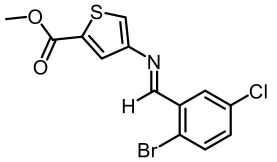

Synthesized according to general procedure GP-11 from methyl 4-aminothiophene-2-carboxylate (157 mg, 1.00 mmol, 1.00 equiv) and 2-bromo-5-chloro-benzaldehyde (220 mg, 1.00 mmol, 1.00 equiv). The title compound **S4** was obtained as a yellow solid (240 mg, 0.669 mmol, 67 %). R_f = 0.35 (pentane/EtOAc 19:1 *v/v*). **M.p.**: 120 – 122 °C.

^{1}H NMR (400 MHz, $CDCl_3$) δ 8.86 (s, 1H), 8.18 (d, *J* = 2.7 Hz, 1H), 7.91 (d, *J* = 1.7 Hz, 1H), 7.54 (d, *J* = 8.5 Hz, 1H), 7.50 (d, *J* = 1.6 Hz, 1H), 7.29 (dd, *J* = 8.6, 2.6 Hz, 1H), 3.92 (s, 3H). **^{13}C NMR** (151 MHz, $CDCl_3$) δ 162.5, 157.8, 151.1, 135.7, 134.5, 134.4, 133.9, 132.5, 128.7, 126.2, 124.0, 123.8, 52.5. **MS** (70 eV, EI): *m/z* (%): 359 (67) [M^+], 357 (50) [M^+], 328 (7), 278 (17), 246 (33), 234 (100), 219 (68), 199 (40), 184 (8), 168 (32), 140 (9), 123 (14), 113 (19), 82 (17), 75 (8), 59 (37). **HRMS** (ESI): *m/z* $[M + H]^+$ calculated for

$C_{13}H_{10}BrClNO_2S$ 357.9299, found: 357.9305. **IR** (neat, cm^{-1}): 3357, 3105, 2954, 2095, 1710, 1440, 1257, 1203, 1070, 1027, 886, 808, 775, 751.

N-(4-Methoxyphenyl)-2-methylbutan-1-imine (S5)

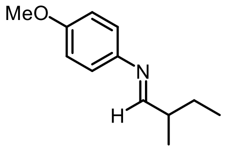

Synthesized according to general procedure GP-12 from 4-methoxyaniline (123 mg, 1.00 mmol, 1.00 equiv) and 2-methylbutanal (103 mg, 1.20 mmol, 1.20 equiv). The reaction mixture was stirred at room temperature for 30 min. The title compound **S5** was obtained as a dark red oil (189 mg, 0.989 mmol, 99 %). ***R_f*** = 0.38 (pentane/EtOAc 19:1 *v/v*).

^{1}H NMR (600 MHz, $CDCl_3$) δ 7.68 (d, *J* = 5.9 Hz, 1H), 7.04 – 7.00 (m, 2H), 6.88 – 6.84 (m, 2H), 3.79 (s, 3H), 2.42 (hept, *J* = 6.8 Hz, 1H), 1.68 – 1.61 (m, 1H), 1.54 – 1.46 (m, 1H), 1.16 (d, *J* = 6.8 Hz, 3H), 0.97 (t, *J* = 7.4 Hz, 3H). **^{13}C NMR** (151 MHz, $CDCl_3$) δ 169.2, 157.8, 145.5, 121.9, 114.3, 55.6, 41.7, 27.2, 17.0, 11.4. **MS** (70 eV, EI): *m/z* (%): 191 (47) [M$^+$], 176 (42), 163 (47), 149 (59), 134 (100), 122 (5), 107 (27), 92 (16), 77 (23), 64 (8). These data are in agreement with those reported previously in the literature.[6]

N-(9-Ethyl-9*H*-carbazol-3-yl)-2,2-dimethylpent-4-en-1-imine (S6)

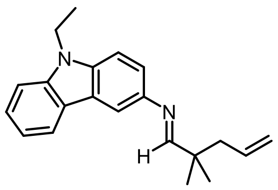

Synthesized according to general procedure GP-11 from 9-ethyl-9*H*-carbazol-3-amine (210 mg, 1.00 mmol, 1.00 equiv) and 2,2-dimethylpent-4-enal (168 mg, 1.05 mmol, 1.05 equiv). The title compound **S6** was obtained as a brown oil (327 mg, 1.07 mmol, quant. yield). ***R_f*** = 0.38 (pentane/EtOAc 19:1 *v/v*).

^{1}H NMR (600 MHz, $CDCl_3$) δ 8.14 (dt, *J* = 7.7, 1.0 Hz, 1H), 7.93 (s, 1H), 7.83 (d, *J* = 2.0 Hz, 1H), 7.52 (ddd, *J* = 8.2, 7.1, 1.2 Hz, 1H), 7.45 (dt, *J* = 8.2, 0.9 Hz, 1H), 7.40 (d, *J* = 8.5 Hz, 1H), 7.31 – 7.26 (m, 2H), 5.96 (ddt, *J* = 16.6, 10.4, 7.4 Hz, 1H), 5.21 – 5.15 (m, 2H), 4.41 (q, *J* = 7.2 Hz, 2H), 2.41 (dt, *J* = 7.4, 1.2 Hz, 2H), 1.48 (t, *J* = 7.2 Hz, 3H), 1.30 (s, 6H). **^{13}C NMR** (151 MHz, $CDCl_3$) δ 171.0, 144.7, 140.6, 138.4, 134.8, 125.9, 123.5, 123.2, 120.7, 119.7, 118.8, 117.8, 112.1, 108.7, 108.6, 45.0, 39.8, 37.8, 24.8, 14.0. **MS** (70 eV, EI): *m/z* (%): 304 (100) [M$^+$], 289 (25), 263 (85), 247 (33), 221 (29), 209 (35), 179 (30) 152 (9). **HRMS** (EI): *m/z* [M]$^+$ calculated for $C_{21}H_{24}N_2$ 304.1934, found: 304.1930. **IR** (neat, cm^{-1}): 3065, 2969, 2928, 1645, 1470, 1381, 1332, 1282, 1230, 1148, 1084, 996, 914, 876, 806, 744.

N-(Furan-2-ylmethyl)-1-phenylmethanimine (S7)

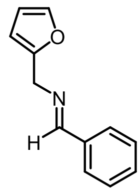

Synthesized according to general procedure GP-12 from furan-2-ylmethanamine (97.1 mg, 1.00 mmol, 1.00 equiv) and benzaldehyde (106 mg, 1.00 mmol, 1.00 equiv). The reaction mixture was stirred at room temperature for 30 min. The title compound **S7** was obtained as a yellow oil (173 mg, 0.936 mmol, 94 %). ***R_f*** = 0.31 (pentane/EtOAc 19:1 *v/v*).

^{1}H NMR (600 MHz, $CDCl_3$) δ 8.35 (s, 1H), 7.79 – 7.74 (m, 2H), 7.46 – 7.36 (m, 4H), 6.35 (dd, *J* = 3.2, 1.9 Hz, 1H), 6.28 (d, *J* = 3.2 Hz, 1H), 4.79 (s, 2H). **^{13}C NMR** (151 MHz, $CDCl_3$) δ 163.2, 152.5, 142.3, 136.1, 131.0, 128.7, 128.5, 110.5, 107.6, 57.4. **MS** (70 eV, EI): *m/z* (%): 185 (74) [M$^+$], 117 (5), 81 (100), 53 (12). **HRMS** (EI): *m/z* [M]$^+$ calculated for $C_{12}H_{11}NO$ 185.0835, found: 185.0833. **IR** (neat, cm^{-1}): 3060, 3026, 2874, 2333, 2097, 1908, 1642, 1502, 1448, 1376, 1329, 1218, 1180, 1146, 1075, 1009, 917, 883, 855, 808, 735, 693. These data are in agreement with those reported previously in the literature.[11]

N-Allyl-1-phenylmethanimine (*S8*)

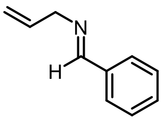

Synthesized according to general procedure GP-11 from prop-2-en-1-amine (57.1 mg, 1.00 mmol, 1.00 equiv) and benzaldehyde (106 mg, 1.00 mmol, 1.00 equiv). The title compound **S8** was obtained as a light-yellow oil (139 mg, 0.954 mmol, 95 %). $\boldsymbol{R_f}$ = 0.40 (pentane/EtOAc 19:1 *v/v*).

^{1}H NMR (600 MHz, $CDCl_3$) δ 8.30 (t, *J* = 1.4 Hz, 1H), 7.79 – 7.73 (m, 2H), 7.46 – 7.38 (m, 3H), 6.08 (ddt, *J* = 17.2, 10.3, 5.7 Hz, 1H), 5.24 (dq, *J* = 17.2, 1.8 Hz, 1H), 5.16 (dq, *J* = 10.3, 1.5 Hz, 1H), 4.27 (dq, *J* = 5.7, 1.5 Hz, 2H). **^{13}C NMR** (151 MHz, $CDCl_3$) δ 162.2, 136.3, 136.0, 130.8, 128.7, 128.3, 116.2, 63.6. **MS** (70 eV, EI): *m/z* (%): 145 (21) [M$^+$], 144 (100), 117 (26), 104 (22), 91 (20), 77 (11), 51 (6). These data are in agreement with those reported previously in the literature.[9]

1-(4-Chlorophenyl)-N-cyclohexylmethanimine (*S9*)

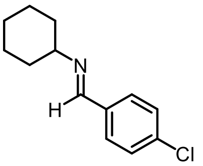

Synthesized according to general procedure GP-11 from cyclohexanamine (99.2 mg, 1.00 mmol, 1.00 equiv) and 4-chlorobenzaldehyde (141 mg, 1.00 mmol, 1.00 equiv). The title compound **S9** was obtained as a light red oil (212 mg, 0.957 mmol, 96 %). $\boldsymbol{R_f}$ = 0.38 (pentane/EtOAc 19:1 *v/v*).

^{1}H NMR (600 MHz, $CDCl_3$) δ 8.27 (s, 1H), 7.69 – 7.62 (m, 2H), 7.39 – 7.34 (m, 2H), 3.19 (tt, *J* = 10.6, 4.1 Hz, 1H), 1.83 (dp, *J* = 11.1, 3.7 Hz, 2H), 1.76 – 1.64 (m, 3H), 1.62 – 1.53 (m, 2H), 1.36 (qt, *J* = 12.6, 3.4 Hz, 2H), 1.26 (qt, *J* = 12.6, 3.5 Hz, 1H). **^{13}C NMR** (151 MHz, $CDCl_3$) δ 157.3, 136.3, 135.3, 129.4, 128.9, 70.1, 34.5, 25.8, 24.9. **MS** (70 eV, EI): *m/z* (%): 221 (95) [M$^+$], 192 (100), 178 (94), 166 (59), 138 (88), 125 (26), 109 (15), 89 (29), 83 (14), 55 (16). **HRMS** (ESI): *m/z* [M + H]$^+$ calculated for $C_{13}H_{17}ClN$ 222.1044, found: 222.1045. **IR** (neat, cm^{-1}): 2927, 2853, 2105, 1704, 1638, 1590, 1486, 1452, 1381, 1346, 1293, 1076, 1008, 966, 887, 823.

N-(2-Bromophenethyl)-2,2-dimethylpropan-1-imine (S10)

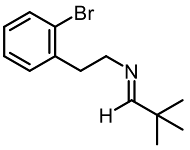

Synthesized according to general procedure GP-11 from 2-(2-bromophenyl)ethan-1-amine (200 mg, 1.00 mmol, 1.00 equiv) and pivalaldehyde (172 mg, 217 µL, 2.00 mmol, 2.00 equiv). The title compound **S10** was obtained as a light-yellow oil (250 mg, 0.933 mmol, 93 %). $\boldsymbol{R_f}$ = 0.31 (pentane/EtOAc 19:1 *v/v*).

^{1}H NMR (600 MHz, $CDCl_3$) δ 7.51 (dd, *J* = 8.0, 1.2 Hz, 1H), 7.28 (t, *J* = 1.2 Hz, 1H), 7.18 (dtd, *J* = 14.5, 7.6, 1.7 Hz, 2H), 7.07 – 7.01 (m, 1H), 3.62 (td, *J* = 7.1, 1.2 Hz, 2H), 3.04 (t, *J* = 7.1 Hz, 2H), 0.98 (d, *J* = 0.8 Hz, 9H). **^{13}C NMR** (151 MHz, $CDCl_3$) δ 173.2, 139.3, 132.8, 131.9, 127.9, 127.2, 124.8, 60.6, 37.5, 36.1, 26.9. **MS** (70 eV, EI): *m/z* (%): 269 (0.05) [M$^+$], 267 (0.1) [M$^+$], 188 (49), 183 (35), 104 (18), 98 (100), 77 (12), 69 (14), 56 (6). **HRMS** (ESI): *m/z* [M + H]$^+$ calculated for $C_{13}H_{19}BrN$ 268.0695, found: 268.0698. **IR** (neat, cm^{-1}): 3061, 2957, 2863, 2830, 2096, 1666, 1469, 1441, 1363, 1211, 1027, 918, 748.

8.5. Characterization data of TMS-protected alcohol

1,2:3,4-Bis-*O*-(1-methylethylidene)-6-*O*-(trimethylsilyl)-α-D-galactopyranose (S11)

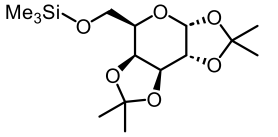

Synthesized according to general procedure GP-13 from 1,2:3,4-bis-*O*-(1-methylethylidene)-α-D-galactopyranose (260 mg, 1.00 mmol, 1.00 equiv). The title compound **S11** was obtained as a light-yellow oil (309 mg, 0.929 mmol, 93 %). $\boldsymbol{R_f}$ = 0.50 (pentane/EtOAc 19:1 *v/v*).

^{1}H NMR (600 MHz, $CDCl_3$) δ 5.51 (d, *J* = 5.0 Hz, 1H), 4.59 (dd, *J* = 8.0, 2.3 Hz, 1H), 4.31 – 4.27 (m, 2H), 3.82 (td, *J* = 6.7, 1.8 Hz, 1H), 3.77 (dd, *J* = 10.2, 7.2 Hz, 1H), 3.70 (dd, *J* = 10.2, 6.1 Hz, 1H), 1.52 (s, 3H), 1.44 (s, 3H), 1.34 (s, 3H), 1.32 (s, 3H), 0.12 (s, 9H). **^{13}C NMR** (151 MHz, $CDCl_3$) δ 109.2, 108.6, 96.5, 71.0, 70.74,

70.70, 68.3, 61.5, 26.2, 26.1, 25.1, 24.5, –0.3. **MS** (70 eV, EI): *m/z* (%): 317 (86), 259 (100), 241 (8), 214 (11), 201 (17), 199 (32), 186 (9), 171 (33), 155 (18), 143 (72), 129 (64), 117 (72), 103 (61), 85 (39), 81 (62), 73 (58), 59 (23), 55 (8). **HRMS** (ESI): *m/z* $[M + Na]^+$ calculated for $C_{15}H_{28}NNaO_6Si$ 355.1547, found: 355.1538. **IR** (neat, cm^{-1}): 2984, 2938, 2328, 2088, 1757, 1457, 1377, 1251, 1210, 1170, 1070, 1001, 841, 751, 684. These data are in agreement with those reported previously in the literature.[12]

9. Property investigation

9.1. Computed log*P* values

The octanol-water partition (log*P*) was calculated using COSMOtherm.[13] Input structures for COSMOtherm were generated from single-point energy calculations at the BP86/def2-TZVPD level of theory using Turbomole[14] based on the minimum energy conformer geometries obtained after geometry optimization and manual conformational search at DFT level using Gaussian 16, Revision A.03.[15] Geometry optimizations were conducted in acetonitrile (SMD solvation) at the M06-2X/6-311++G(d,p) level of theory. Frequencies were calculated at the same level of theory and used to verify the nature of all stationary points as minima (no imaginary frequencies). It was found that in all cases fluorinated motifs (-CH_2F, -CF_2H and -CF_3) preferred trans orientation in relation to carbonyl group (C=O).
Table S6 summarizes the computed log*P* values. The results indicate the increase of lipophilicity with the increase of the number of fluorine atoms (*N*-Me < *N*-CH_2F < *N*-CF_2H < *N*-CF_3).

Table S6. Computed log*P* values of **DMF** and its fluorinated analogues.

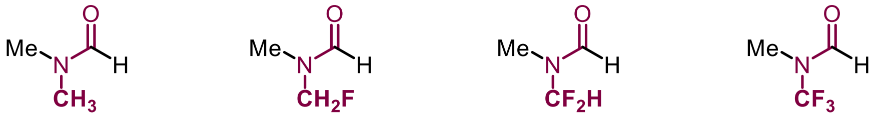

N-**Me** *N*-**CH_2F** *N*-**CF_2H** *N*-**CF_3**

entry	Compound	log*P* (wet octanol)	log*P* (dry octanol)
1	*N*-CF_3	0.82	0.70
2	*N*-CF_2H	0.27	0.05
**3**	***N*-CH_2F**	**-0.48**	**-0.71**
4	*N*-CH_3	-0.61	-0.85

9.1.1. XYZ coordinates of computed structure

***N*-CF_3 formamide**

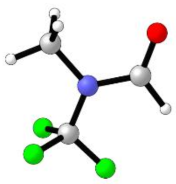

C	0.88233	-0.12741	-0.00275
C	-1.46999	-0.75916	-0.01575
O	-2.64139	-0.46163	0.01370
F	1.08996	-1.44018	-0.06105
N	-0.48104	0.19477	-0.05324
C	-0.84913	1.61351	-0.00532
H	0.04497	2.21916	-0.13814
H	-1.54957	1.82600	-0.81114
H	-1.30984	1.84657	0.95473
H	-1.11093	-1.79299	-0.02258
F	1.56184	0.43537	-1.01519
F	1.46425	0.32362	1.12325

Zero-point correction = 0.079852 (Hartree/Particle)
Thermal correction to Energy = 0.087758
Thermal correction to Enthalpy = 0.088702
Thermal correction to Gibbs Free Energy = 0.046192
Sum of electronic and zero-point Energies = -546.148324
Sum of electronic and thermal Energies = -546.140418
Sum of electronic and thermal Enthalpies = -546.139474
Sum of electronic and thermal Free Energies = -546.181984

_N_-CF$_2$H formamide

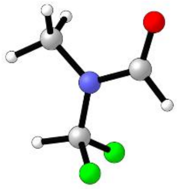

C	1.08260	0.33249	-0.08443
H	1.48096	1.32108	-0.29494
C	-0.98391	-0.87545	0.04088
O	-2.18982	-0.95986	0.13009
F	1.55263	-0.07963	1.13706
N	-0.32719	0.31254	-0.12727
C	-1.08600	1.56216	-0.12834
H	-0.43807	2.36813	-0.46751
H	-1.92677	1.46640	-0.81359
H	-1.46127	1.78929	0.87127
H	-0.32109	-1.75064	0.07807
F	1.60282	-0.56684	-0.96949

Zero-point correction = 0.088357 (Hartree/Particle)
Thermal correction to Energy = 0.095695
Thermal correction to Enthalpy = 0.096640
Thermal correction to Gibbs Free Energy = 0.055762
Sum of electronic and zero-point Energies = -446.889807
Sum of electronic and thermal Energies = -446.882469
Sum of electronic and thermal Enthalpies = -446.881525
Sum of electronic and thermal Free Energies = -446.922402

_N_-CH$_2$F formamide

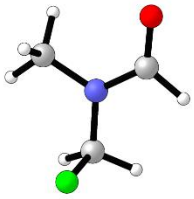

C	1.23947	-0.30167	0.55765
H	1.76178	0.39756	1.20768
H	1.22620	-1.30760	0.97283
C	-1.07449	-0.75910	0.09153
O	-2.19532	-0.46275	-0.27204
F	1.98572	-0.35909	-0.63731
N	-0.07624	0.14363	0.28903
C	-0.31190	1.54798	-0.03029
H	0.50271	2.13947	0.38516
H	-0.35456	1.70158	-1.11108
H	-1.25522	1.86855	0.41019
H	-0.77457	-1.79429	0.31088

Zero-point correction = 0.096416 (Hartree/Particle)
Thermal correction to Energy = 0.103050
Thermal correction to Enthalpy = 0.103994
Thermal correction to Gibbs Free Energy = 0.065502
Sum of electronic and zero-point Energies = -347.634963
Sum of electronic and thermal Energies = -347.628329
Sum of electronic and thermal Enthalpies = -347.627385
Sum of electronic and thermal Free Energies = -347.665877

DMF

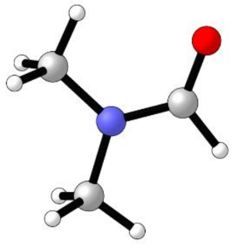

C	-1.57713	-0.77069	0.00003
H	-2.16473	-0.52419	-0.88830
H	-1.36409	-1.83952	-0.00007
C	0.86037	-0.63950	-0.00003
O	1.95147	-0.08343	0.00004
N	-0.33331	-0.02324	-0.00007
C	-0.44062	1.42382	-0.00000
H	-0.98417	1.75784	-0.88794
H	-0.98272	1.75777	0.88884
H	0.55698	1.85716	-0.00079
H	0.76893	-1.73629	-0.00010
H	-2.16450	-0.52436	0.88856

Zero-point correction = 0.103079 (Hartree/Particle)
Thermal correction to Energy = 0.109216
Thermal correction to Enthalpy = 0.110160
Thermal correction to Gibbs Free Energy = 0.073776
Sum of electronic and zero-point Energies = -248.379897
Sum of electronic and thermal Energies = -248.373761
Sum of electronic and thermal Enthalpies = -248.372817
Sum of electronic and thermal Free Energies = -248.409200

9.2. NMR conformational studies

The *N*-CH_2F formamides display distinct rotamers in ^1H NMR at room temperature. Hence, high temperature ^1H NMR experiment was performed on compound **34** in toluene-d_8 as solvent (Figure S5). Our analysis showed coalescence at 60 °C. The corresponding *N*-CF_3 formamide analogue of **34** showed no rotamers at ambient temperature (coalescence at 15 °C),[3] while its *N*-CF_2H analogue showed coalescence at 40 °C (Figure S6).[16]

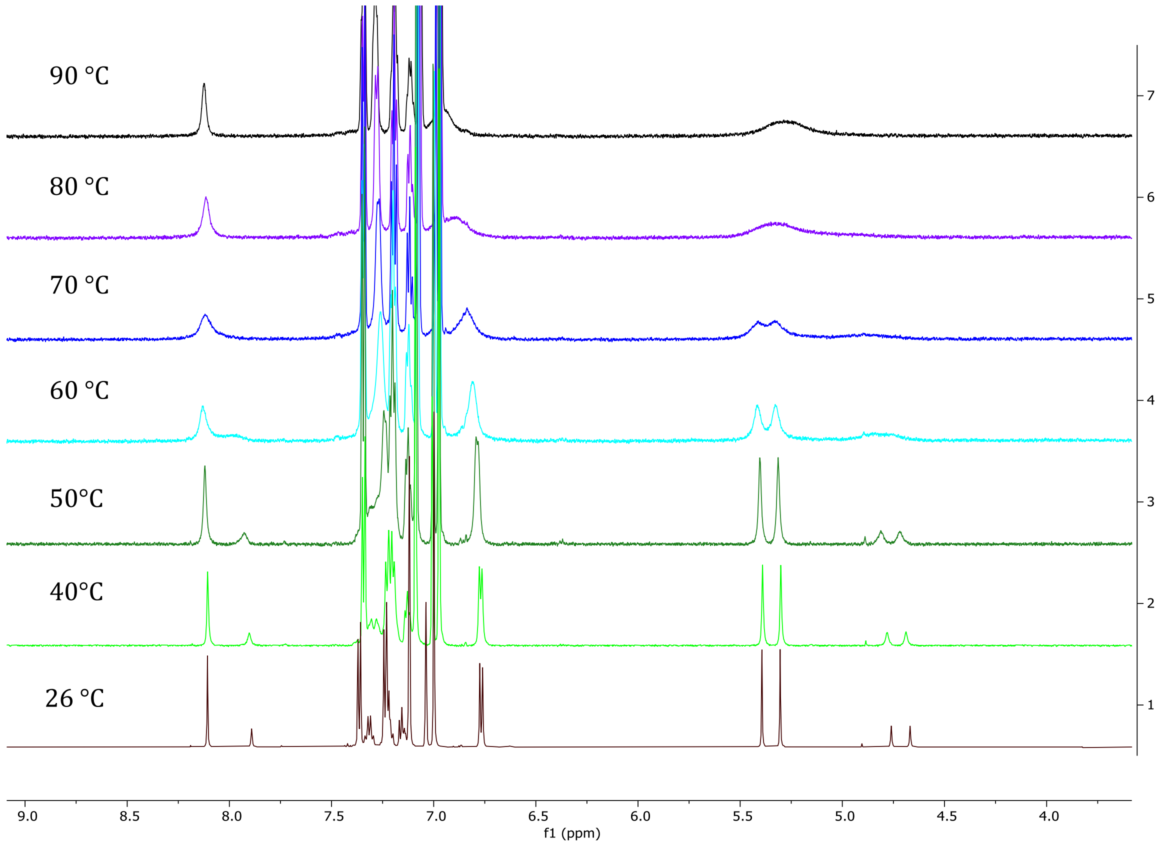

Figure S5. High temperature ^1H NMR study of *N*-CH_2F formamide **34** (^1H Coalescence temperature 60°C).

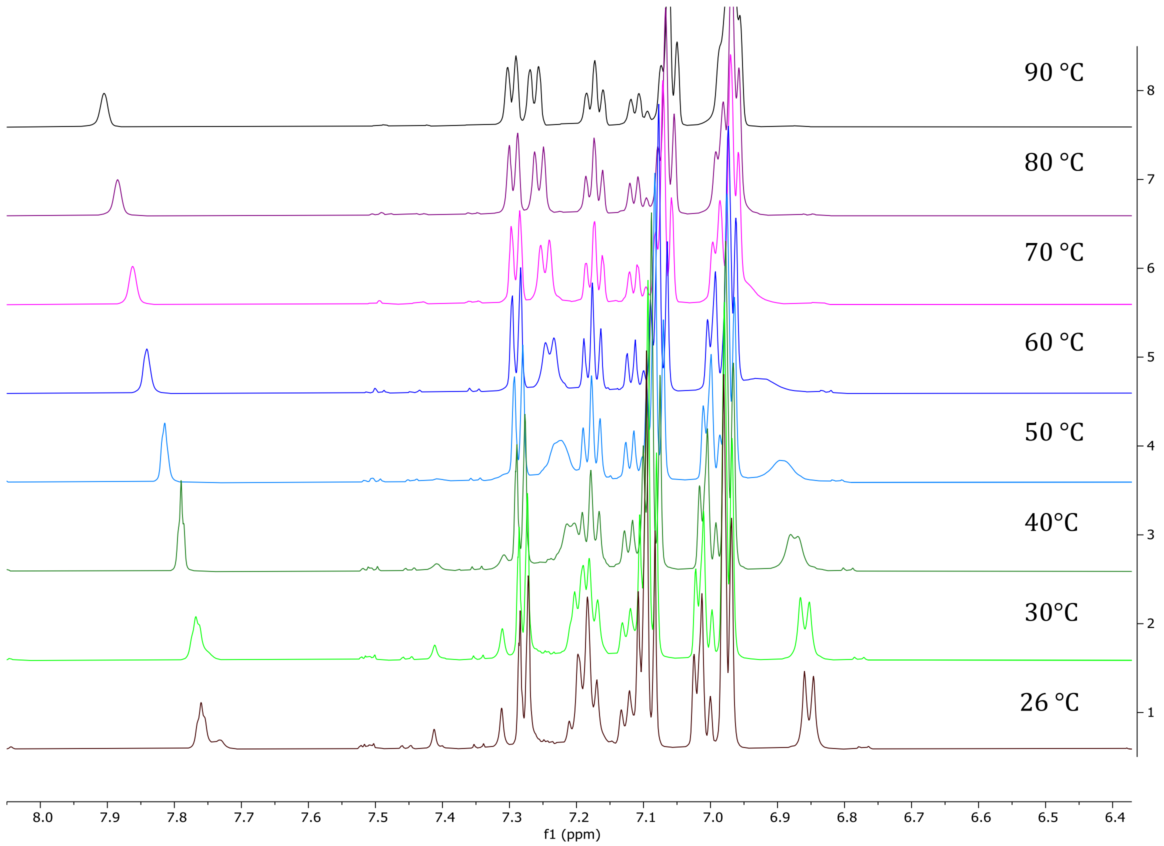

Figure S6. High temperature ^1H NMR study of *N*-CF_2H formamide analogue of **34** (^1H Coalescence temperature 40 °C)

10. NMR spectra

10.1. *N*-CH_2F and *N*-CHRF carbamoyl fluorides

[1,1'-Biphenyl]-4-yl(fluoromethyl)carbamic fluoride (1)

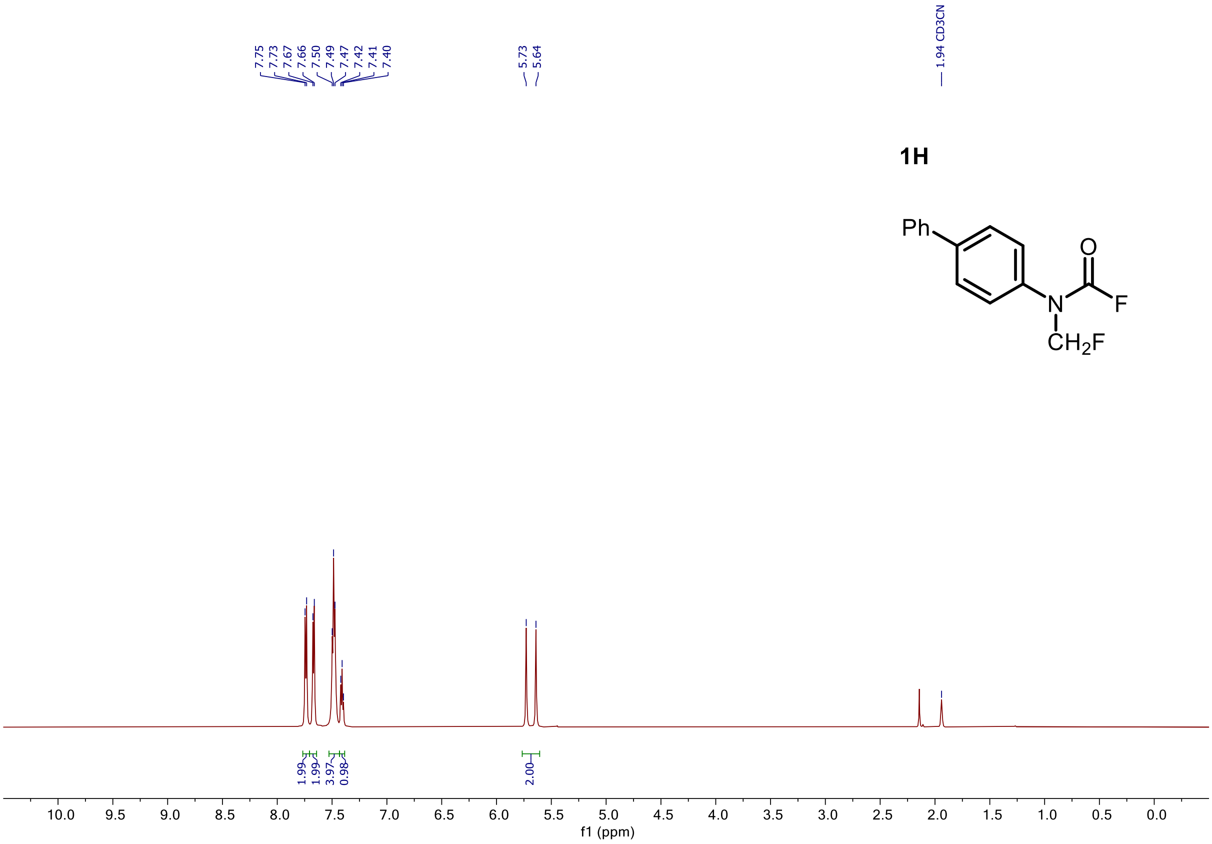

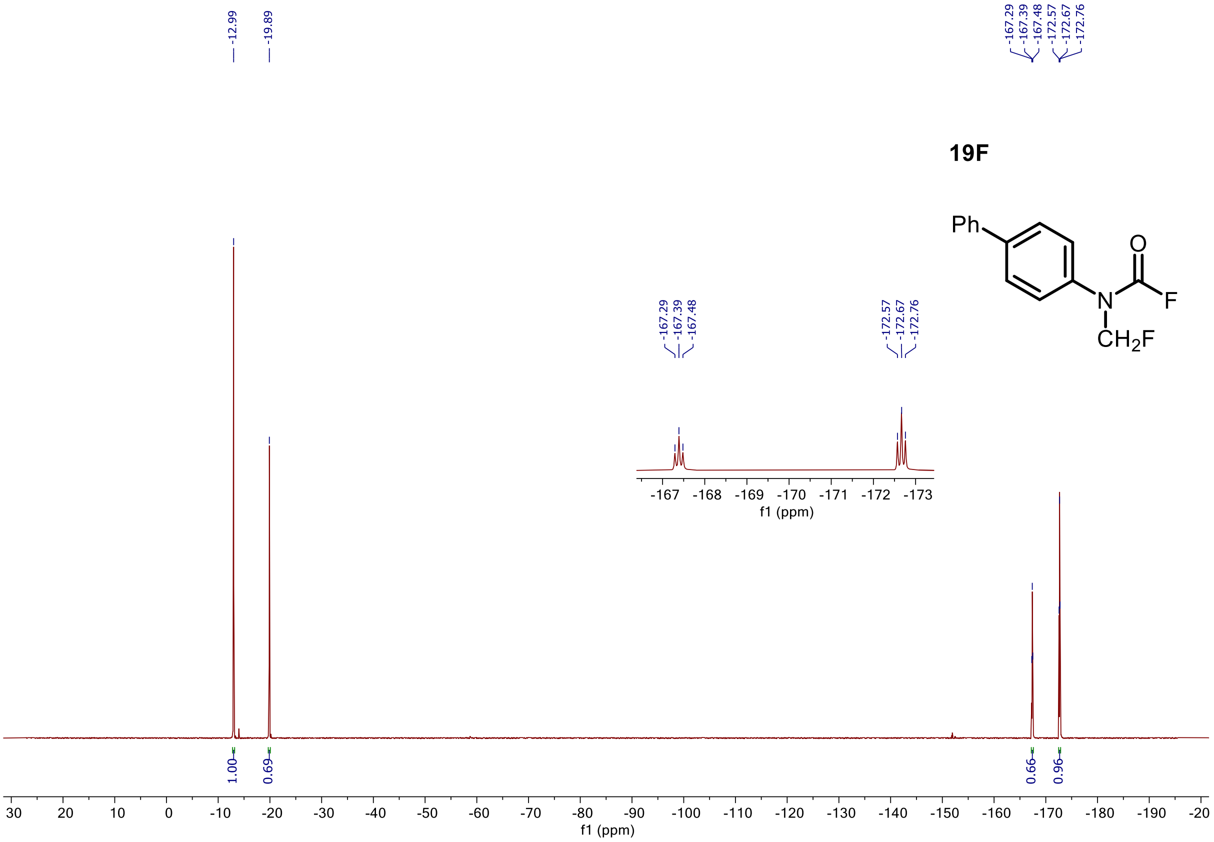

19F
Ph
O
F
N
CH2F
-12.99
-19.89
-167.29
-167.39
-167.48
-172.57
-172.67
-172.76
1.00
0.69
0.66
0.96
f1 (ppm)

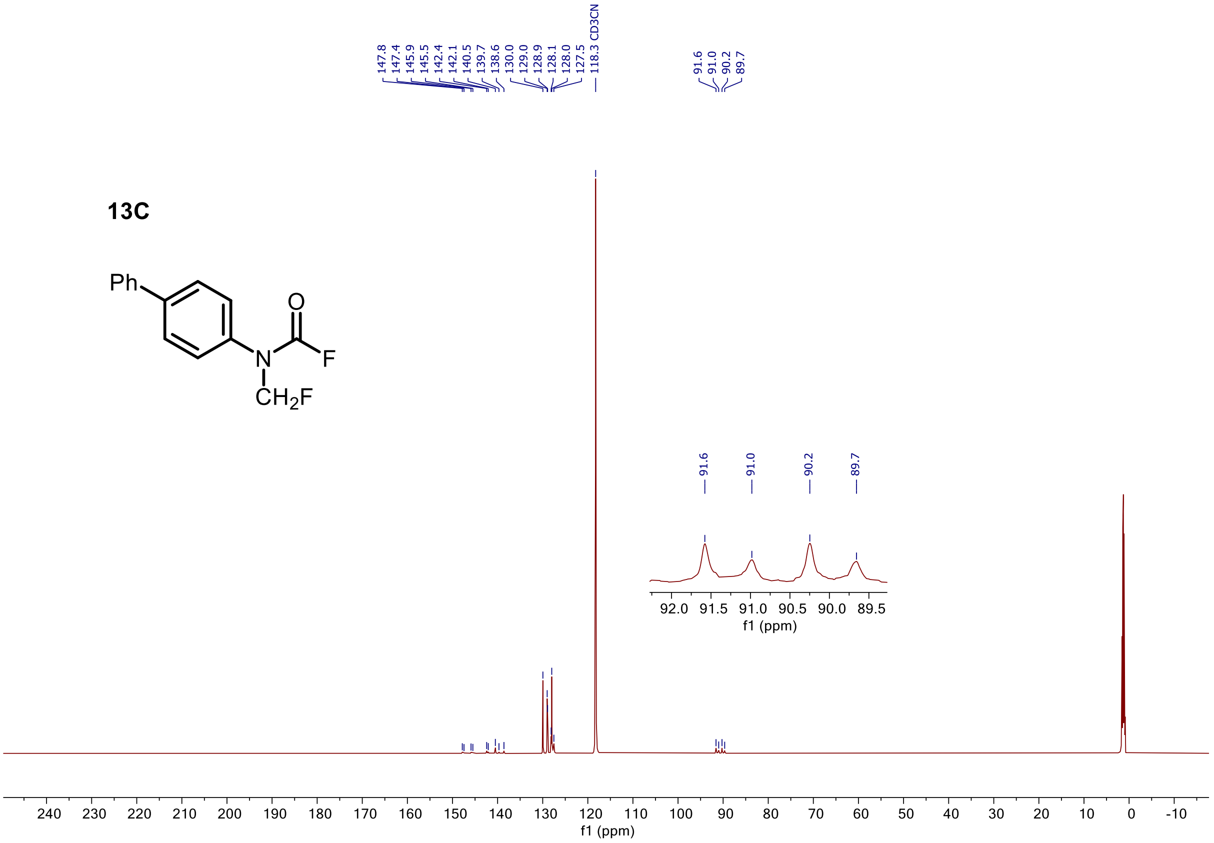

13C
Ph
O
F
N
CH2F
147.8
147.4
145.9
145.5
142.4
142.1
140.5
139.7
138.6
130.0
129.0
128.9
128.1
128.0
127.5
118.3 CD3CN
91.6
91.0
90.2
89.7
f1 (ppm)

(Fluoromethyl)(3,4,5-trimethoxyphenyl)carbamic fluoride (2)

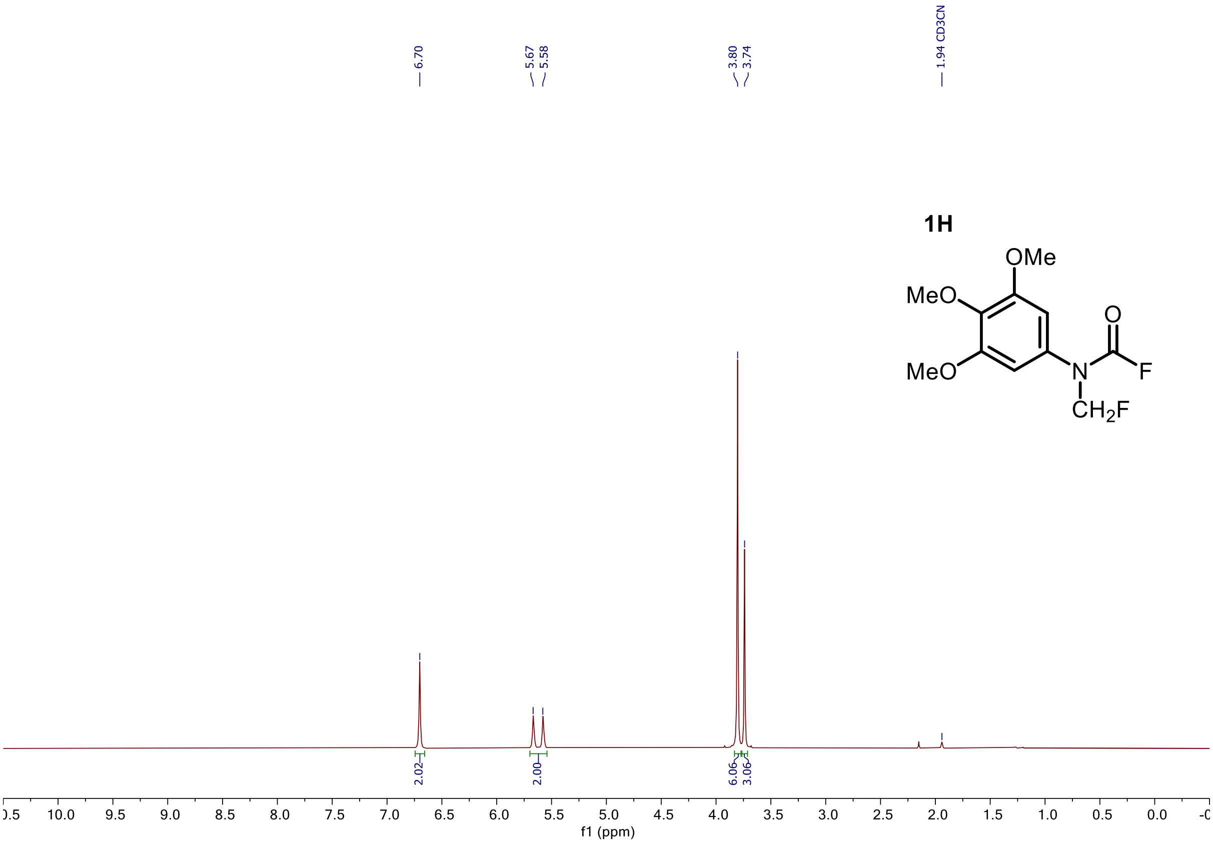

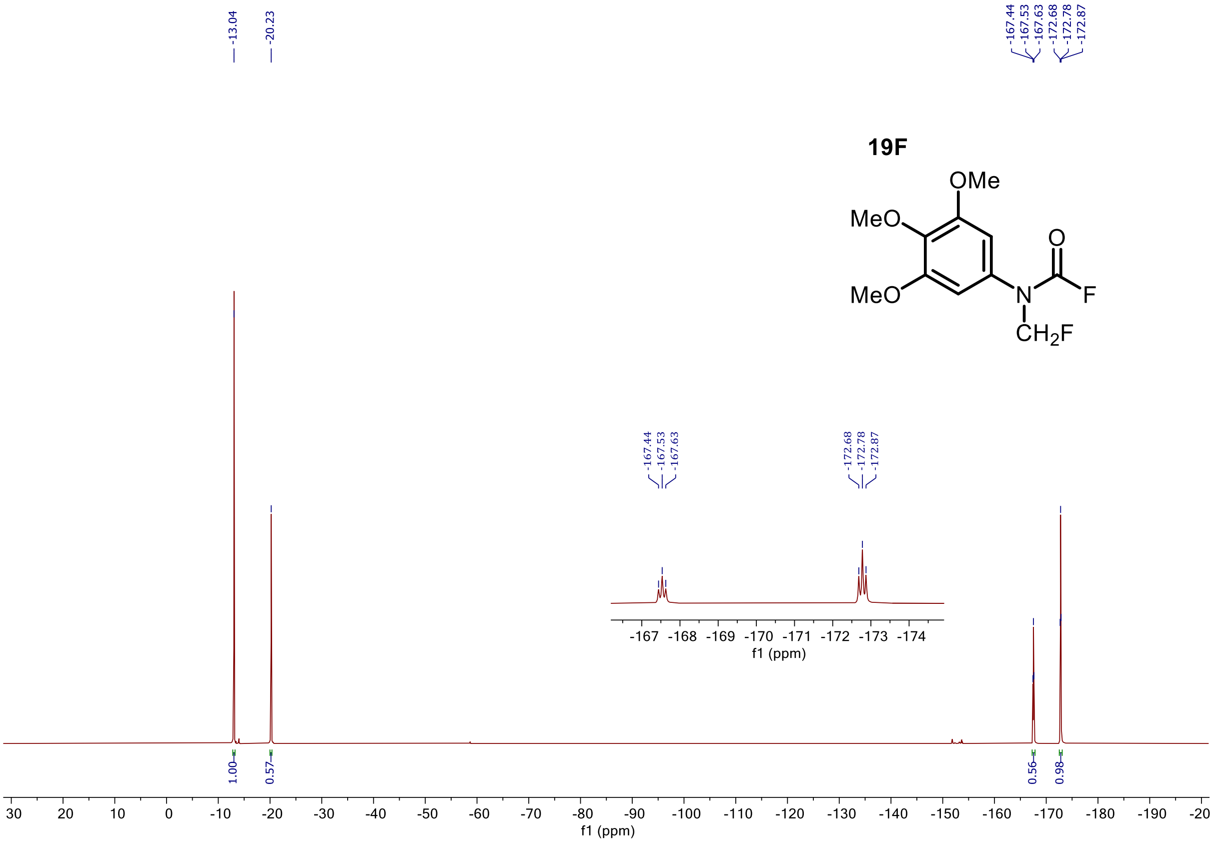

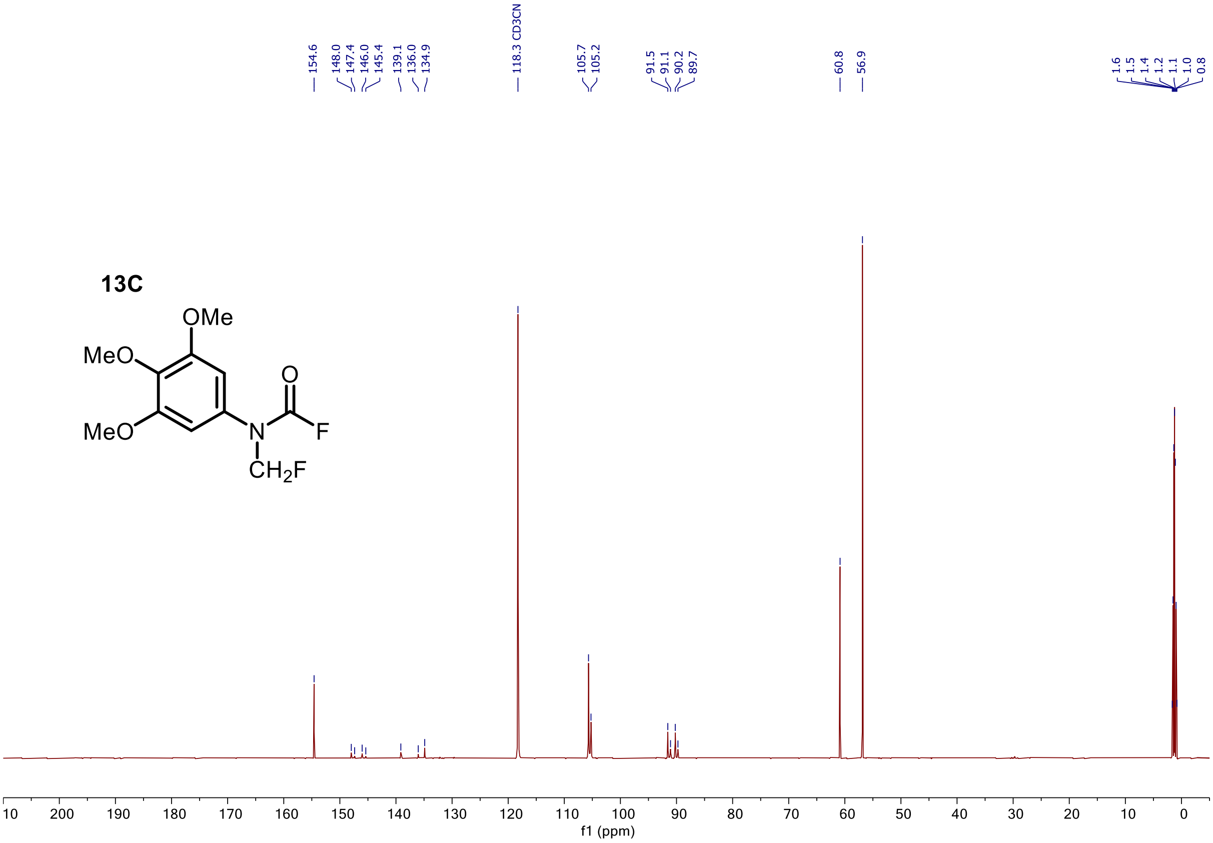

Methyl 4-((fluorocarbonyl)(fluoromethyl)amino)benzoate(3)

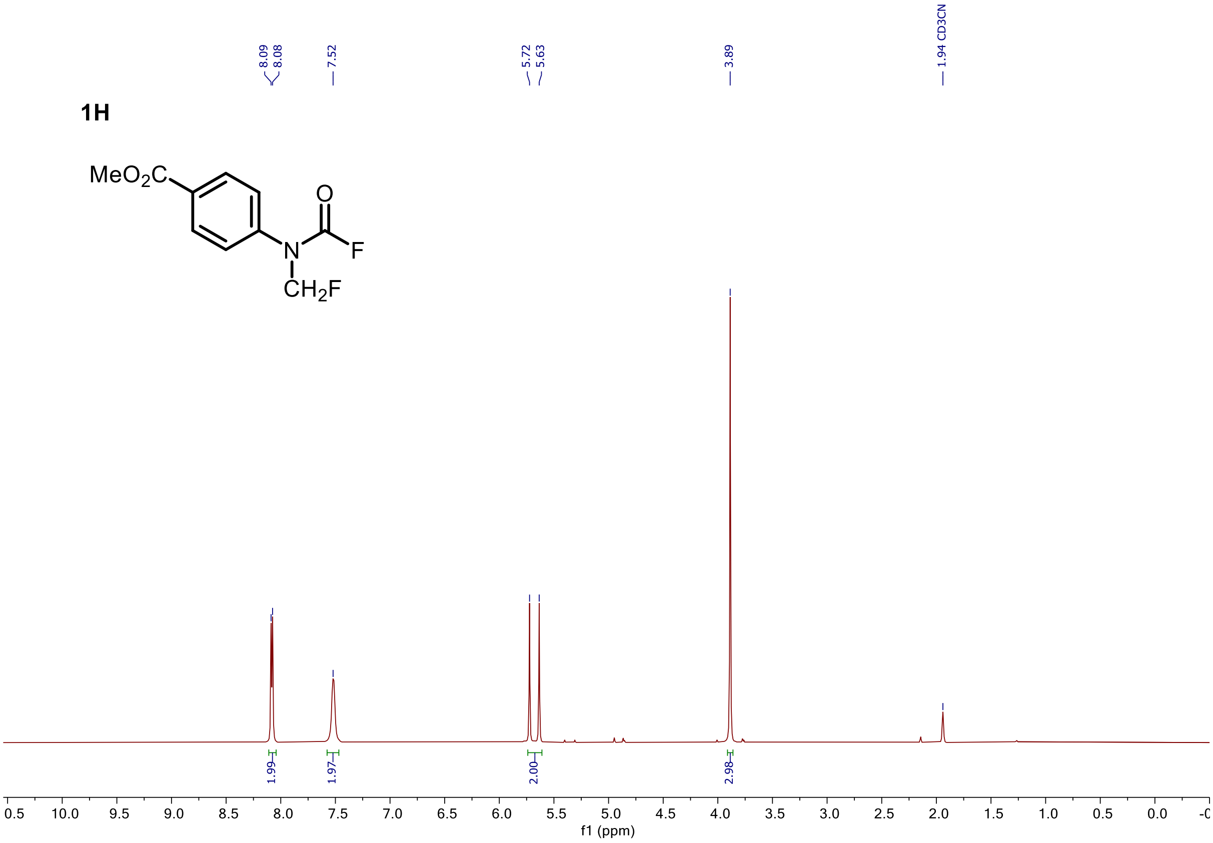

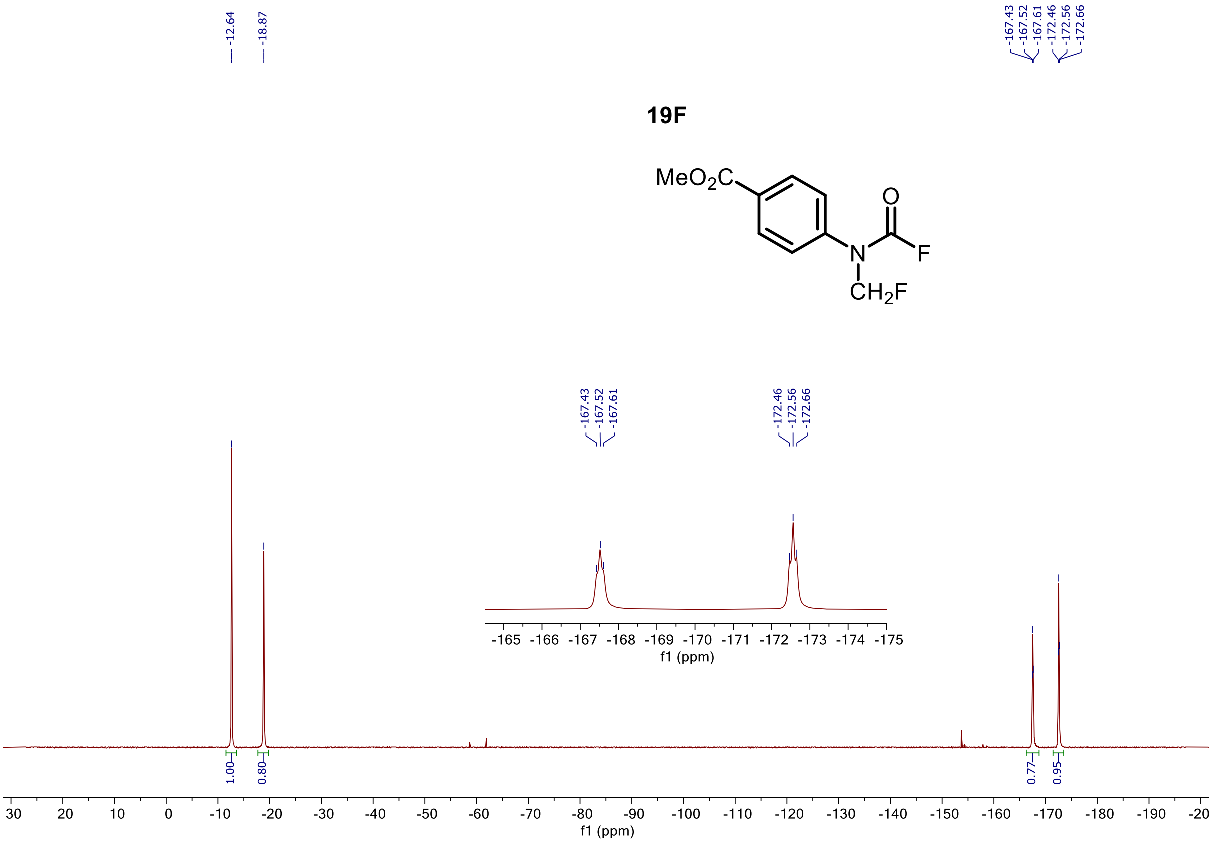
-12.64
-18.87
-167.43
-167.52
-167.61
-172.46
-172.56
-172.66
19F
MeO2C
O
F
N
CH2F
1.00
0.80
0.77
0.95
f1 (ppm)

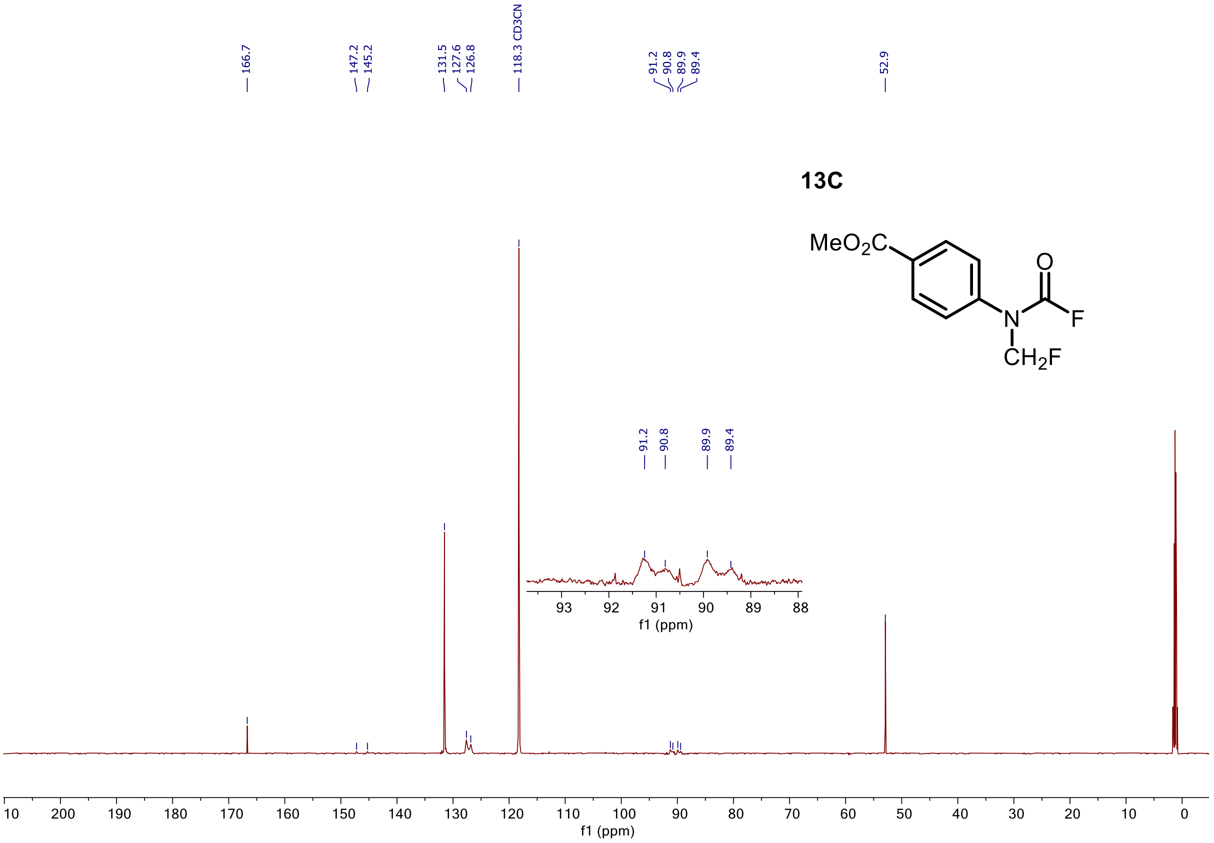
166.7
147.2
145.2
131.5
127.6
126.8
118.3 CD3CN
91.2
90.8
89.9
89.4
52.9
13C
MeO2C
O
F
N
CH2F
f1 (ppm)

(2-Fluoro-4-iodophenyl)(fluoromethyl)carbamic fluoride (4)

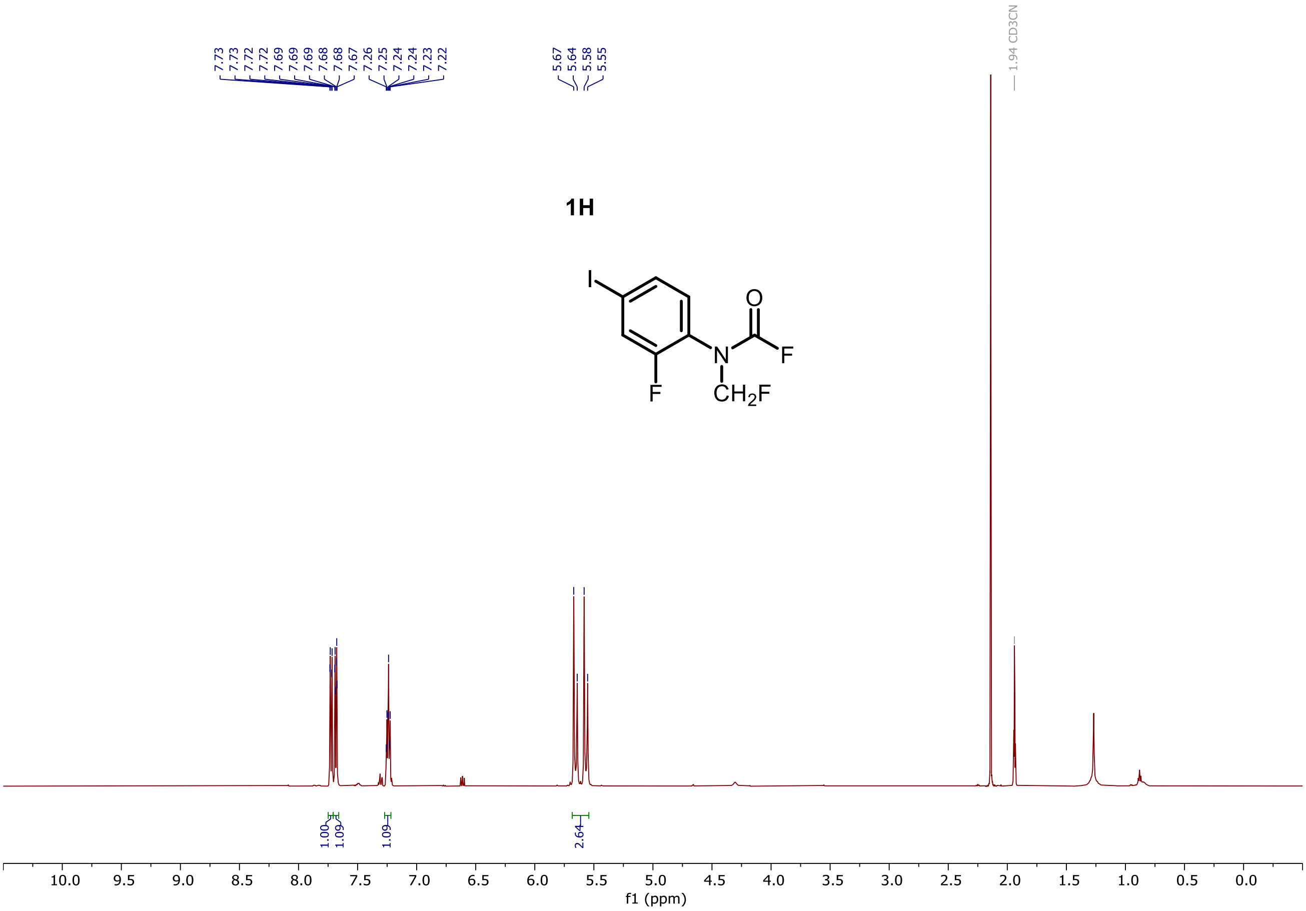

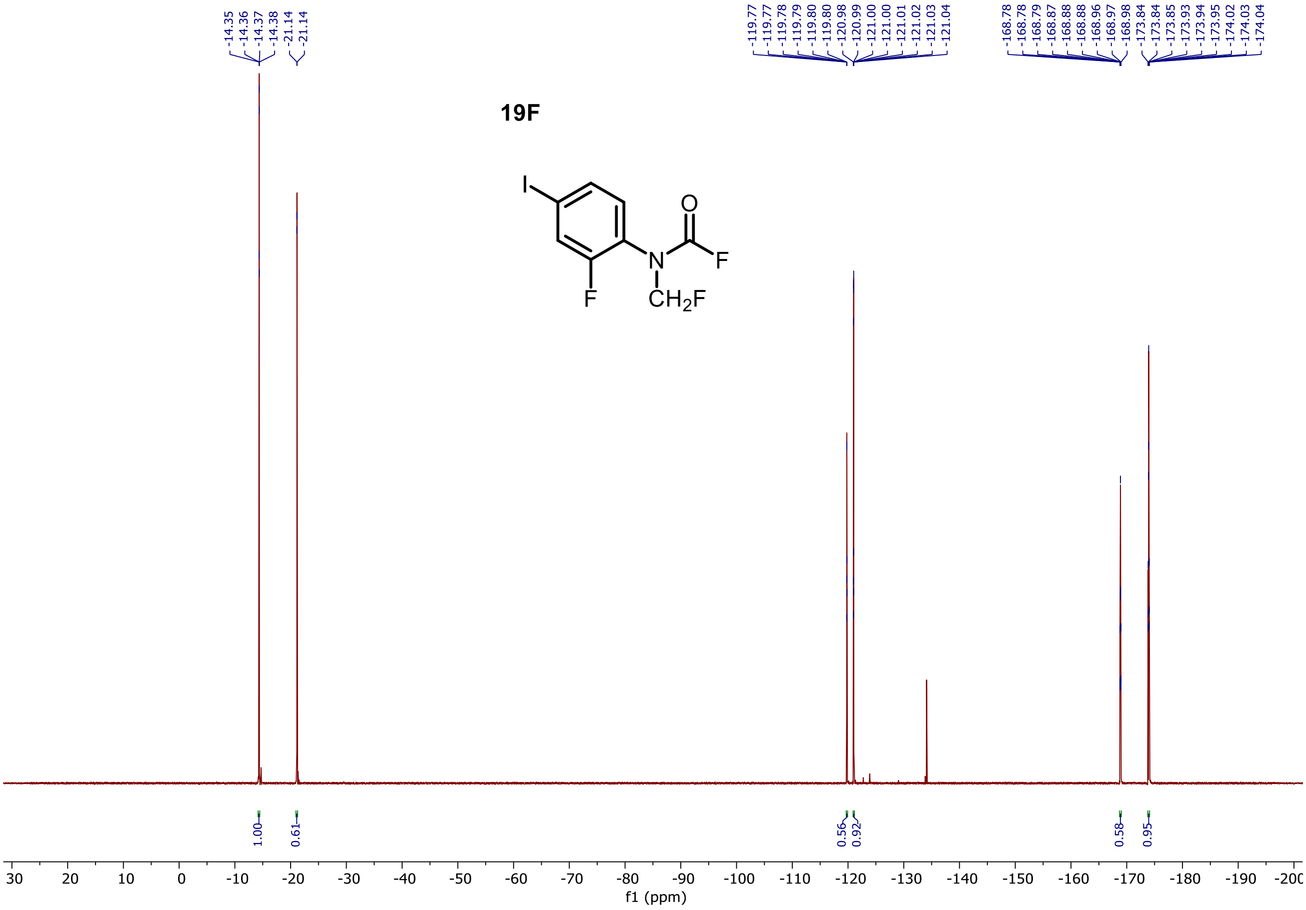

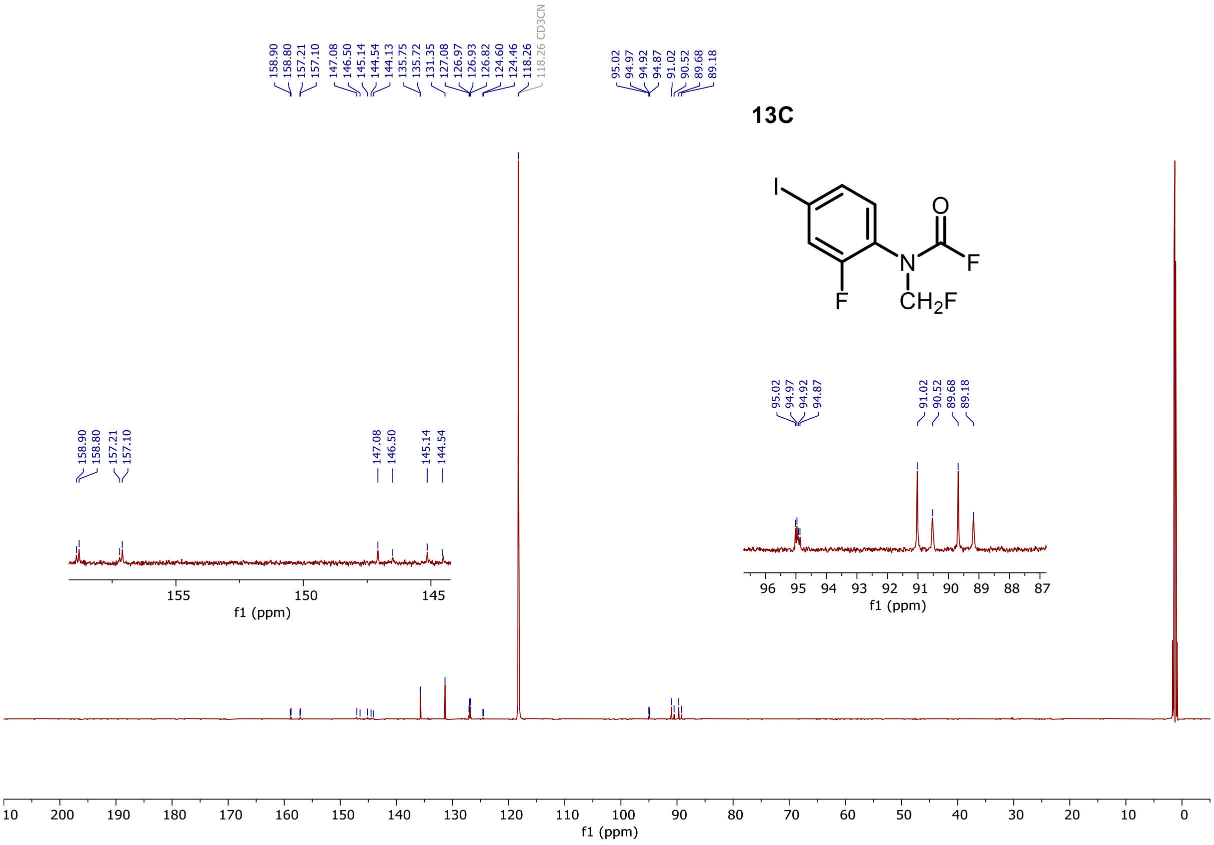

(4-Bromo-3-chlorophenyl)(fluoromethyl)carbamic fluoride (5)

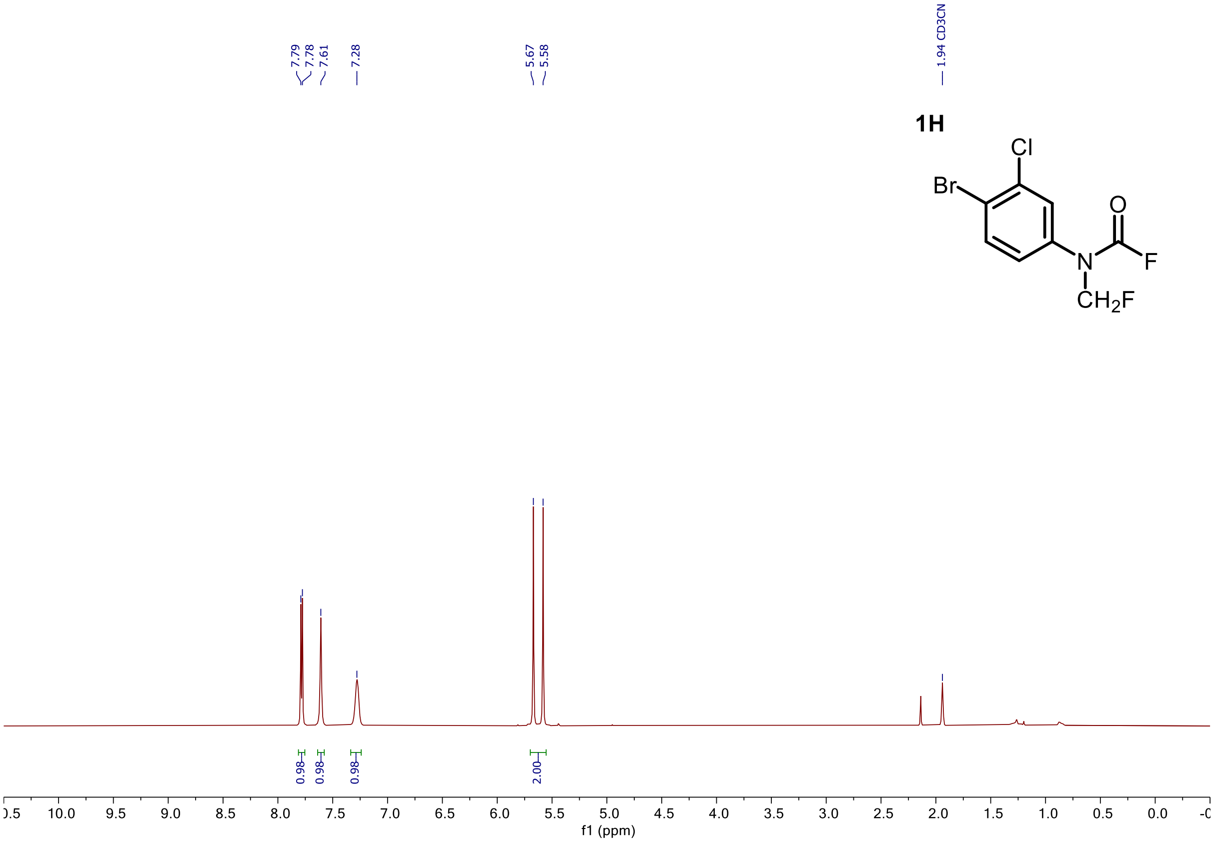

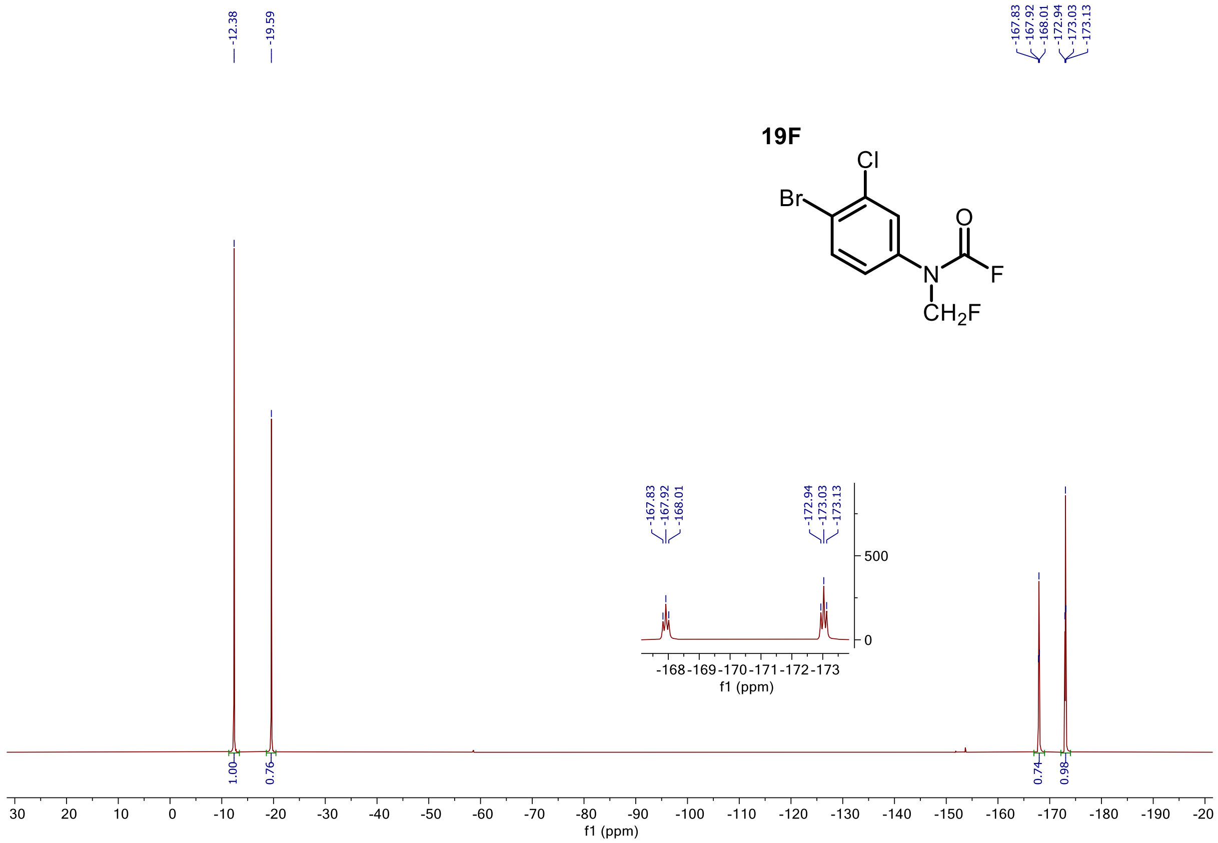

-12.38
-19.59
-167.83
-167.92
-168.01
-172.94
-173.03
-173.13
19F
Cl
Br
O
N
F
CH2F
500
0
-168 -169 -170 -171 -172 -173
f1 (ppm)
1.00
0.76
0.74
0.98
30 20 10 0 -10 -20 -30 -40 -50 -60 -70 -80 -90 -100 -110 -120 -130 -140 -150 -160 -170 -180 -190 -20
f1 (ppm)

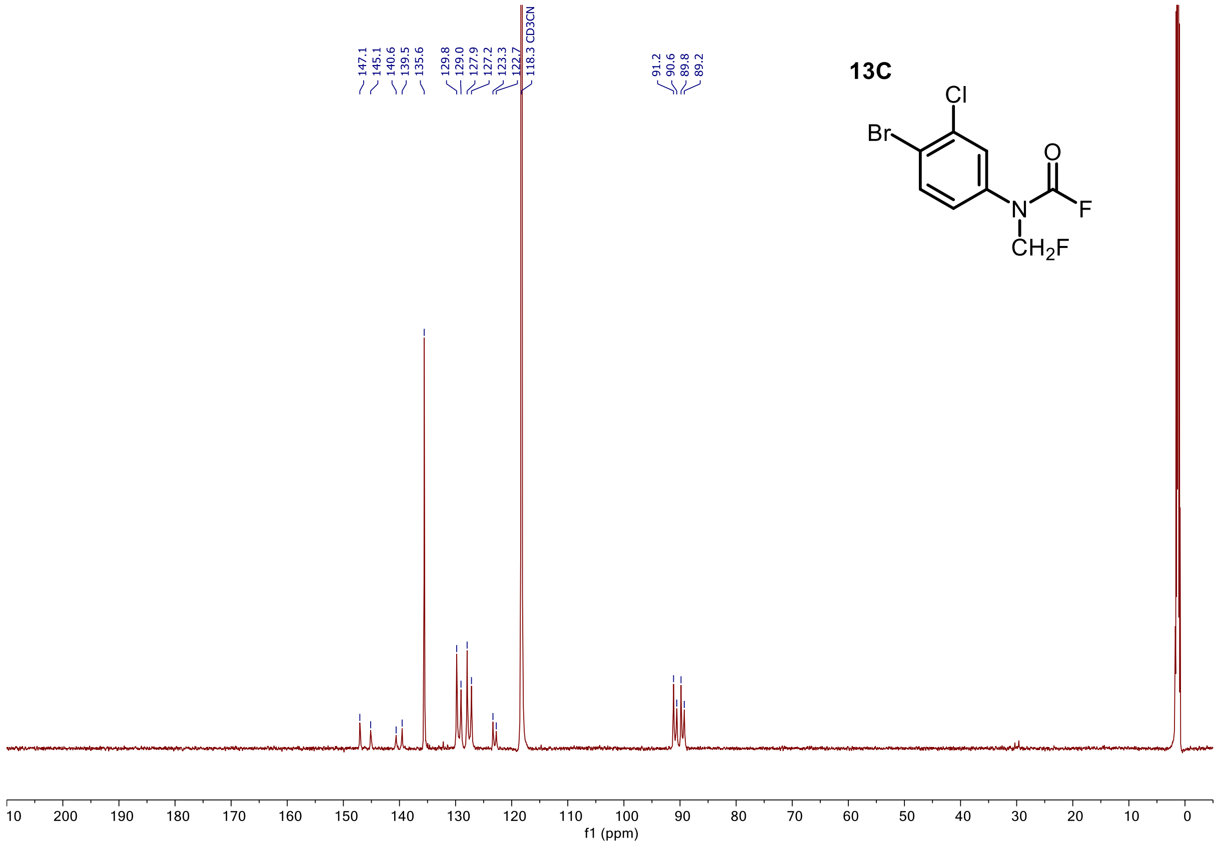

147.1
145.1
140.6
139.5
135.6
129.8
129.0
127.9
127.2
123.3
122.7
118.3 CD3CN
91.2
90.6
89.8
89.2
13C
Cl
Br
O
N
F
CH2F
10 200 190 180 170 160 150 140 130 120 110 100 90 80 70 60 50 40 30 20 10 0
f1 (ppm)

(Fluoromethyl)(2-isopropylphenyl)carbamic fluoride (6)

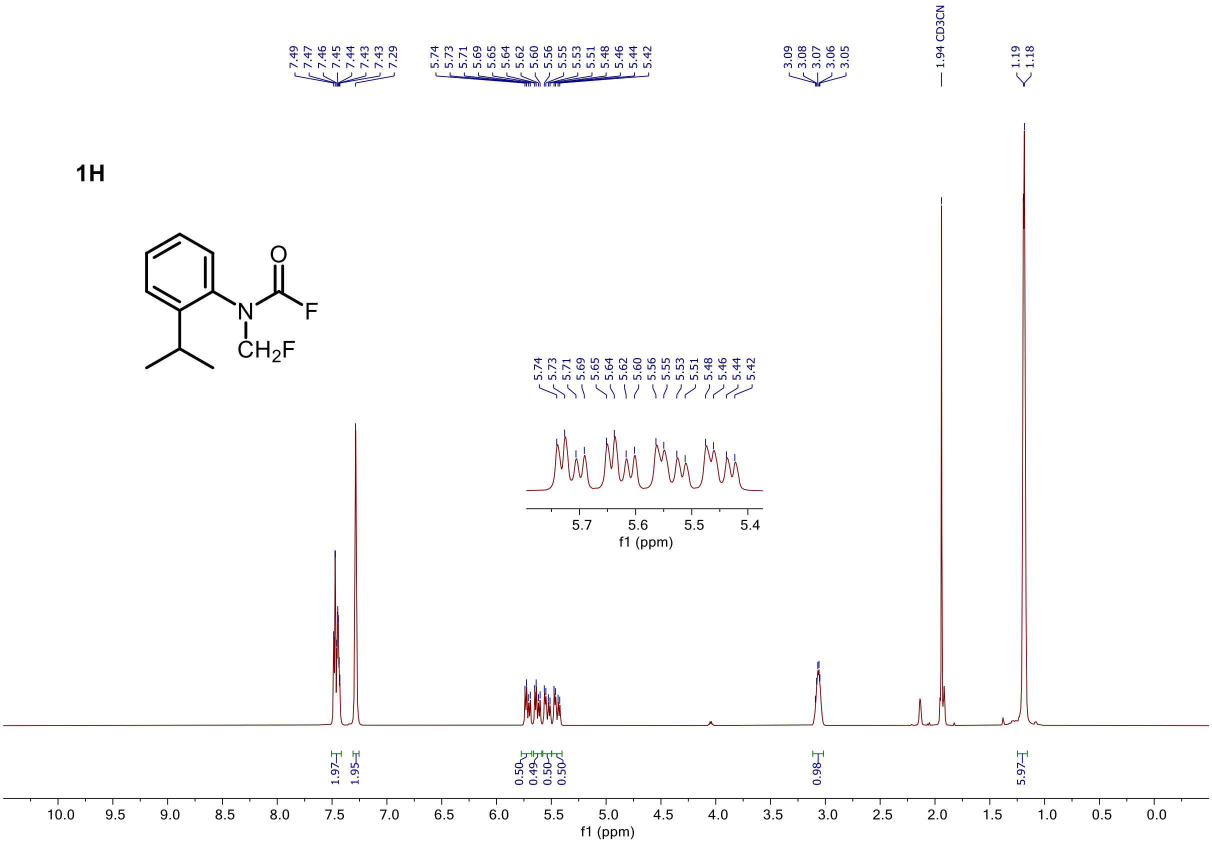

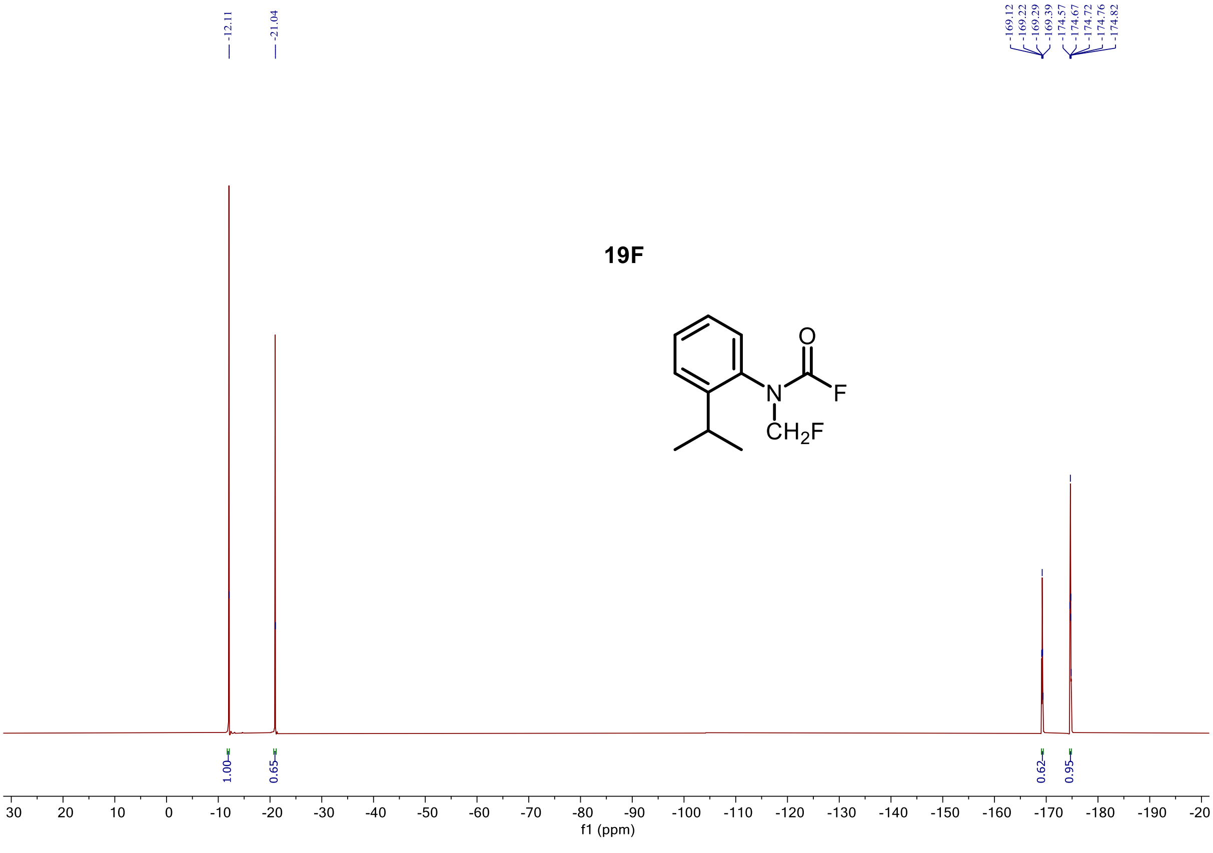

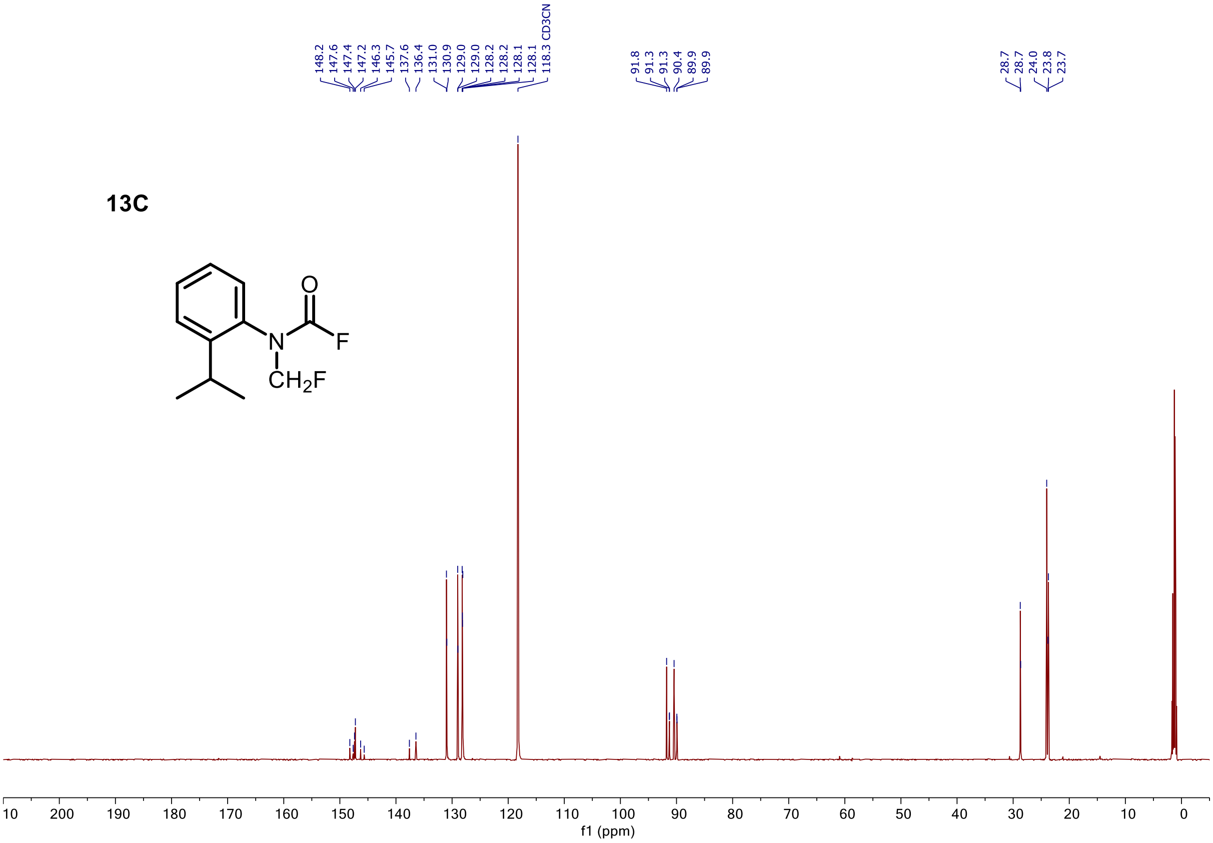

(Fluoromethyl)(2-methoxy-5-methylphenyl)carbamic fluoride (7)

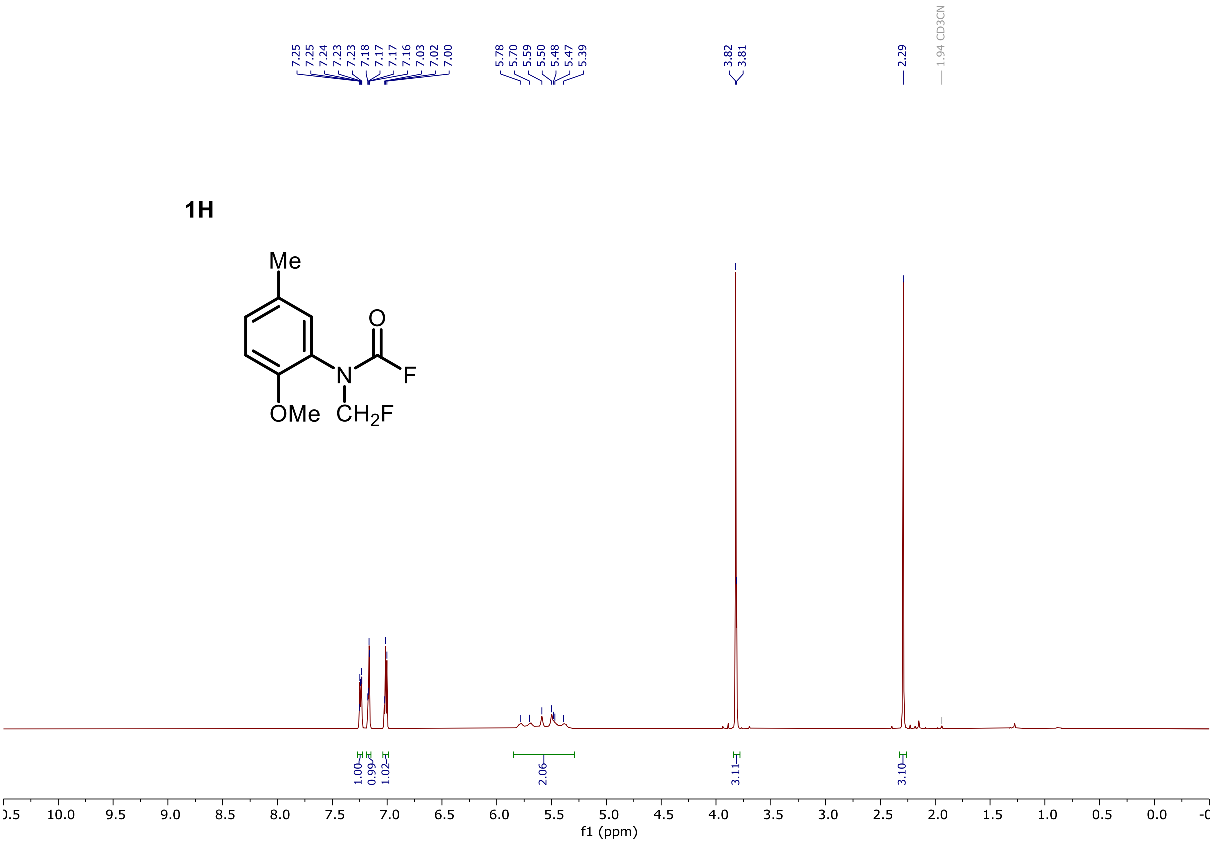

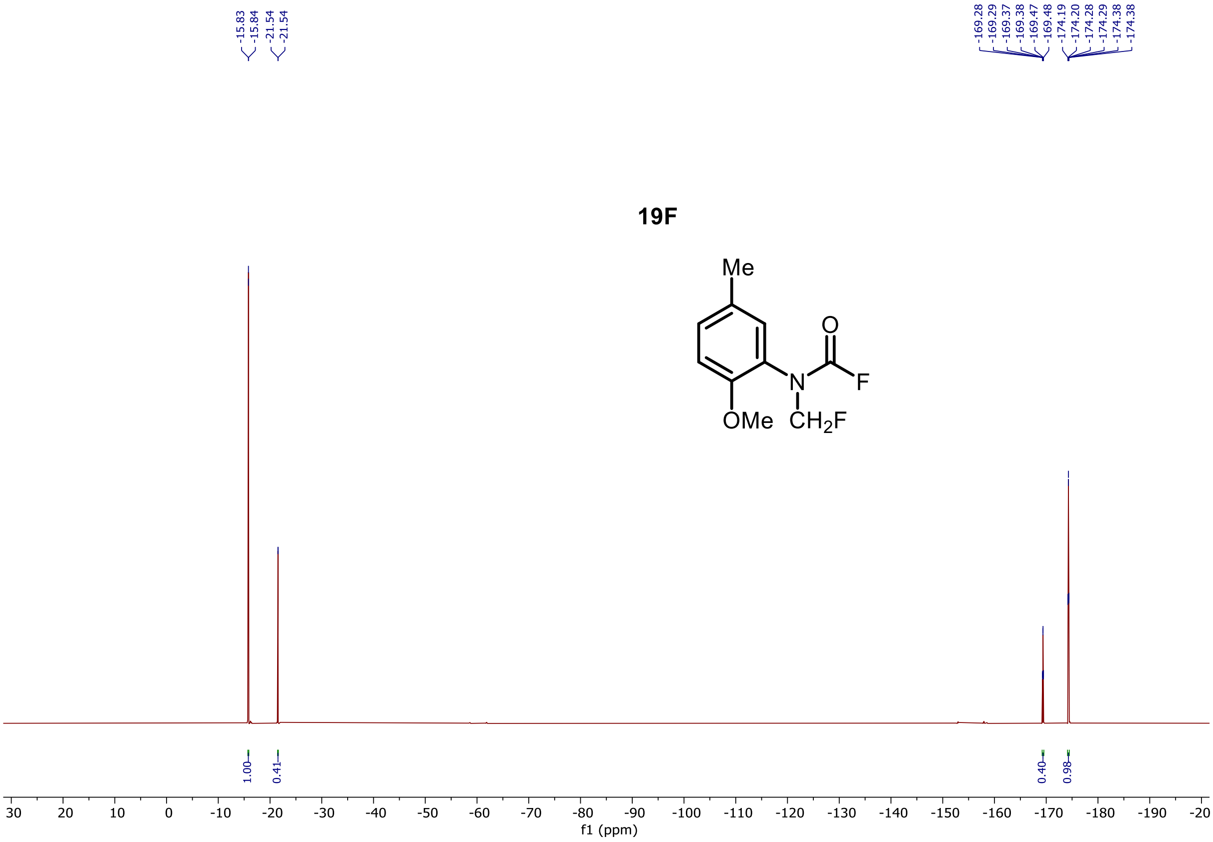

19F
Me
O
N
F
OMe
CH2F
f1 (ppm)

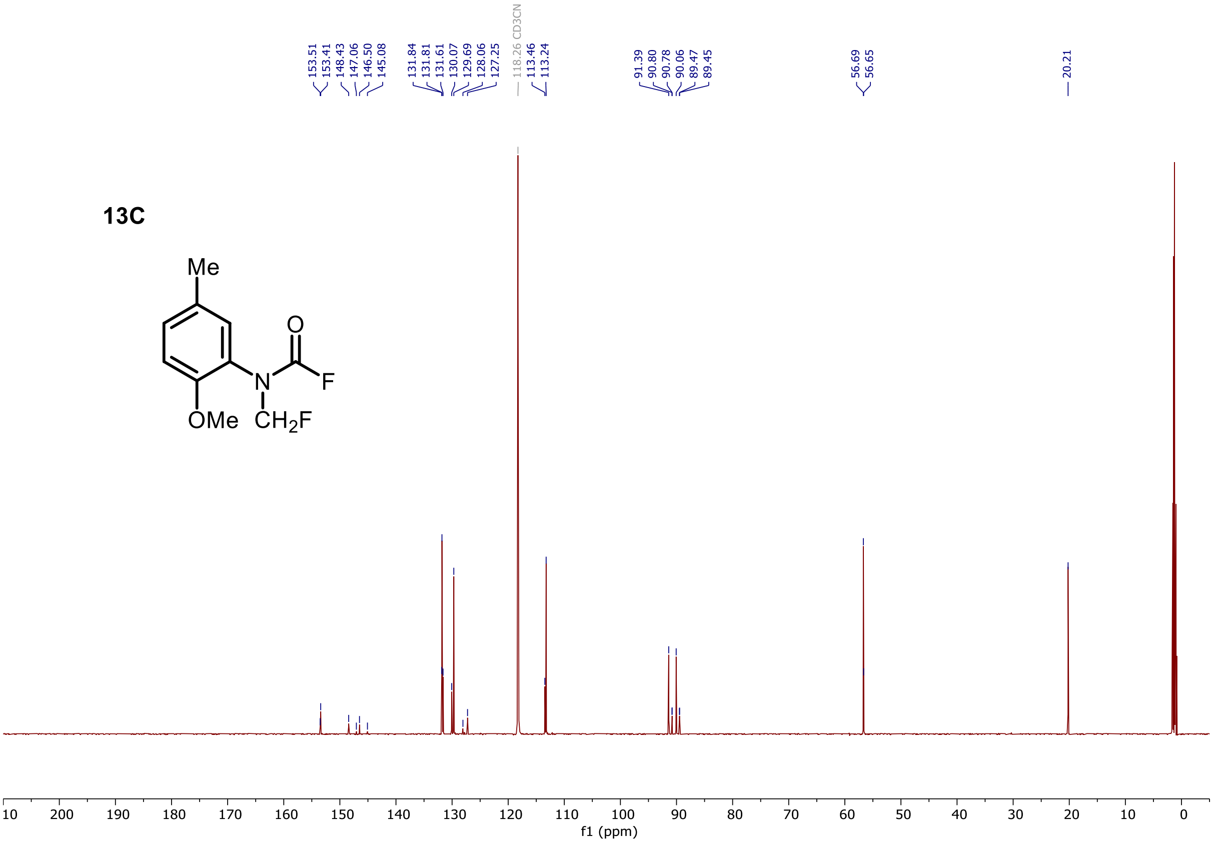

13C
Me
O
N
F
OMe
CH2F
118.26 CD3CN
f1 (ppm)

(4-Bromobenzyl)(fluoromethyl)carbamic fluoride (8)

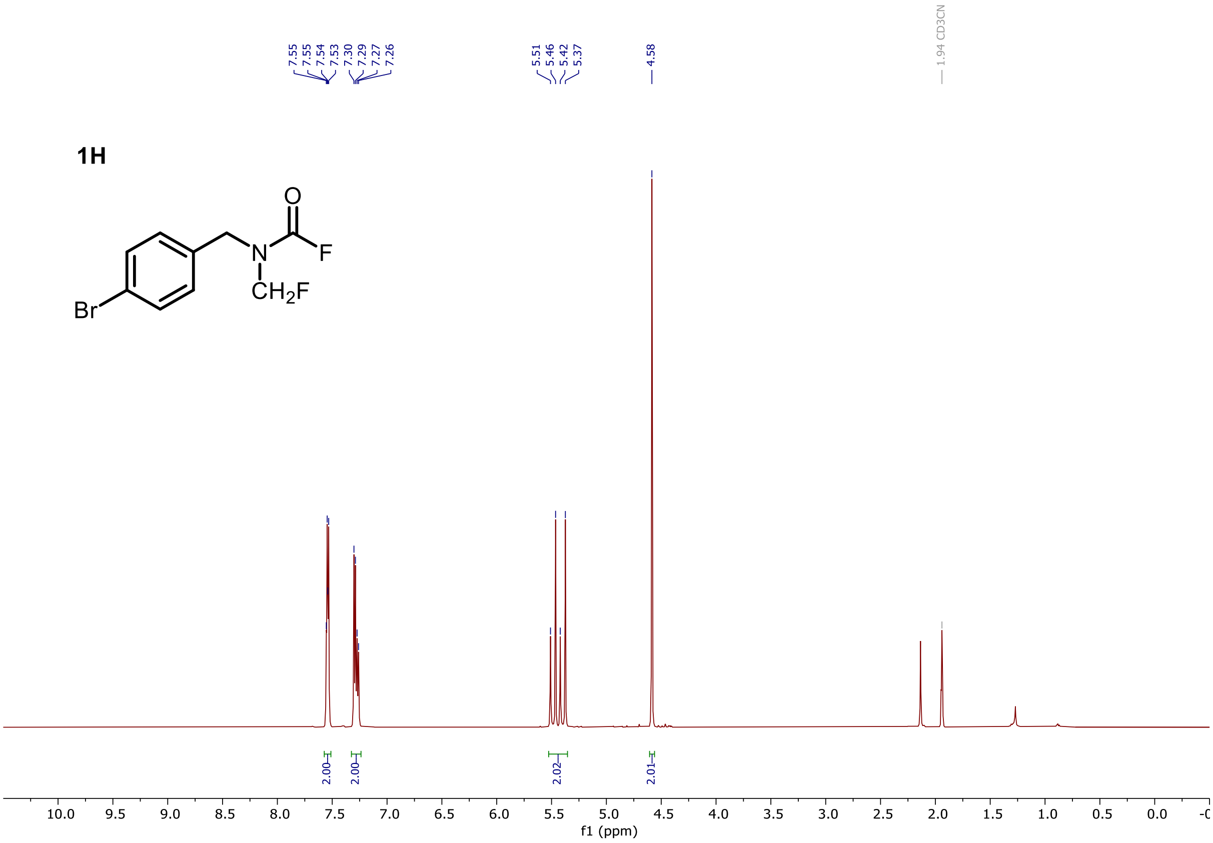

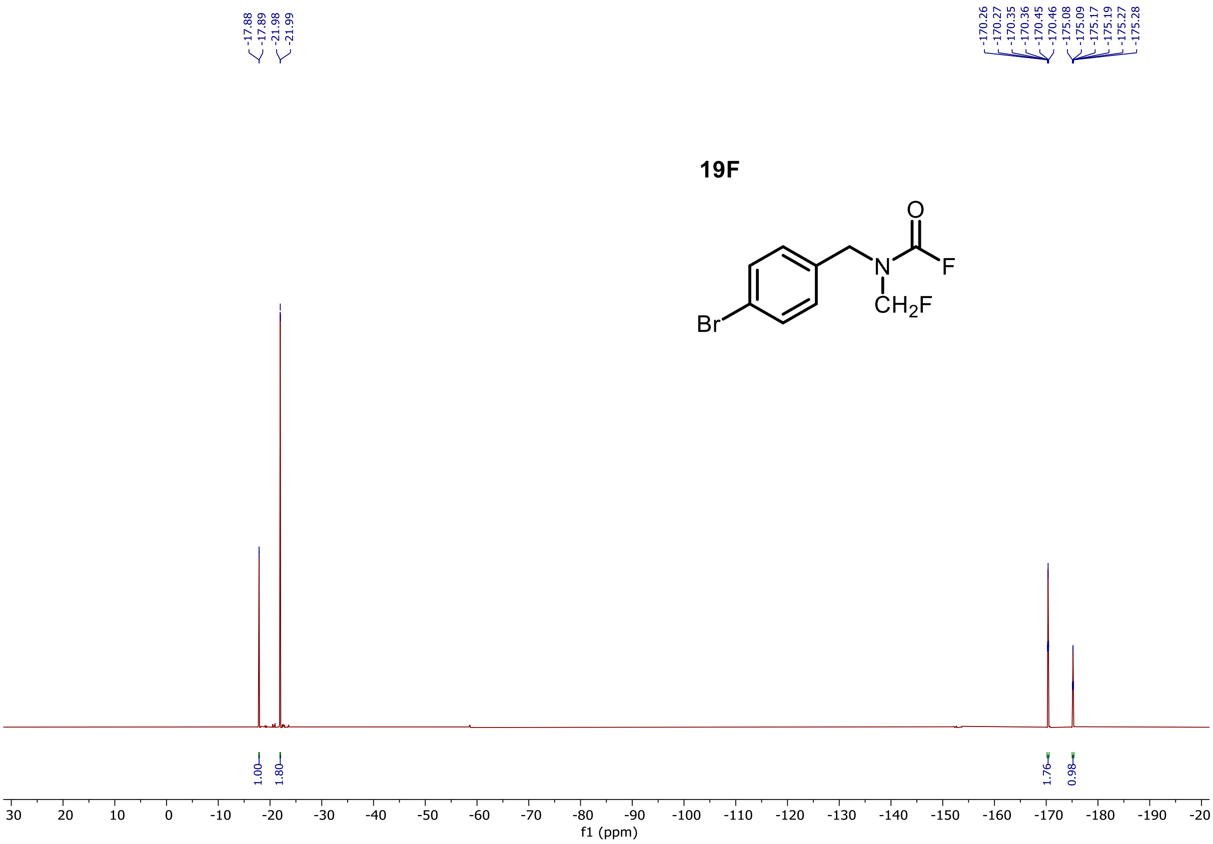

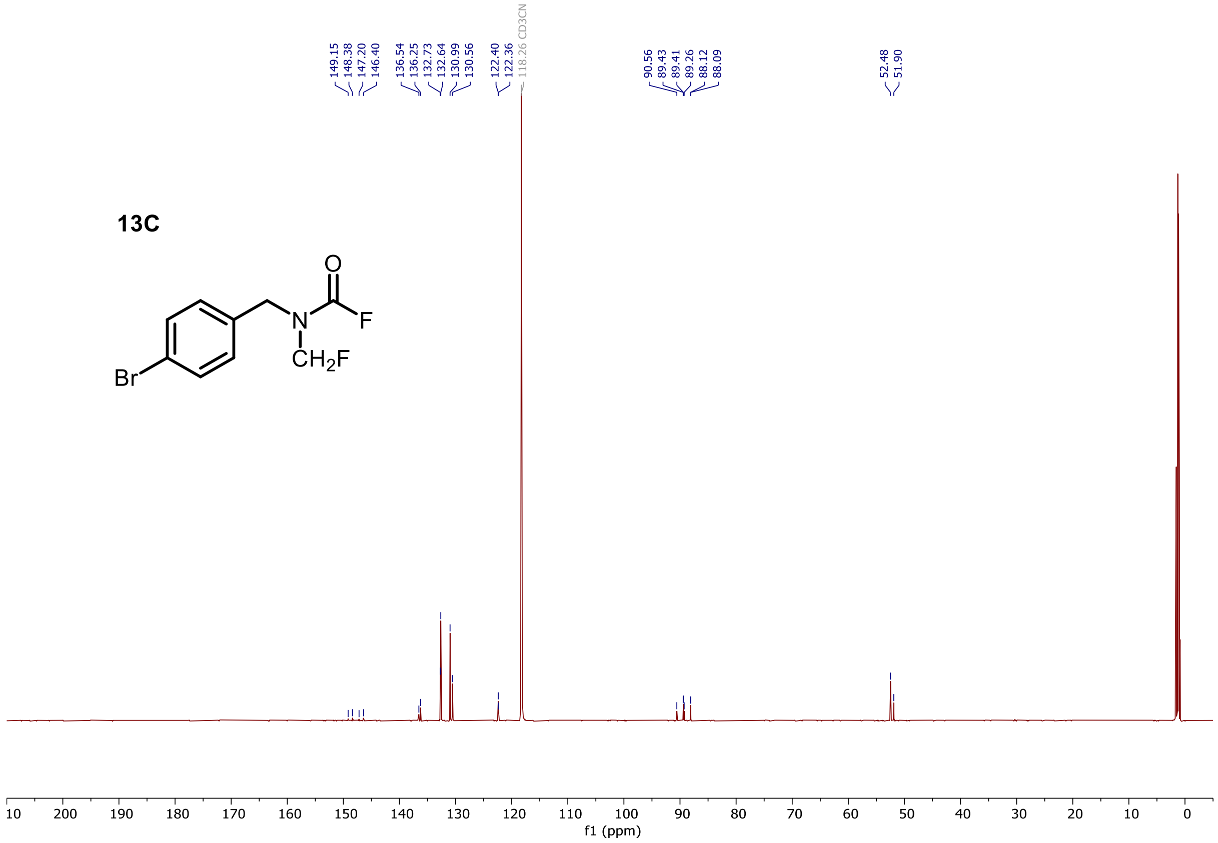

(2-Bromophenethyl)(fluoromethyl)carbamic fluoride (9)

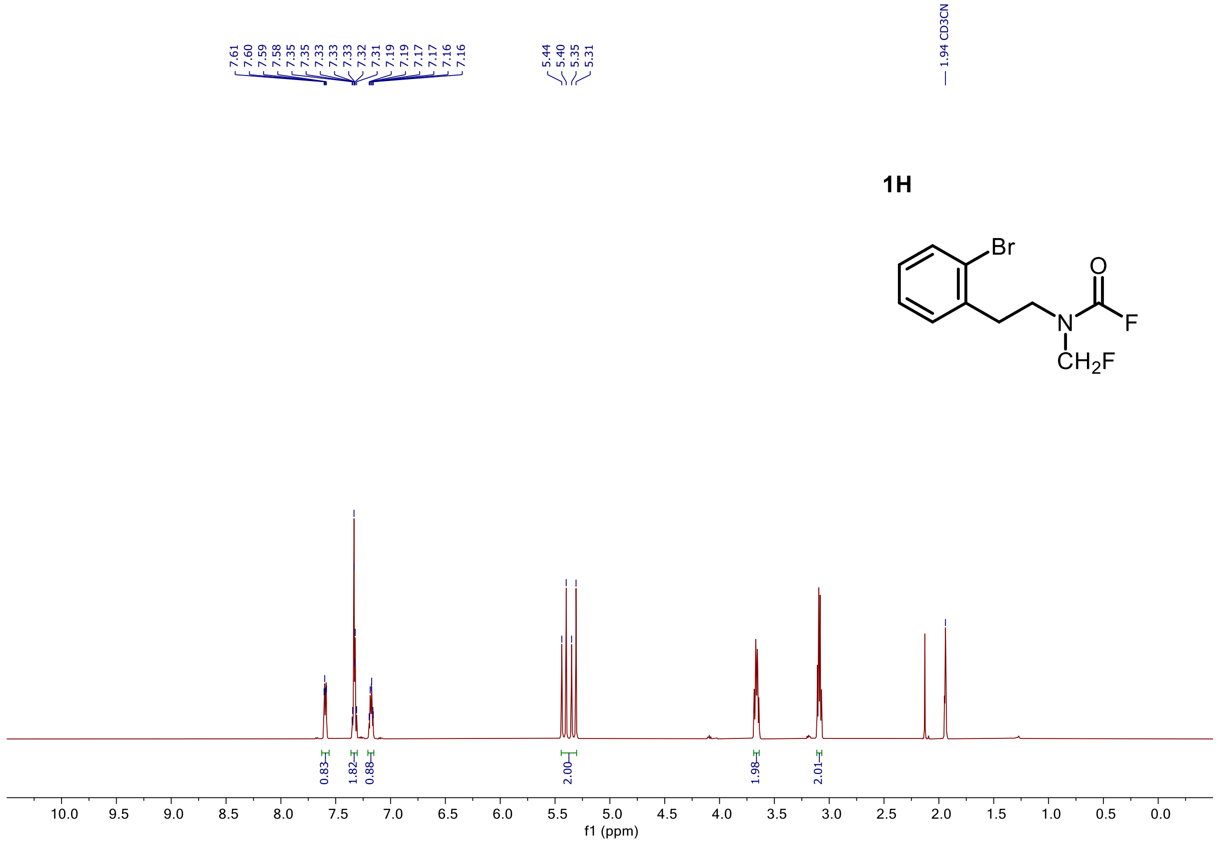

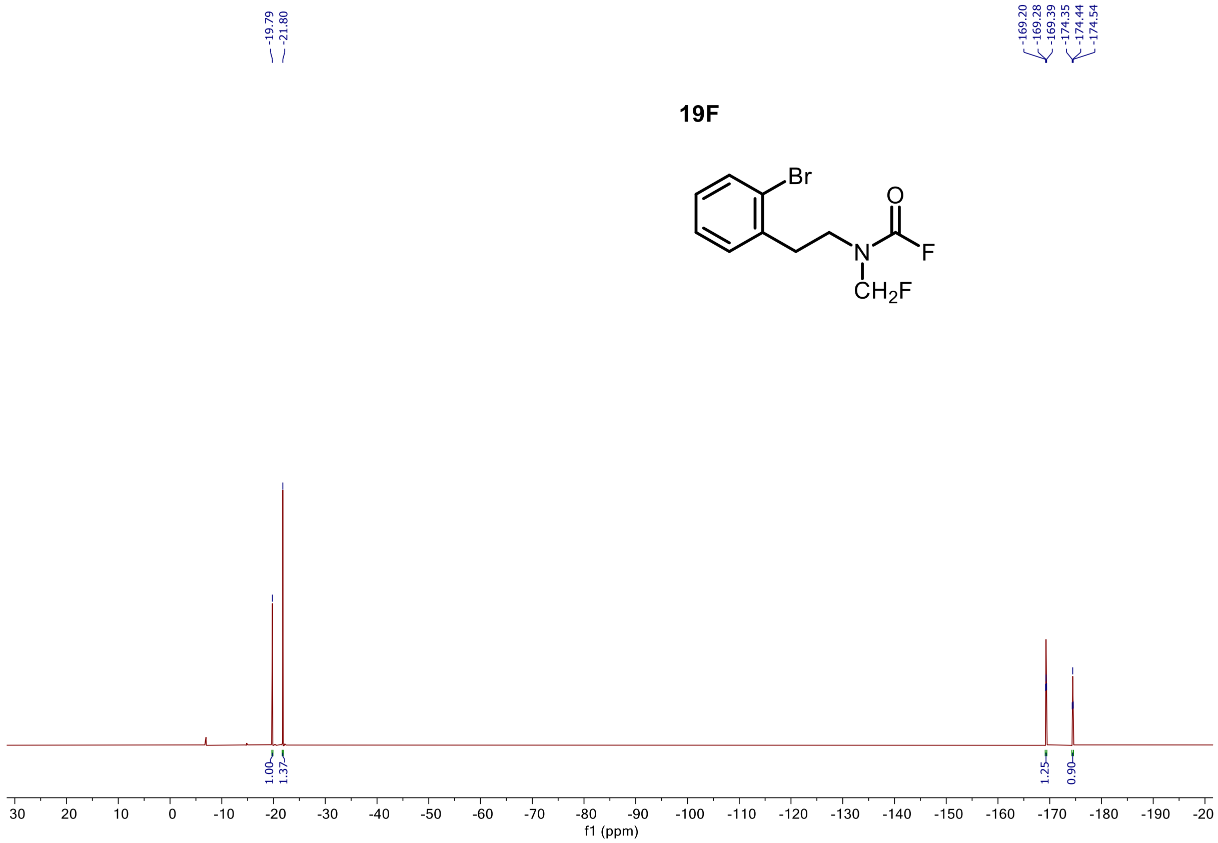

-19.79
-21.80
-169.20
-169.28
-169.39
-174.35
-174.44
-174.54
19F
Br
O
N
F
CH2F
1.00
1.37
1.25
0.90
30 20 10 0 -10 -20 -30 -40 -50 -60 -70 -80 -90 -100 -110 -120 -130 -140 -150 -160 -170 -180 -190 -20
f1 (ppm)

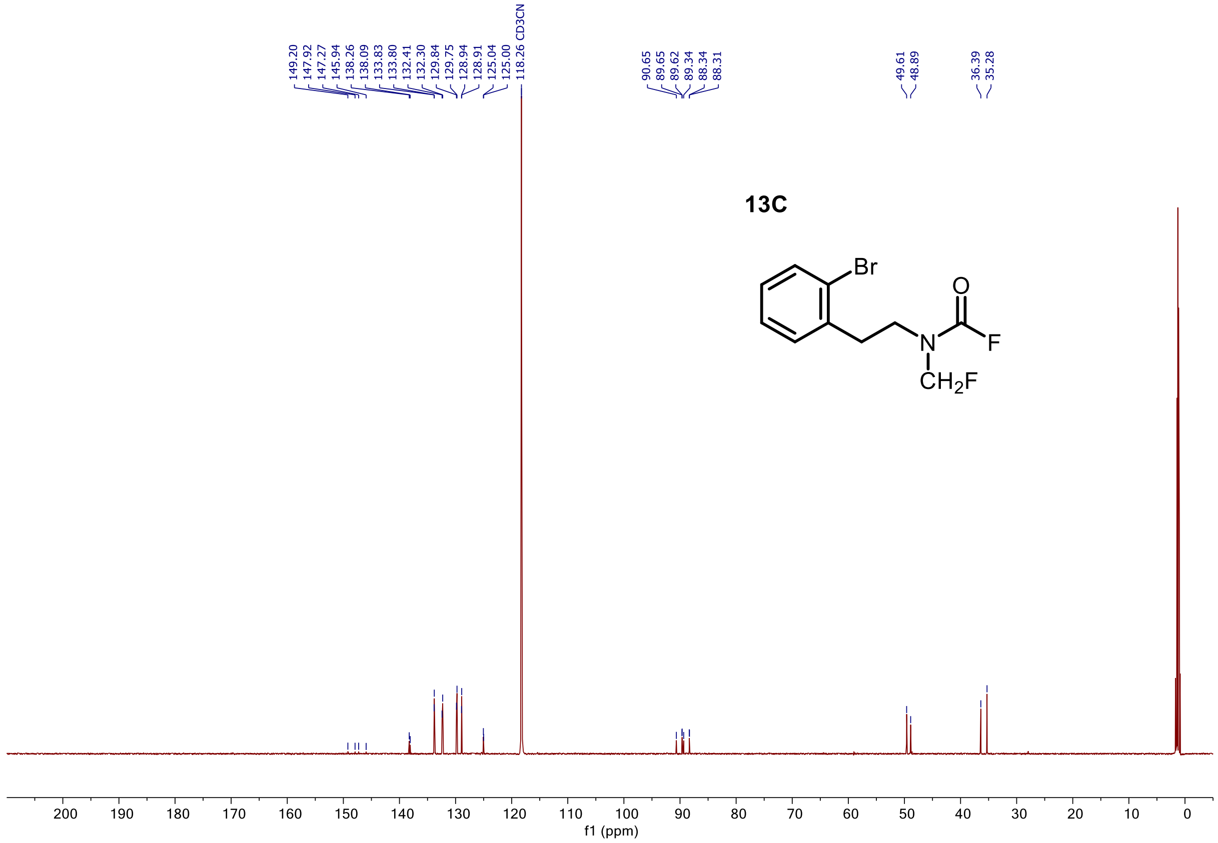

149.20
147.92
147.27
145.94
138.26
138.09
133.83
133.80
132.41
132.30
129.84
129.75
128.94
128.91
125.04
125.00
118.26 CD3CN
90.65
89.65
89.62
89.34
88.34
88.31
49.61
48.89
36.39
35.28
13C
Br
O
N
F
CH2F
200 190 180 170 160 150 140 130 120 110 100 90 80 70 60 50 40 30 20 10 0
f1 (ppm)

(3-(4-Chlorophenyl)-3-(furan-3-yl)propyl)(fluoromethyl)carbamic fluoride (10)

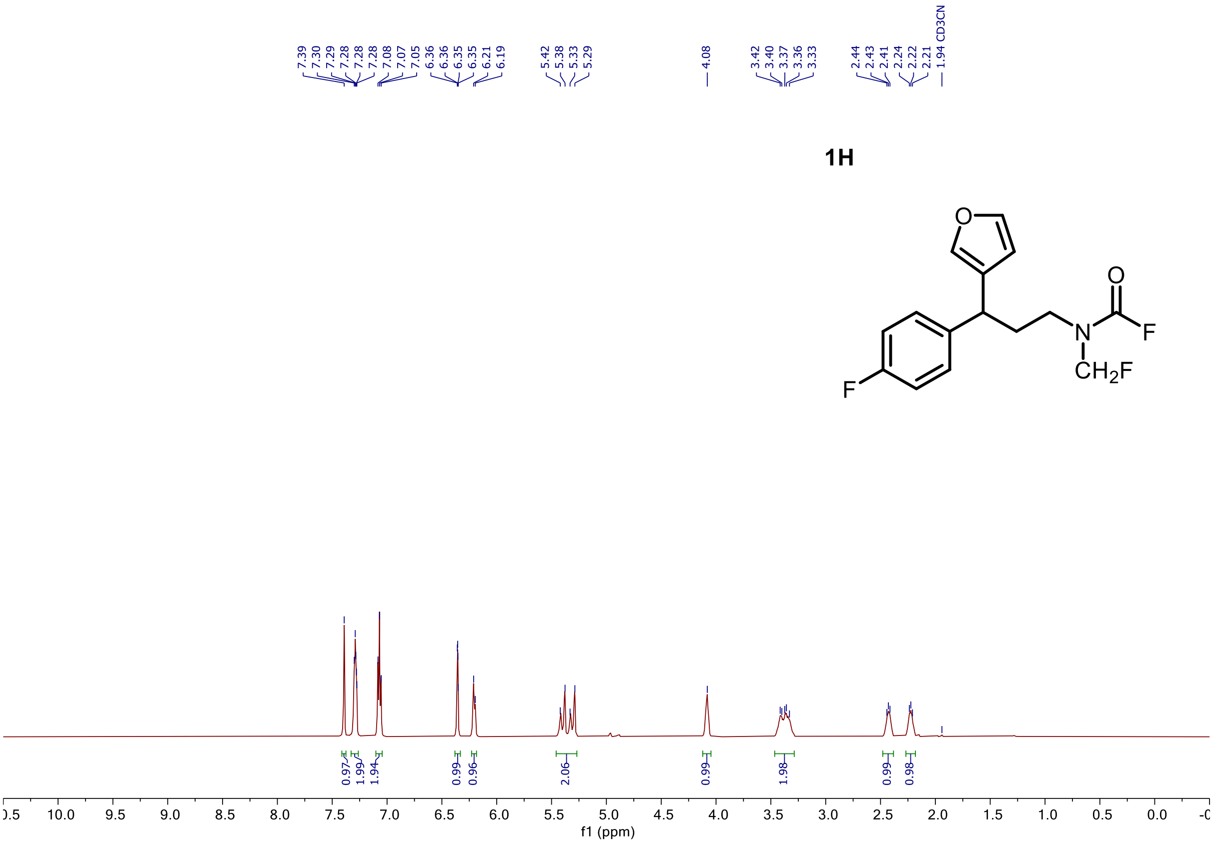

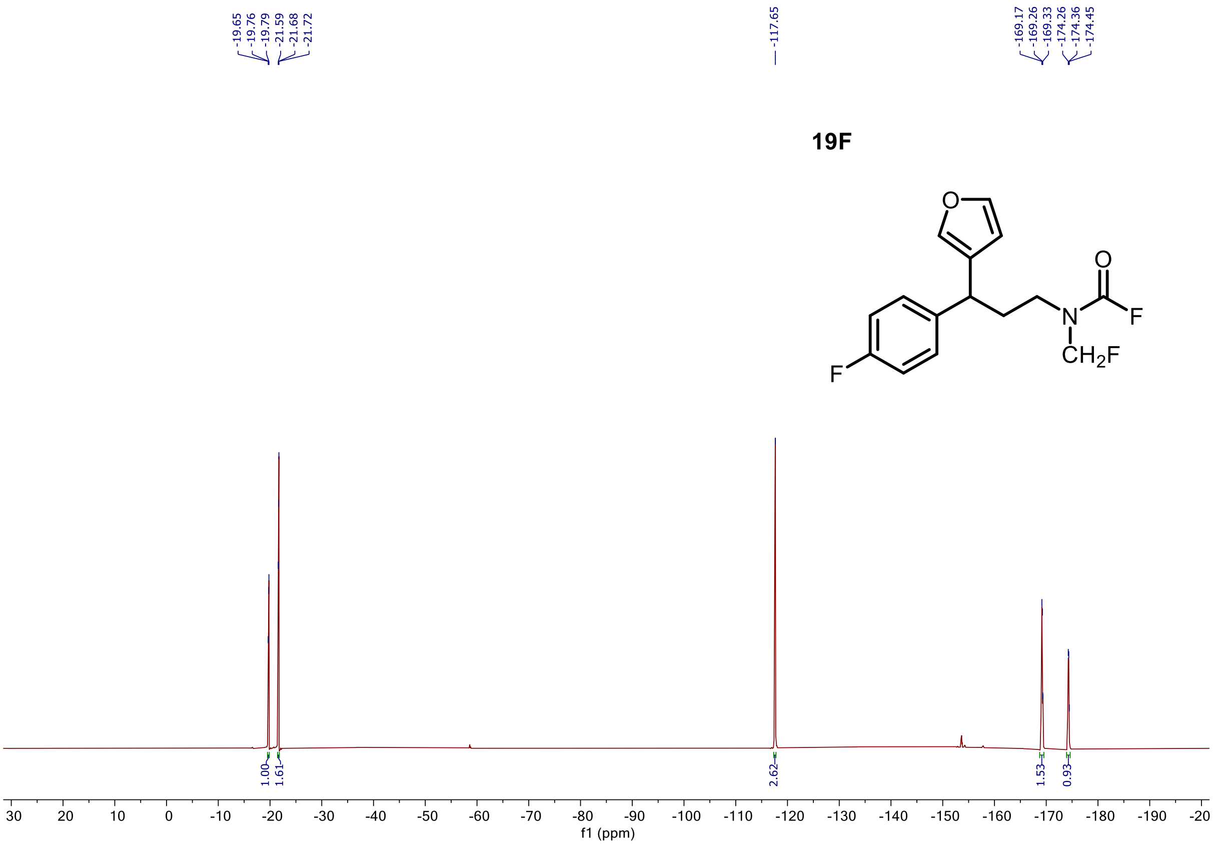

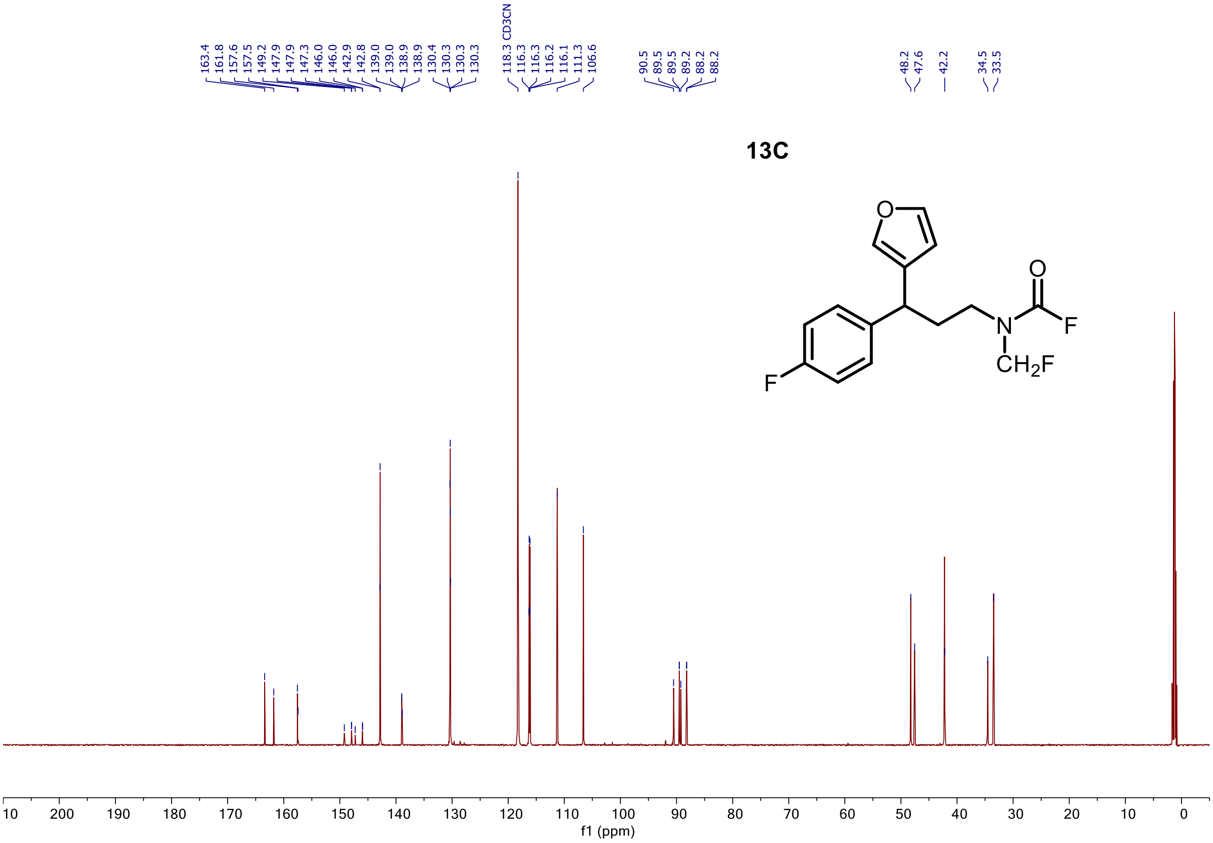

tert-Butyl 4-((fluorocarbonyl)(fluoromethyl)amino)piperidine-1-carboxylate (11)

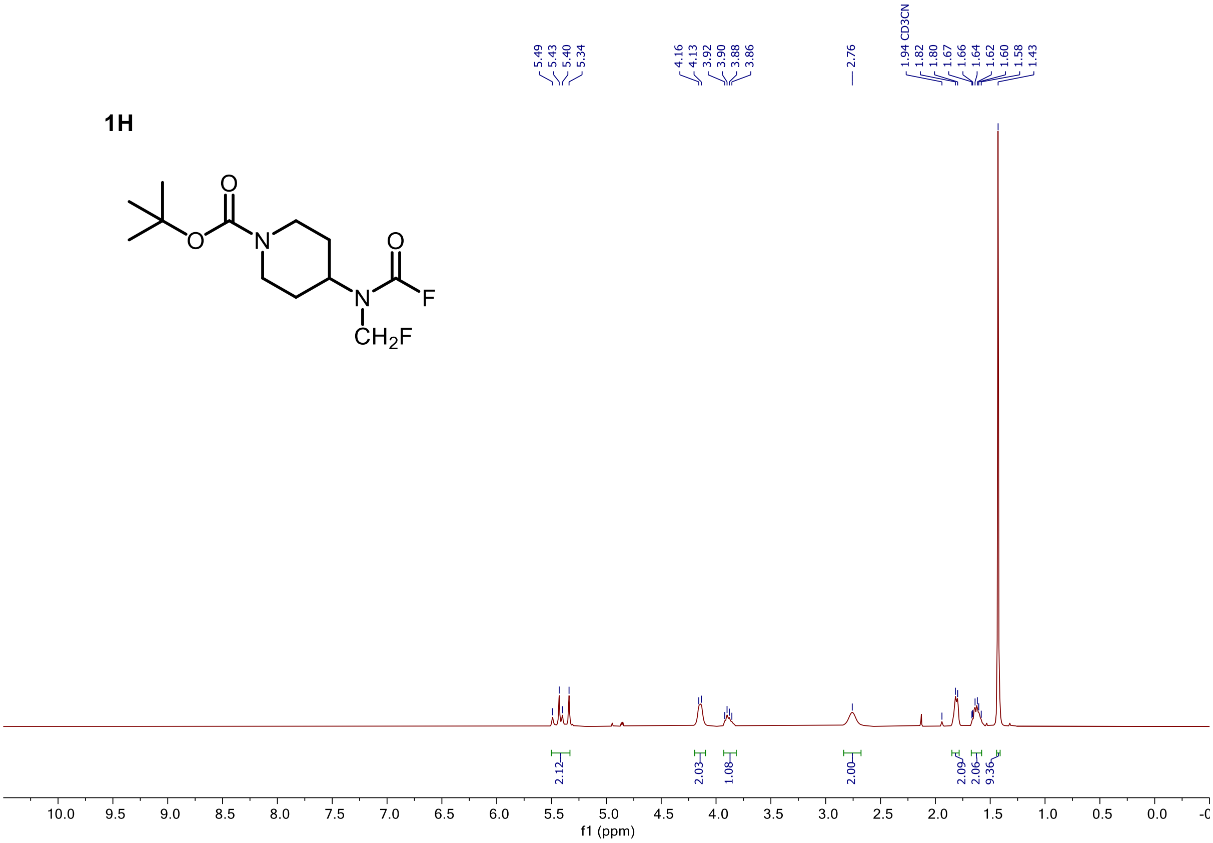

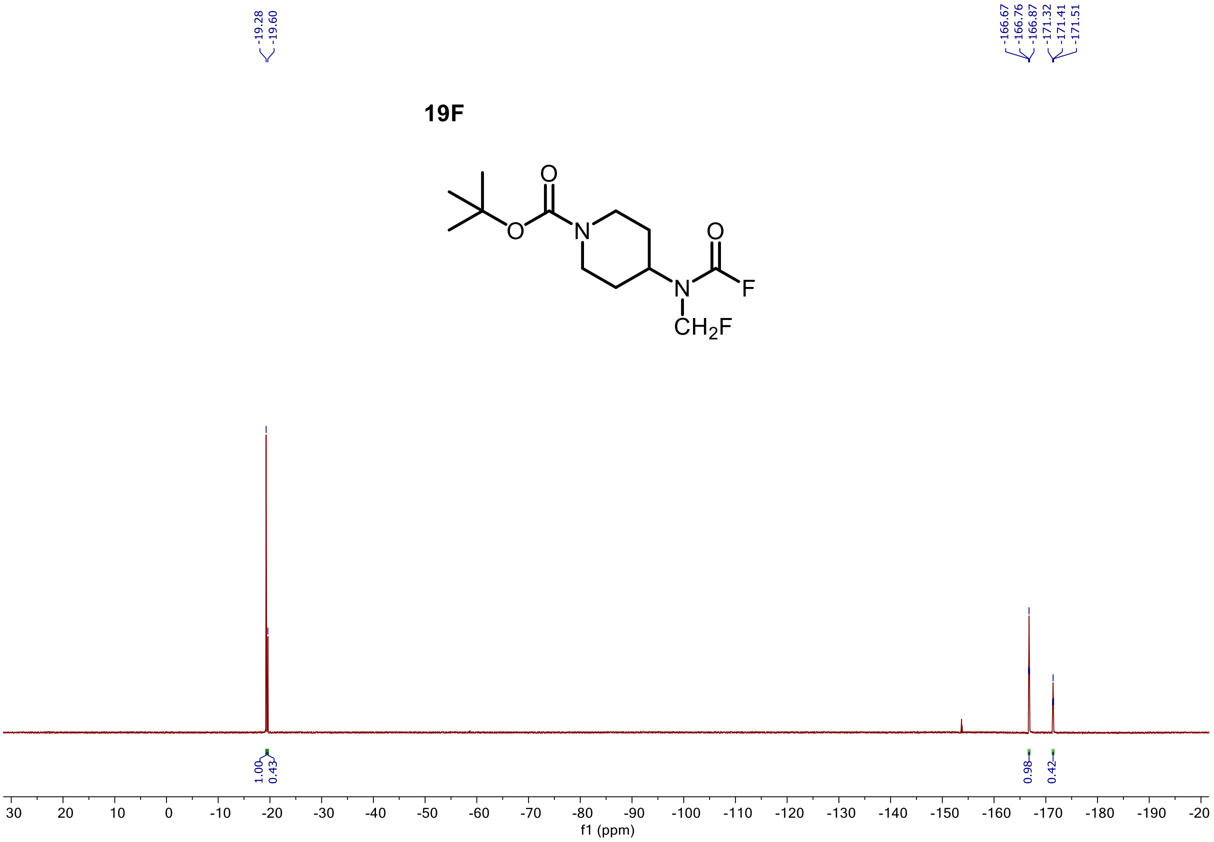
-19.28
-19.60
-166.67
-166.76
-166.87
-171.32
-171.41
-171.51
19F
O
O
N
O
N
F
CH_2F
1.00
0.43
0.98
0.42
30 20 10 0 -10 -20 -30 -40 -50 -60 -70 -80 -90 -100 -110 -120 -130 -140 -150 -160 -170 -180 -190 -20
f1 (ppm)

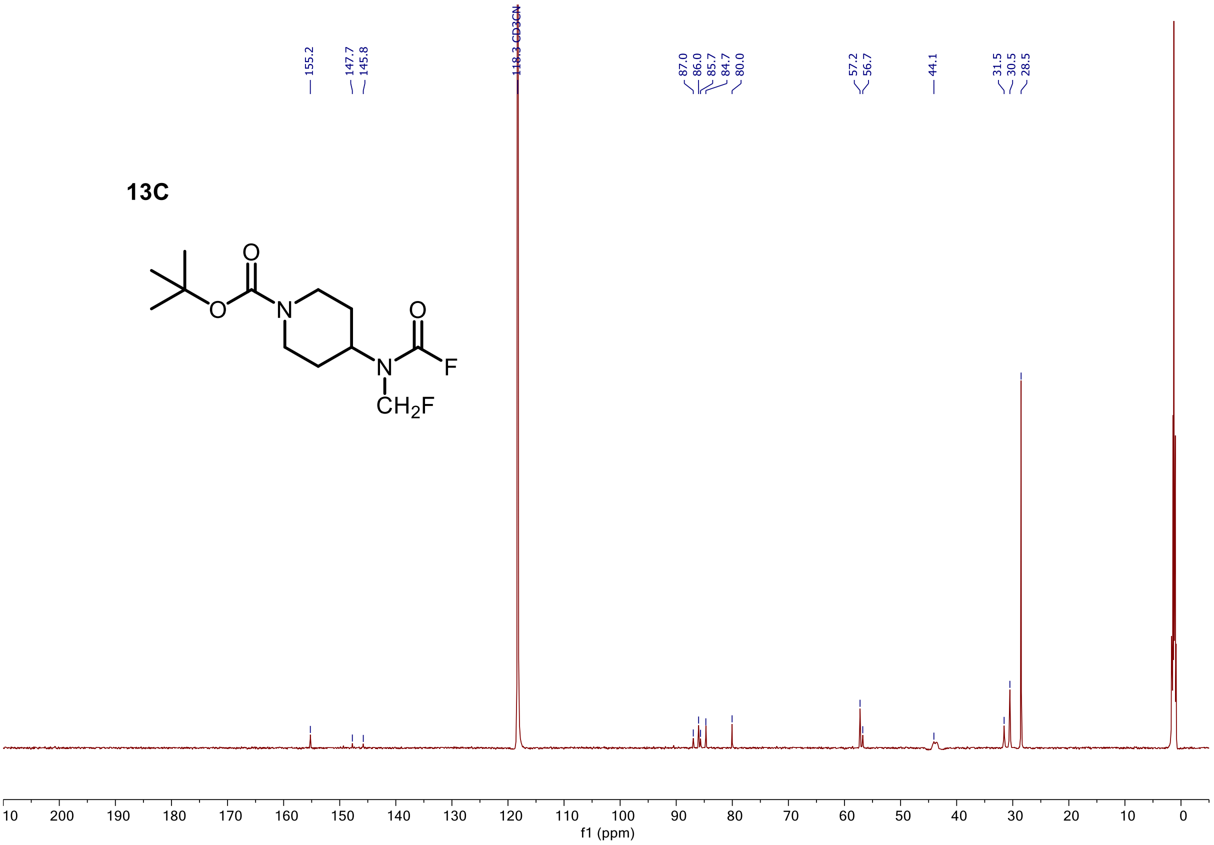
155.2
147.7
145.8
118.3 CD3CN
87.0
86.0
85.7
84.7
80.0
57.2
56.7
44.1
31.5
30.5
28.5
13C
O
O
N
O
N
F
CH_2F
10 200 190 180 170 160 150 140 130 120 110 100 90 80 70 60 50 40 30 20 10 0
f1 (ppm)

Methyl *N*-(fluorocarbonyl)-*N*-(fluoromethyl)-*L*-phenylalaninate (12)

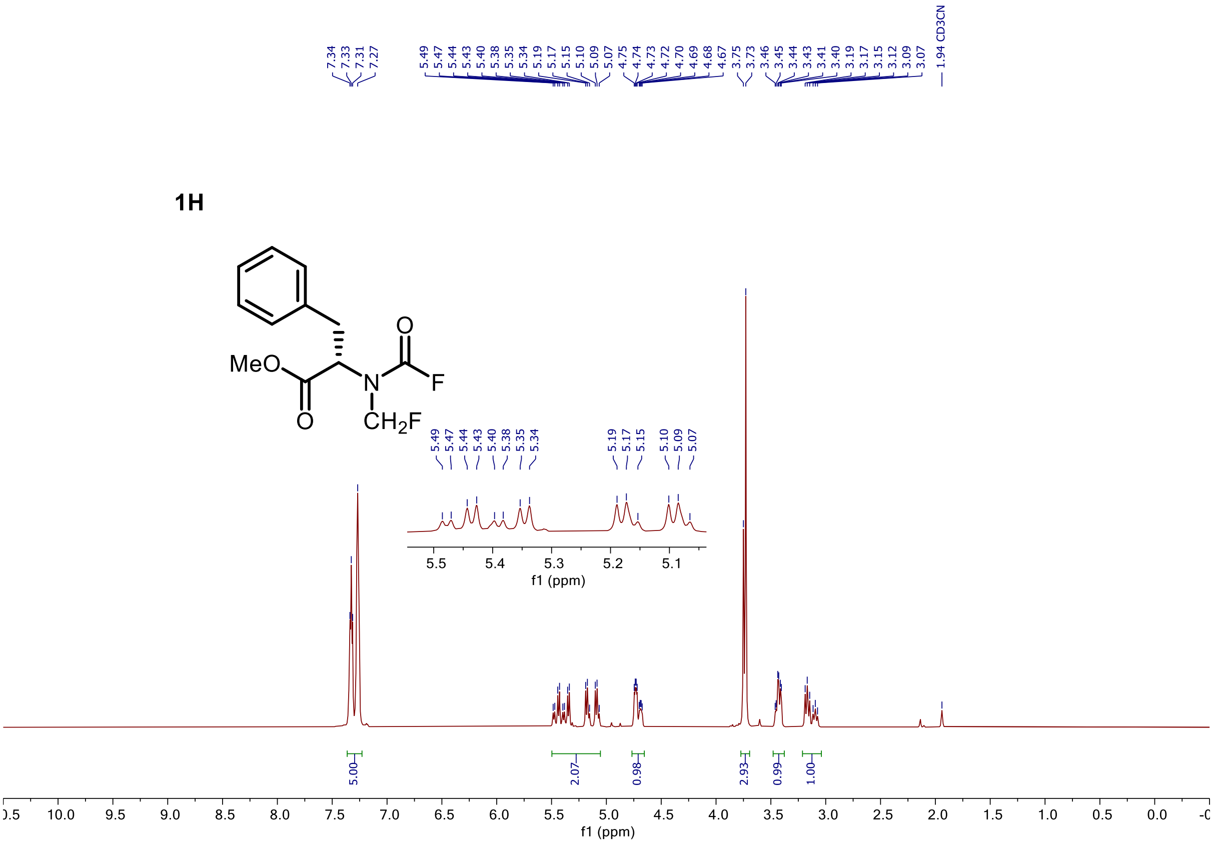

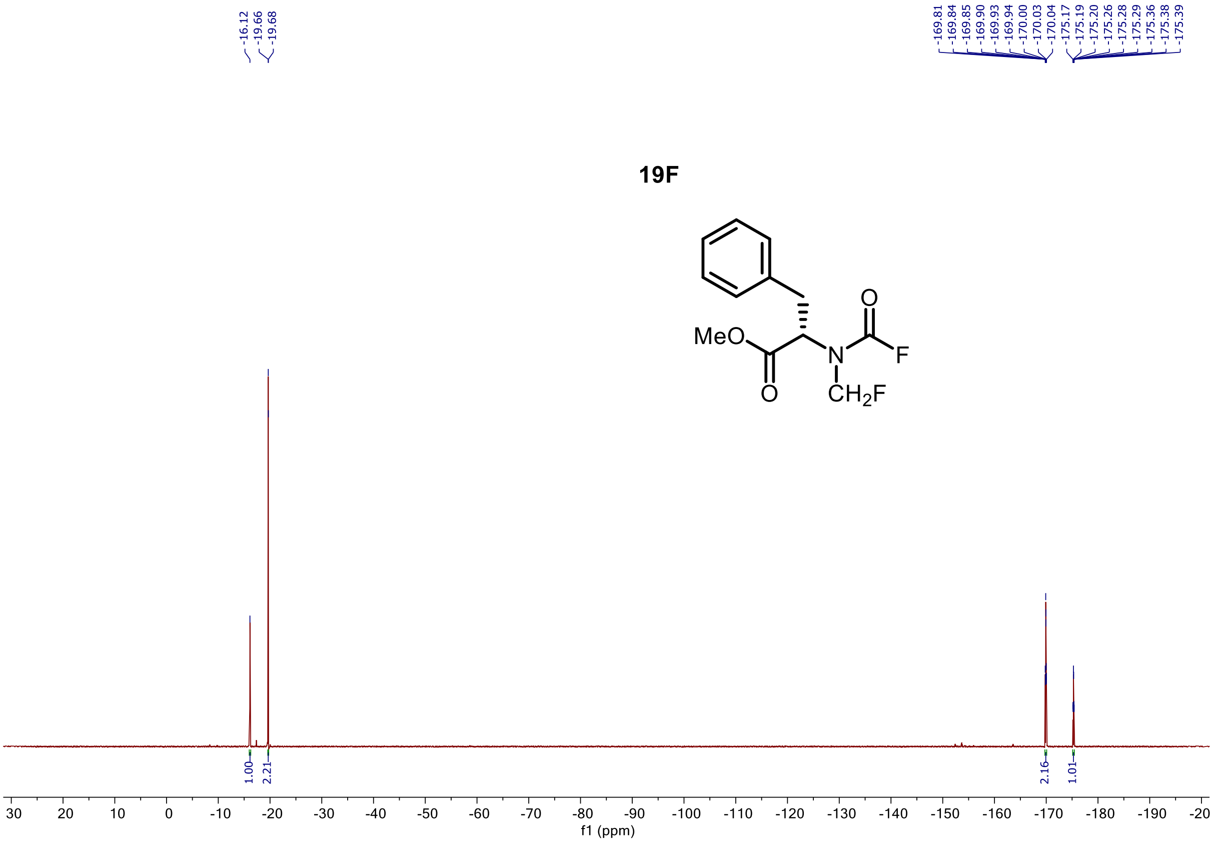

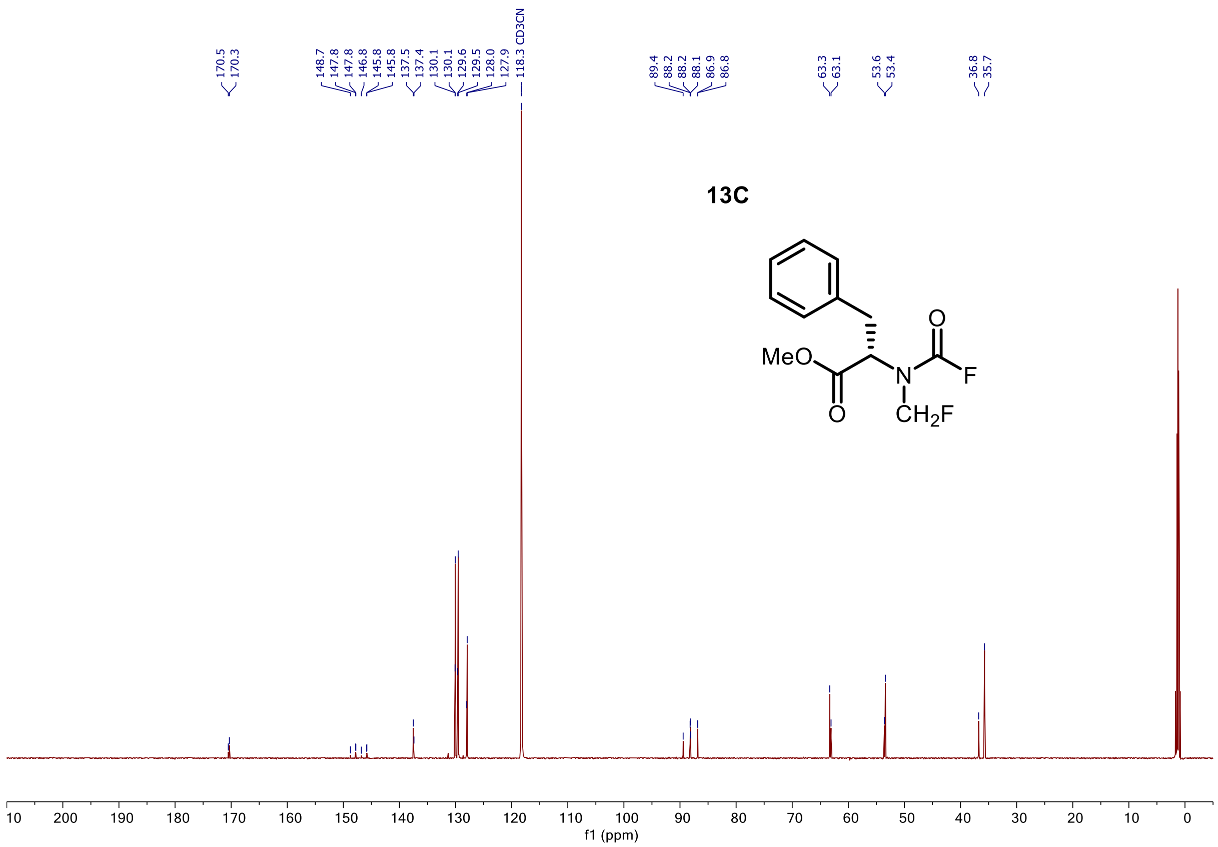

(1-(2,6-Dimethylphenoxy)propan-2-yl)(fluoromethyl)carbamic fluoride (13)

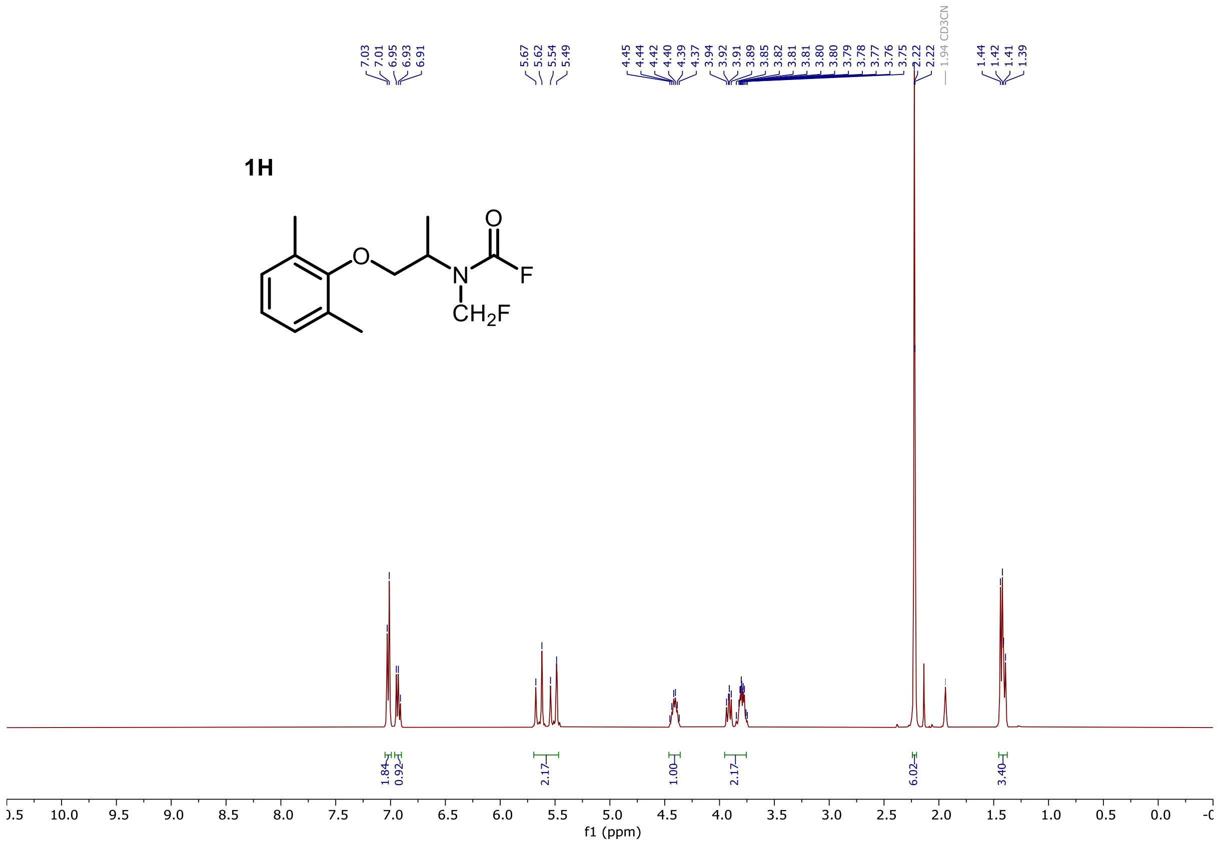

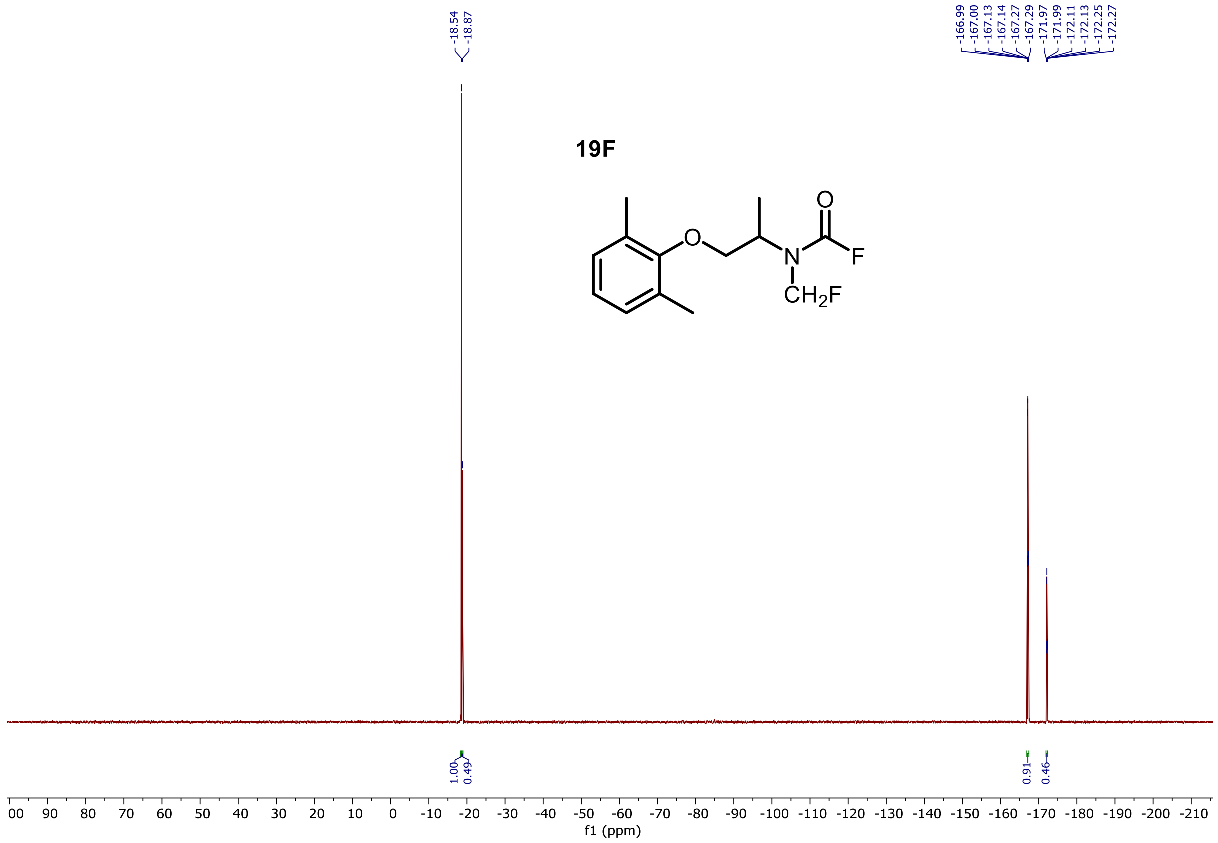

-18.54
-18.87
-166.99
-167.00
-167.13
-167.14
-167.27
-167.29
-171.97
-171.99
-172.11
-172.13
-172.25
-172.27
19F
O
O
N
F
CH2F
1.00
0.49
0.91
0.46
00 90 80 70 60 50 40 30 20 10 0 -10 -20 -30 -40 -50 -60 -70 -80 -90 -100 -110 -120 -130 -140 -150 -160 -170 -180 -190 -200 -210
f1 (ppm)

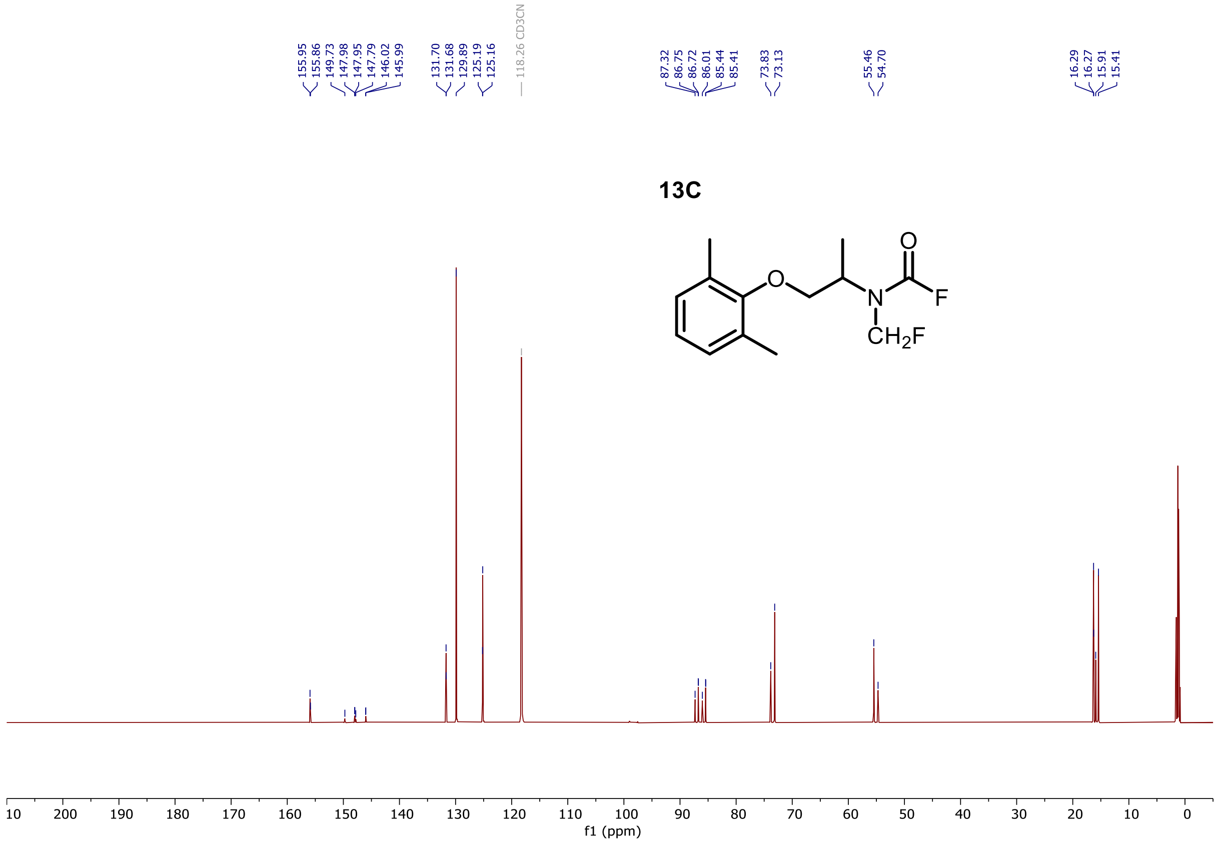

155.95
155.86
149.73
147.98
147.95
147.79
146.02
145.99
131.70
131.68
129.89
125.19
125.16
118.26 CD3CN
87.32
86.75
86.72
86.01
85.44
85.41
73.83
73.13
55.46
54.70
16.29
16.27
15.91
15.41
13C
O
O
N
F
CH2F
10 200 190 180 170 160 150 140 130 120 110 100 90 80 70 60 50 40 30 20 10 0
f1 (ppm)

((1R,2R,3R,5S,7S)-2,5-dimethyladamantan-1-yl)(fluoromethyl)carbamic fluoride (14)

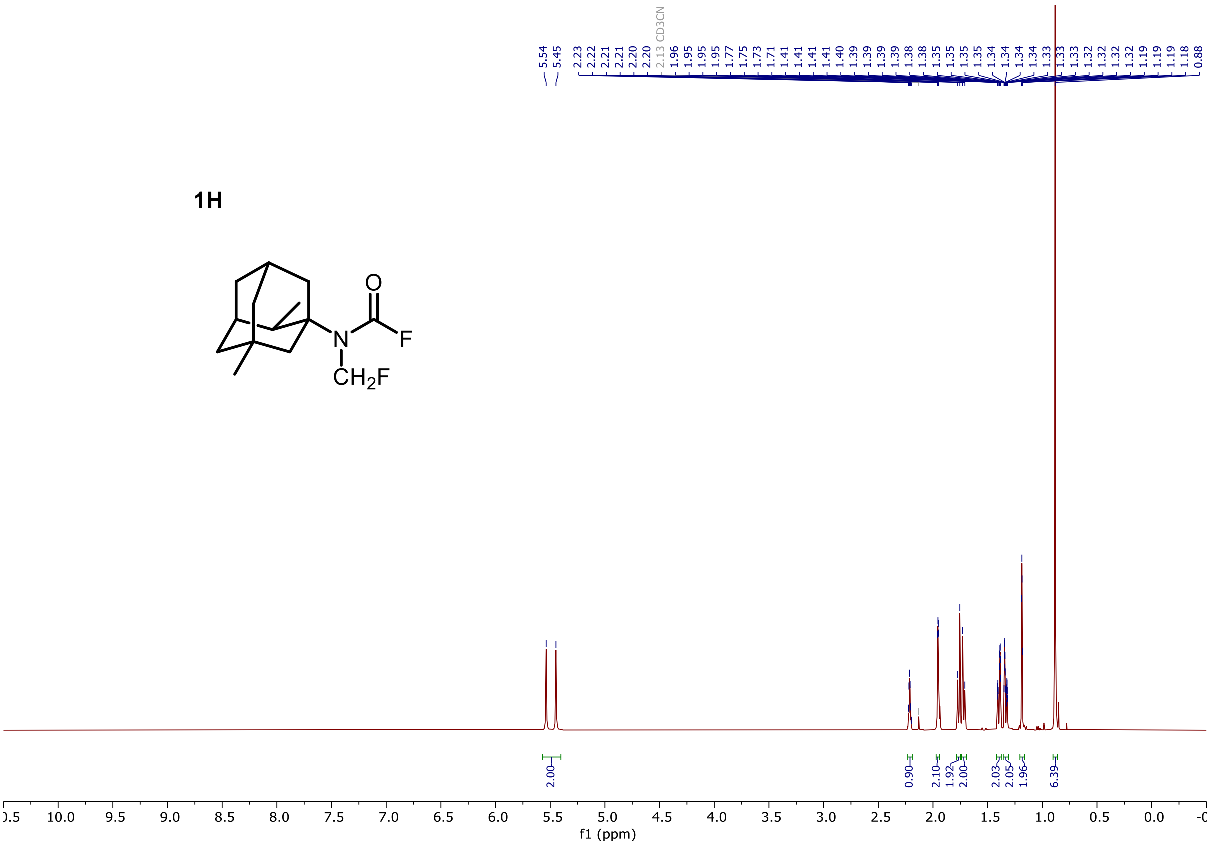

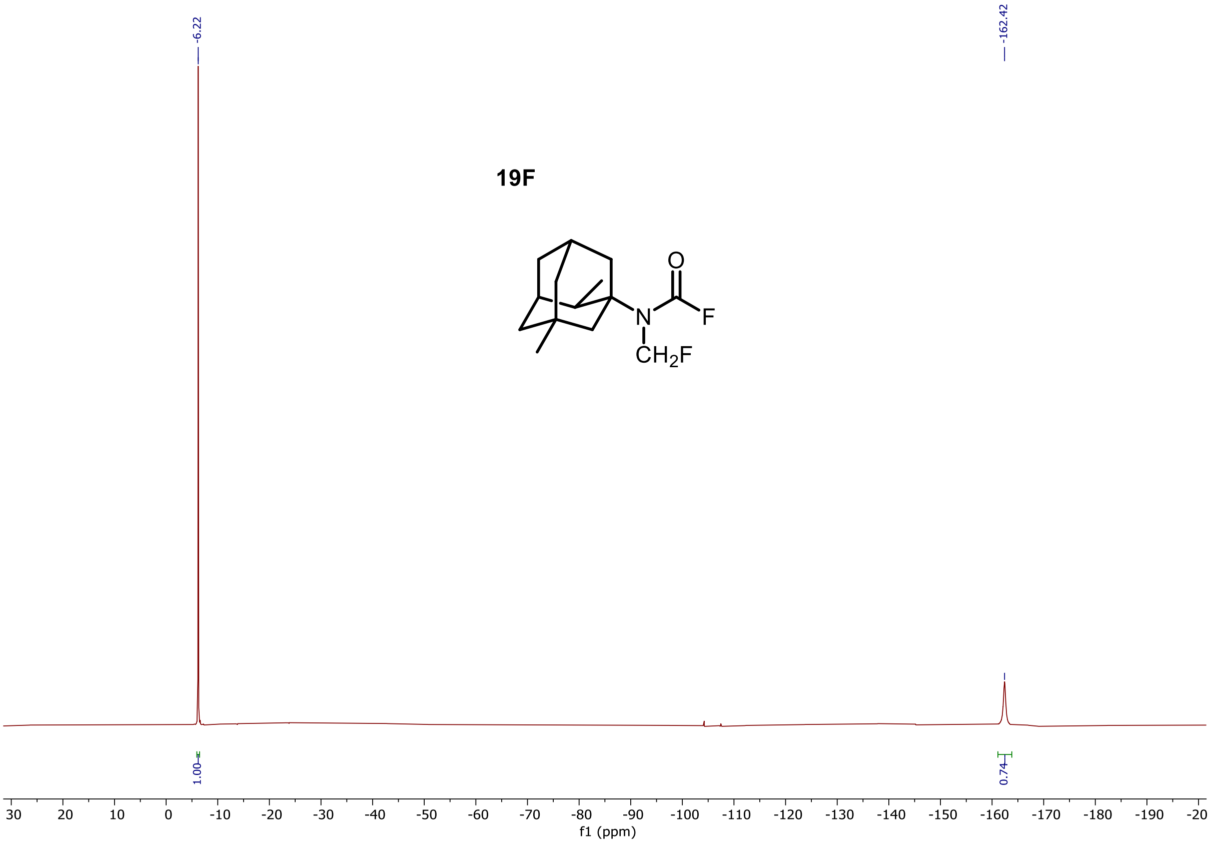

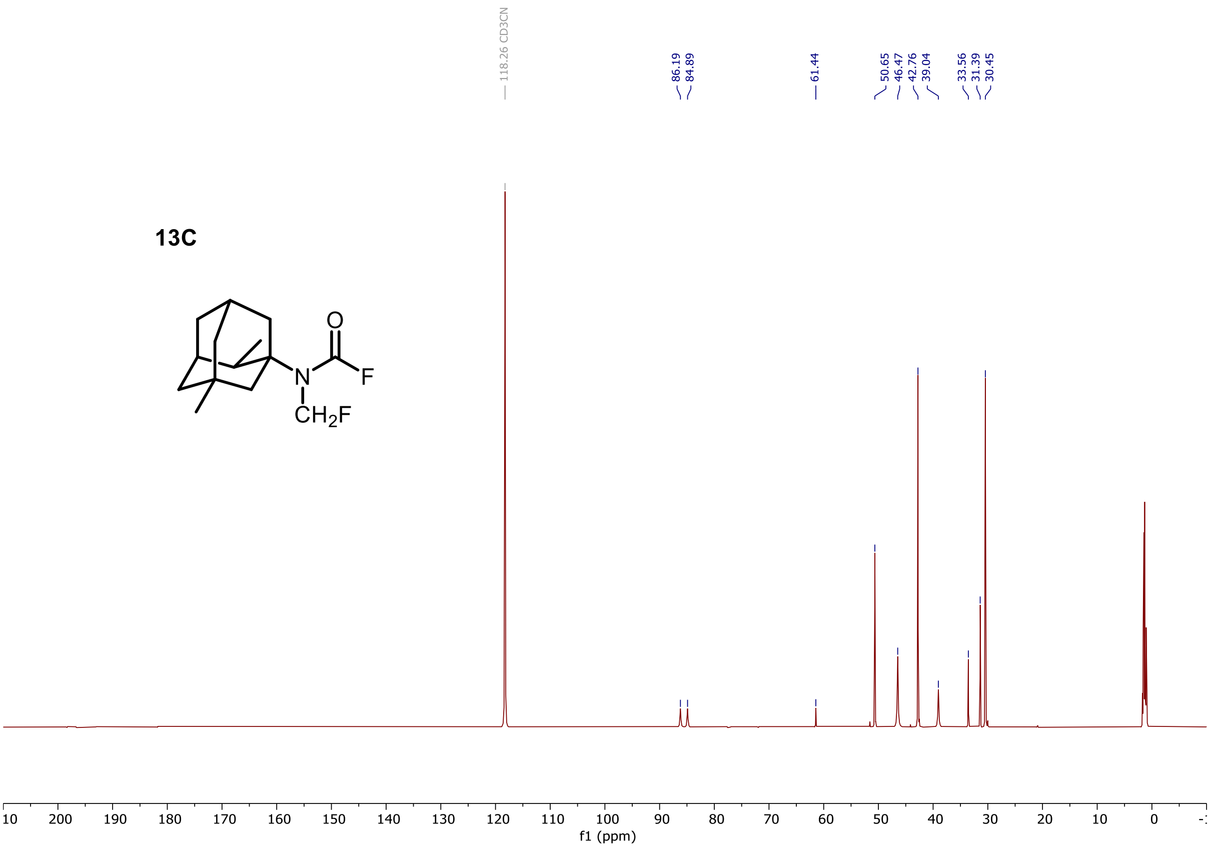

[1,1'-Biphenyl]-4-yl(fluoromethyl-*d*2)carbamic fluoride (15)

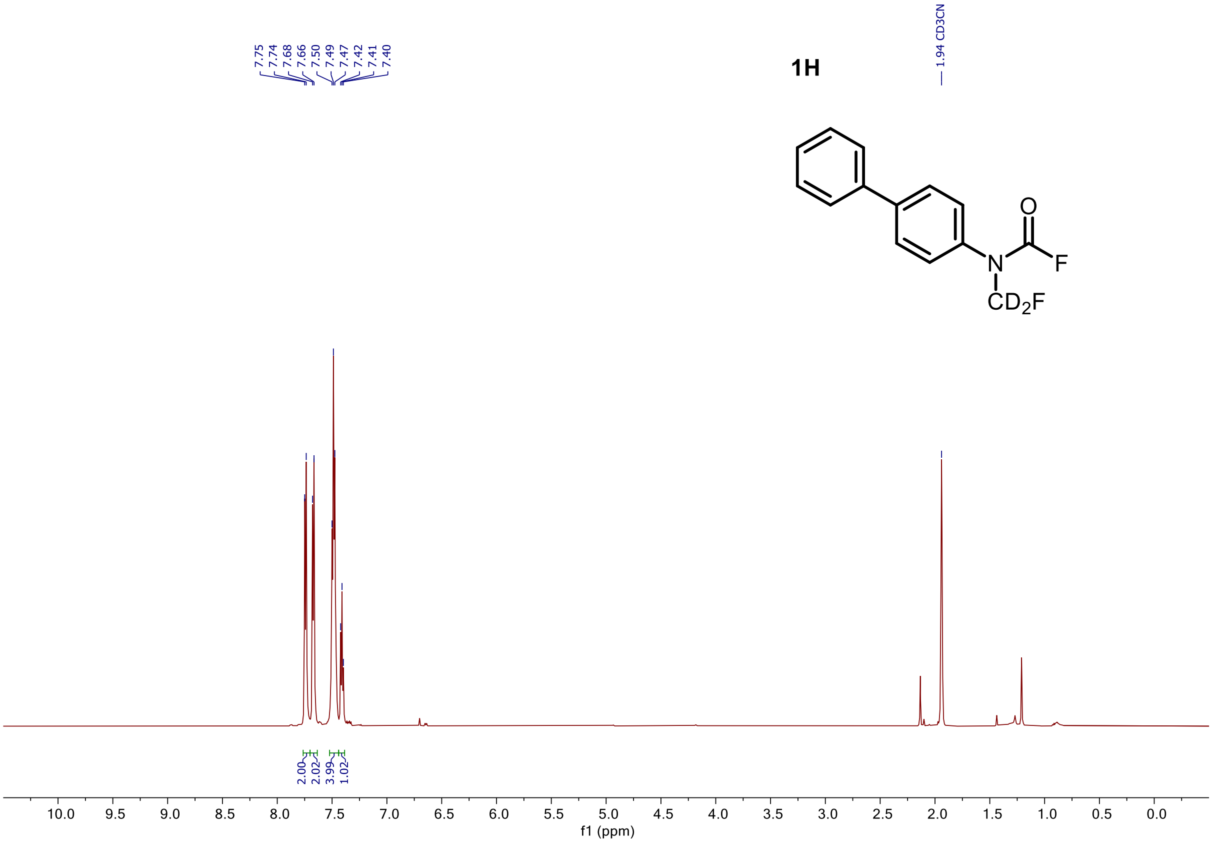

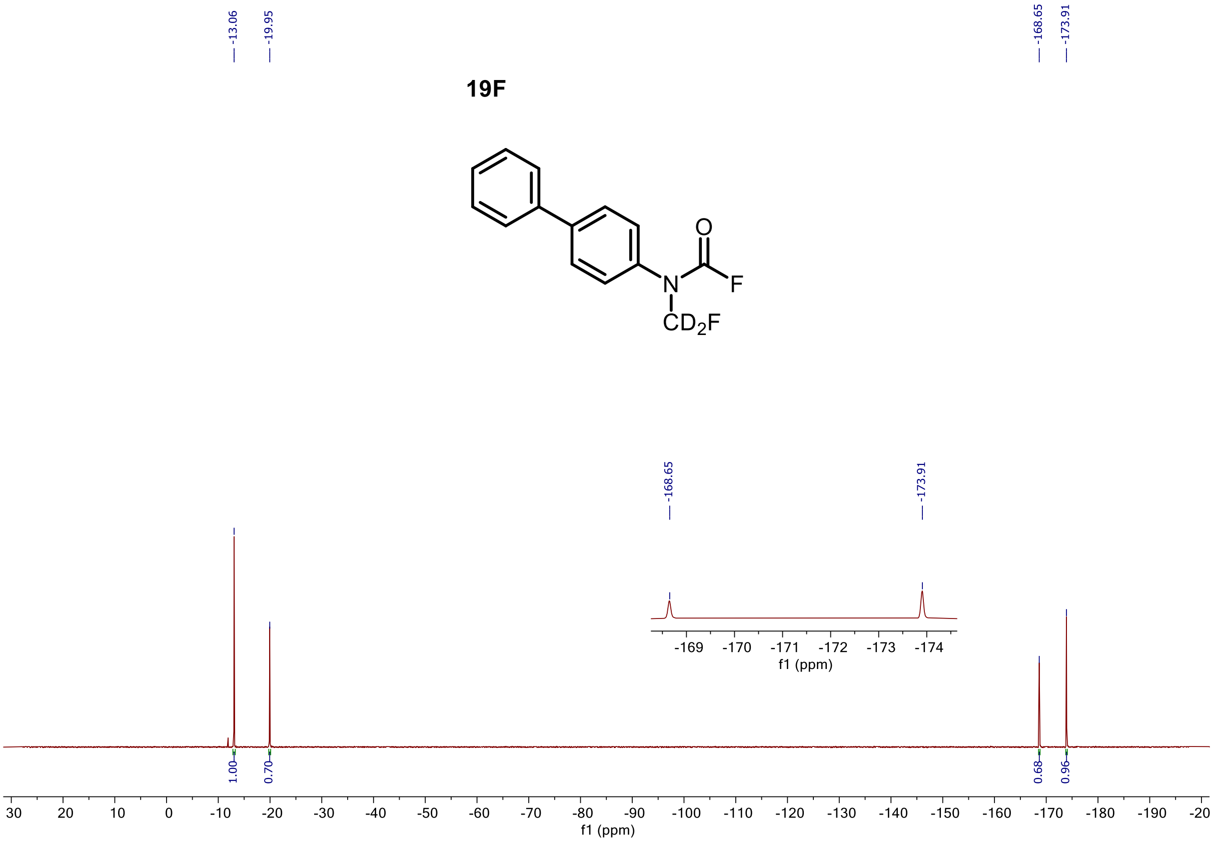
-13.06
-19.95
-168.65
-173.91
19F
O
F
N
CD2F
-169 -170 -171 -172 -173 -174
f1 (ppm)
1.00
0.70
0.68
0.96
30 20 10 0 -10 -20 -30 -40 -50 -60 -70 -80 -90 -100 -110 -120 -130 -140 -150 -160 -170 -180 -190 -20
f1 (ppm)

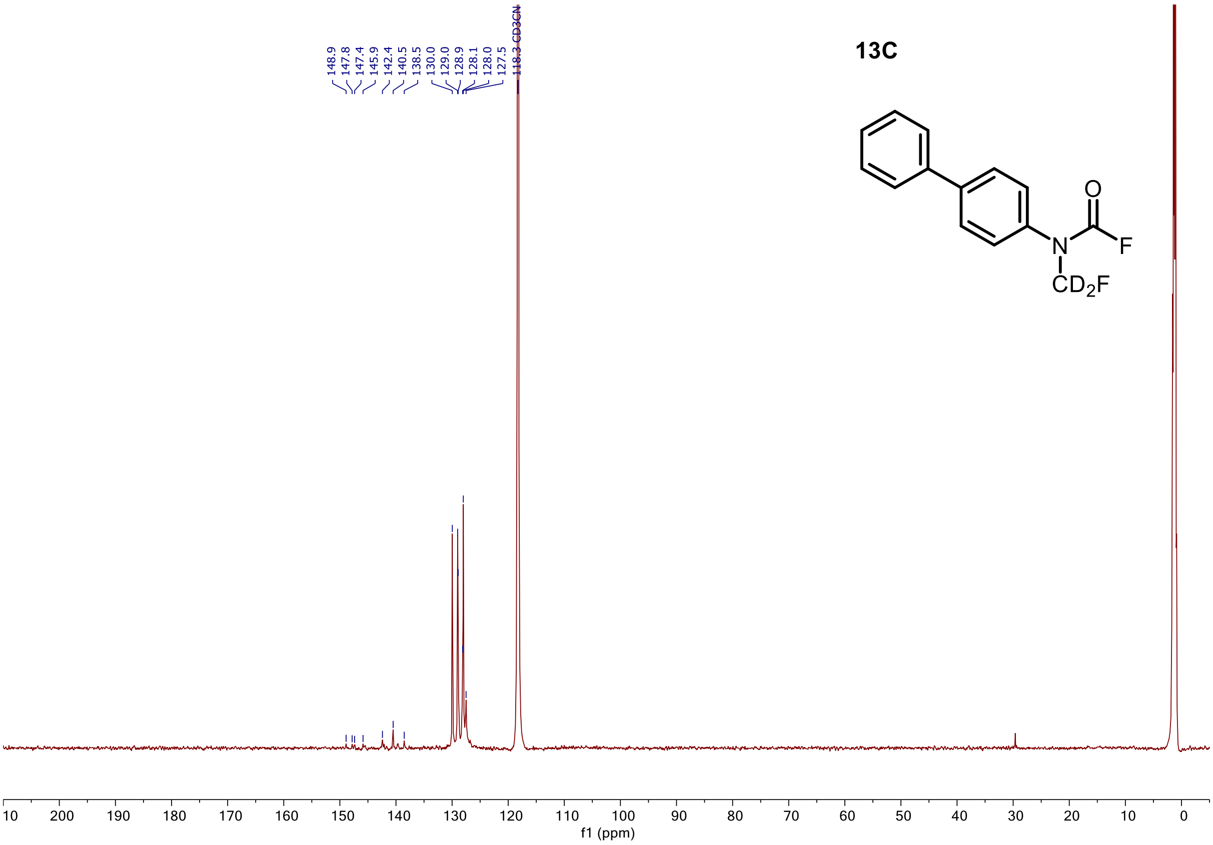
148.9
147.8
147.4
145.9
142.4
140.5
138.5
130.0
129.0
128.9
128.1
128.0
127.5
118.3 CD3CN
13C
O
F
N
CD2F
10 200 190 180 170 160 150 140 130 120 110 100 90 80 70 60 50 40 30 20 10 0
f1 (ppm)

(Fluoro(phenyl)methyl)(phenyl)carbamic fluoride (16)

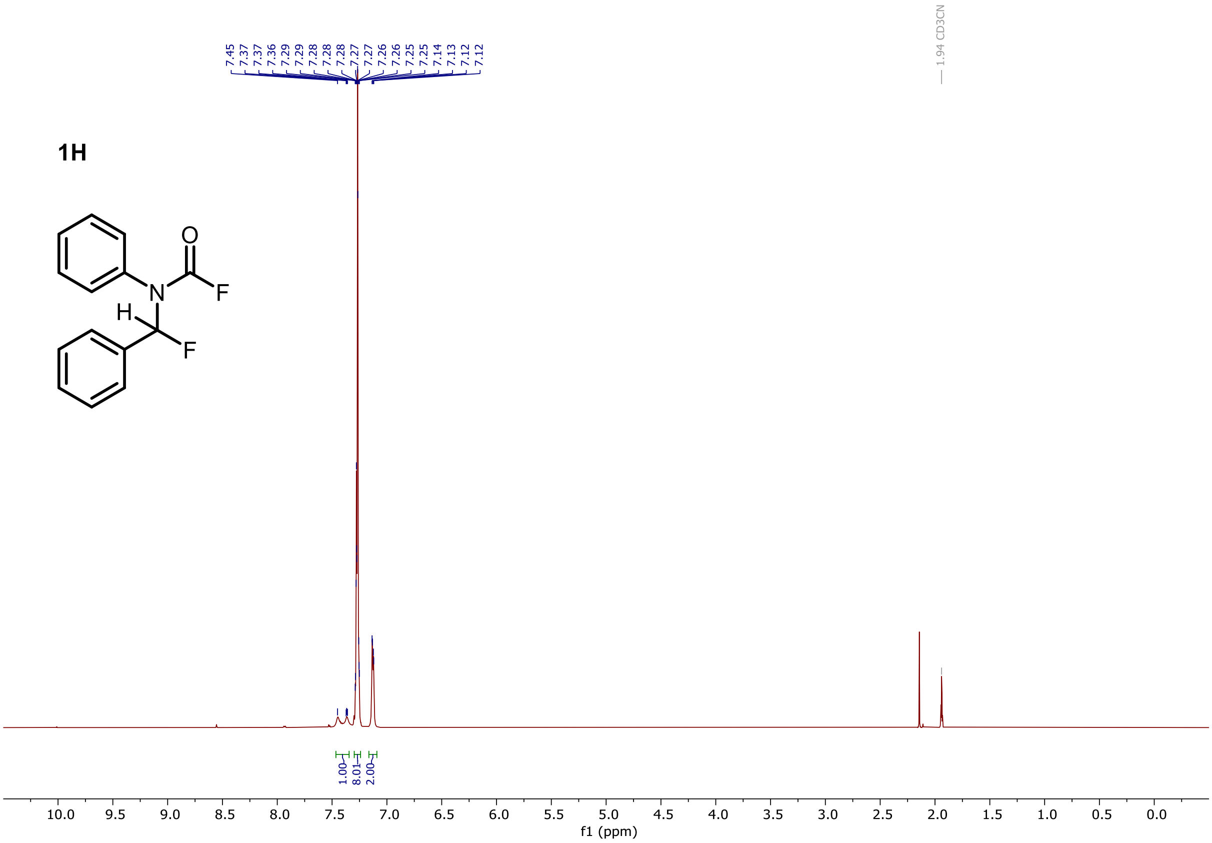

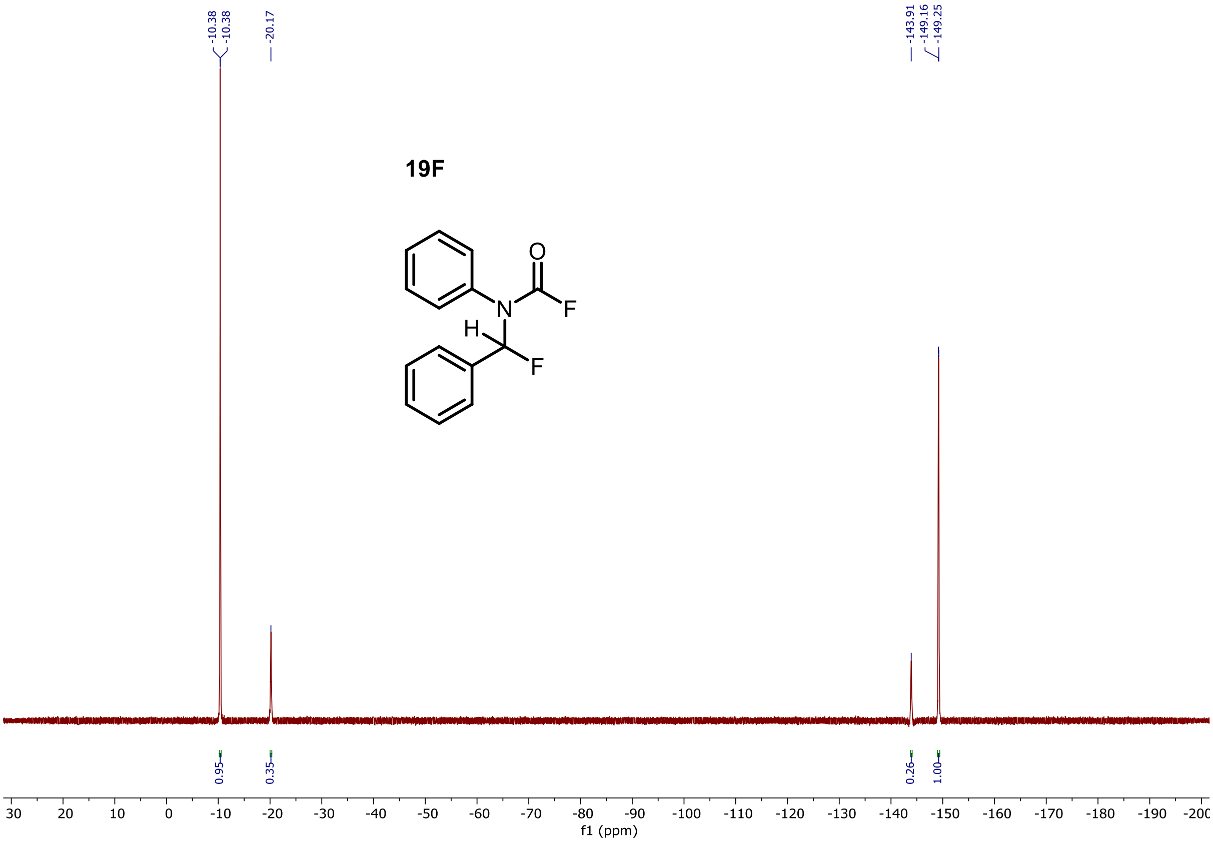

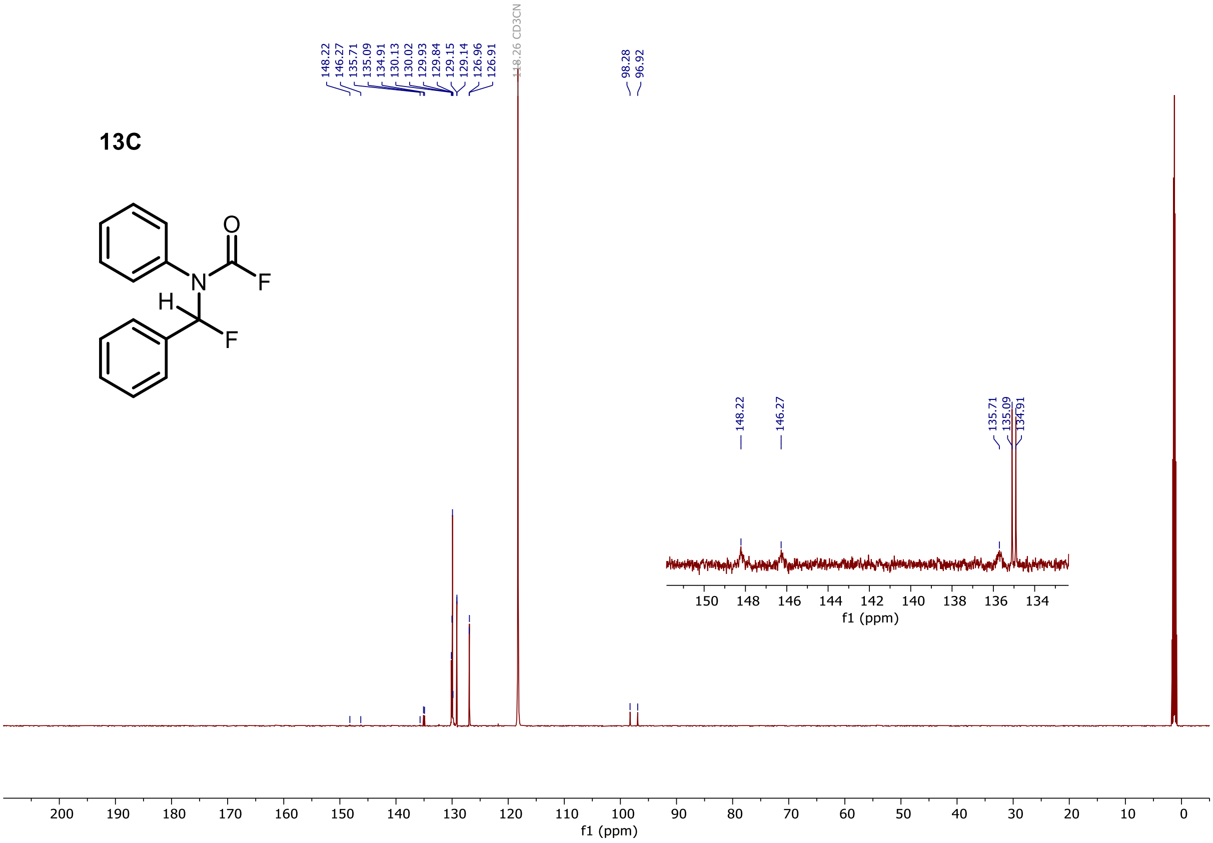

(Fluoro(phenyl)methyl)(4-(trifluoromethyl)phenyl)carbamic fluoride (17)

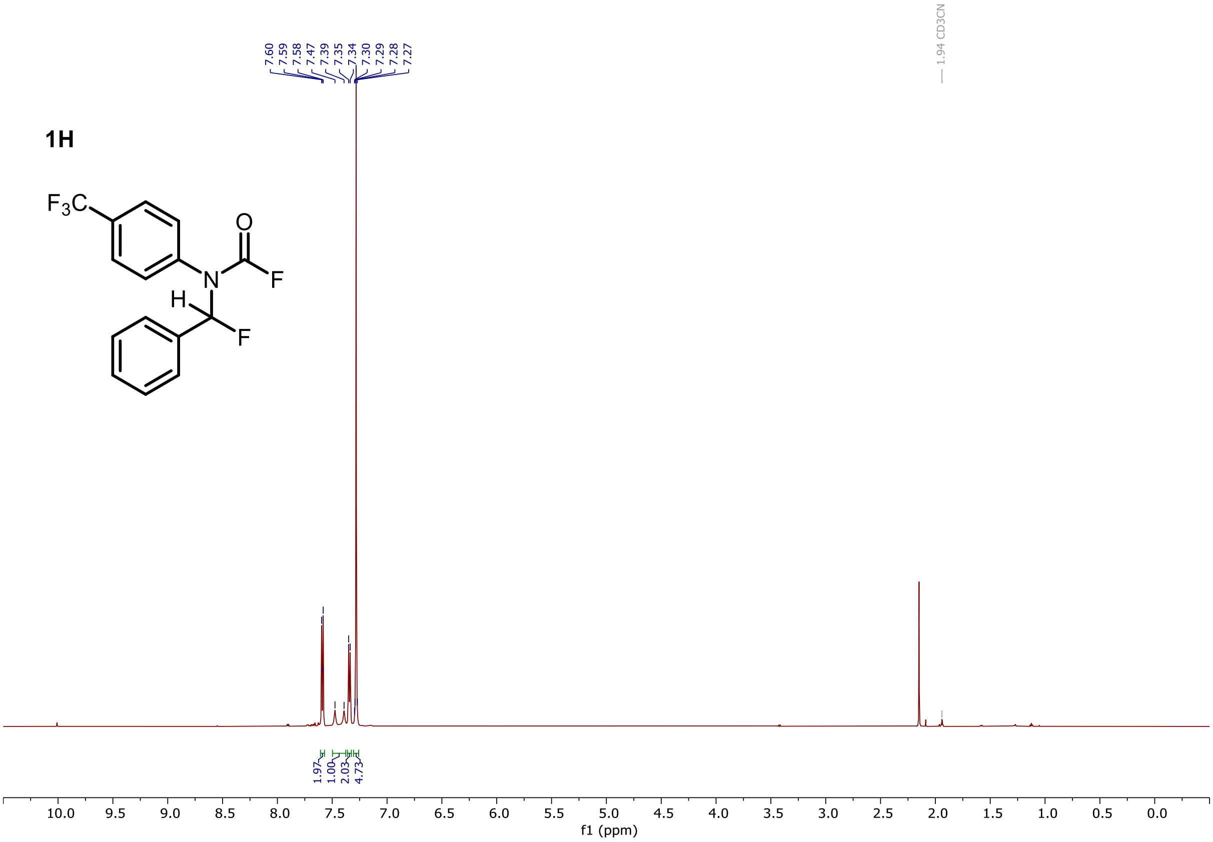

19F

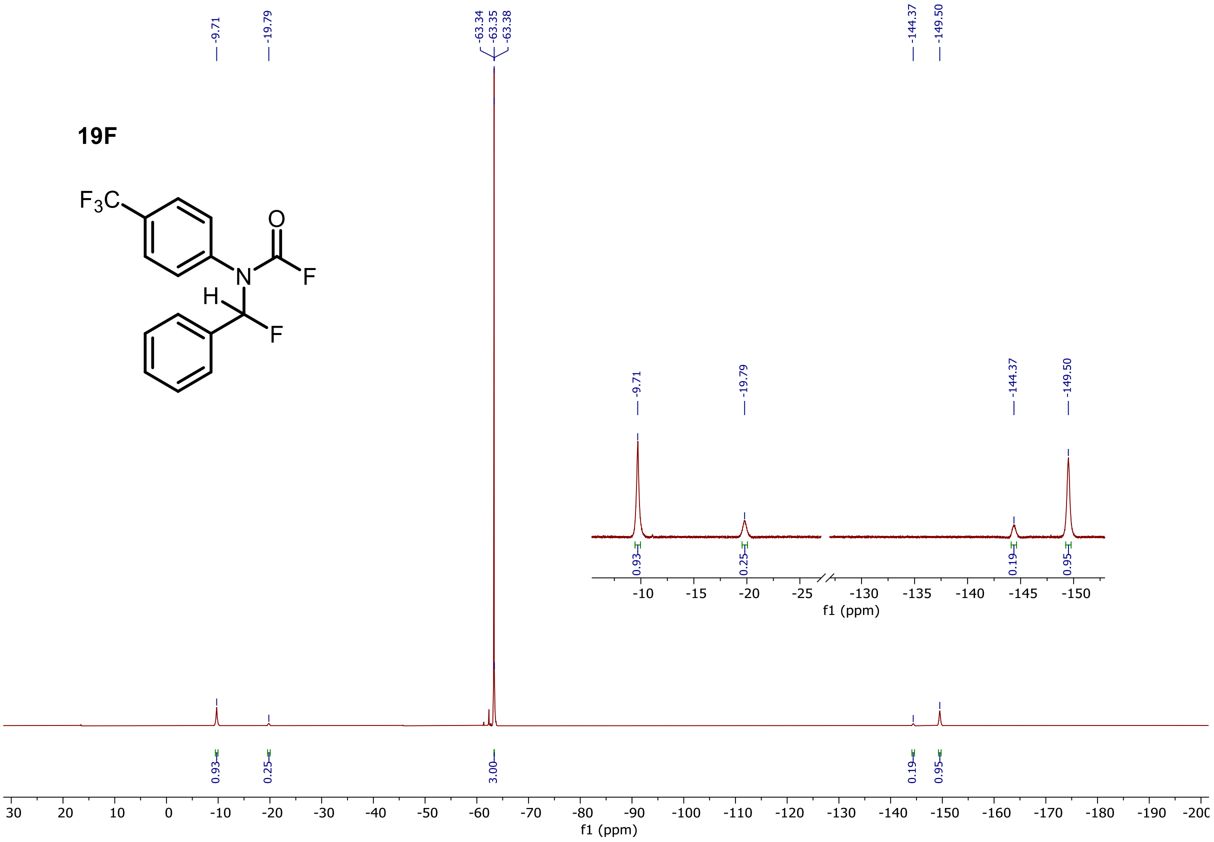

-9.71
-19.79
-63.34
-63.35
-63.38
-144.37
-149.50
F_3C
O
N
F
H
F
0.93
0.25
3.00
0.19
0.95
f1 (ppm)

13C

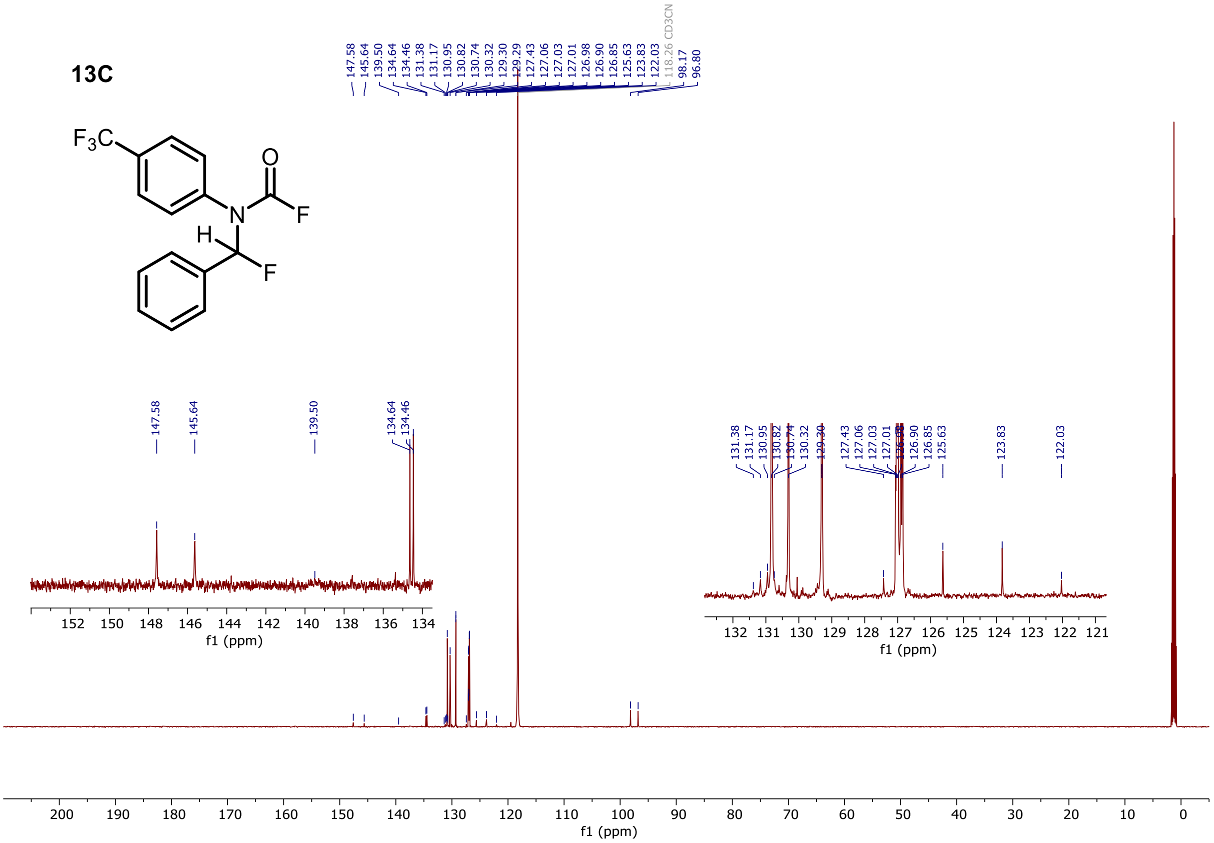

147.58
145.64
139.50
134.64
134.46
131.38
131.17
130.95
130.82
130.74
130.32
129.30
129.29
127.43
127.06
127.03
127.01
126.98
126.90
126.85
125.63
123.83
122.03
118.26 CD3CN
98.17
96.80
F_3C
O
N
F
H
F
f1 (ppm)

Methyl 4-((fluoro(*o*-tolyl)methyl)(fluorocarbonyl)amino)benzoate (18)

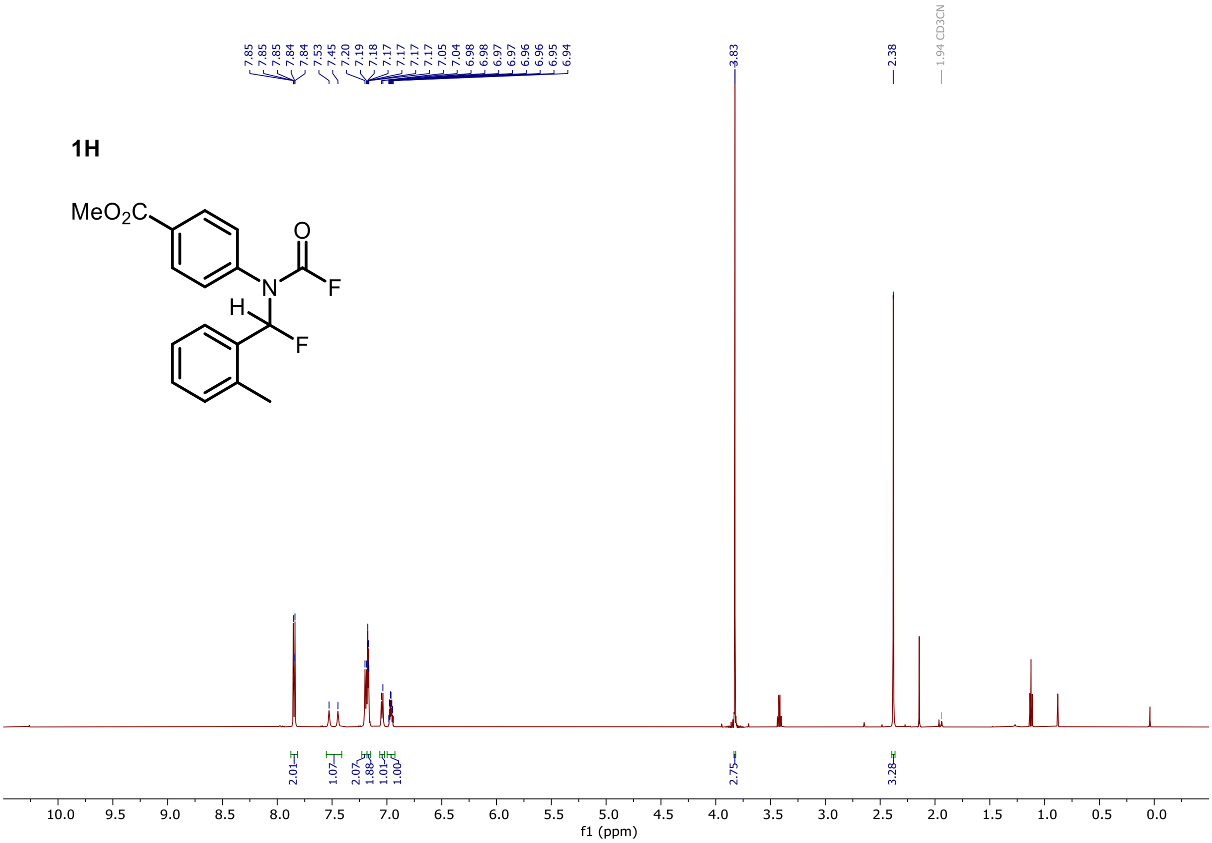

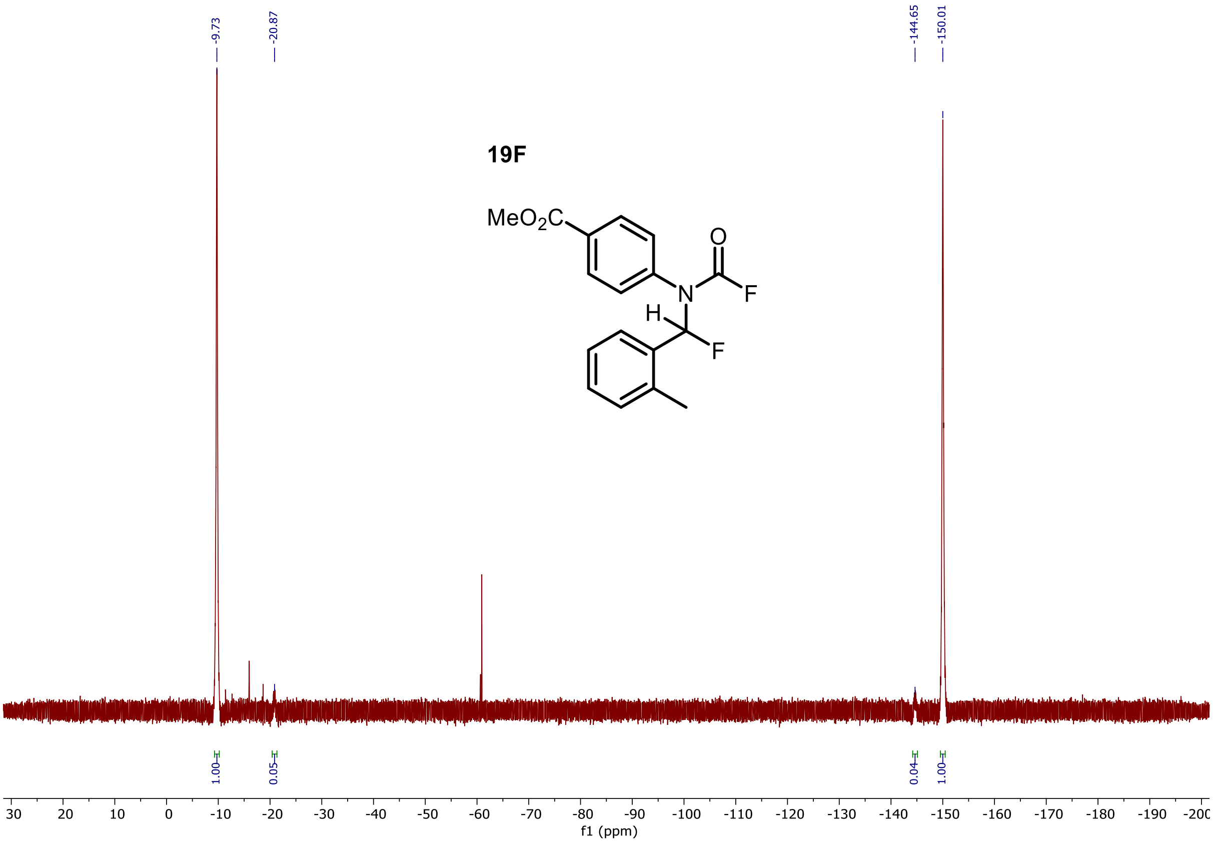

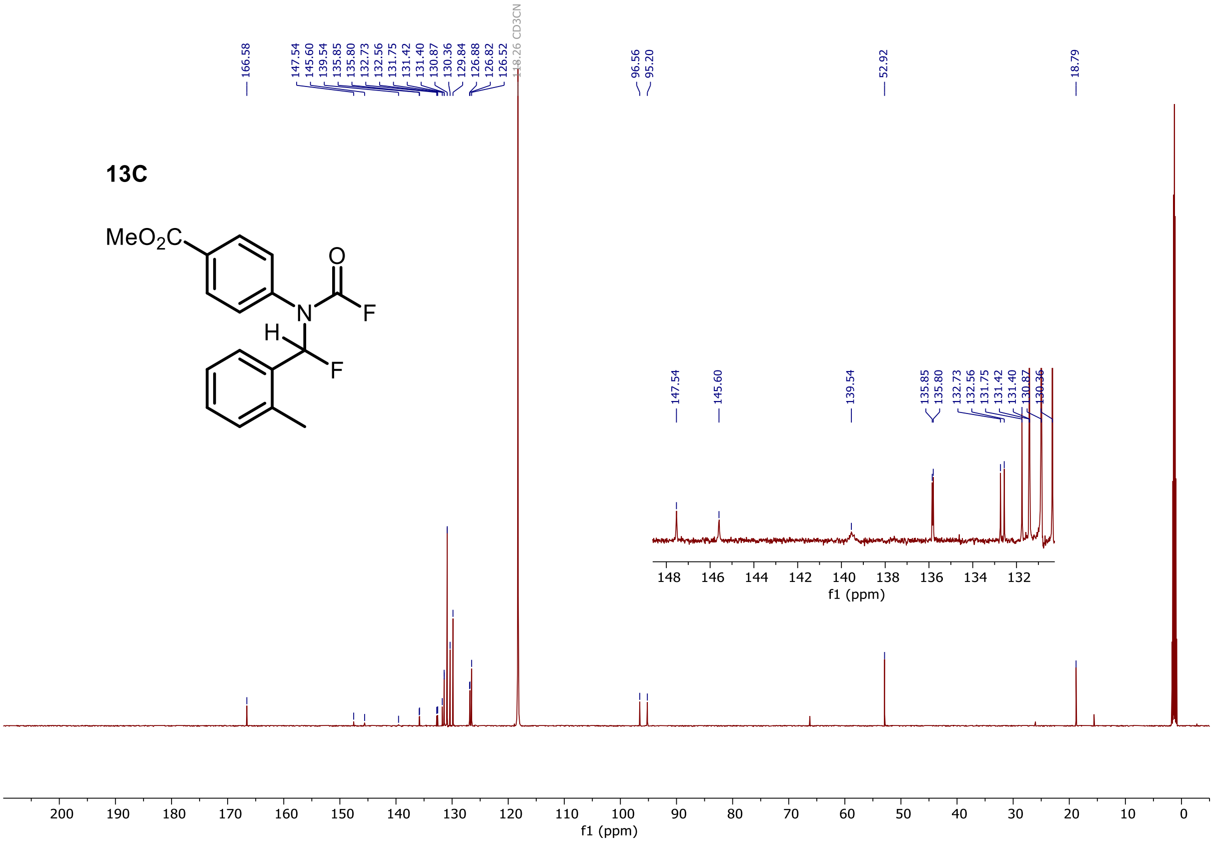

Methyl 4-(((2-bromo-5-chlorophenyl)fluoromethyl)(fluorocarbonyl)amino)thiophene-2-carboxylate (19)

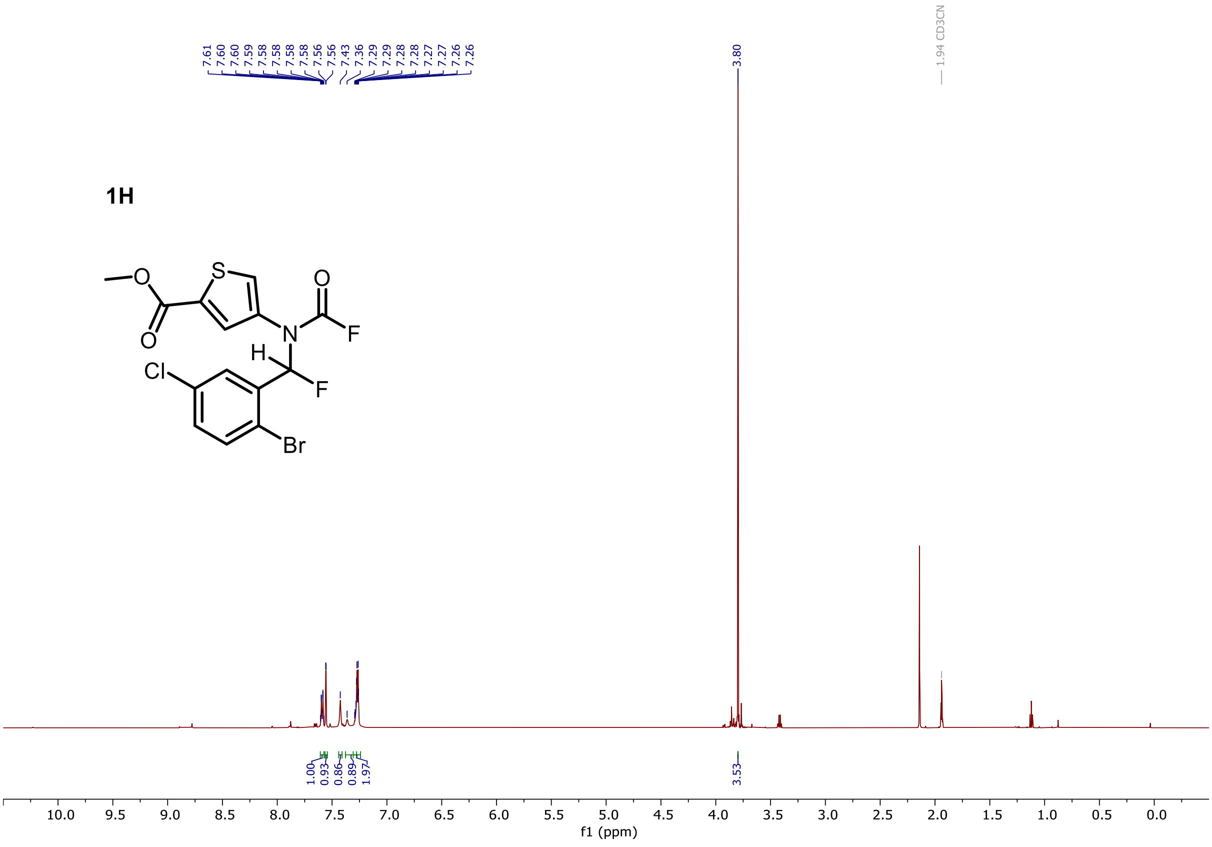

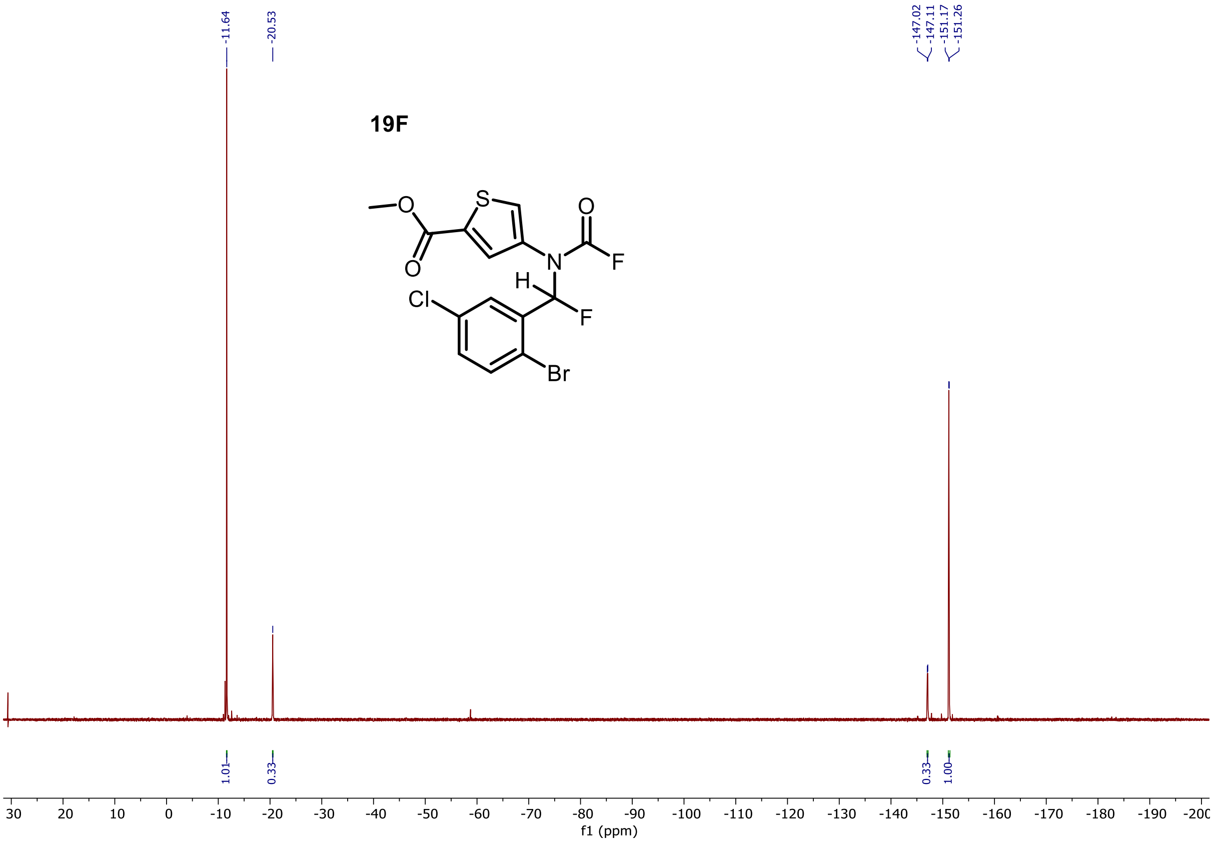

19F
-11.64
-20.53
-147.02
-147.11
-151.17
-151.26
1.01
0.33
0.33
1.00
30 20 10 0 -10 -20 -30 -40 -50 -60 -70 -80 -90 -100 -110 -120 -130 -140 -150 -160 -170 -180 -190 -200
f1 (ppm)

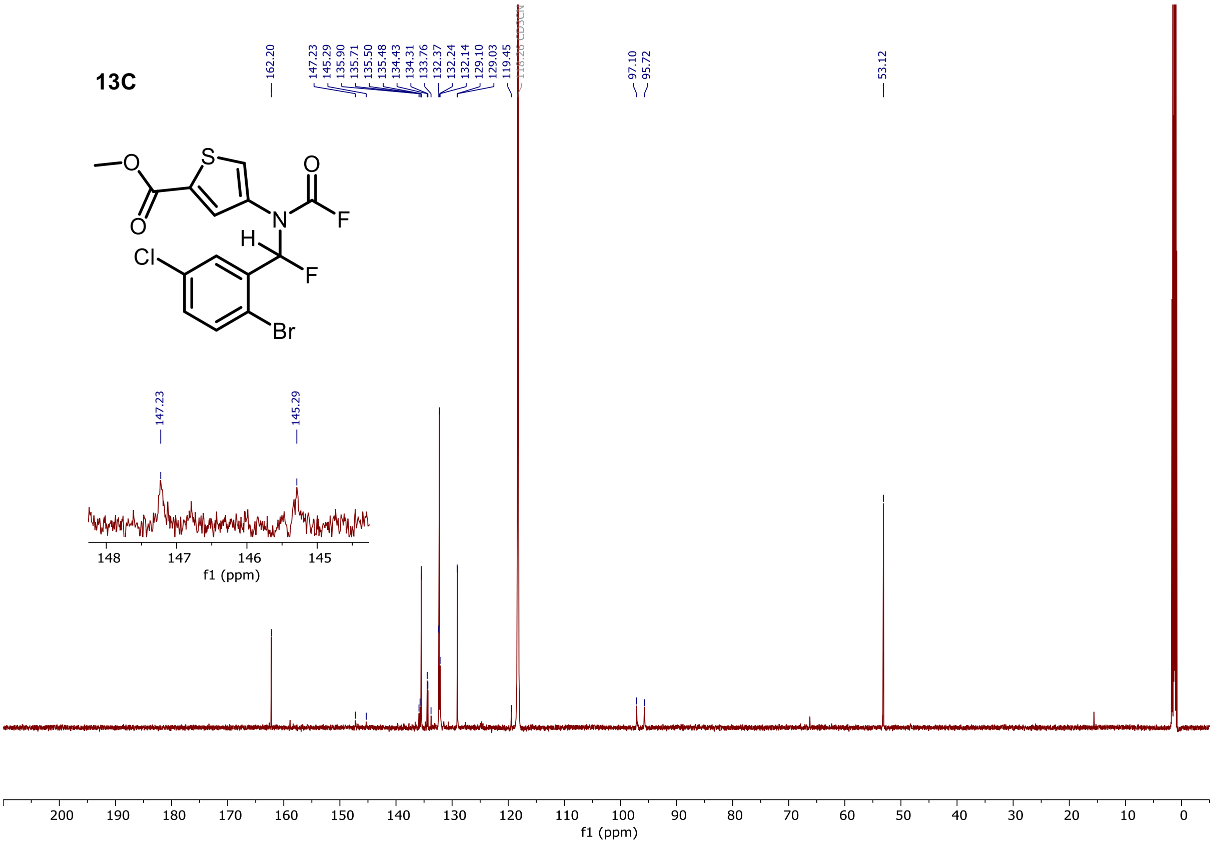

13C
162.20
147.23
145.29
135.90
135.71
135.50
135.48
134.43
134.31
133.76
132.37
132.24
132.14
129.10
129.03
119.45
118.26 CD3CN
97.10
95.72
53.12
148 147 146 145
f1 (ppm)
200 190 180 170 160 150 140 130 120 110 100 90 80 70 60 50 40 30 20 10 0
f1 (ppm)

(1-Fluoro-2-methylbutyl)(4-methoxyphenyl)carbamic fluoride (20)

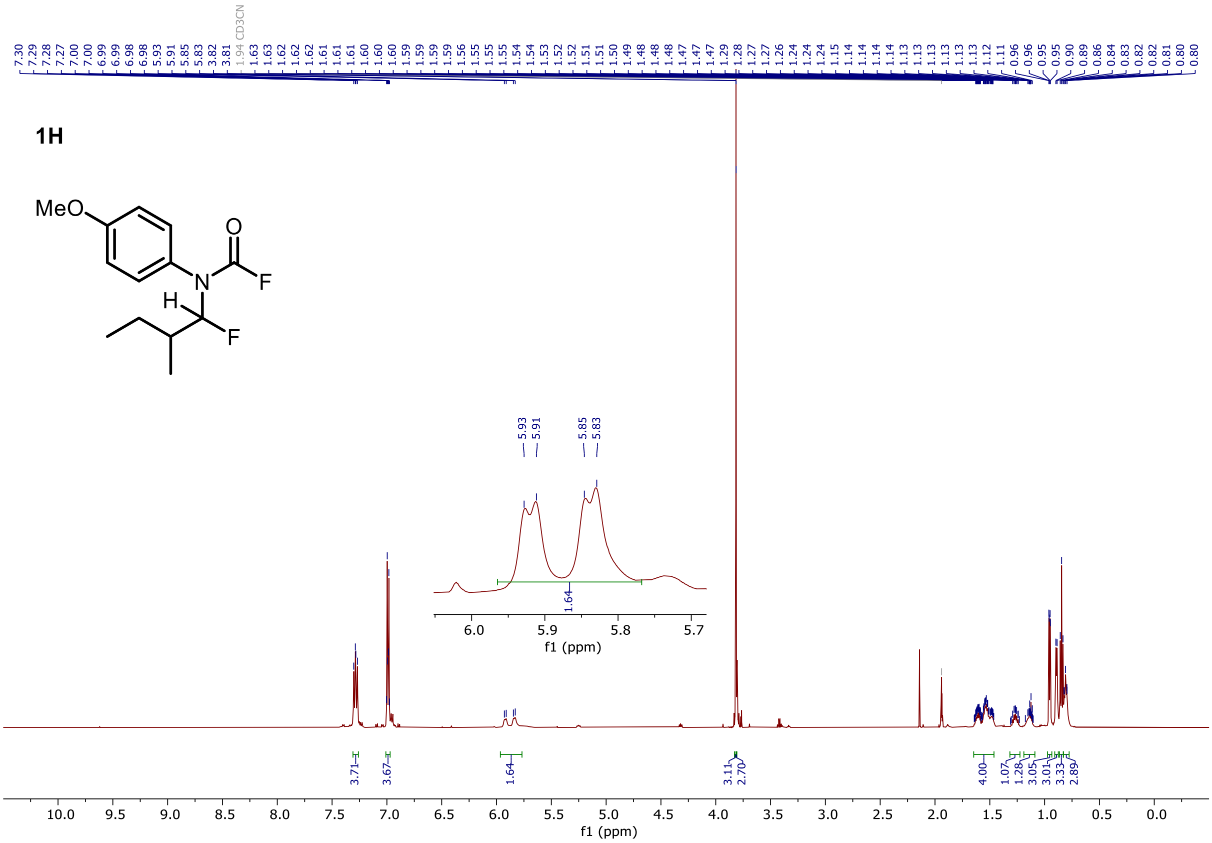

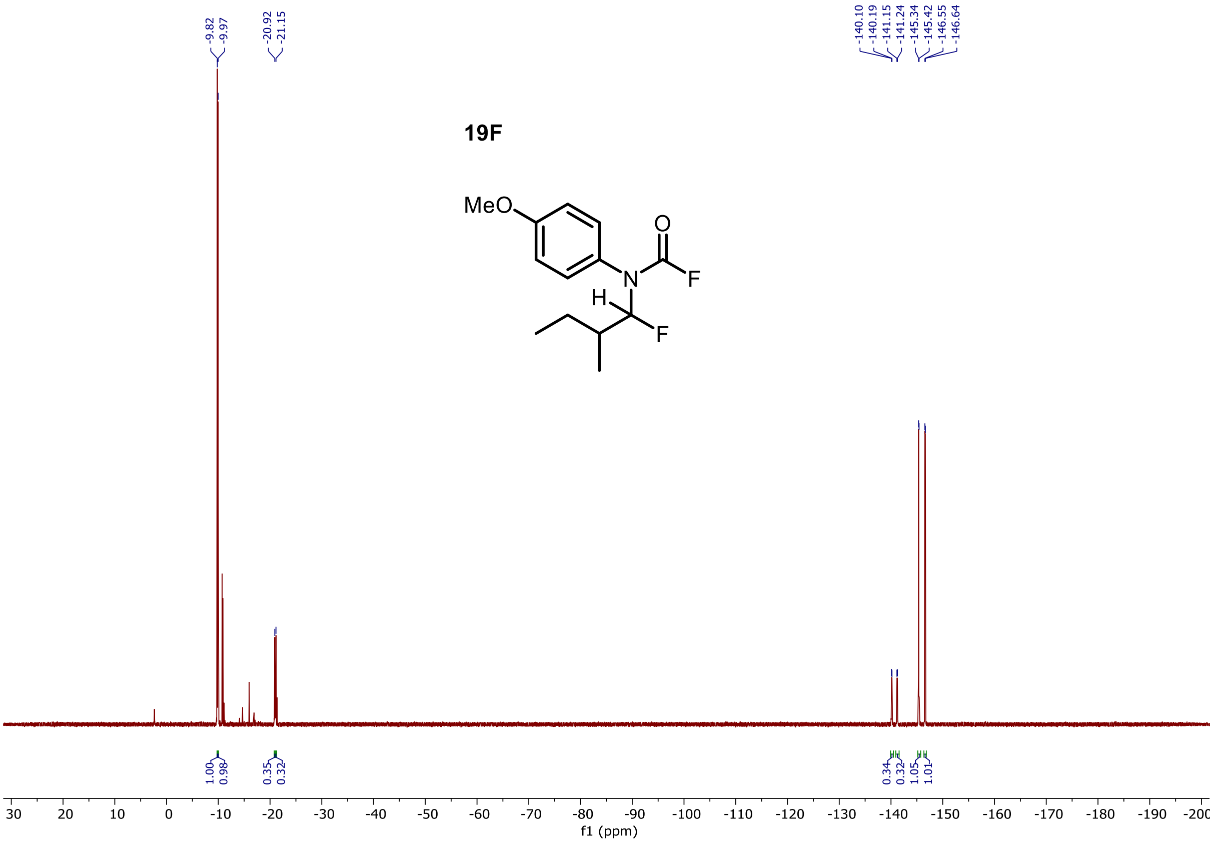

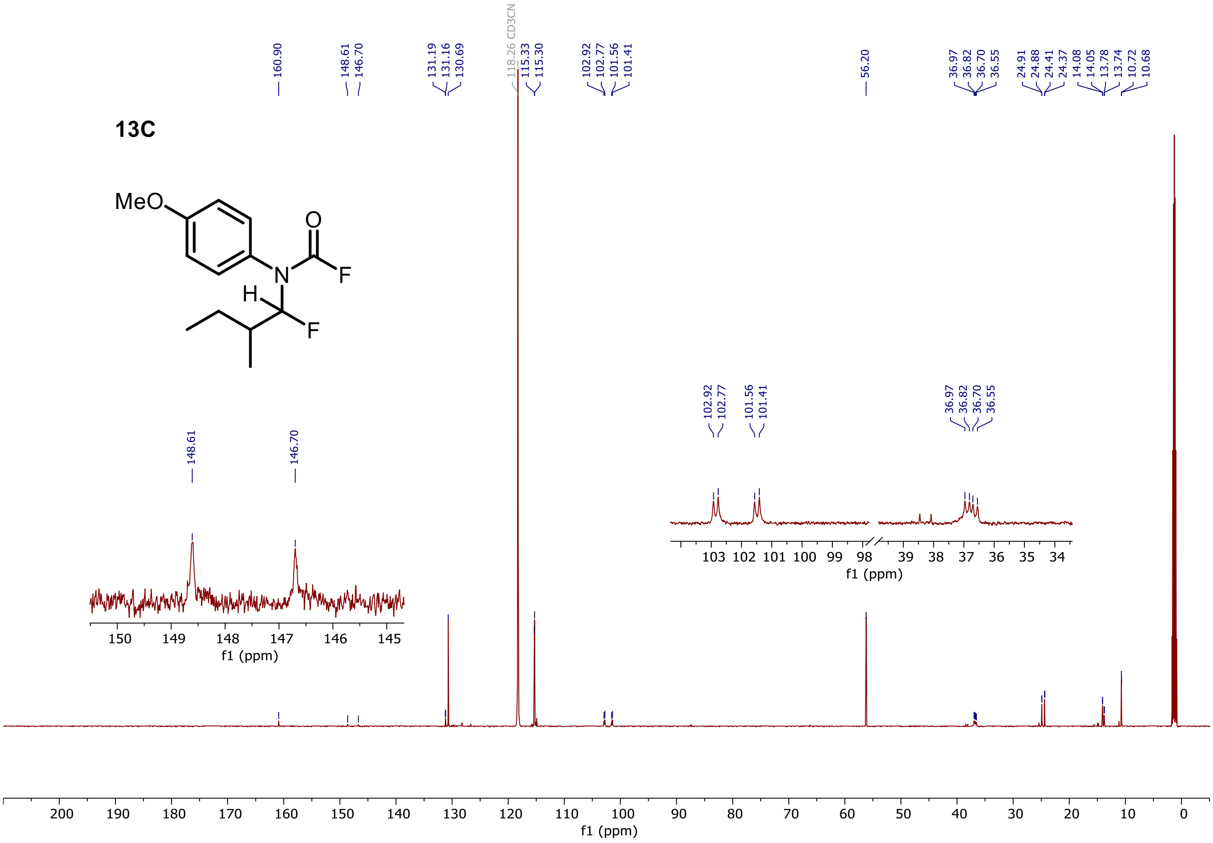

(9-Ethyl-9*H*-carbazol-3-yl)(1-fluoro-2,2-dimethylpent-4-en-1-yl)carbamic fluoride (21)

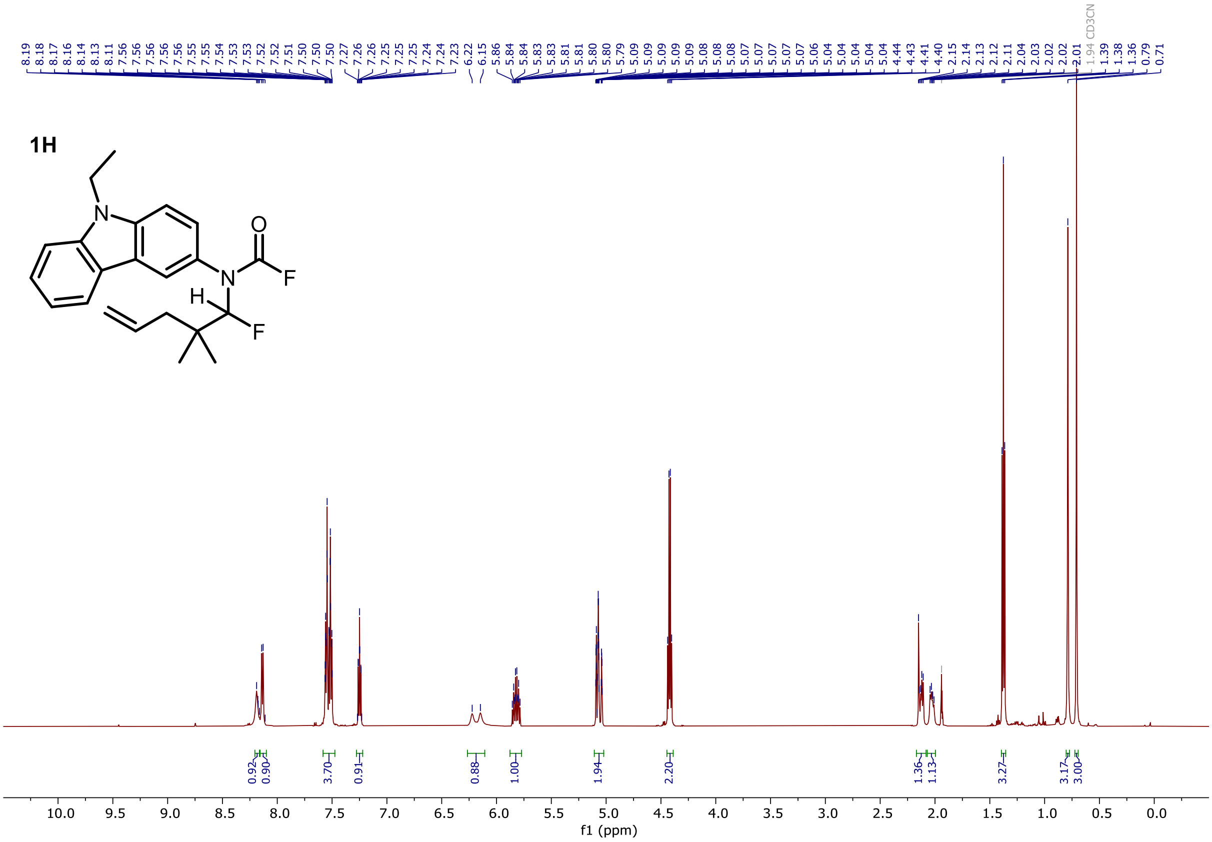

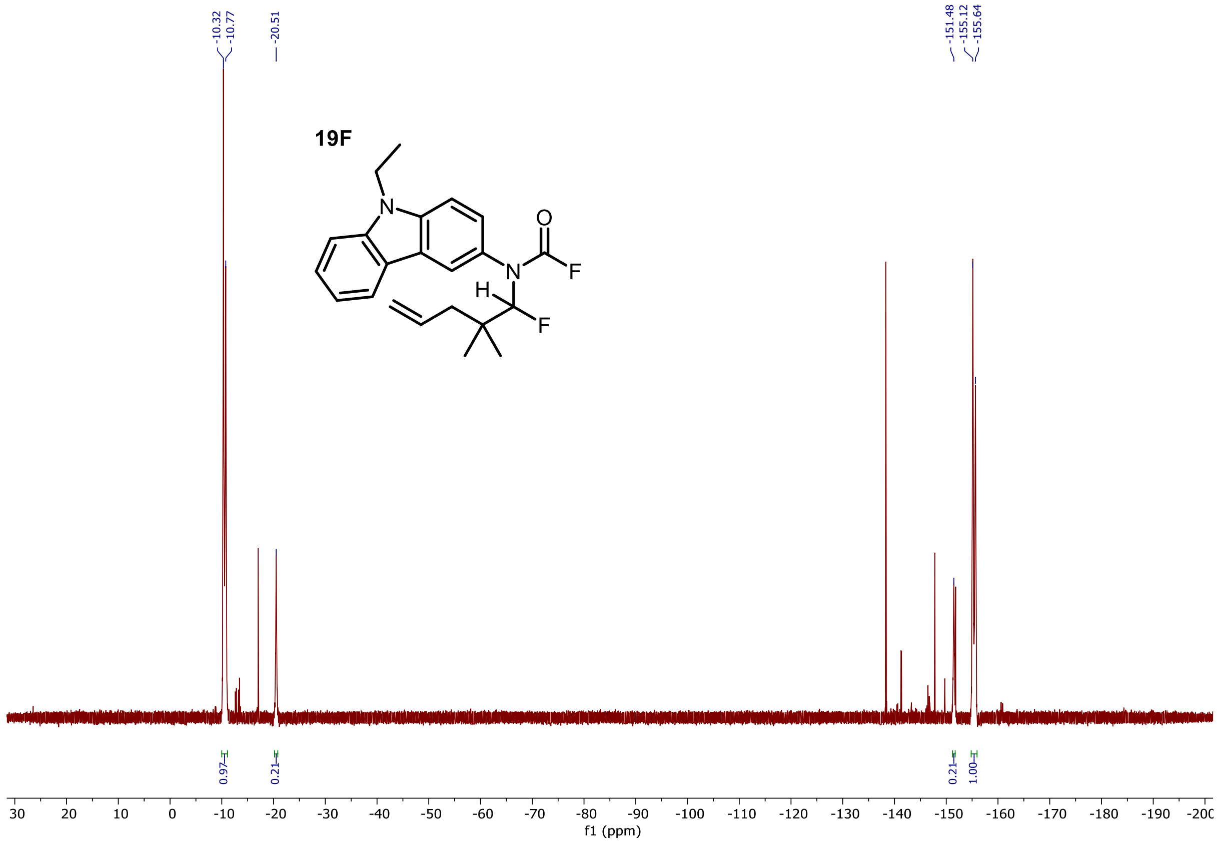
19F
-10.32
-10.77
-20.51
-151.48
-155.12
-155.64
0.97
0.21
0.21
1.00
f1 (ppm)

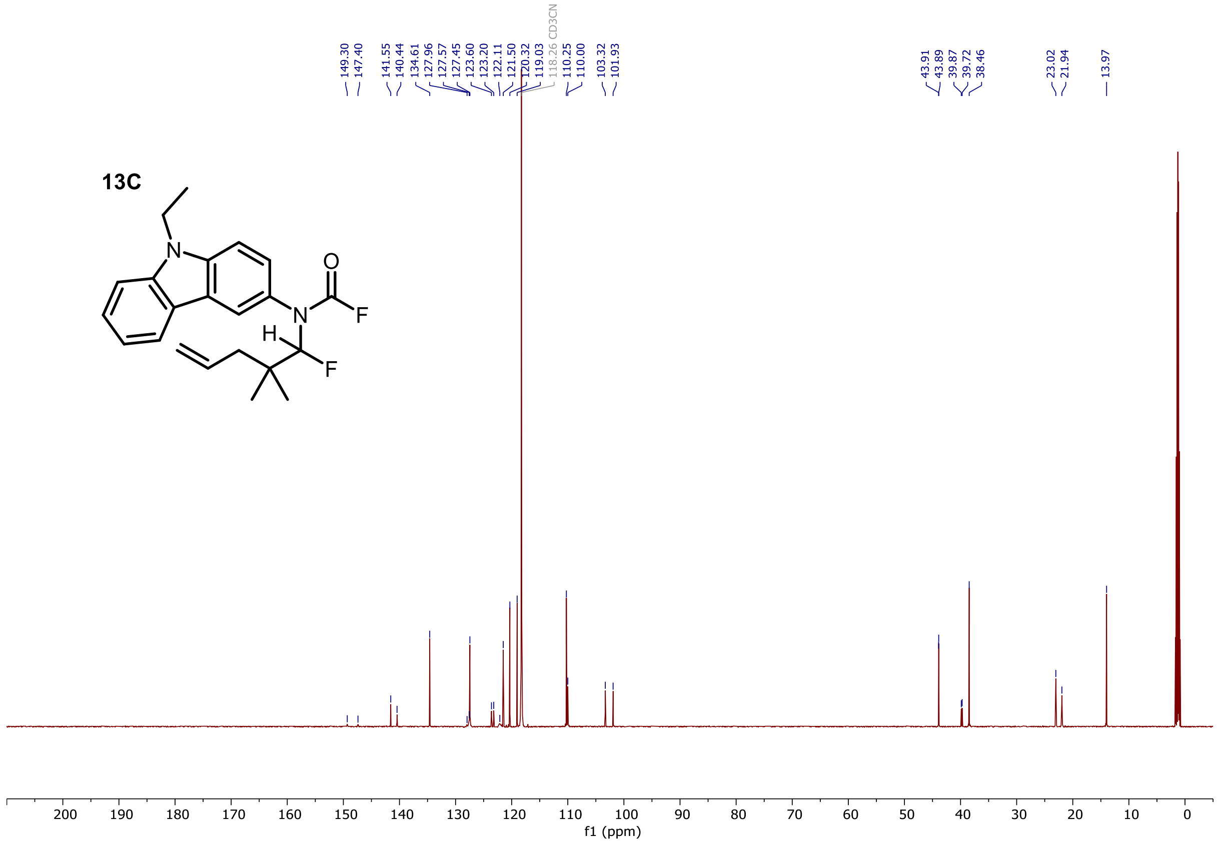
13C
149.30
147.40
141.55
140.44
134.61
127.96
127.57
127.45
123.60
123.20
122.11
121.50
120.32
119.03
118.26 CD3CN
110.25
110.00
103.32
101.93
43.91
43.89
39.87
39.72
38.46
23.02
21.94
13.97
f1 (ppm)

(Fluoro(phenyl)methyl)(furan-2-ylmethyl)carbamic fluoride (22)

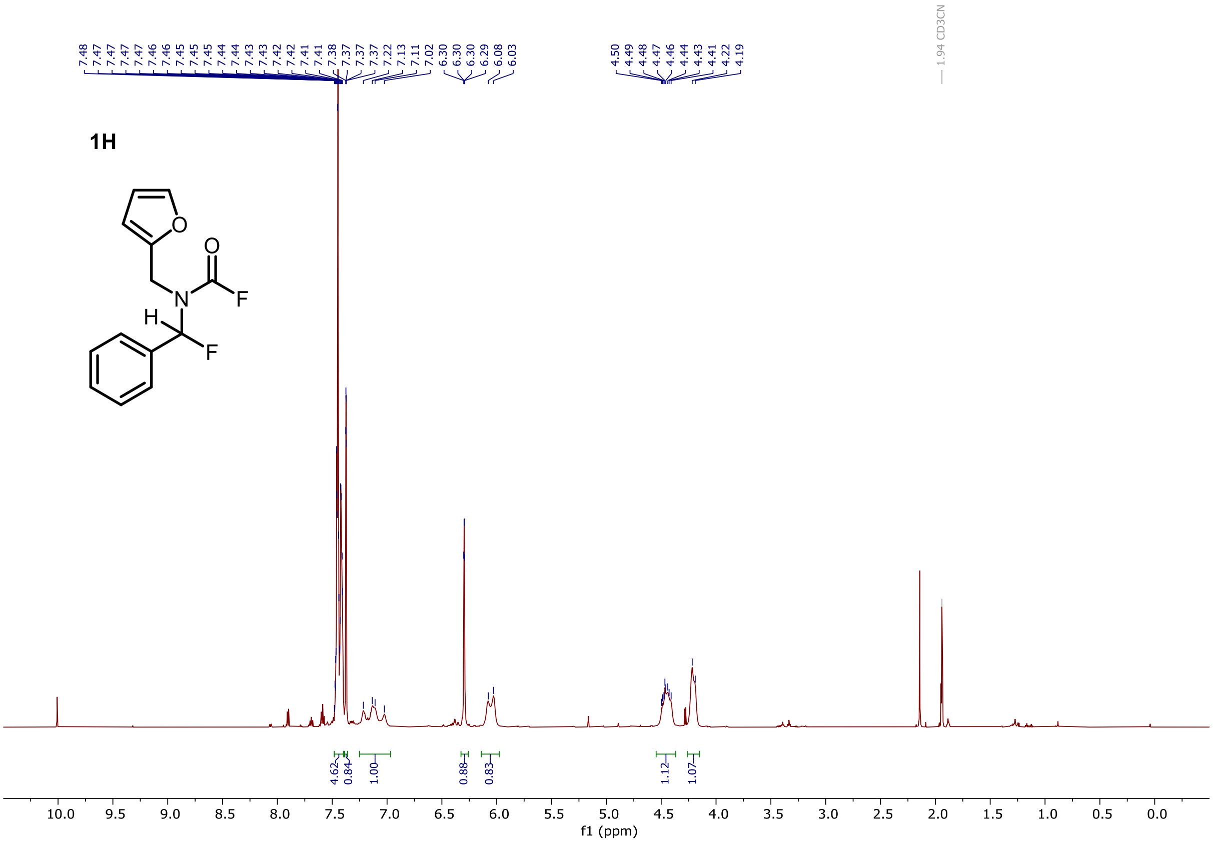

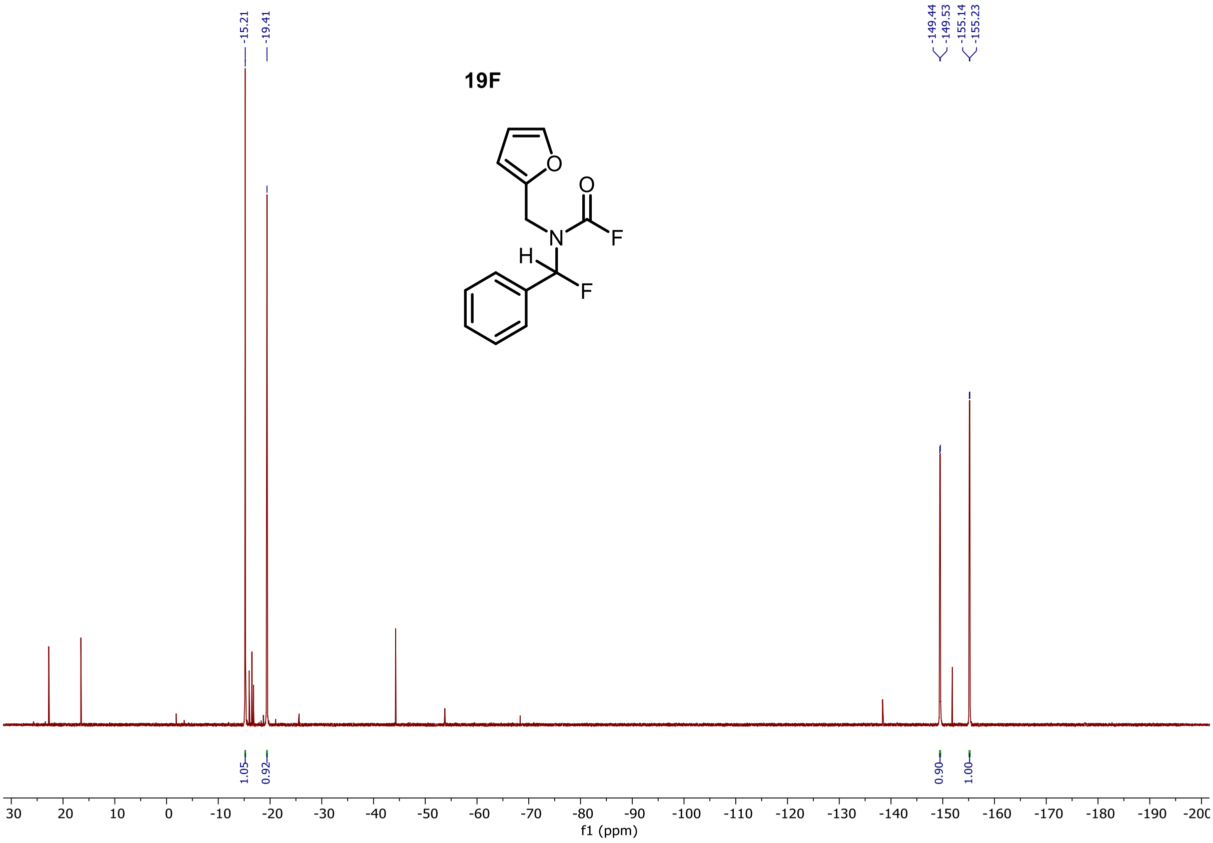

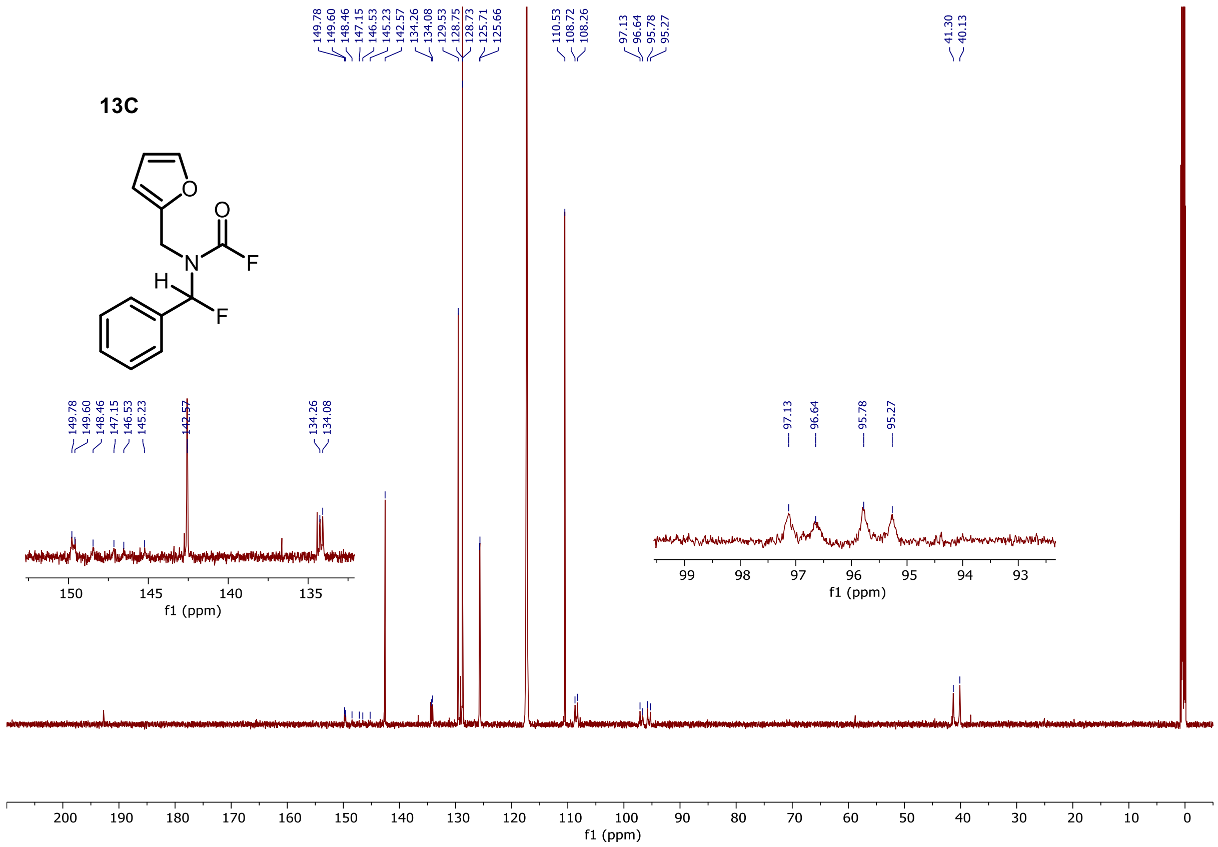

Allyl(fluoro(phenyl)methyl)carbamic fluoride (23)

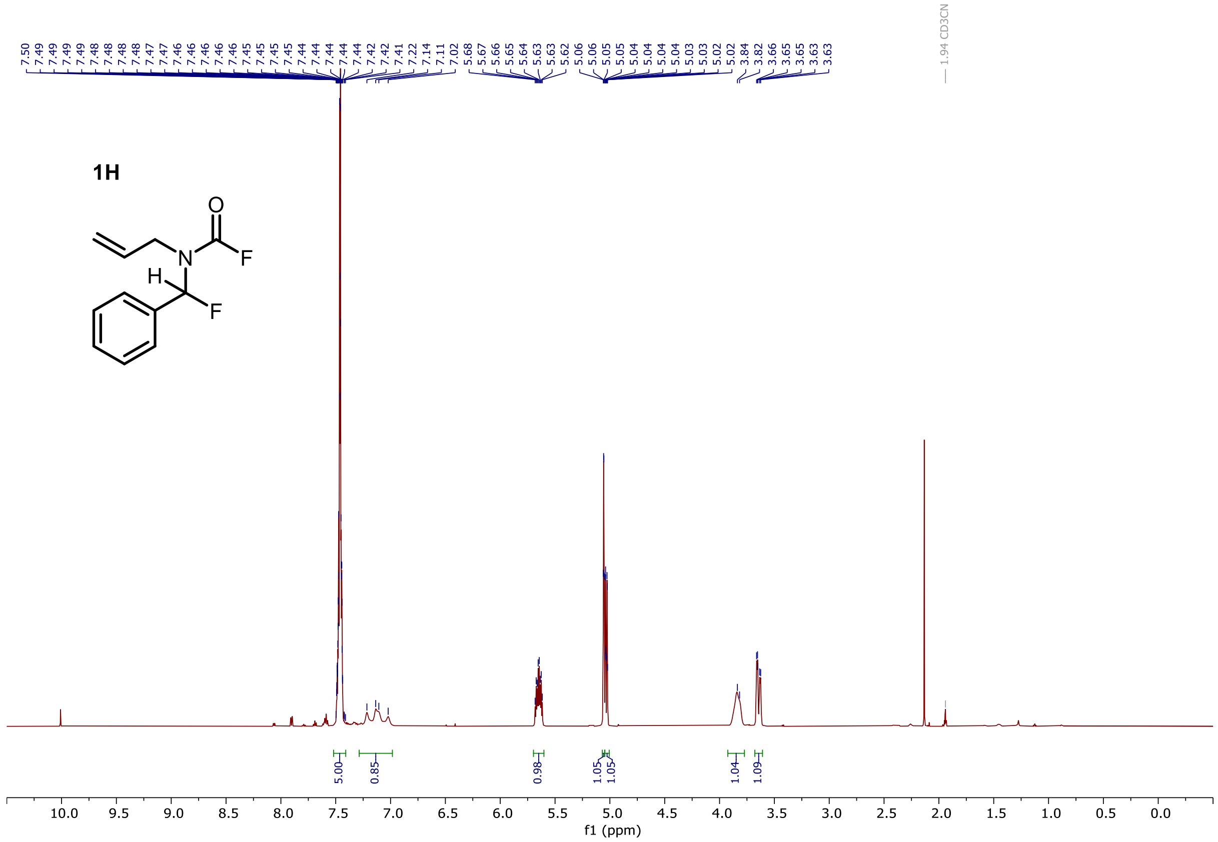

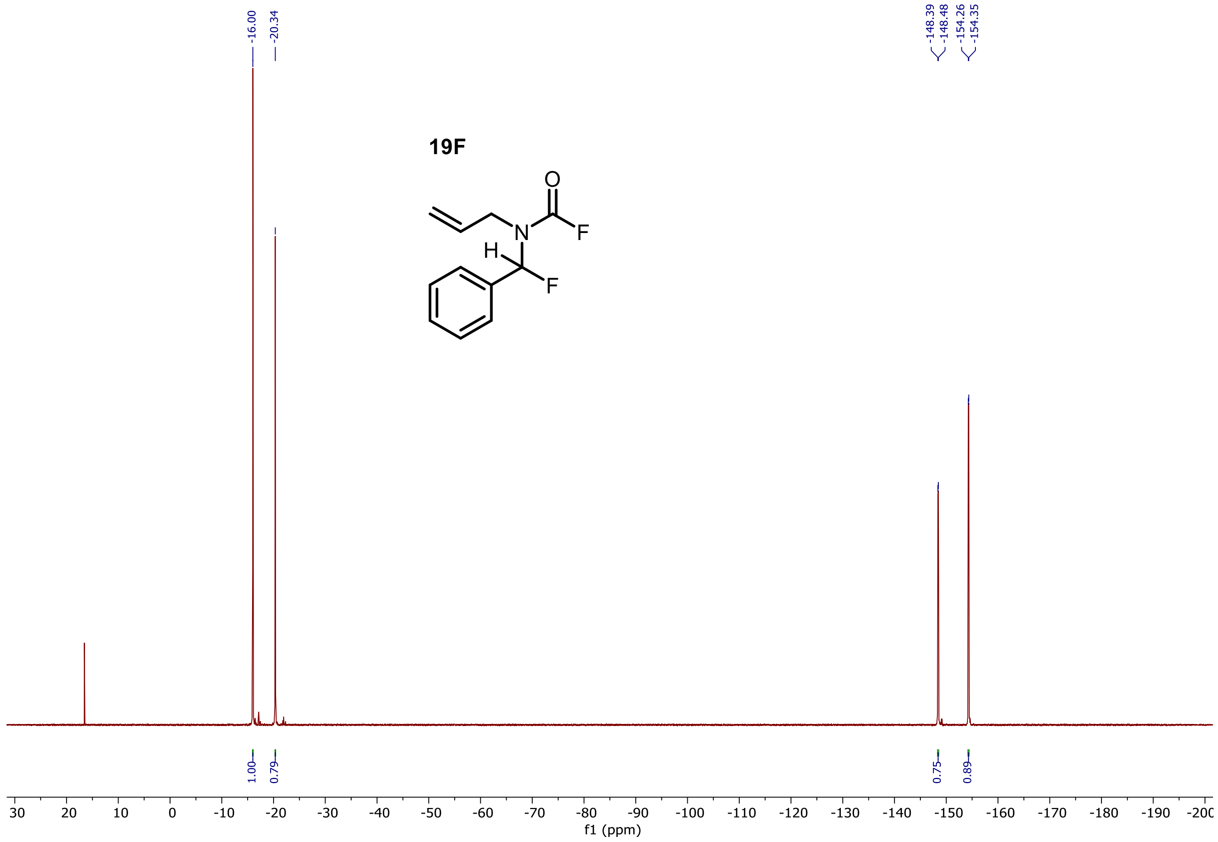
19F
O
N
F
H
F
-16.00
-20.34
-148.39
-148.48
-154.26
-154.35
1.00
0.79
0.75
0.89
f1 (ppm)

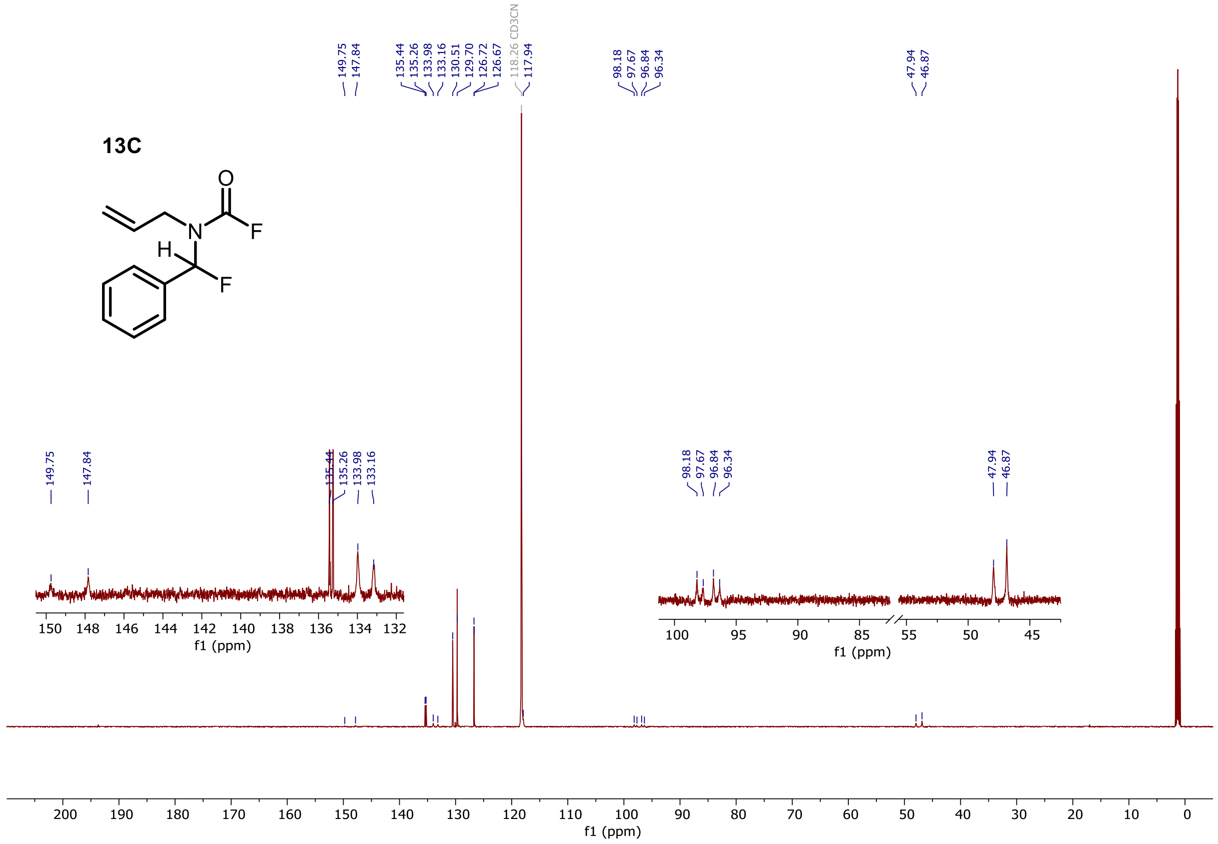
13C
O
N
F
H
F
149.75
147.84
135.44
135.26
133.98
133.16
130.51
129.70
126.72
126.67
118.26 CD3CN
117.94
98.18
97.67
96.84
96.34
47.94
46.87
f1 (ppm)

((4-Chlorophenyl)fluoromethyl)(cyclohexyl)carbamic fluoride (24)

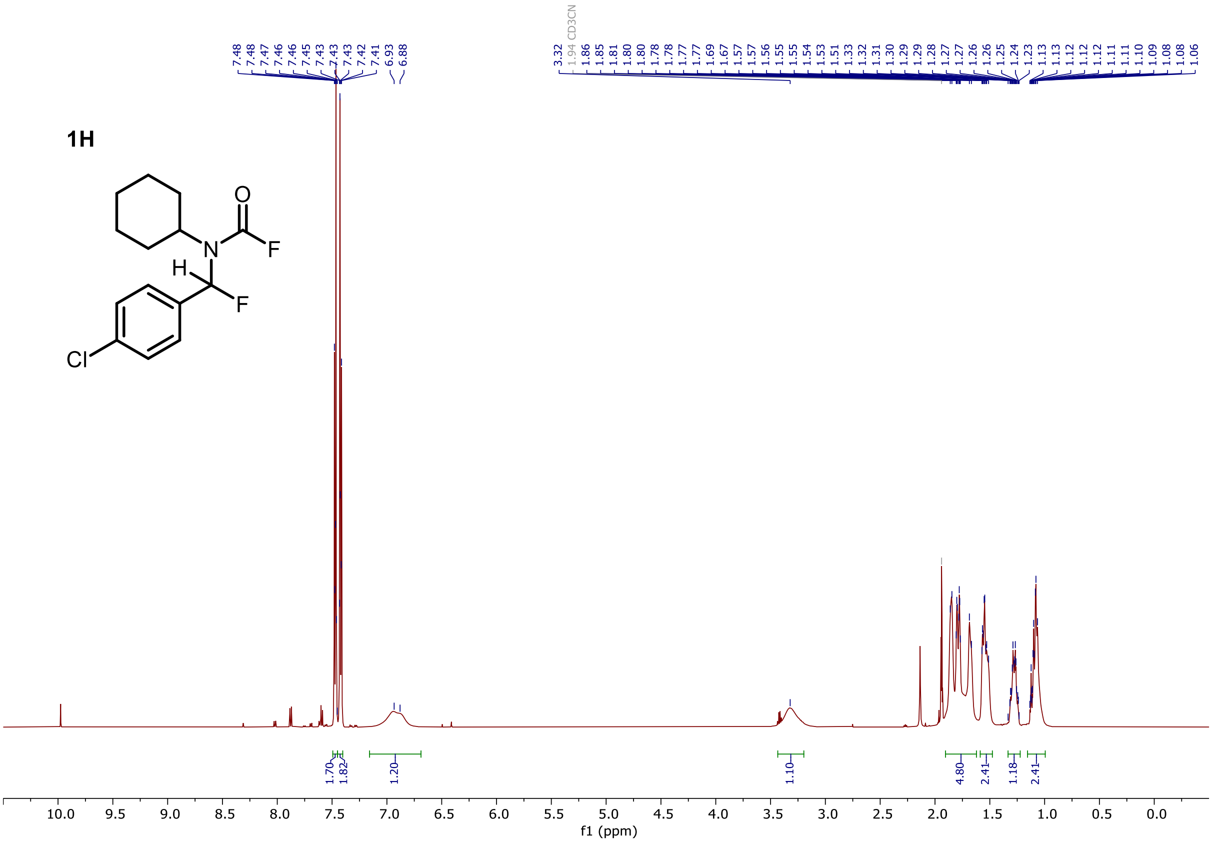

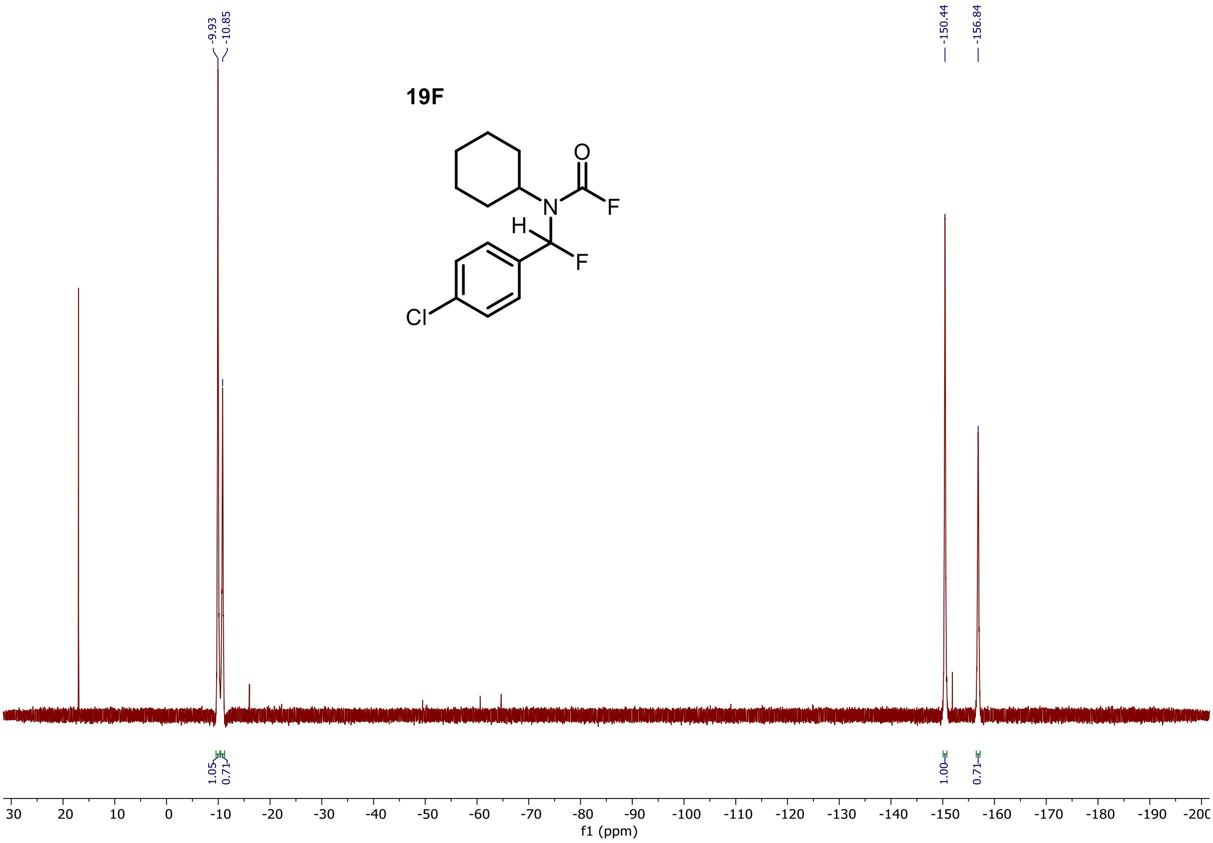

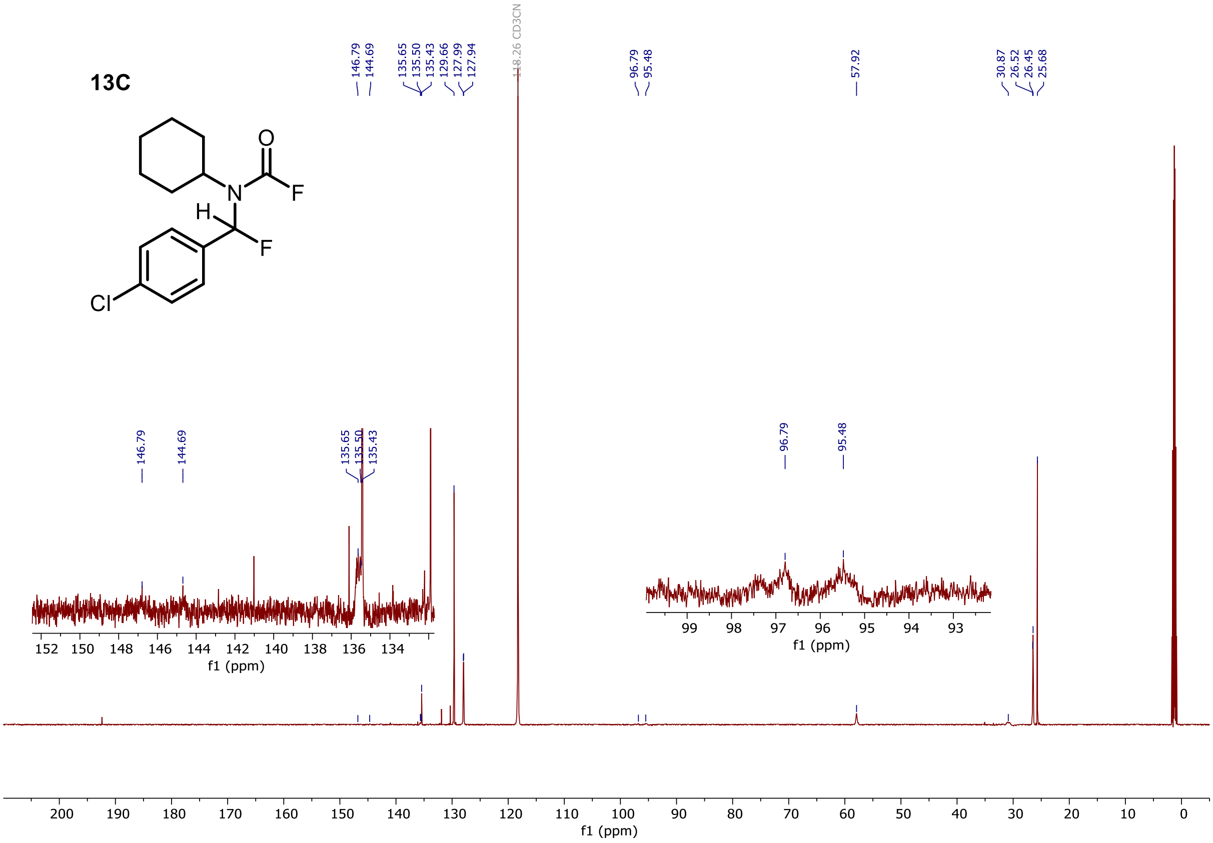

(2-Bromophenethyl)(1-fluoro-2,2-dimethylpropyl)carbamic fluoride (25)

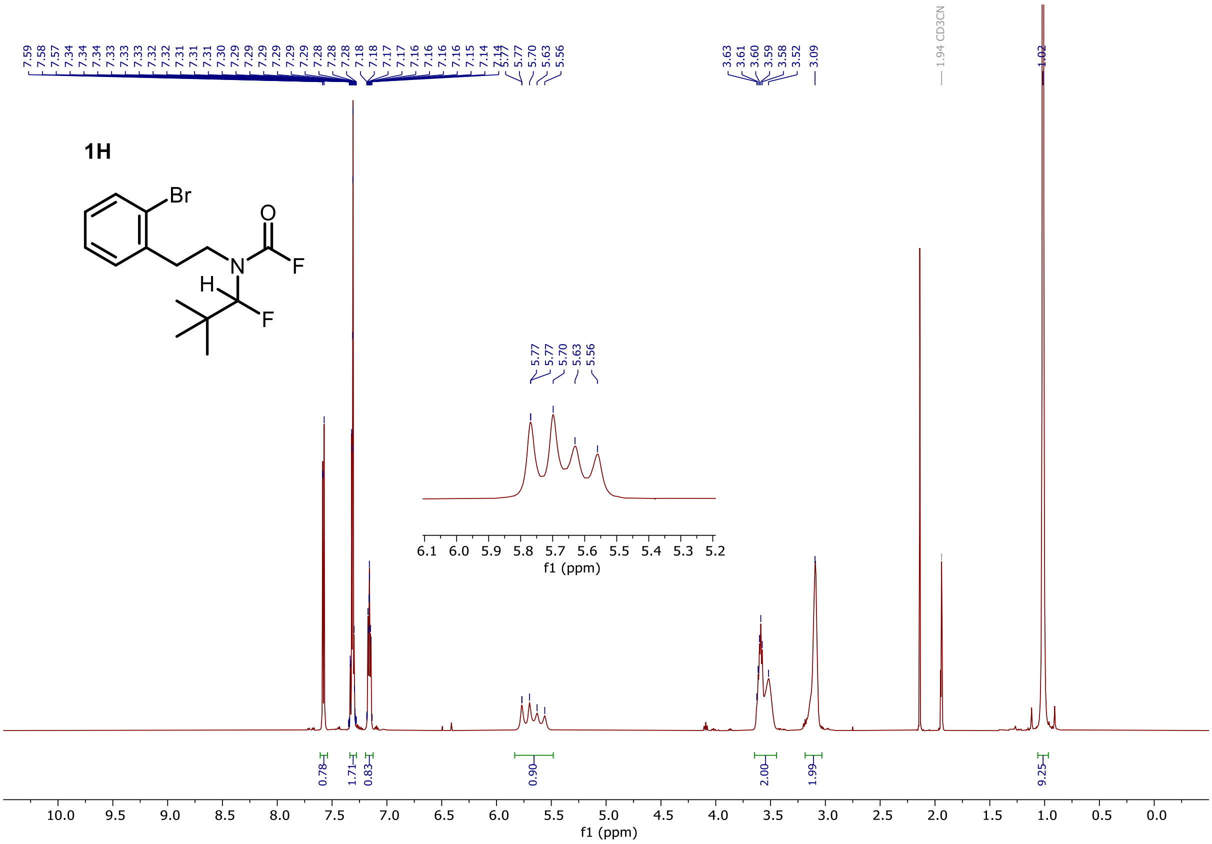

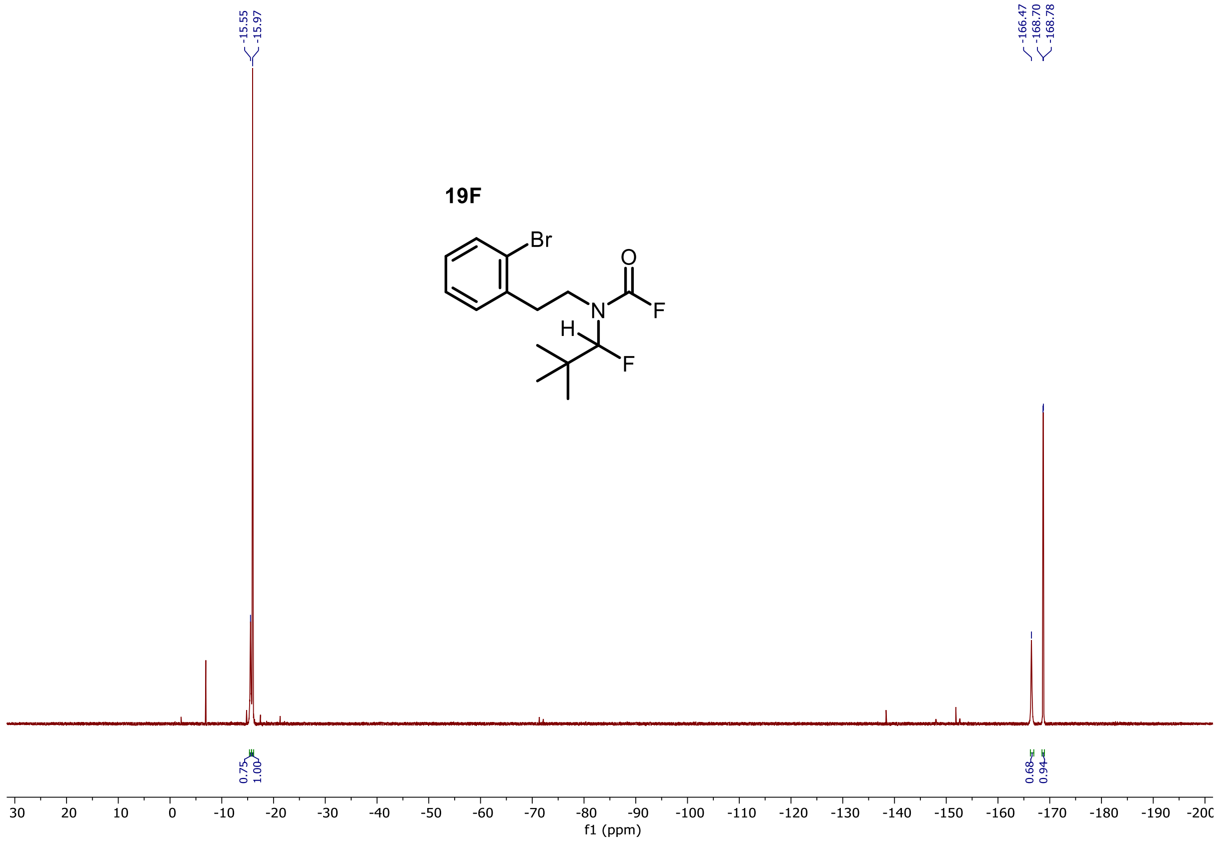

-15.55
-15.97
-166.47
-168.70
-168.78
19F
Br
O
N
F
H
F
0.75
1.00
0.68
0.94
f1 (ppm)

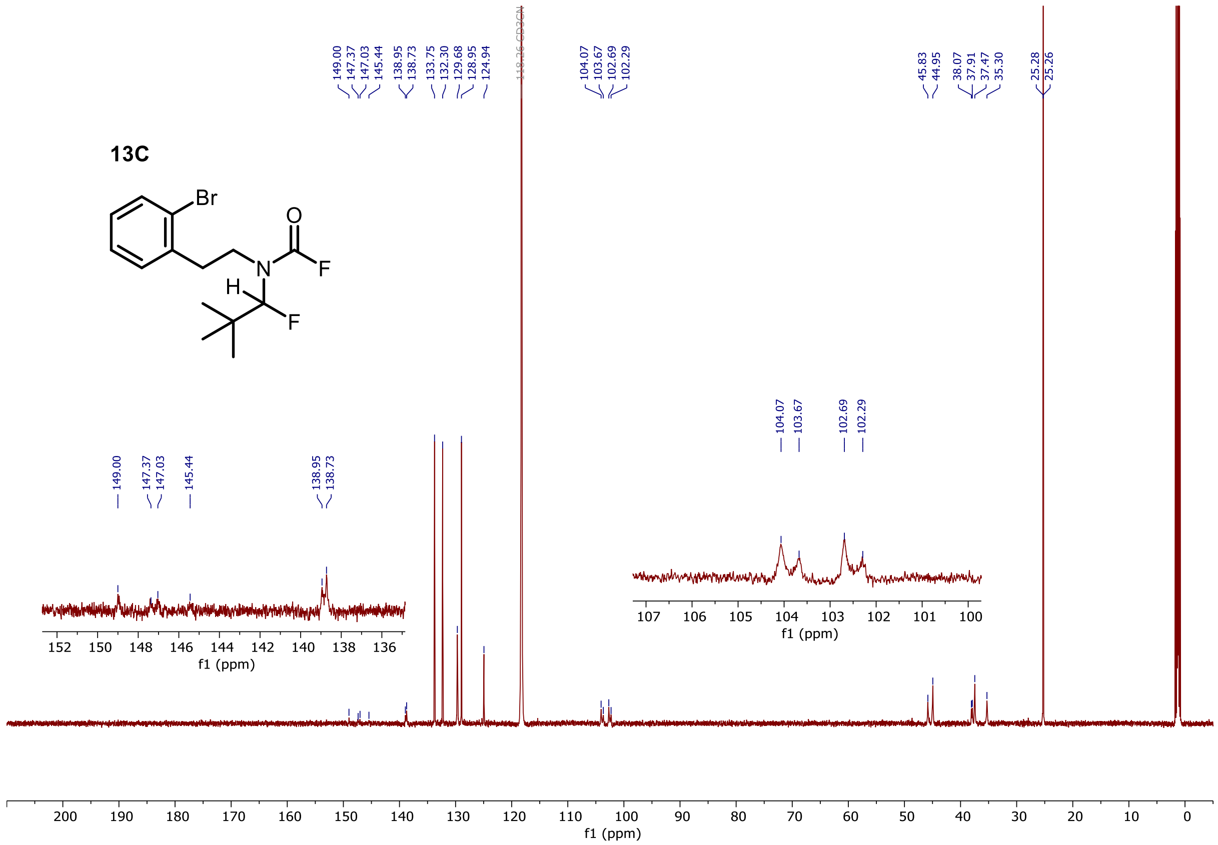

149.00
147.37
147.03
145.44
138.95
138.73
133.75
132.30
129.68
128.95
124.94
118.26 CD3CN
104.07
103.67
102.69
102.29
45.83
44.95
38.07
37.91
37.47
35.30
25.28
25.26
13C
Br
O
N
F
H
F
f1 (ppm)

10.2. *N*-CH_2F and *N*-CHRF derivatives

tert-Butyl 4-((fluoromethyl)(((4-oxo-2-phenyl-4H-chromen-7-yl)oxy)carbonyl)amino)piperidine-1-carboxylate (26)

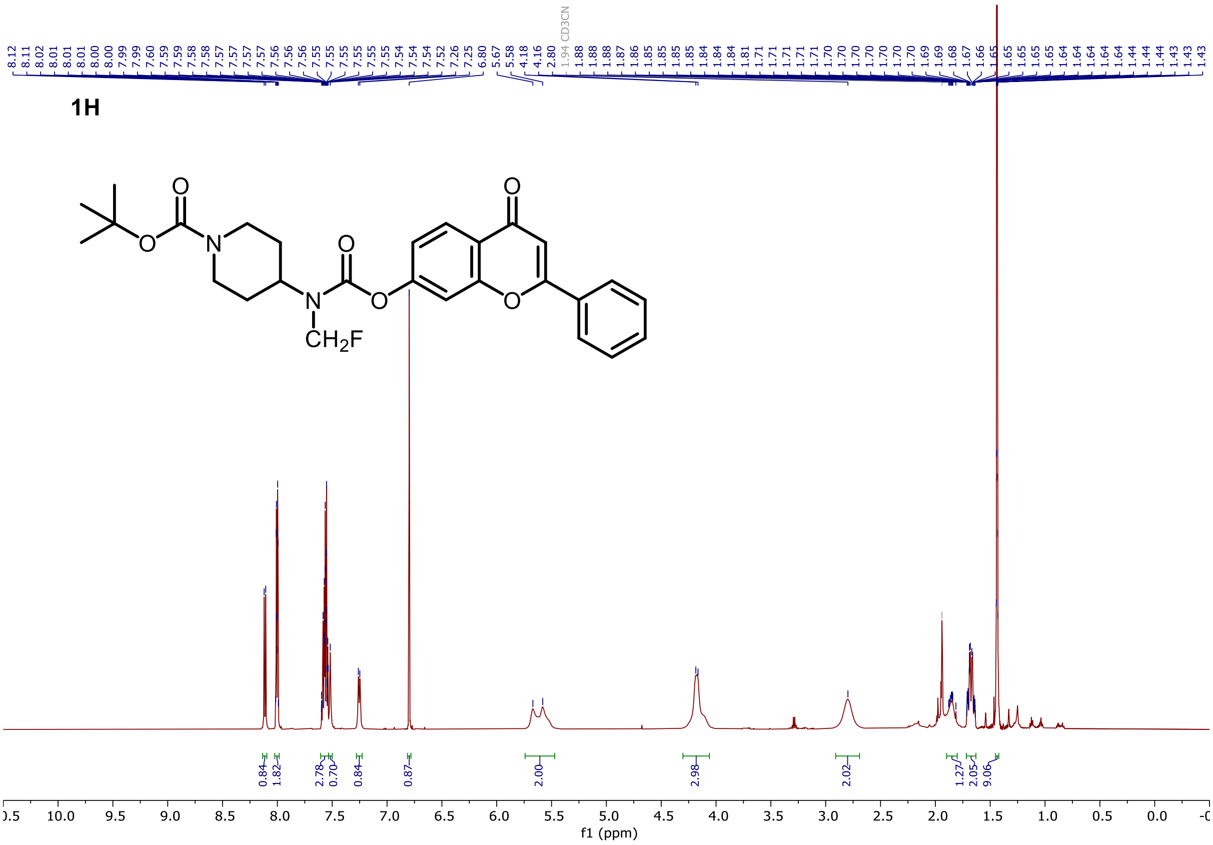

19F

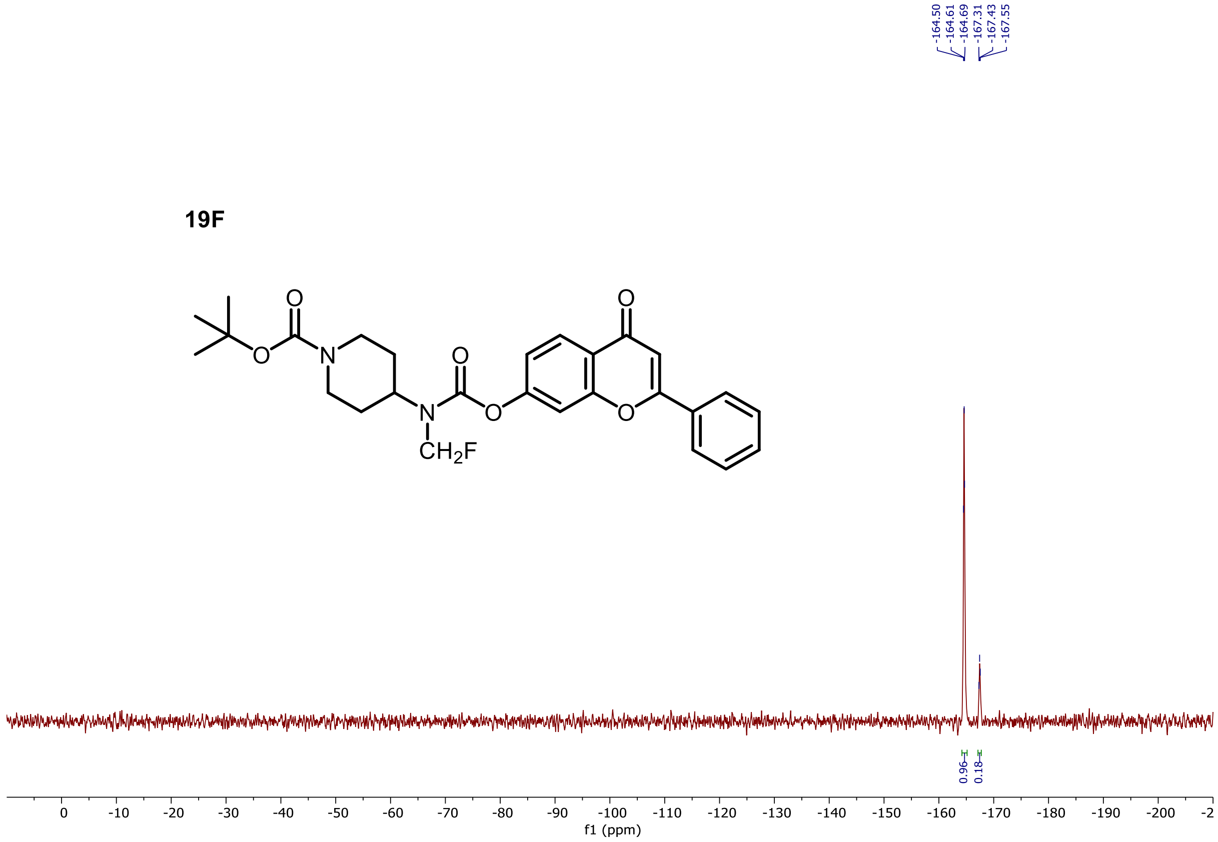
-164.50
-164.61
-164.69
-167.31
-167.43
-167.55
0.96
0.18
f1 (ppm)

13C

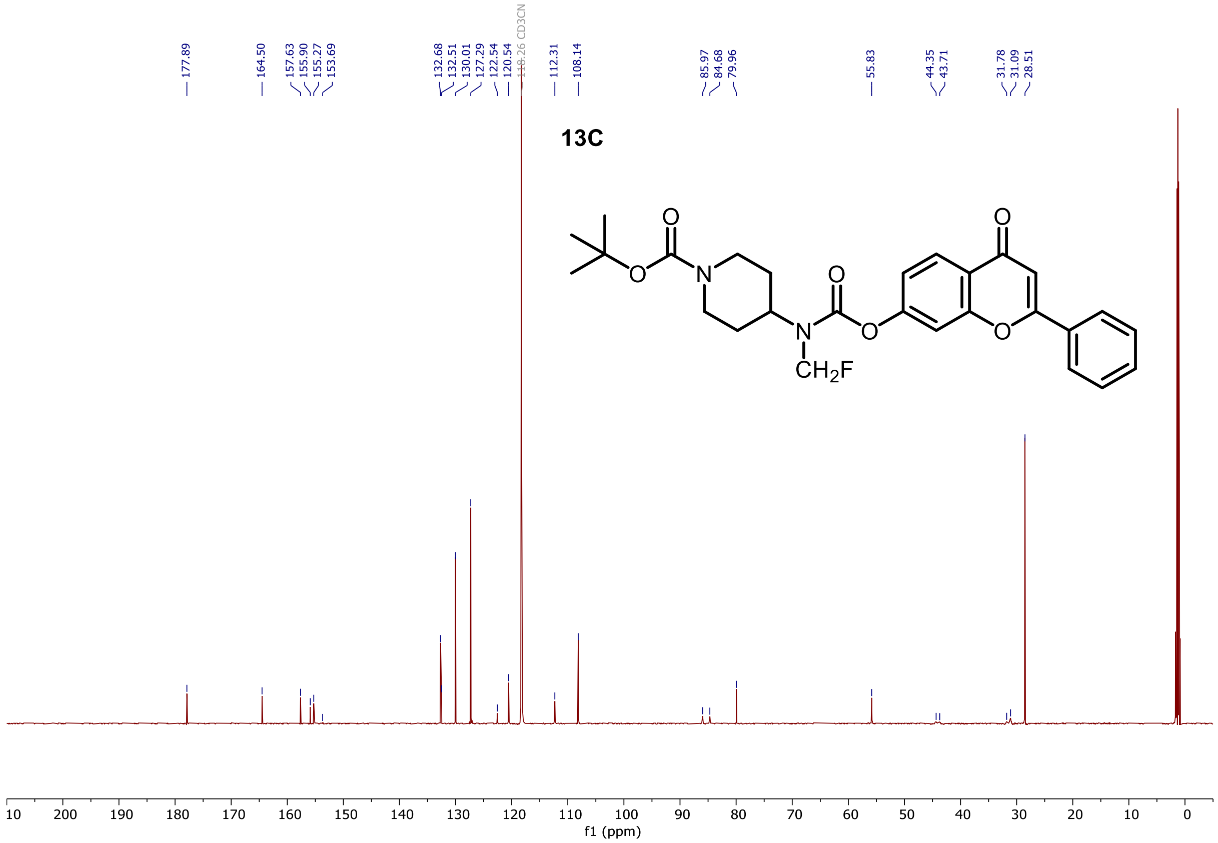
177.89
164.50
157.63
155.90
155.27
153.69
132.68
132.51
130.01
127.29
122.54
120.54
118.26 CD3CN
112.31
108.14
85.97
84.68
79.96
55.83
44.35
43.71
31.78
31.09
28.51
f1 (ppm)

Methyl (*S*)-3-(4-(([1,1'-biphenyl]-4-yl(fluoromethyl)carbamoyl)oxy)phenyl)-2-((*tert*-butoxy-carbonyl)amino)propanoate (27)

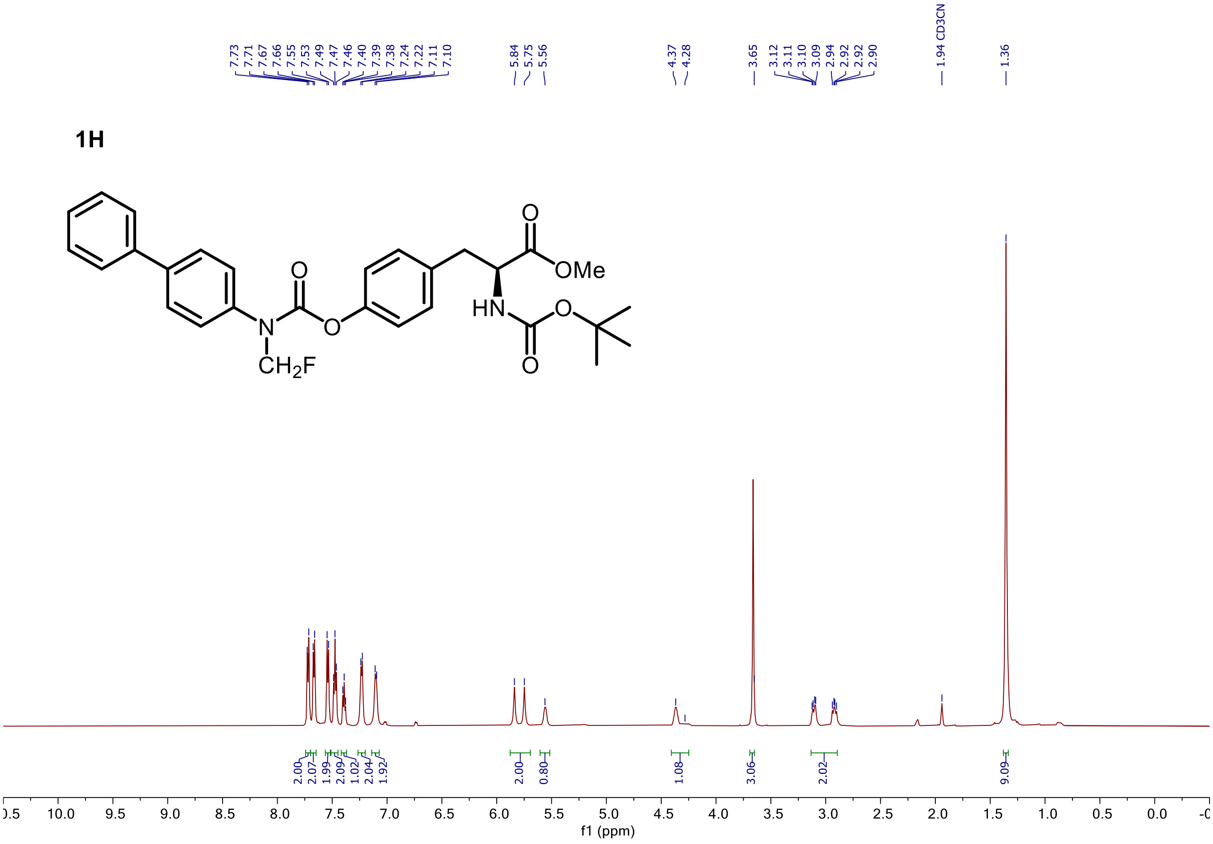

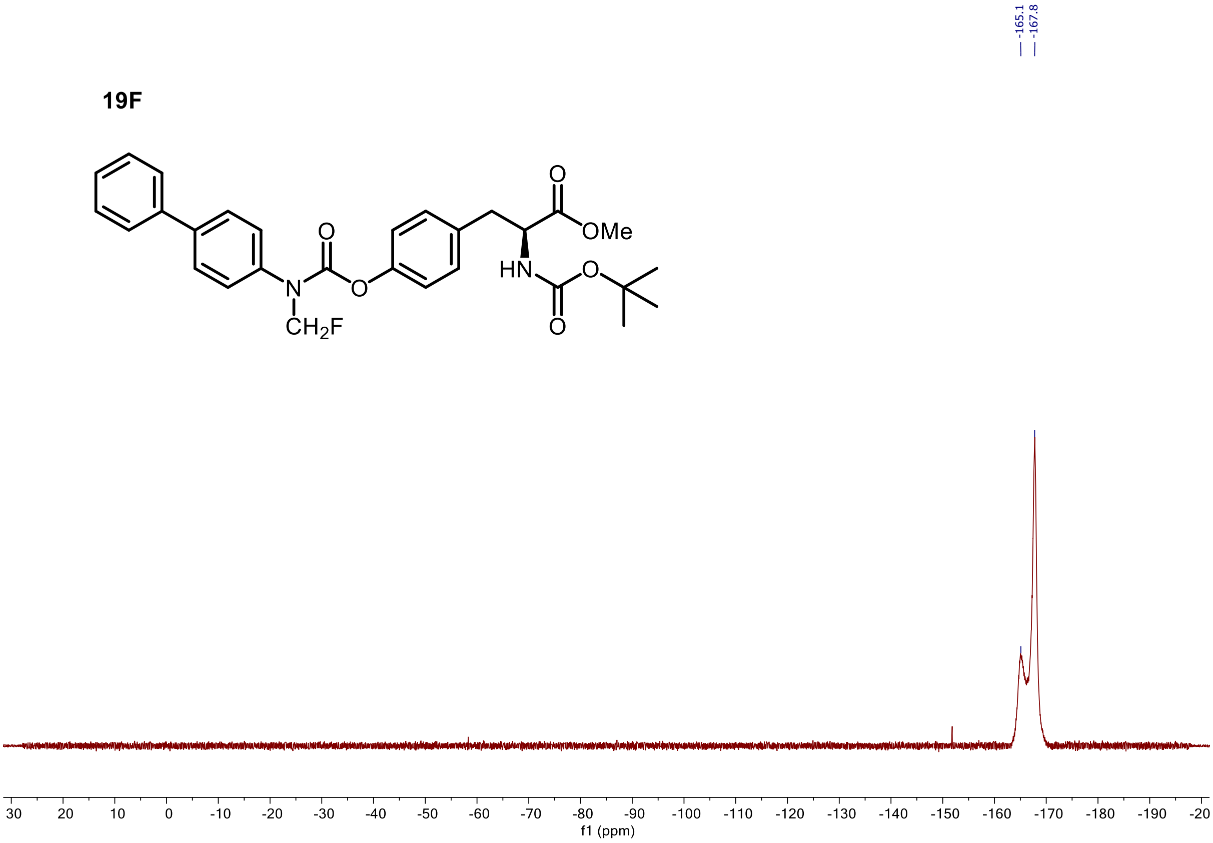

13C

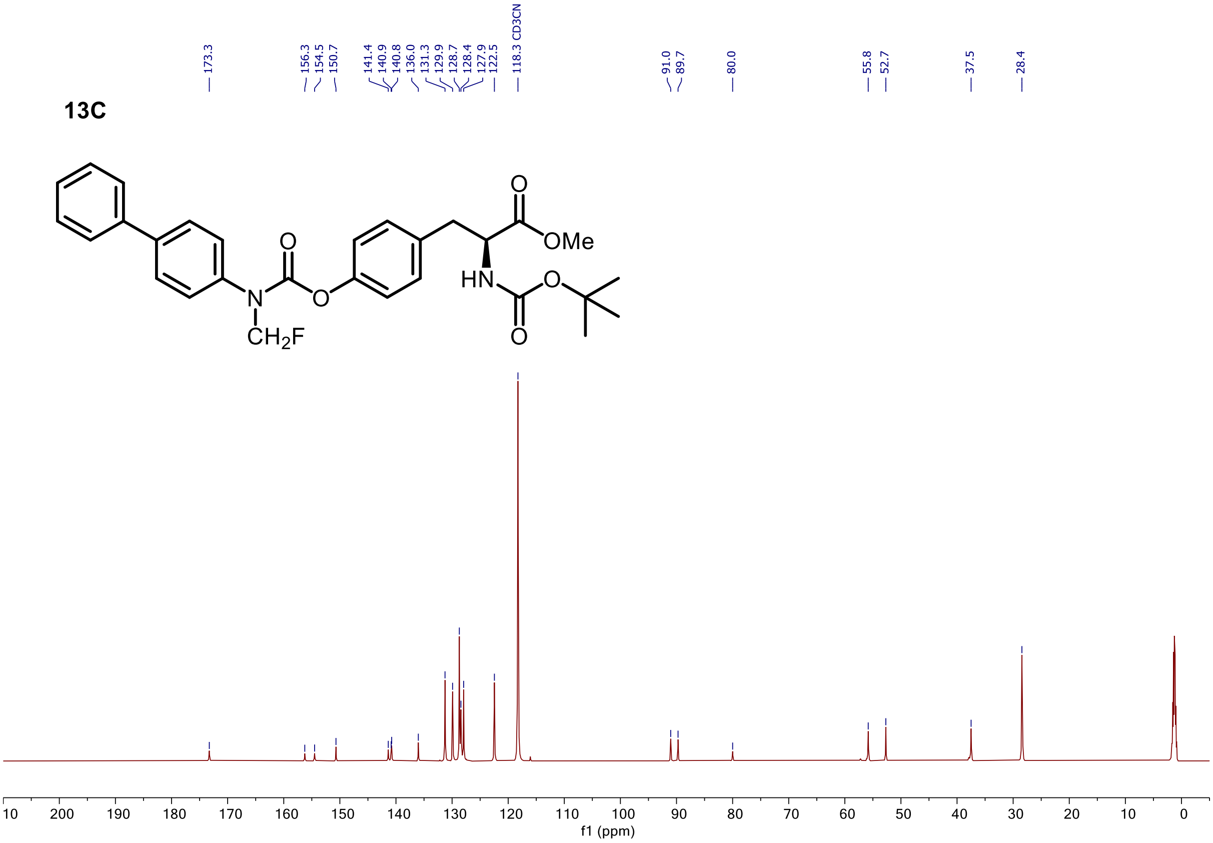

173.3
156.3
154.5
150.7
141.4
140.9
140.8
136.0
131.3
129.9
128.7
128.4
127.9
122.5
118.3 CD3CN
91.0
89.7
80.0
55.8
52.7
37.5
28.4
OMe
HN
N
CH2F
f1 (ppm)

((3a*R*,5*R*,5a*S*,8a*S*,8b*R*)-2,2,7,7-Tetramethyltetrahydro-5*H*-bis([1,3]dioxolo)[4,5-*b*:4',5'-*d*]pyran-5-yl)methyl (fluoro(phenyl)methyl)(phenyl)carbamate (28)

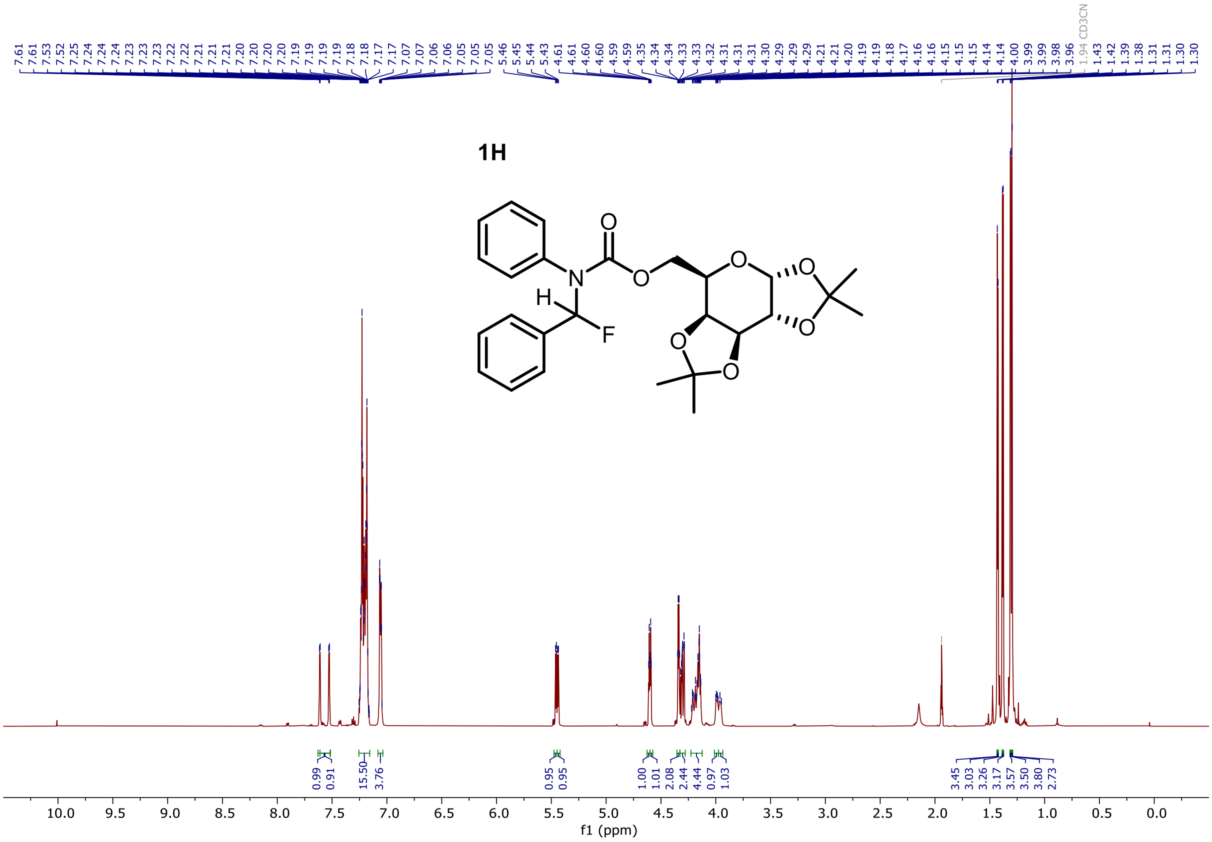

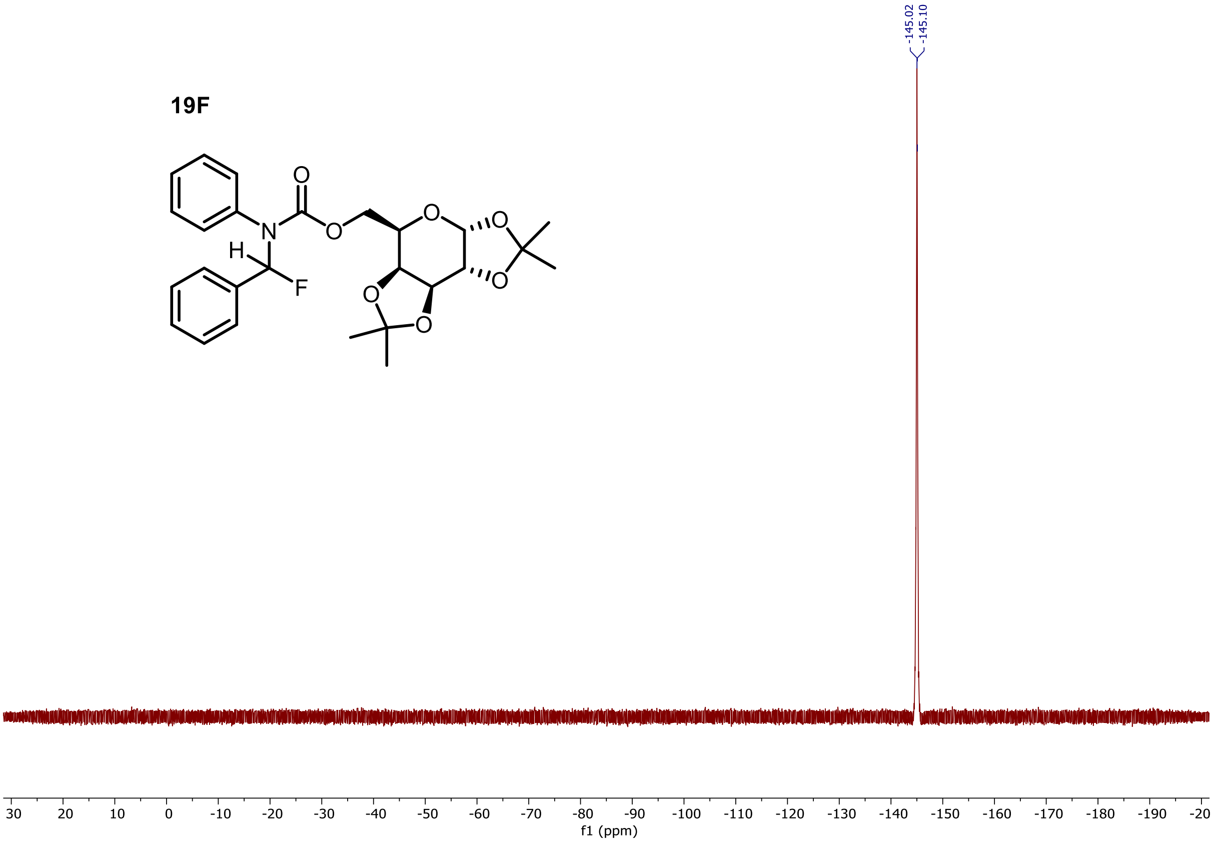

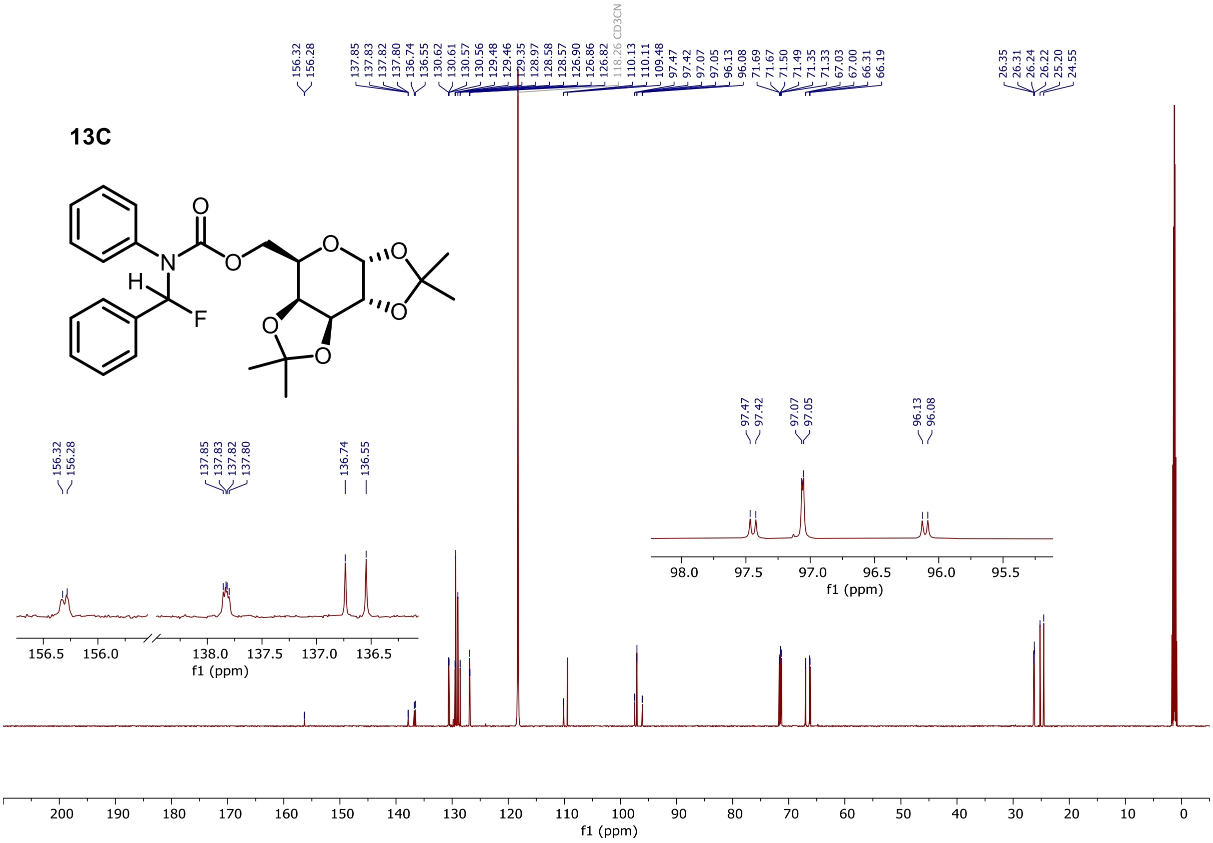

S-Ethyl (fluoromethyl)(3,4,5-trimethoxyphenyl)carbamothioate (29)

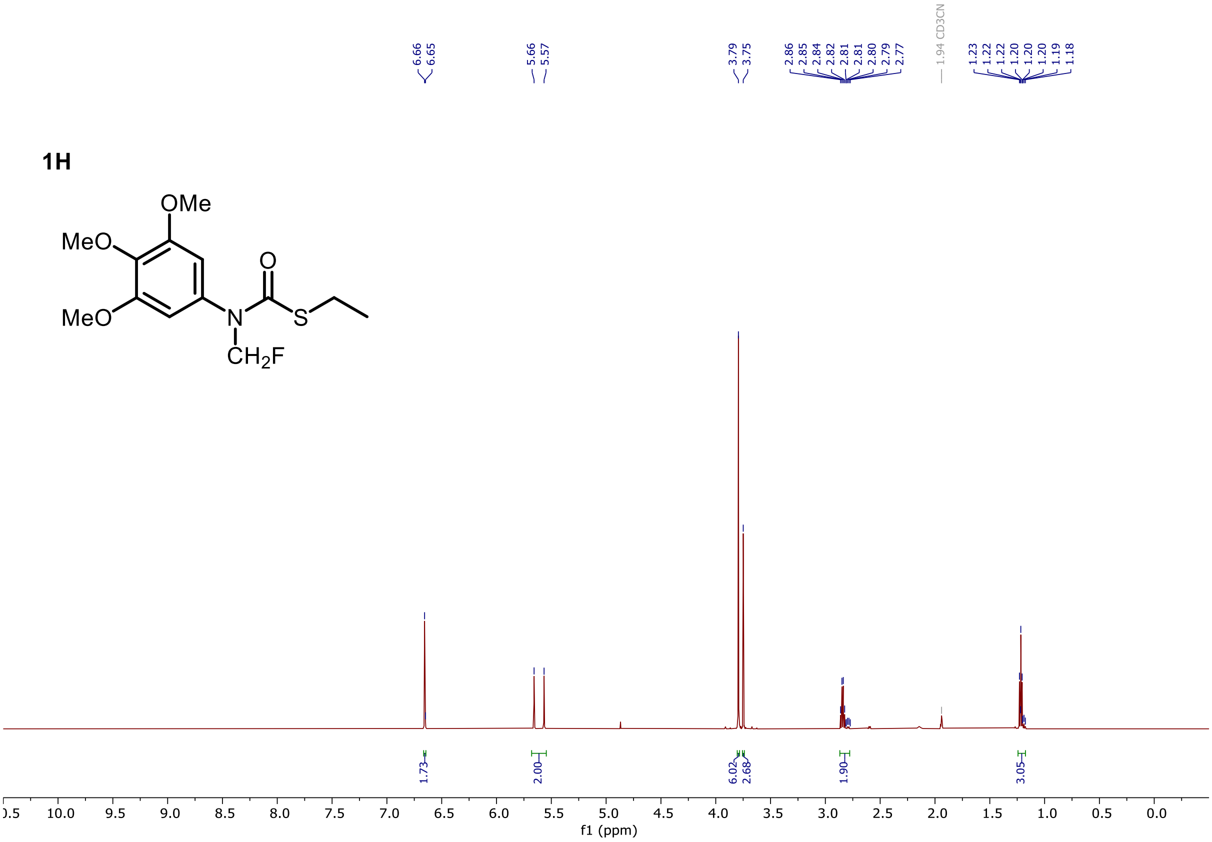

19F

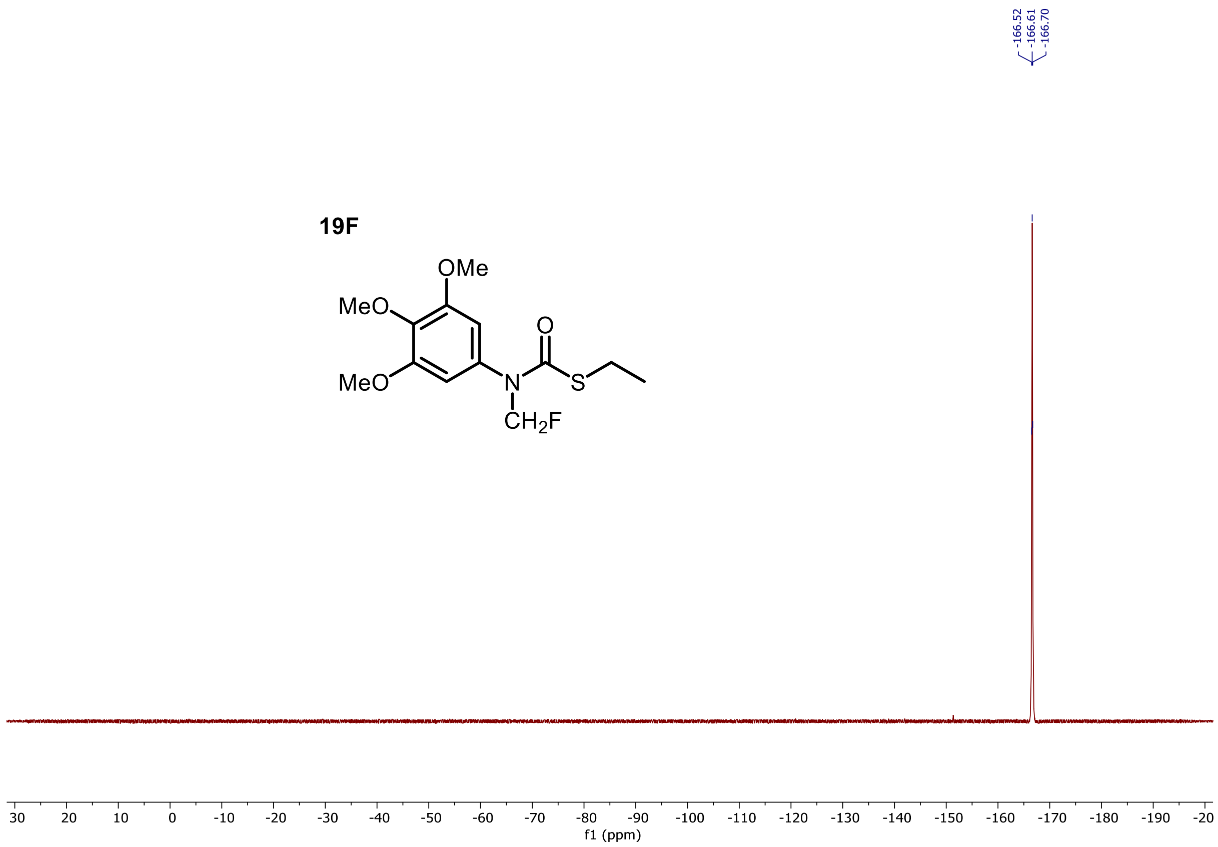

-166.52
-166.61
-166.70
OMe
MeO
MeO
O
N
S
CH2F
30 20 10 0 -10 -20 -30 -40 -50 -60 -70 -80 -90 -100 -110 -120 -130 -140 -150 -160 -170 -180 -190 -20
f1 (ppm)

13C

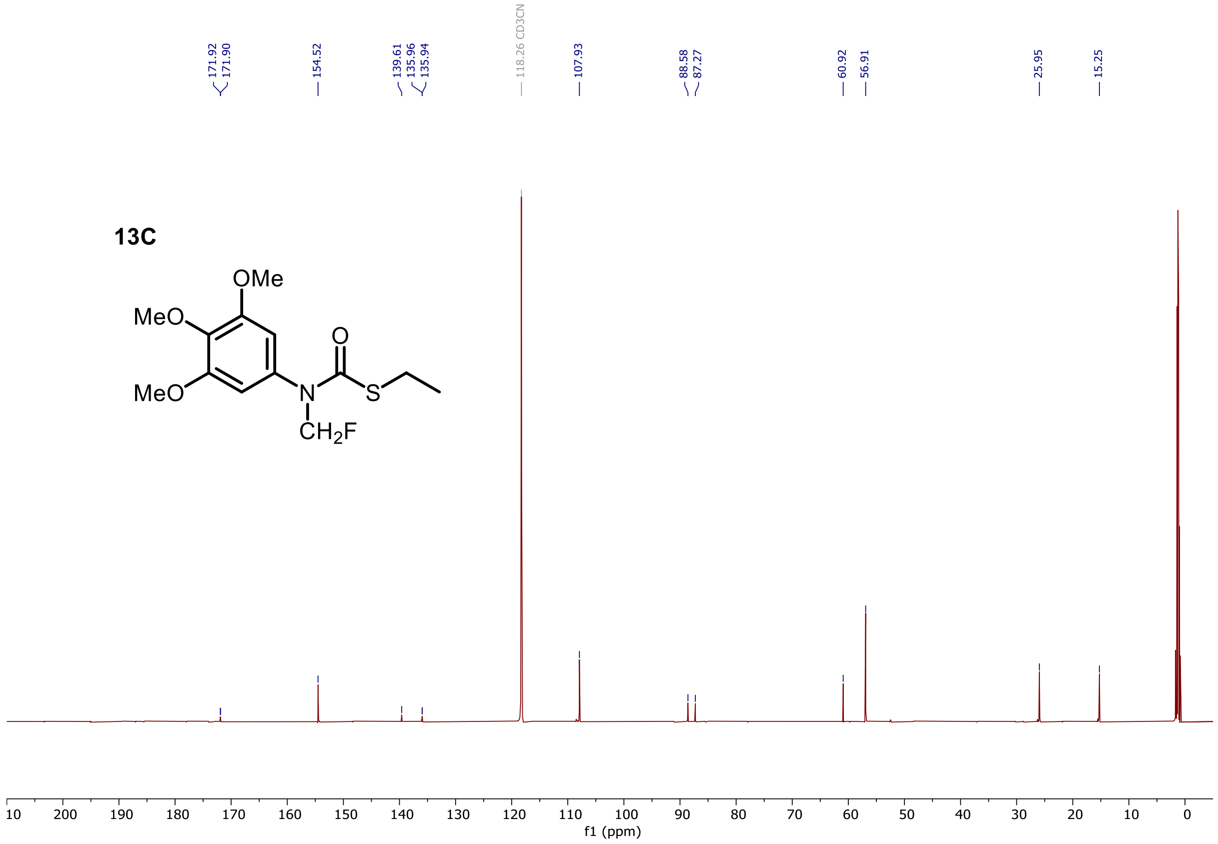

171.92
171.90
154.52
139.61
135.96
135.94
118.26 CD3CN
107.93
88.58
87.27
60.92
56.91
25.95
15.25
OMe
MeO
MeO
O
N
S
CH2F
10 200 190 180 170 160 150 140 130 120 110 100 90 80 70 60 50 40 30 20 10 0
f1 (ppm)

***S*-(Pyridin-2-yl) (fluoromethyl)(2-isopropylphenyl)carbamothioate (30)**

1H

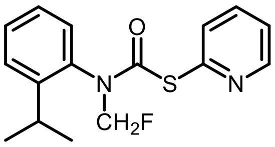

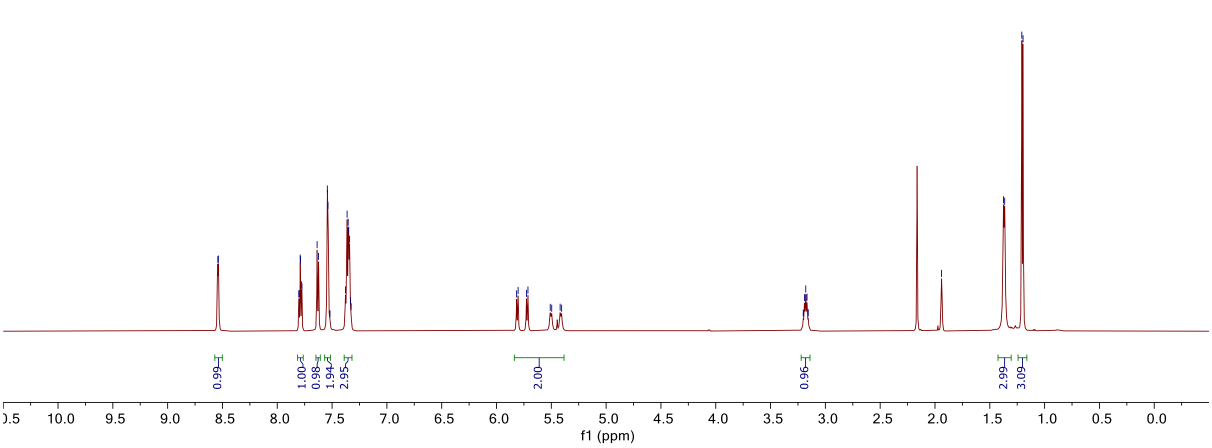

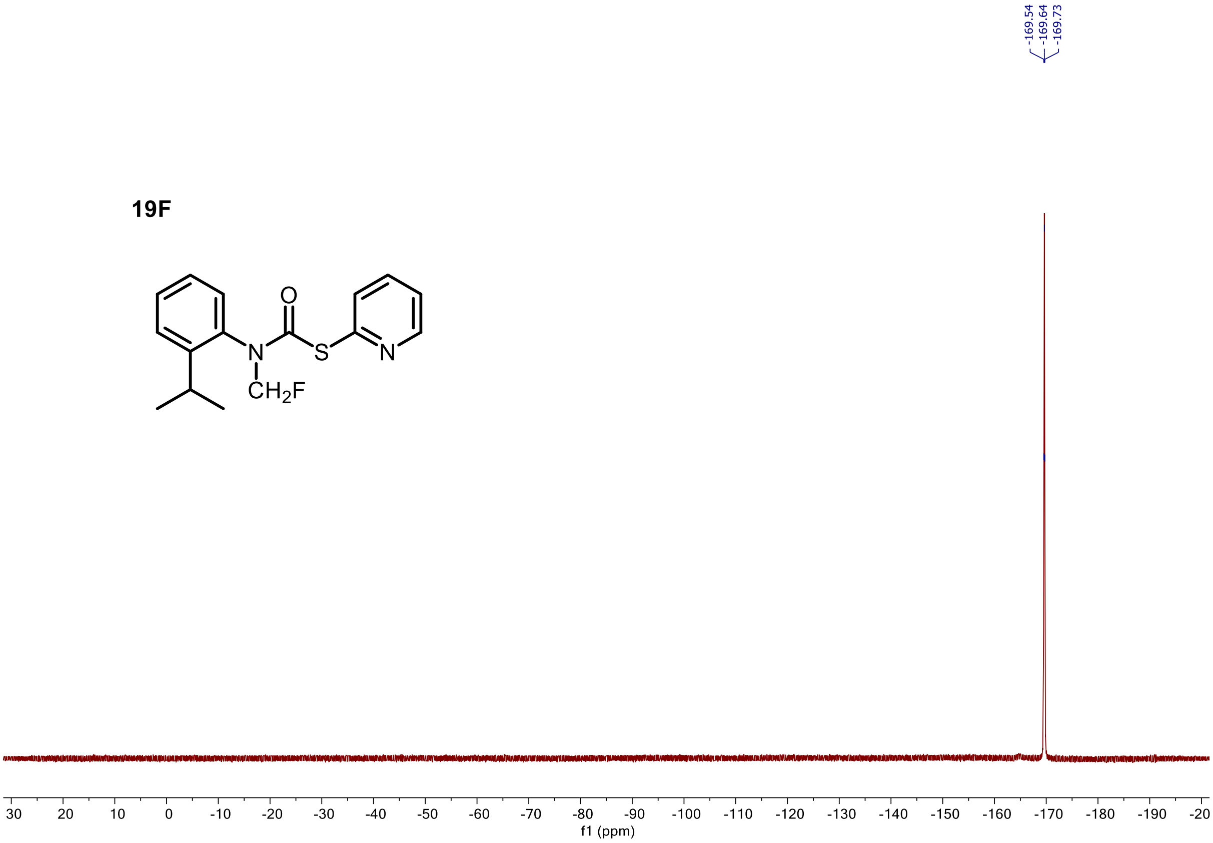
-169.54
-169.64
-169.73
19F
O
N
S
N
CH2F
30 20 10 0 -10 -20 -30 -40 -50 -60 -70 -80 -90 -100 -110 -120 -130 -140 -150 -160 -170 -180 -190 -20
f1 (ppm)

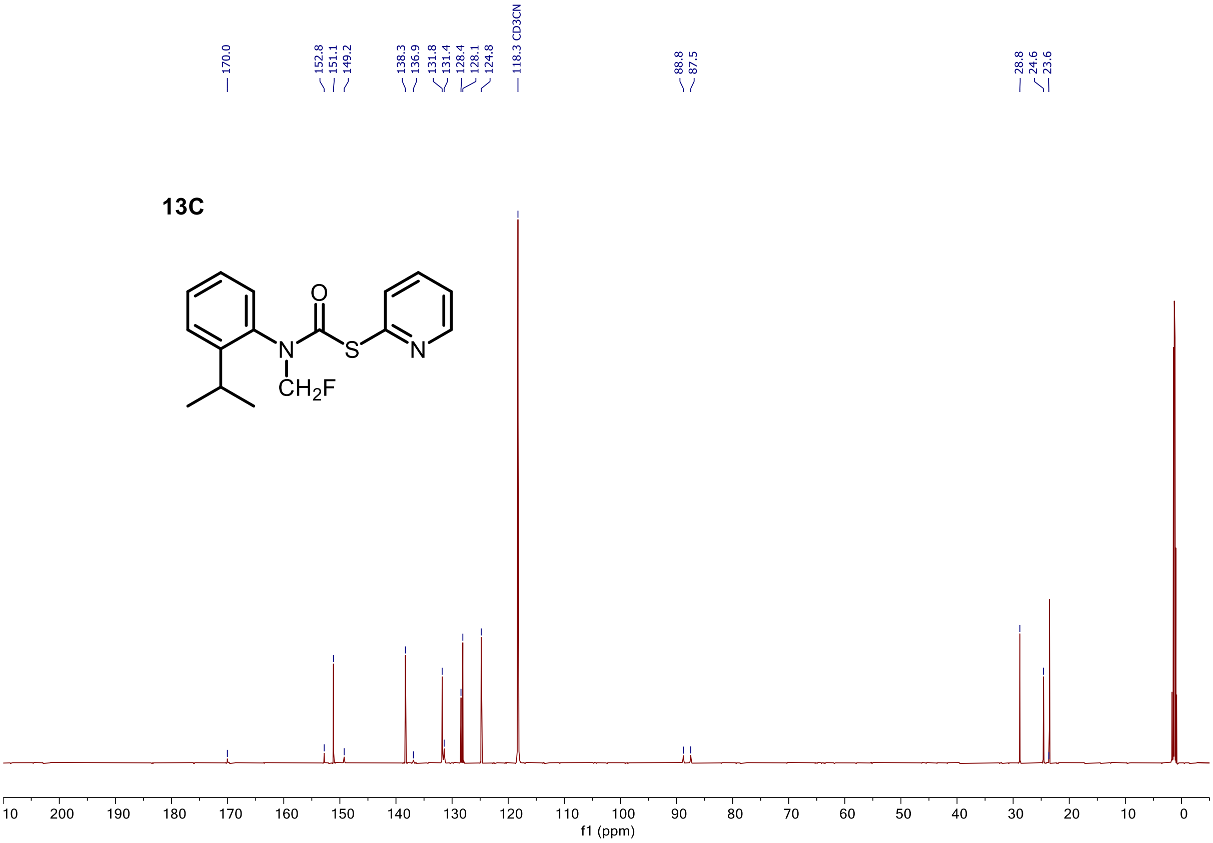
170.0
152.8
151.1
149.2
138.3
136.9
131.8
131.4
128.4
128.1
124.8
118.3 CD3CN
88.8
87.5
28.8
24.6
23.6
13C
O
N
S
N
CH2F
10 200 190 180 170 160 150 140 130 120 110 100 90 80 70 60 50 40 30 20 10 0
f1 (ppm)

***S*-(2-Chlorophenyl) (fluoro(phenyl)methyl)(phenyl)carbamothioate (31)**

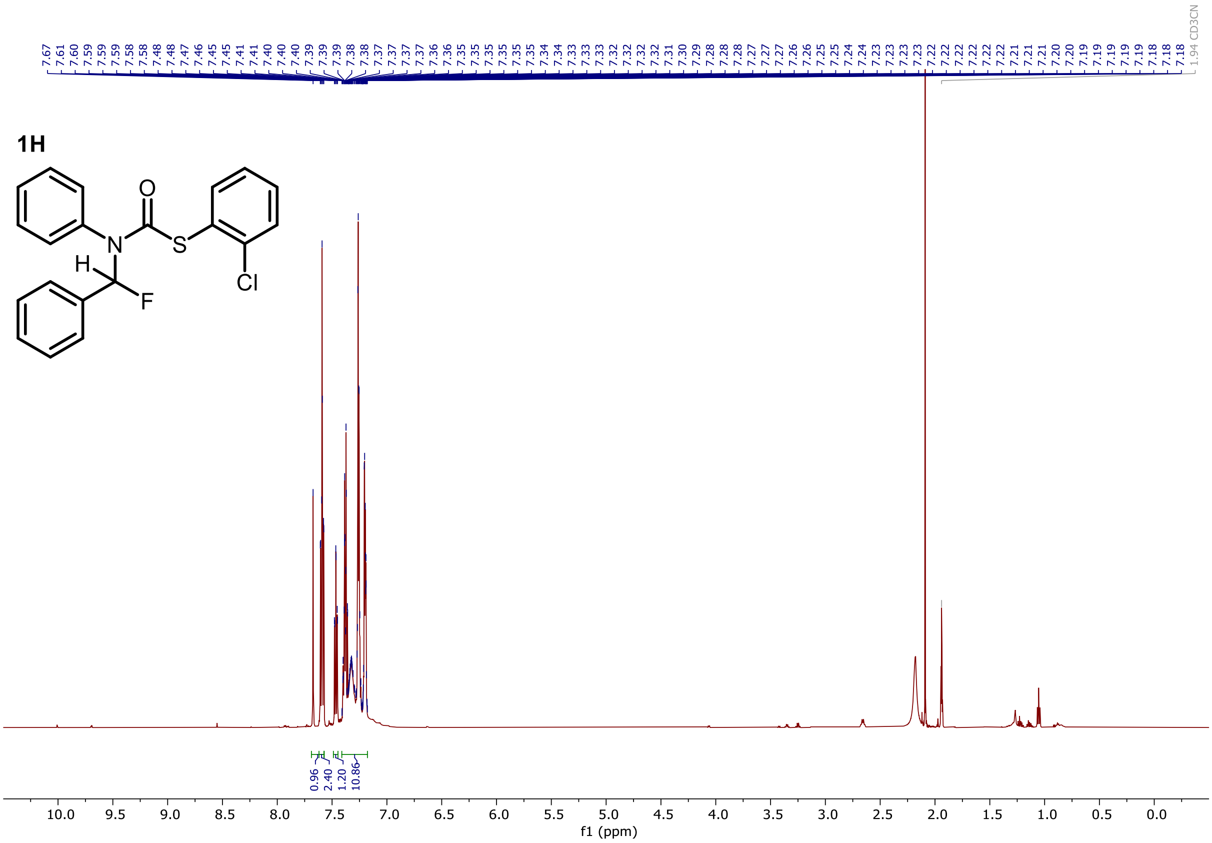

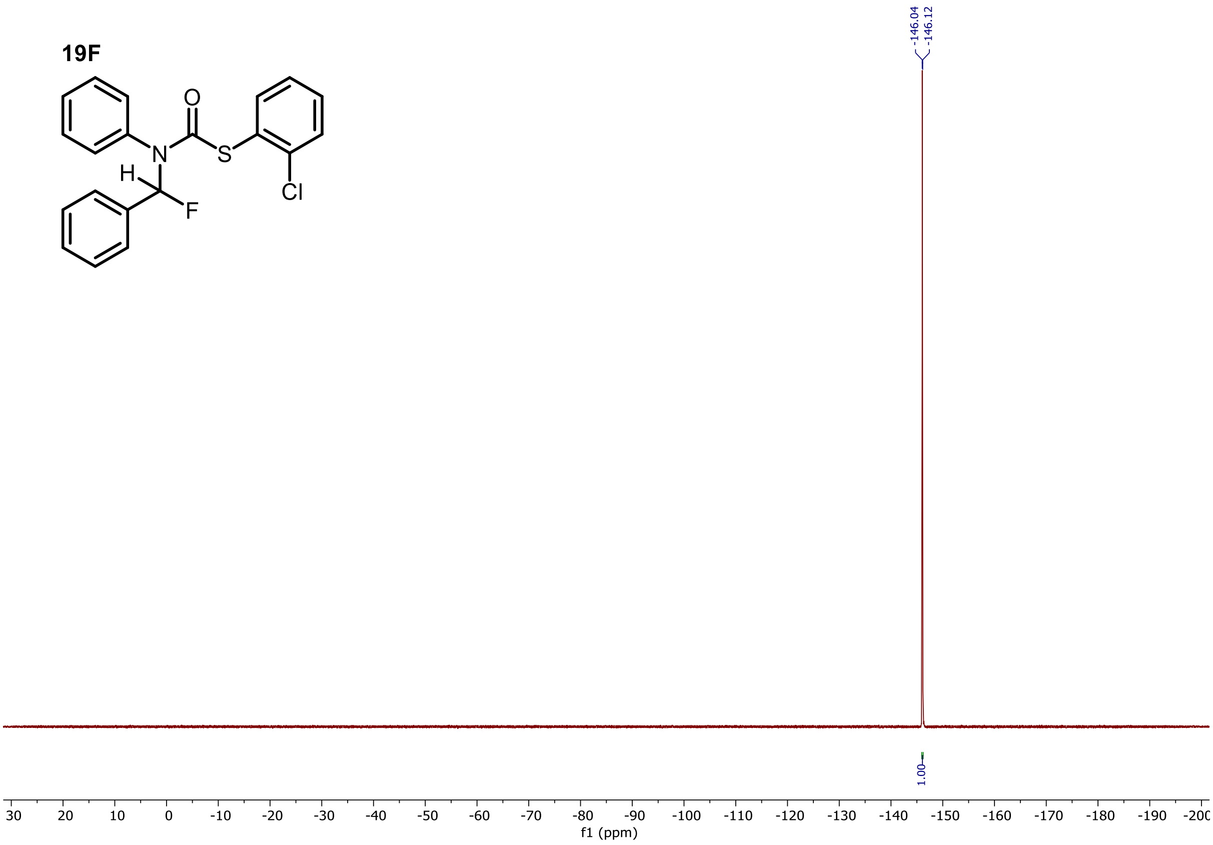

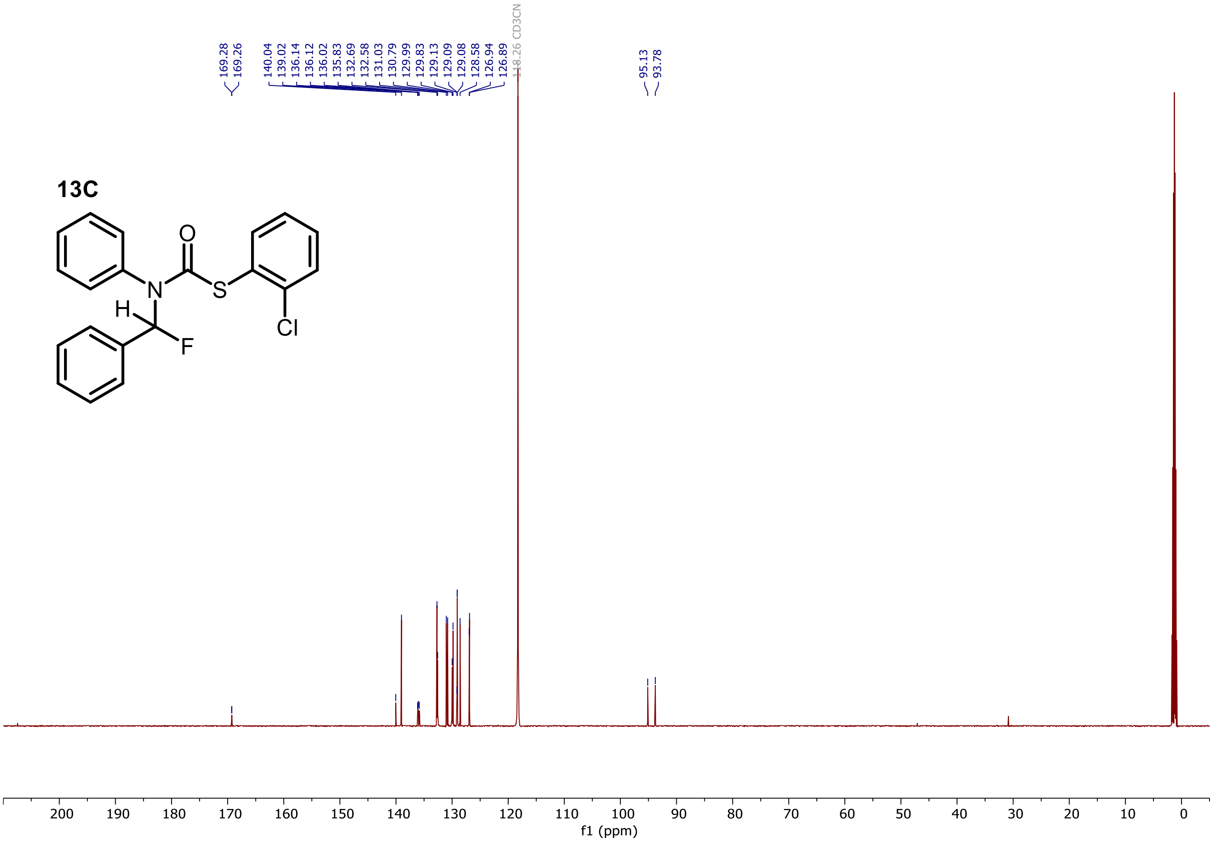

N-(1-(2,6-Dimethylphenoxy)propan-2-yl)-*N*-(fluoromethyl)formamide (32)

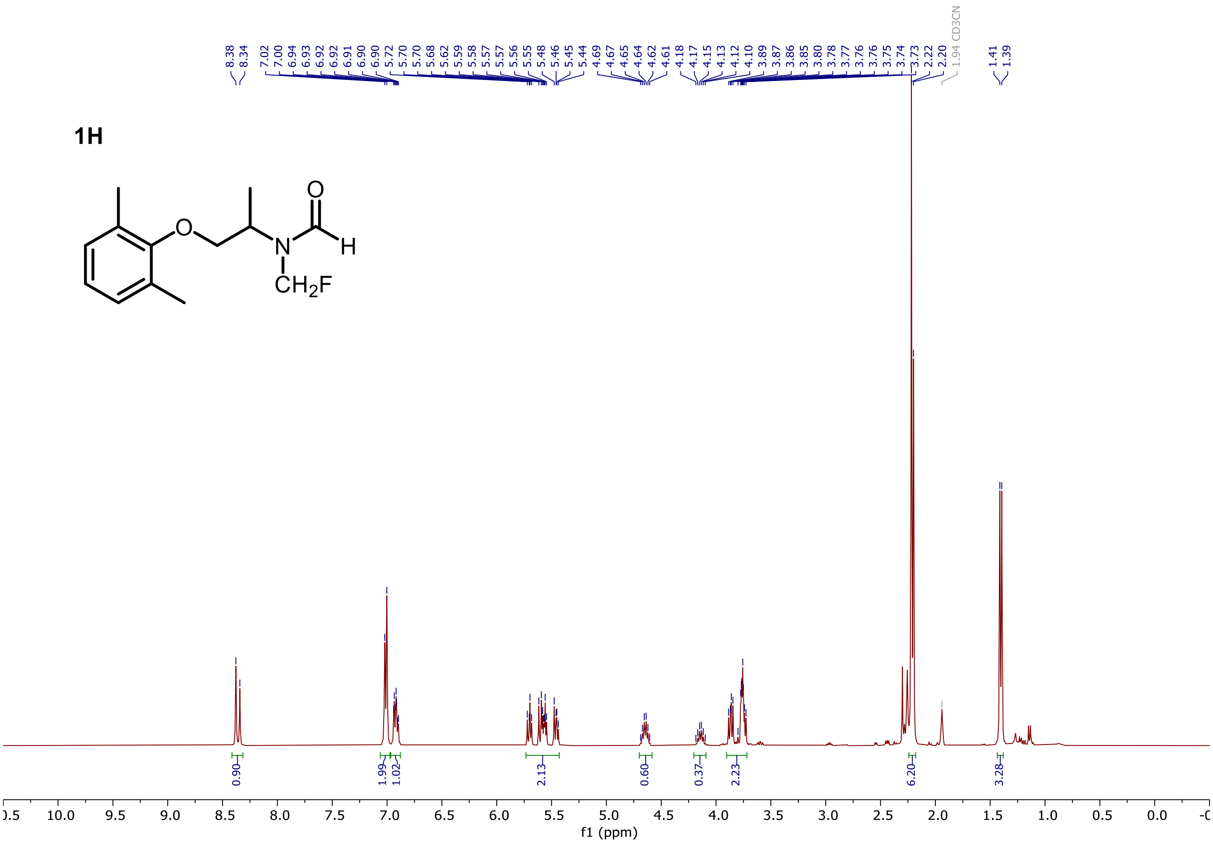

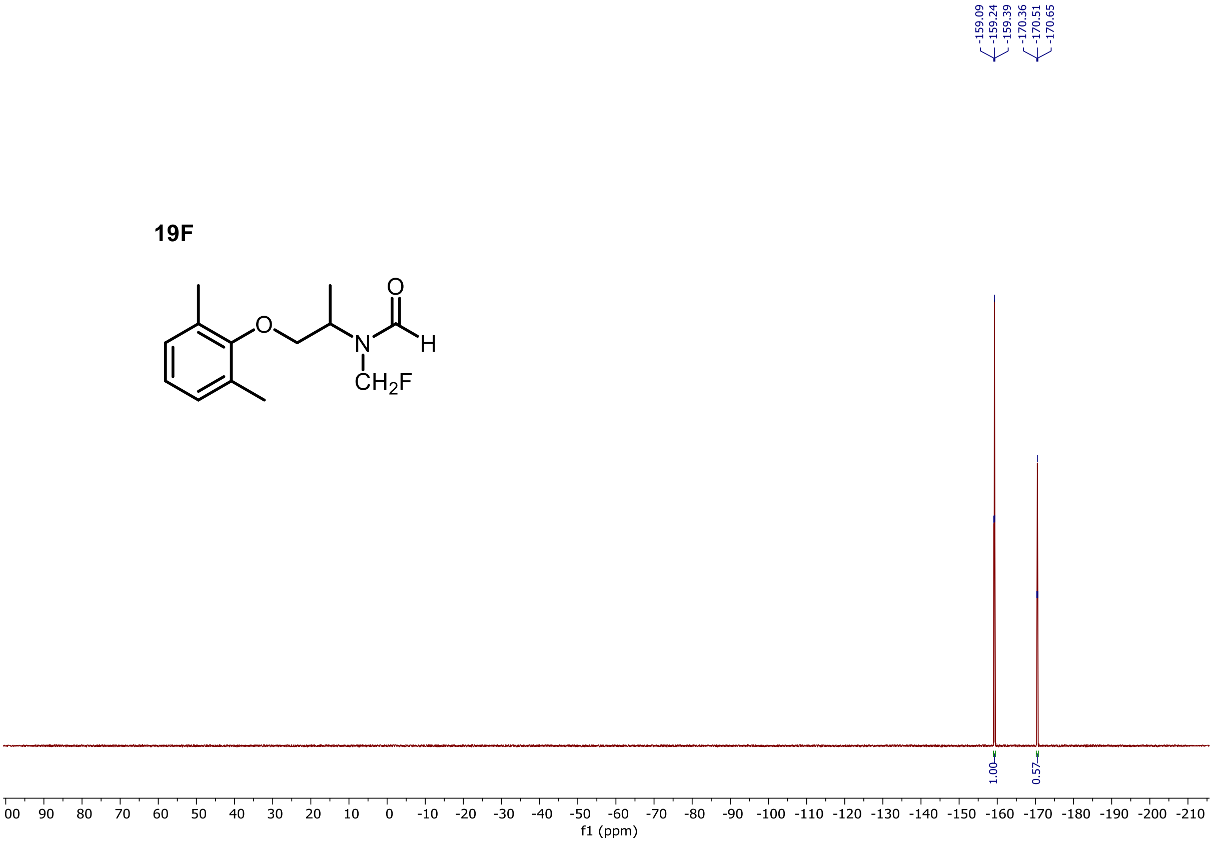
19F
-159.09
-159.24
-159.39
-170.36
-170.51
-170.65
1.00
0.57
f1 (ppm)

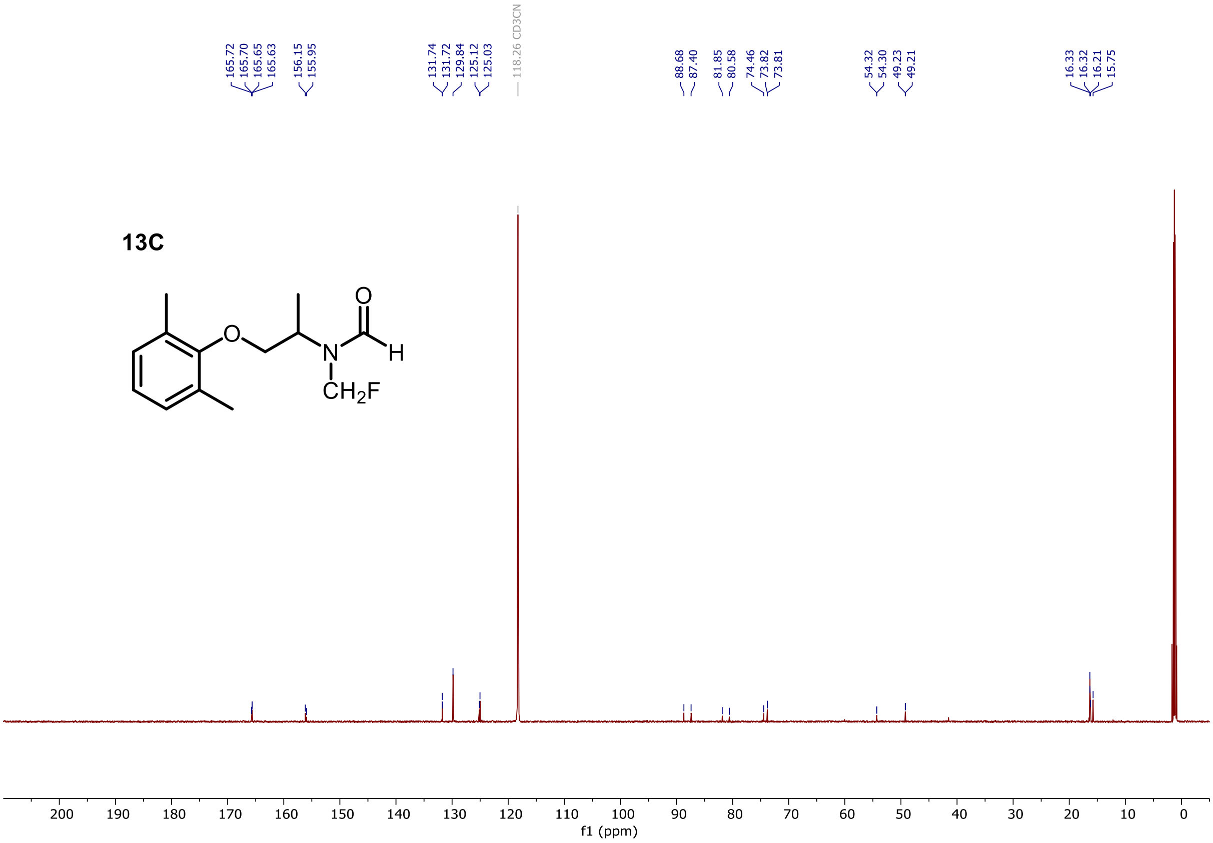
13C
165.72
165.70
165.65
165.63
156.15
155.95
131.74
131.72
129.84
125.12
125.03
118.26 CD3CN
88.68
87.40
81.85
80.58
74.46
73.82
73.81
54.32
54.30
49.23
49.21
16.33
16.32
16.21
15.75
f1 (ppm)

N-([1,1'-Biphenyl]-4-yl)-N-(fluoromethyl-d2)formamide-d (33)

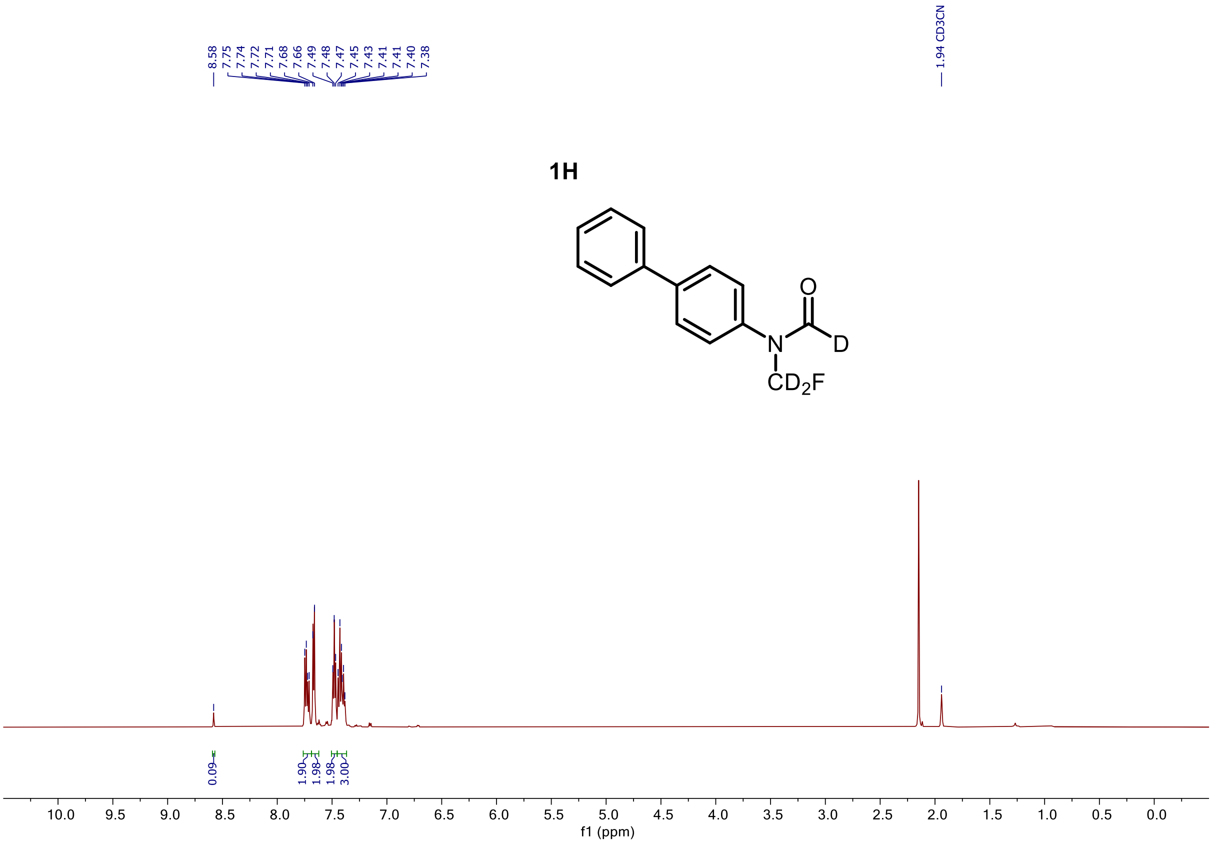

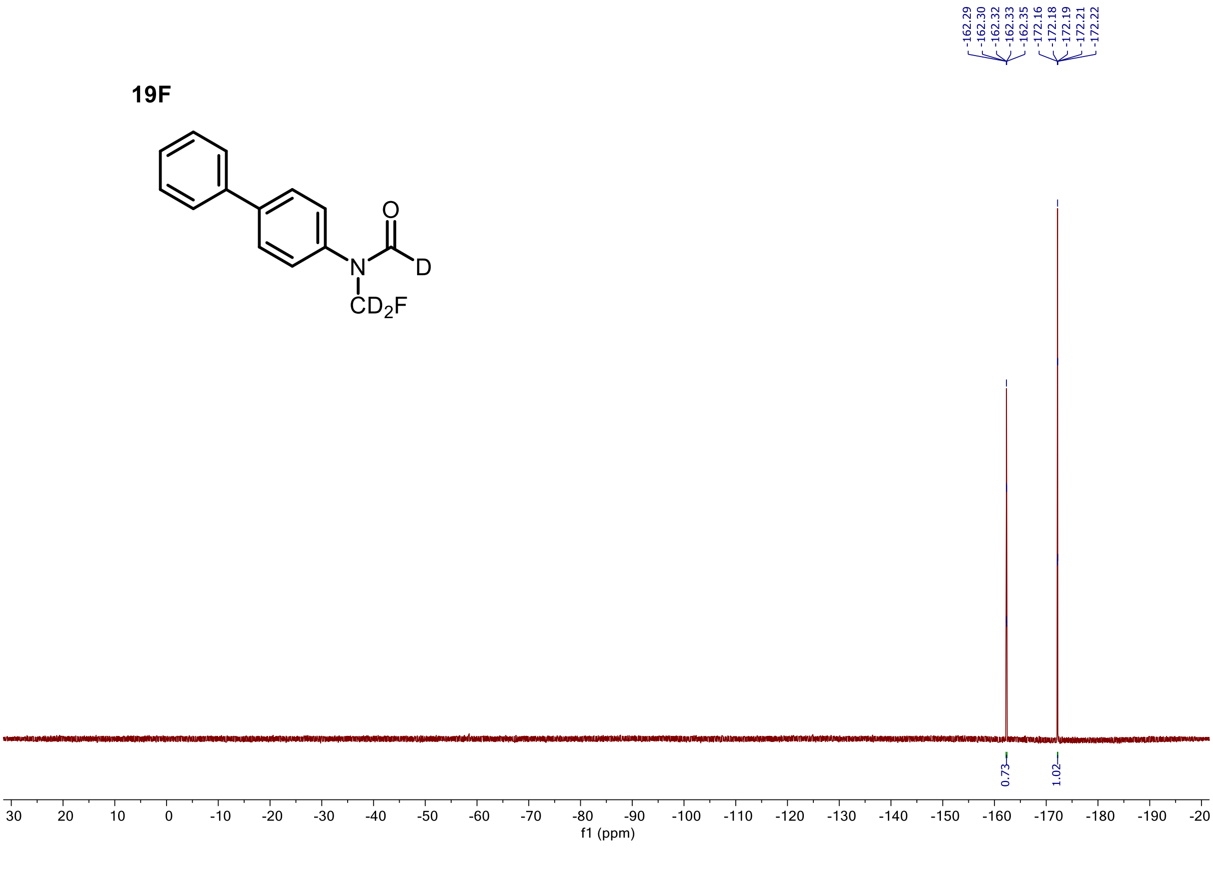

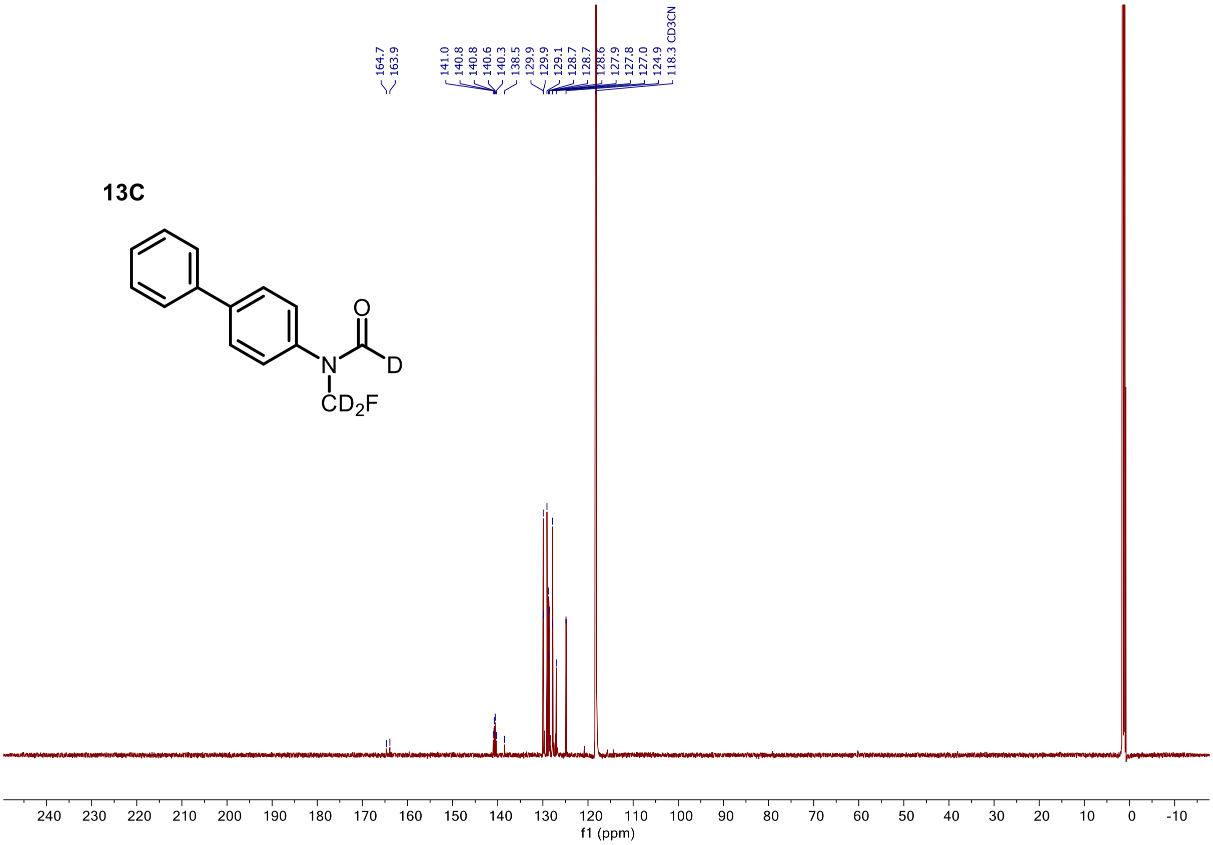

N-([1,1'-Biphenyl]-4-yl)-*N*-(fluoromethyl)formamide (34)

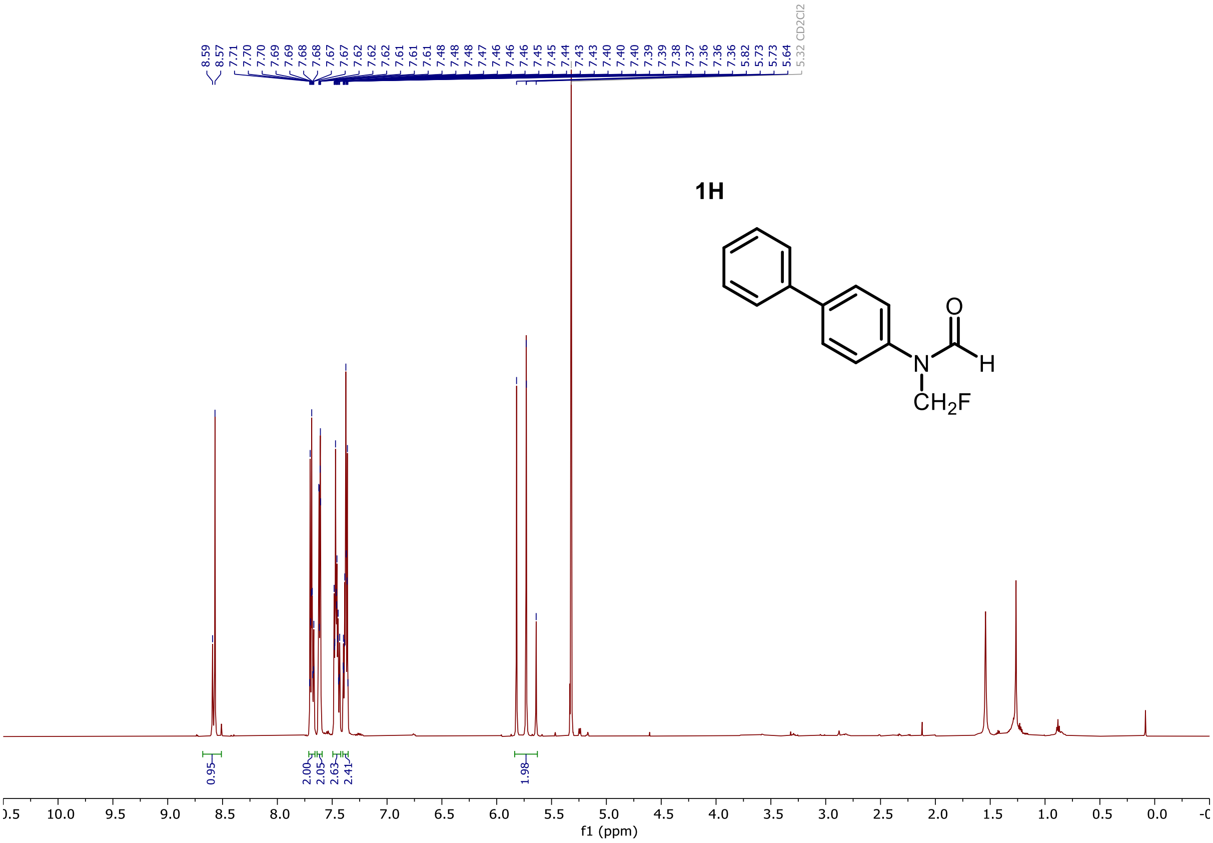

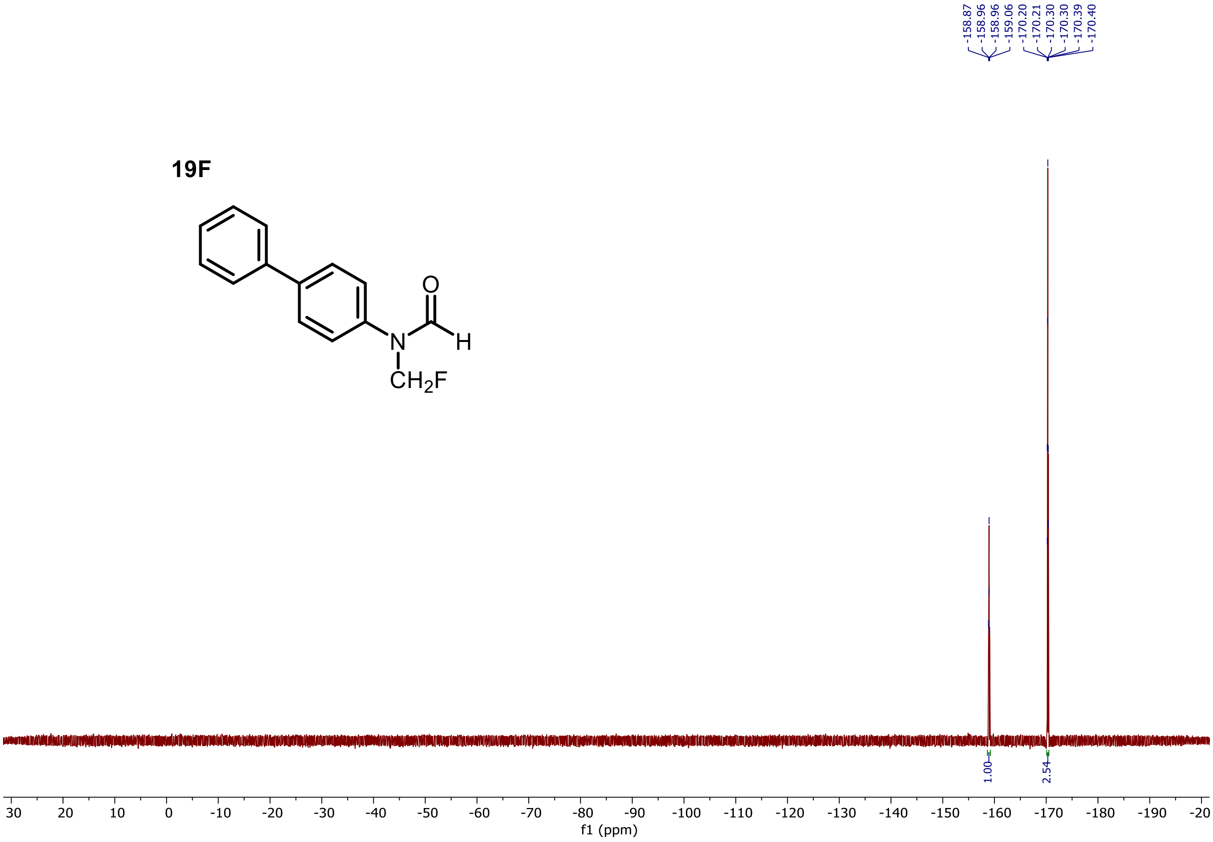
-158.87
-158.96
-158.96
-159.06
-170.20
-170.21
-170.30
-170.30
-170.39
-170.40
19F
O
N
H
CH2F
1.00
2.54
30 20 10 0 -10 -20 -30 -40 -50 -60 -70 -80 -90 -100 -110 -120 -130 -140 -150 -160 -170 -180 -190 -20
f1 (ppm)

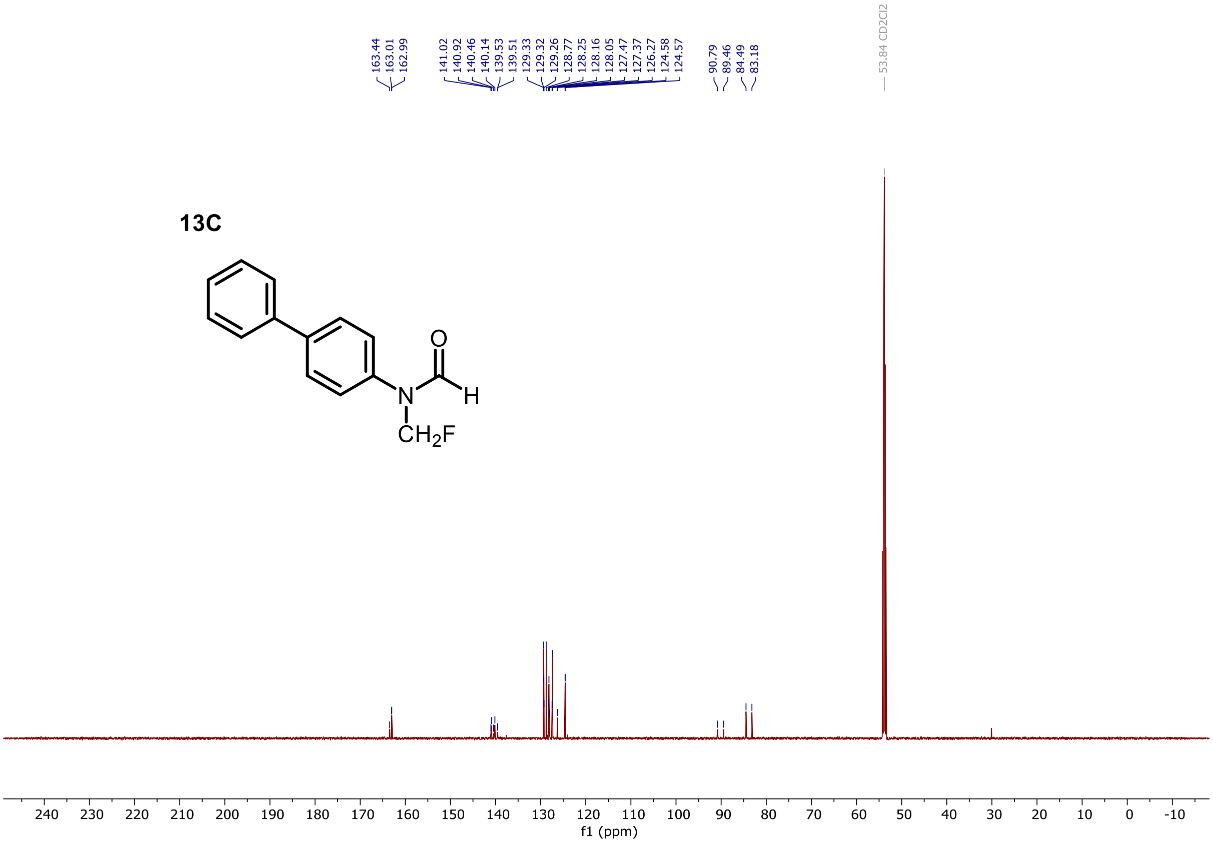
163.44
163.01
162.99
141.02
140.92
140.46
140.14
139.53
139.51
129.33
129.32
129.26
128.77
128.25
128.16
128.05
127.47
127.37
126.27
124.58
124.57
90.79
89.46
84.49
83.18
53.84 CD2Cl2
13C
O
N
H
CH2F
240 230 220 210 200 190 180 170 160 150 140 130 120 110 100 90 80 70 60 50 40 30 20 10 0 -10
f1 (ppm)

_N_-(2-Bromophenethyl)-_N_-(1-fluoro-2,2-dimethylpropyl)formamide (35)

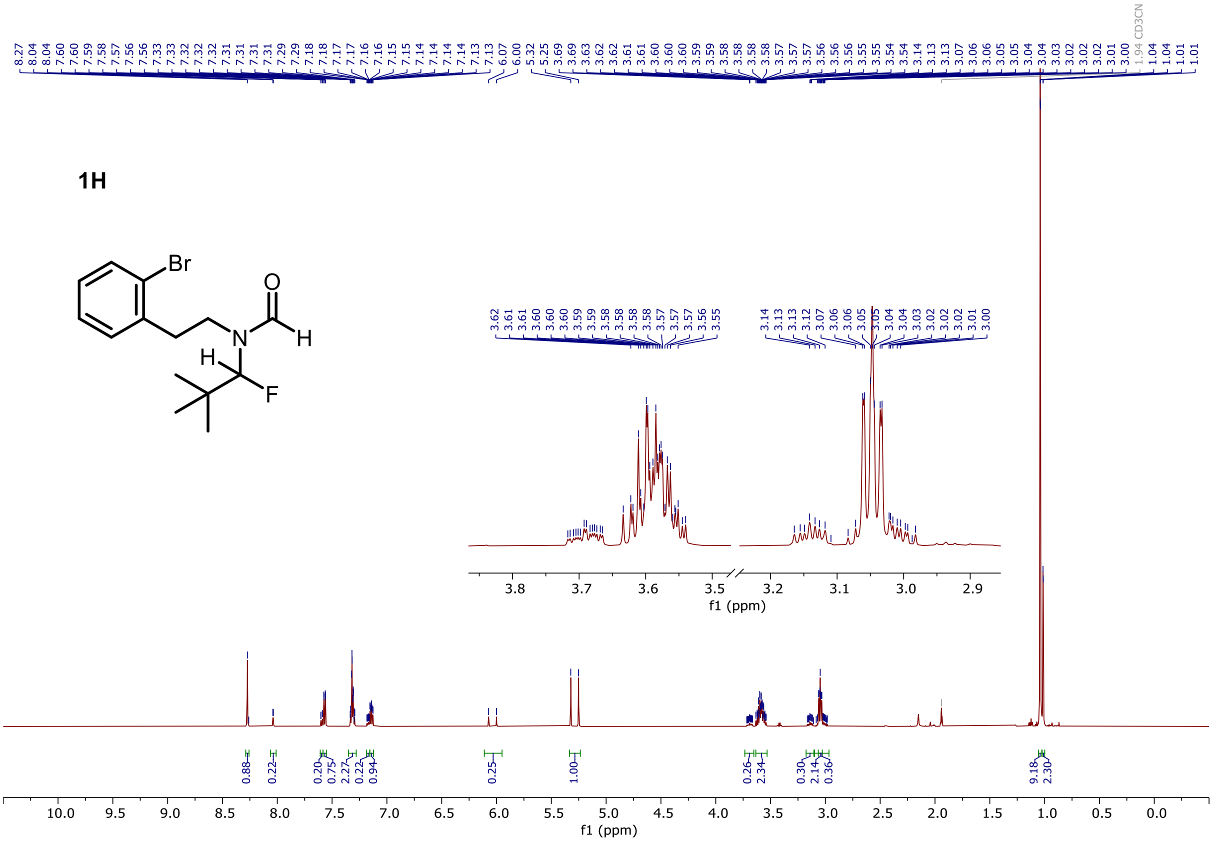

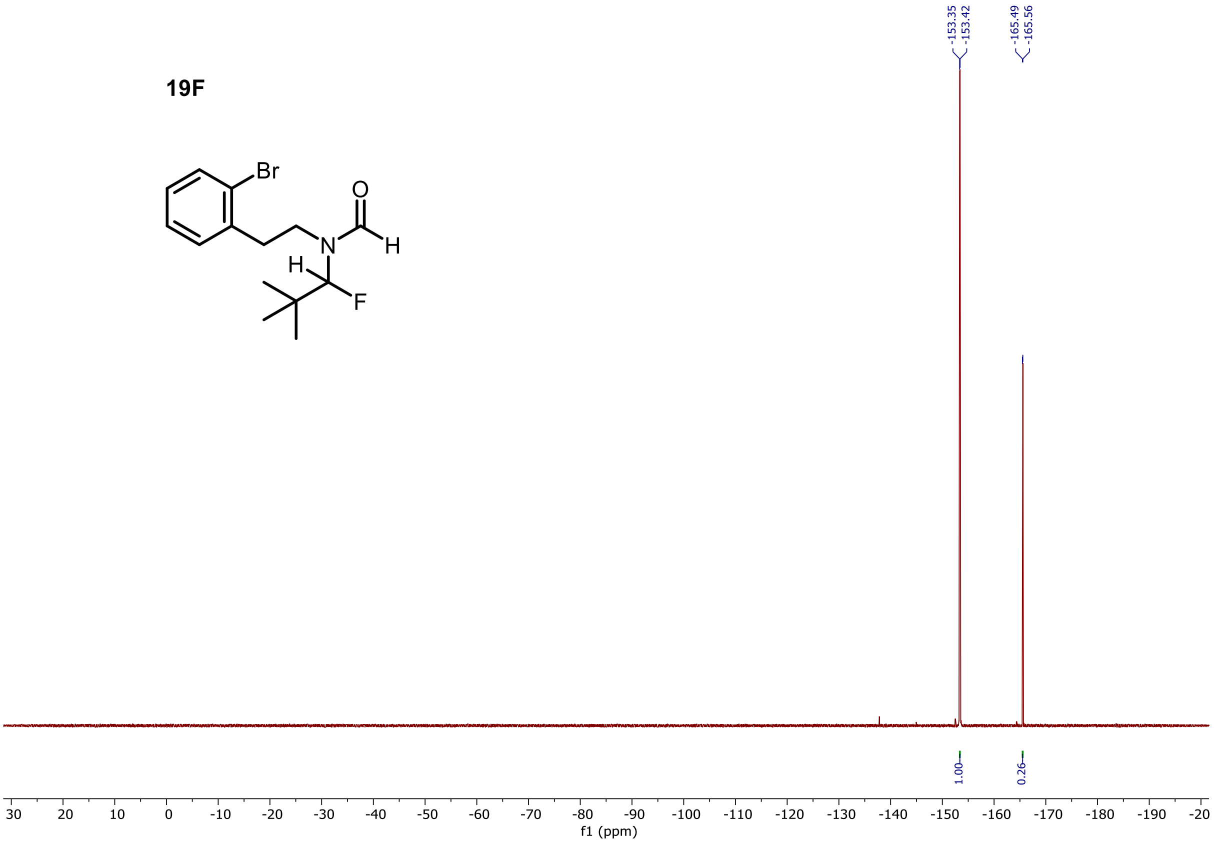

13C

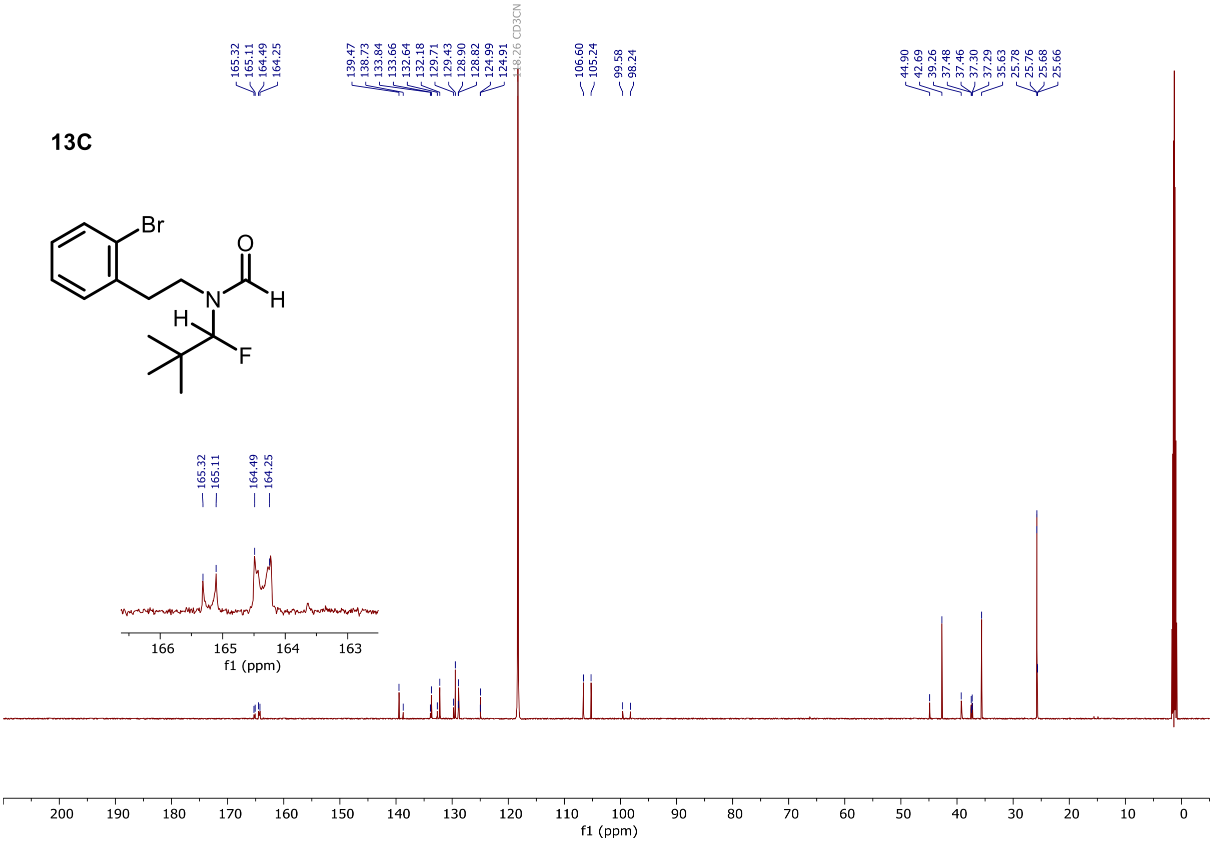

165.32
165.11
164.49
164.25
139.47
138.73
133.84
133.66
132.64
132.18
129.71
129.43
128.90
128.82
124.99
124.91
118.26 CD3CN
106.60
105.24
99.58
98.24
44.90
42.69
39.26
37.48
37.46
37.30
37.29
35.63
25.78
25.76
25.68
25.66
Br
O
N
H
H
F
f1 (ppm)

N-(9-Ethyl-9H-carbazol-3-yl)-N-(1-fluoro-2,2-dimethylpent-4-en-1-yl)formamide-d (36)

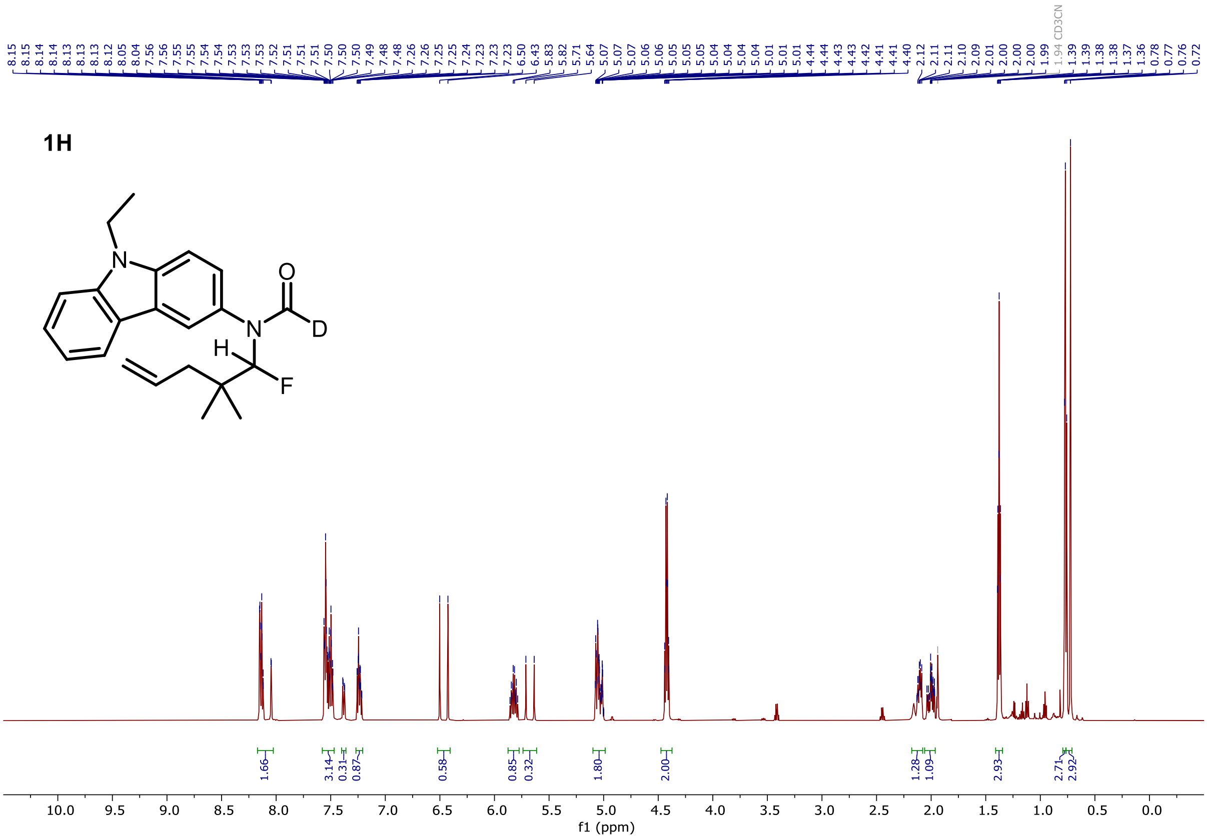

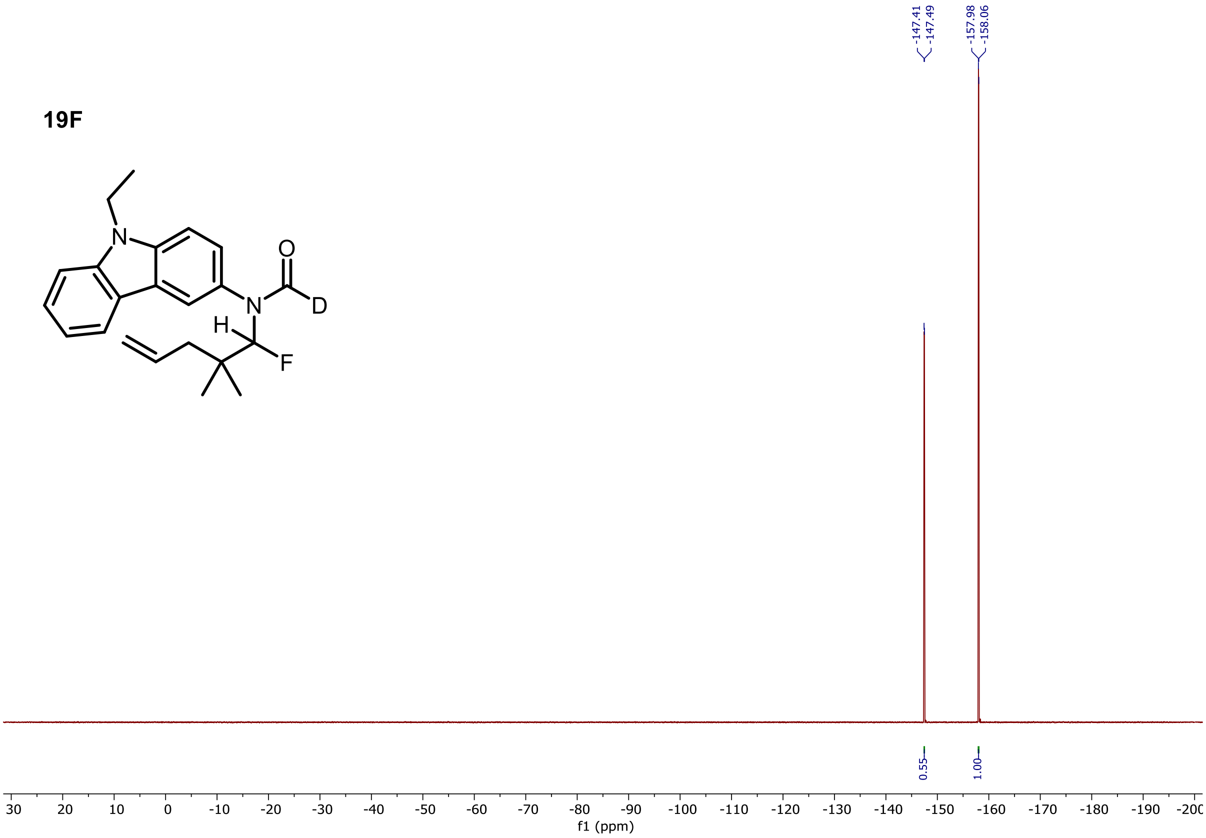

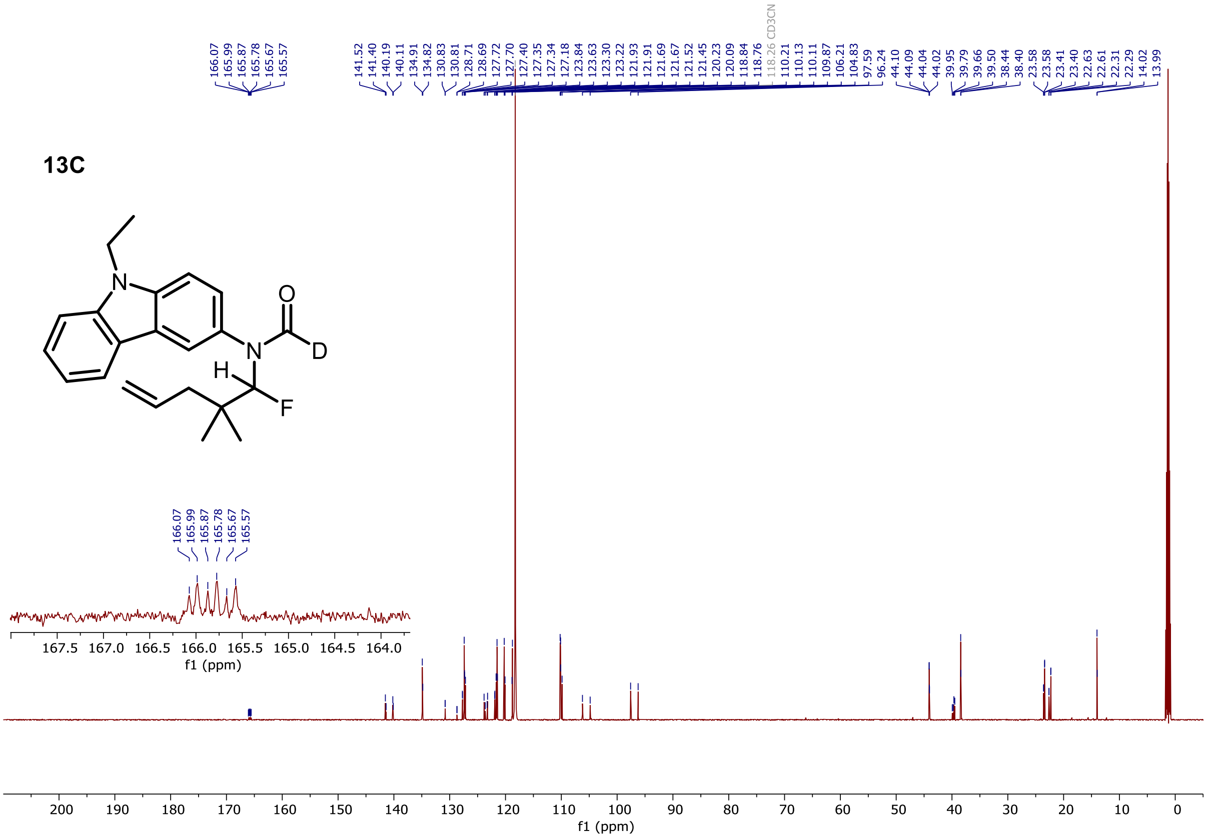

(Fluoromethyl)(3,4,5-trimethoxyphenyl)carbamoyl azide (37)

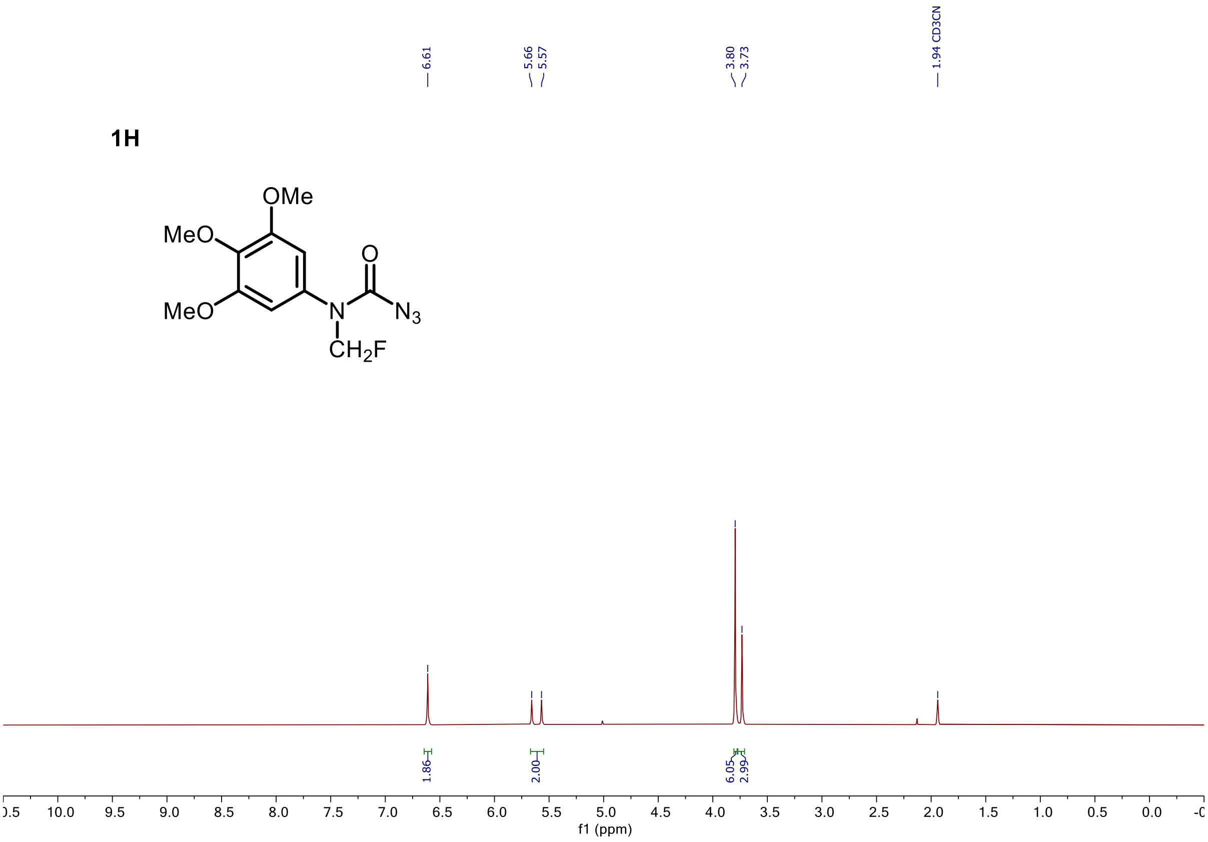

19F

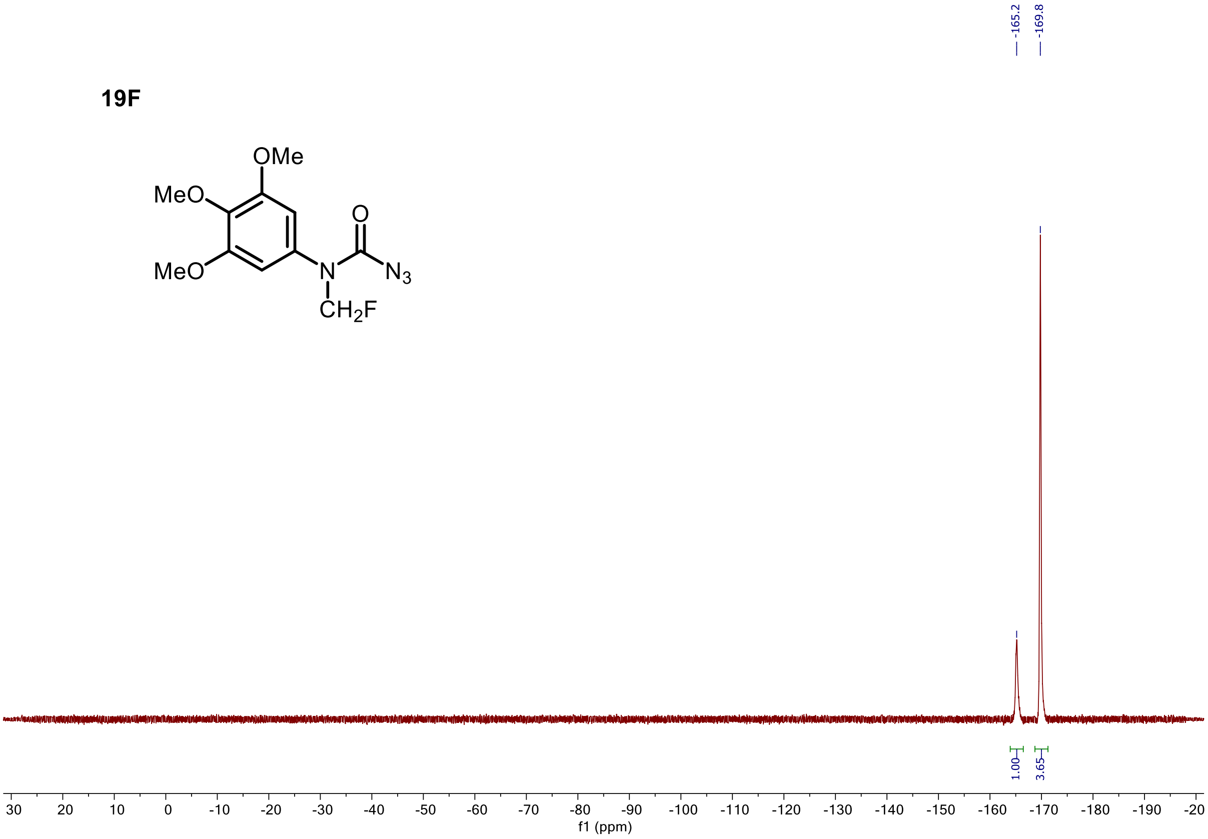
OMe
MeO
MeO
O
N
N3
CH2F
-165.2
-169.8
1.00
3.65
30 20 10 0 -10 -20 -30 -40 -50 -60 -70 -80 -90 -100 -110 -120 -130 -140 -150 -160 -170 -180 -190 -20
f1 (ppm)

13C

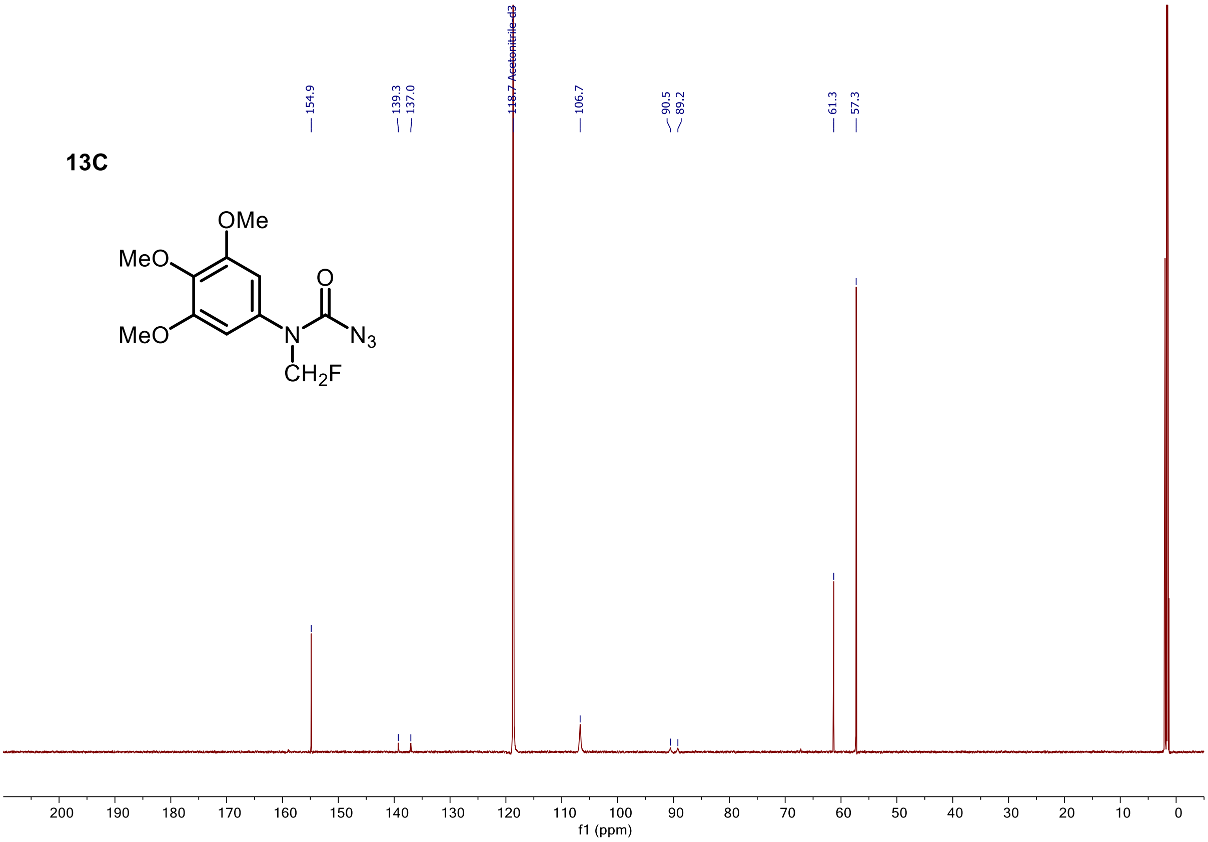
OMe
MeO
MeO
O
N
N3
CH2F
154.9
139.3
137.0
118.7 Acetonitrile-d3
106.7
90.5
89.2
61.3
57.3
200 190 180 170 160 150 140 130 120 110 100 90 80 70 60 50 40 30 20 10 0
f1 (ppm)

(1-(2,6-Dimethylphenoxy)propan-2-yl)(fluoromethyl)carbamoyl azide (38)

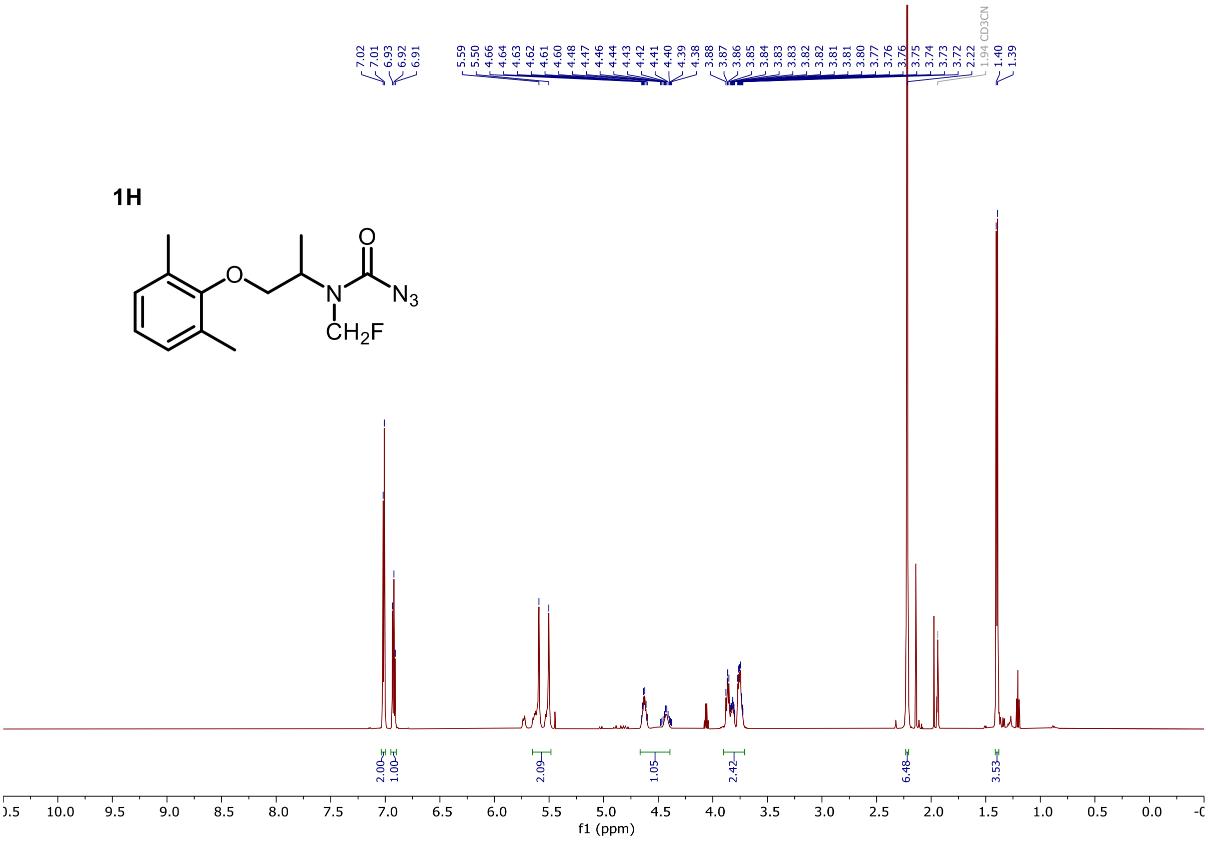

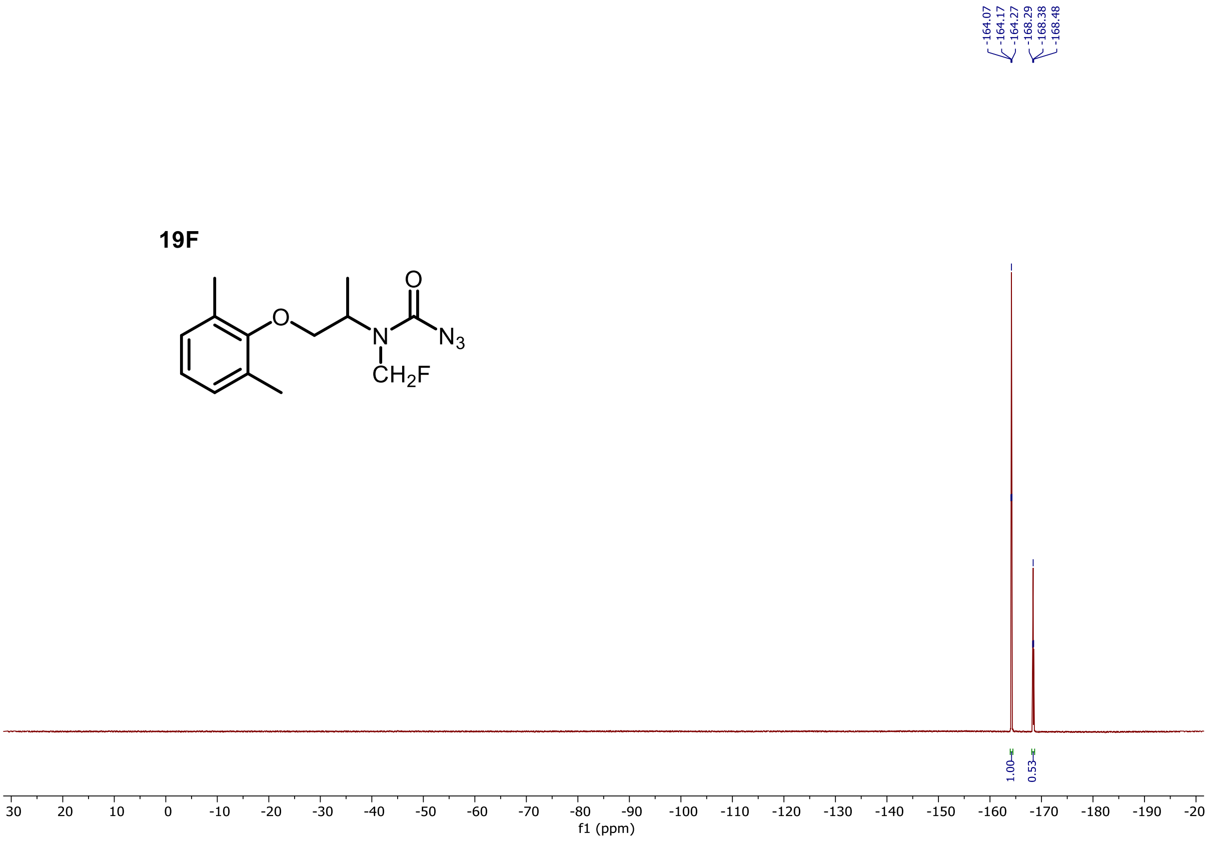

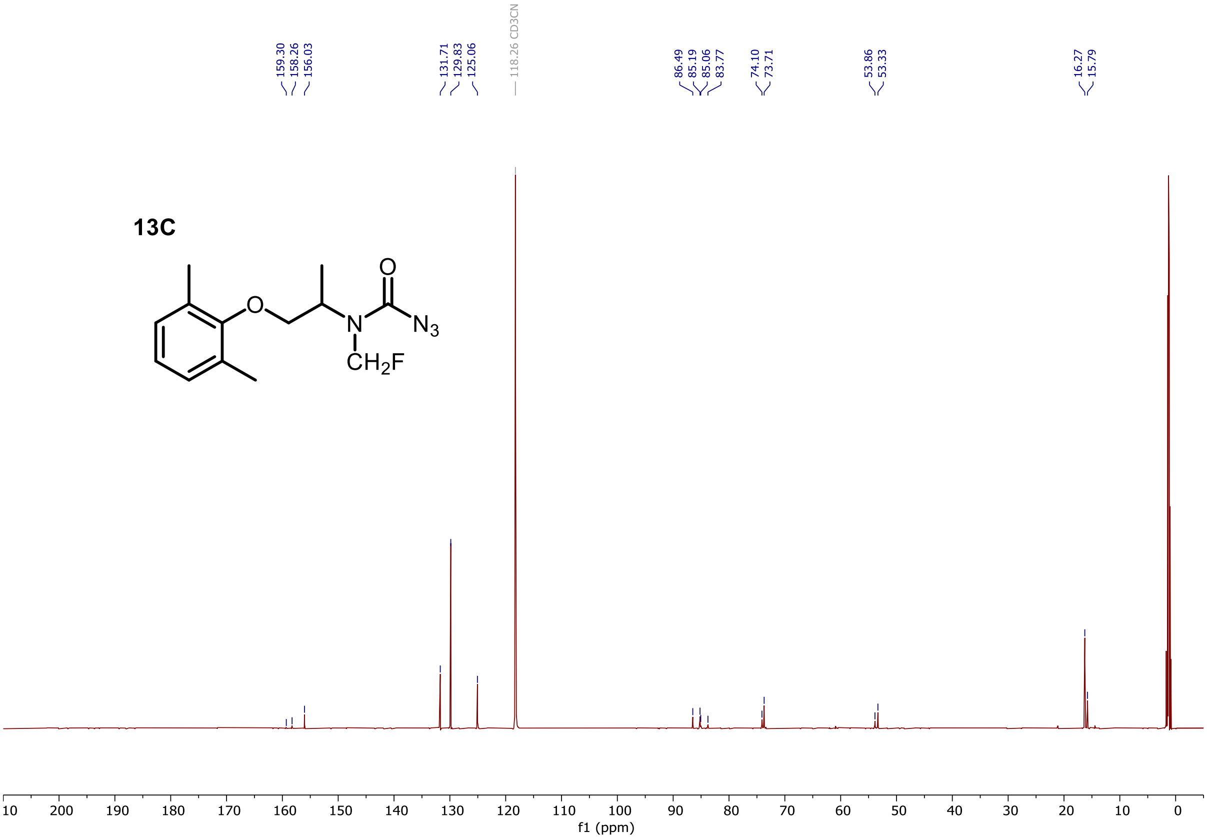

((4-Chlorophenyl)fluoromethyl)(cyclohexyl)carbamoyl azide (39)

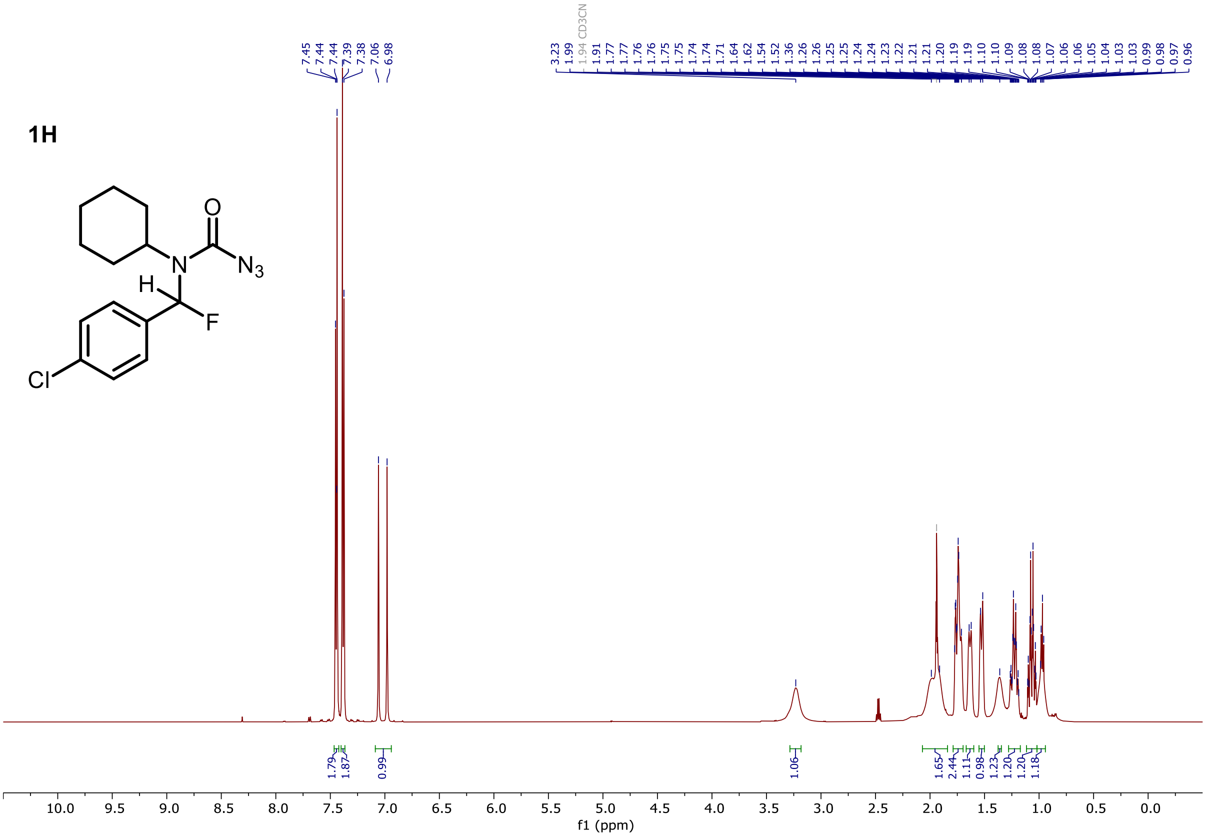

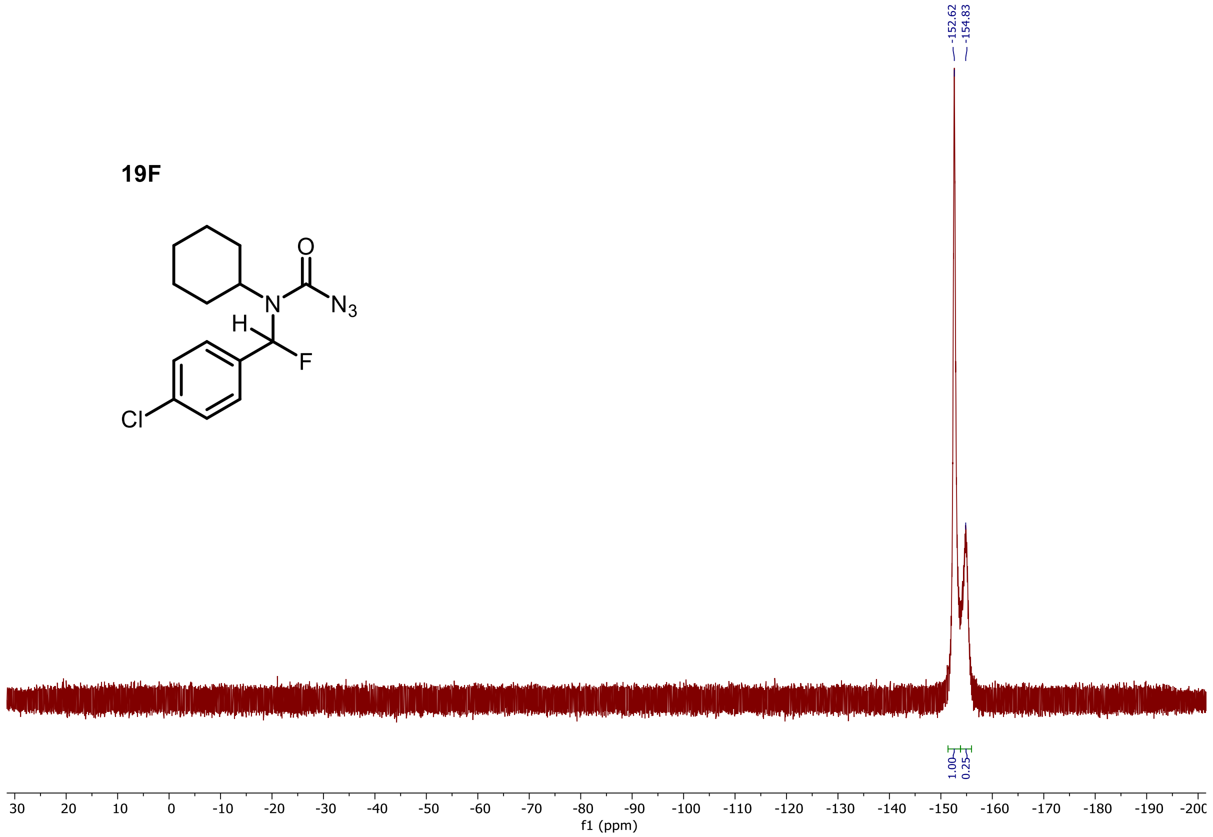
19F
-152.62
-154.83
1.00
0.25
30 20 10 0 -10 -20 -30 -40 -50 -60 -70 -80 -90 -100 -110 -120 -130 -140 -150 -160 -170 -180 -190 -200
f1 (ppm)

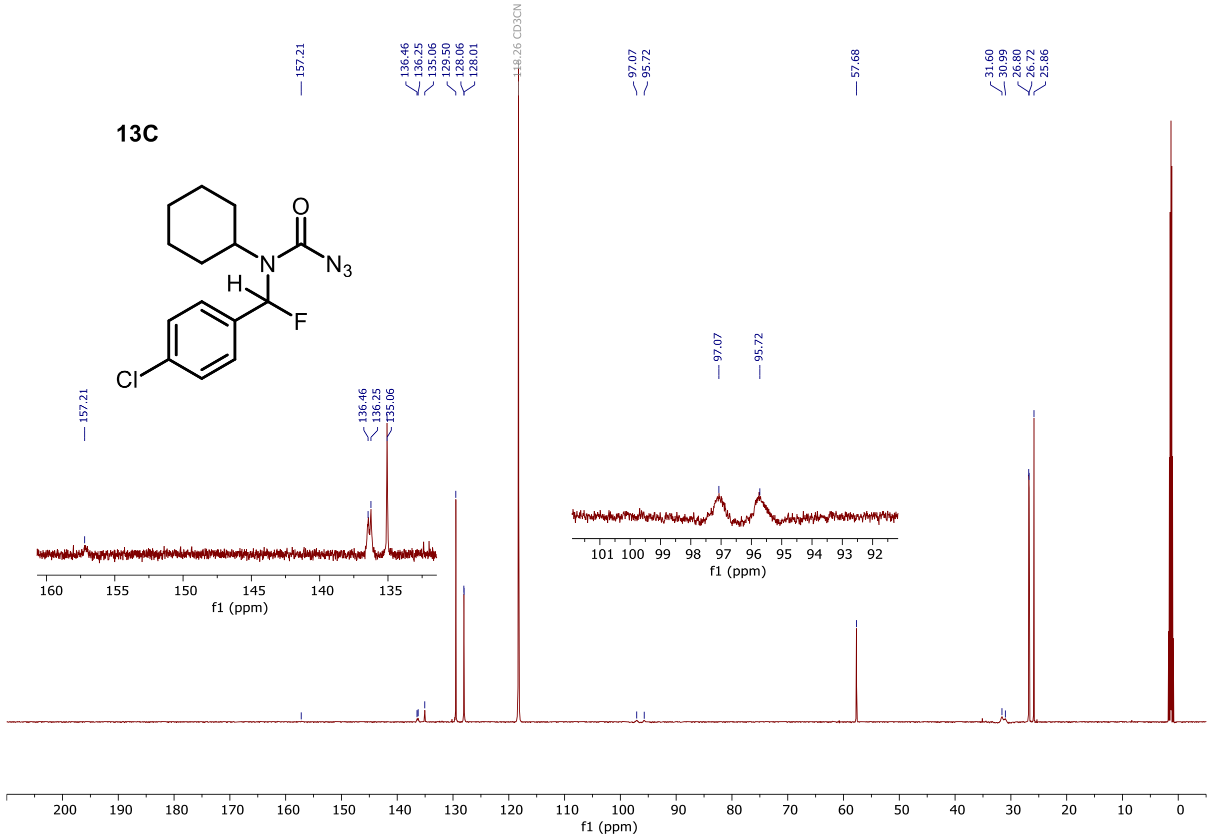
13C
157.21
136.46
136.25
135.06
129.50
128.06
128.01
118.26 CD3CN
97.07
95.72
57.68
31.60
30.99
26.80
26.72
25.86
160 155 150 145 140 135
f1 (ppm)
101 100 99 98 97 96 95 94 93 92
f1 (ppm)
200 190 180 170 160 150 140 130 120 110 100 90 80 70 60 50 40 30 20 10 0
f1 (ppm)

(Fluoromethyl)(2-methoxy-5-methylphenyl)carbamoyl cyanide (40)

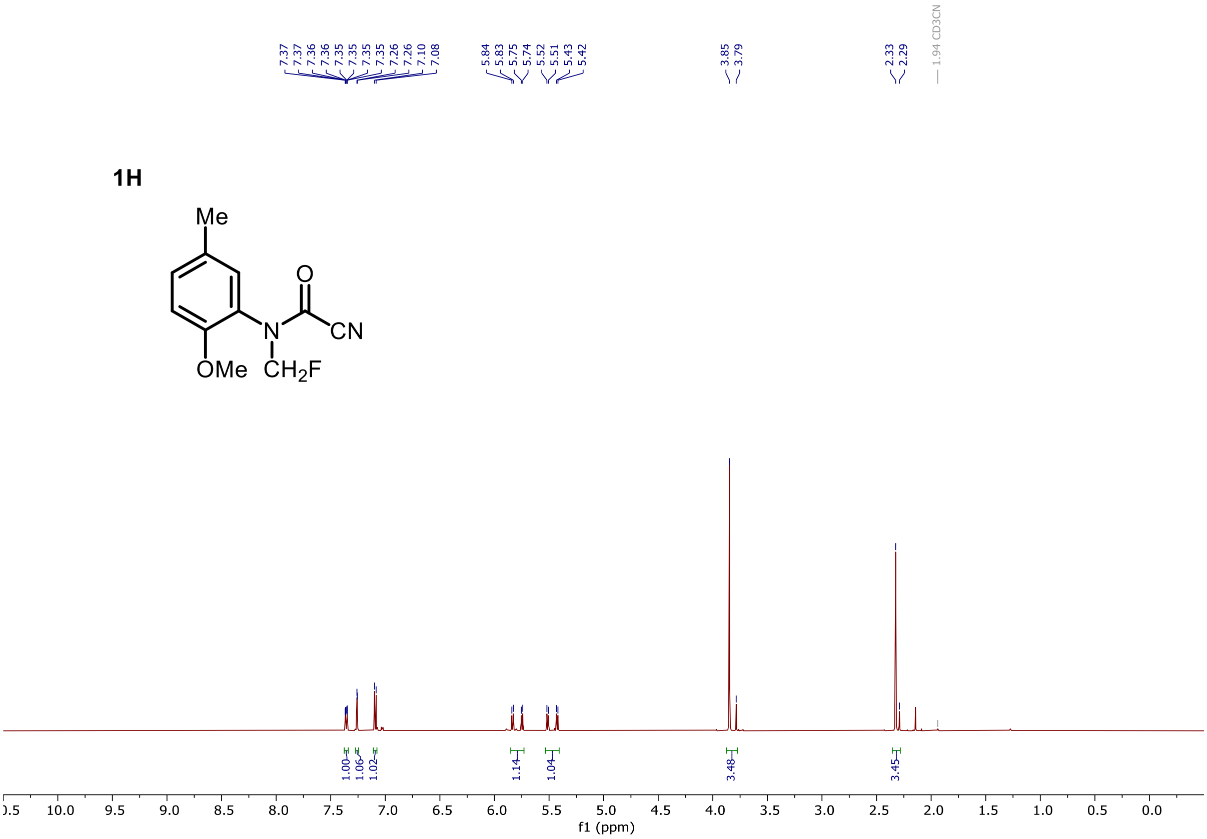

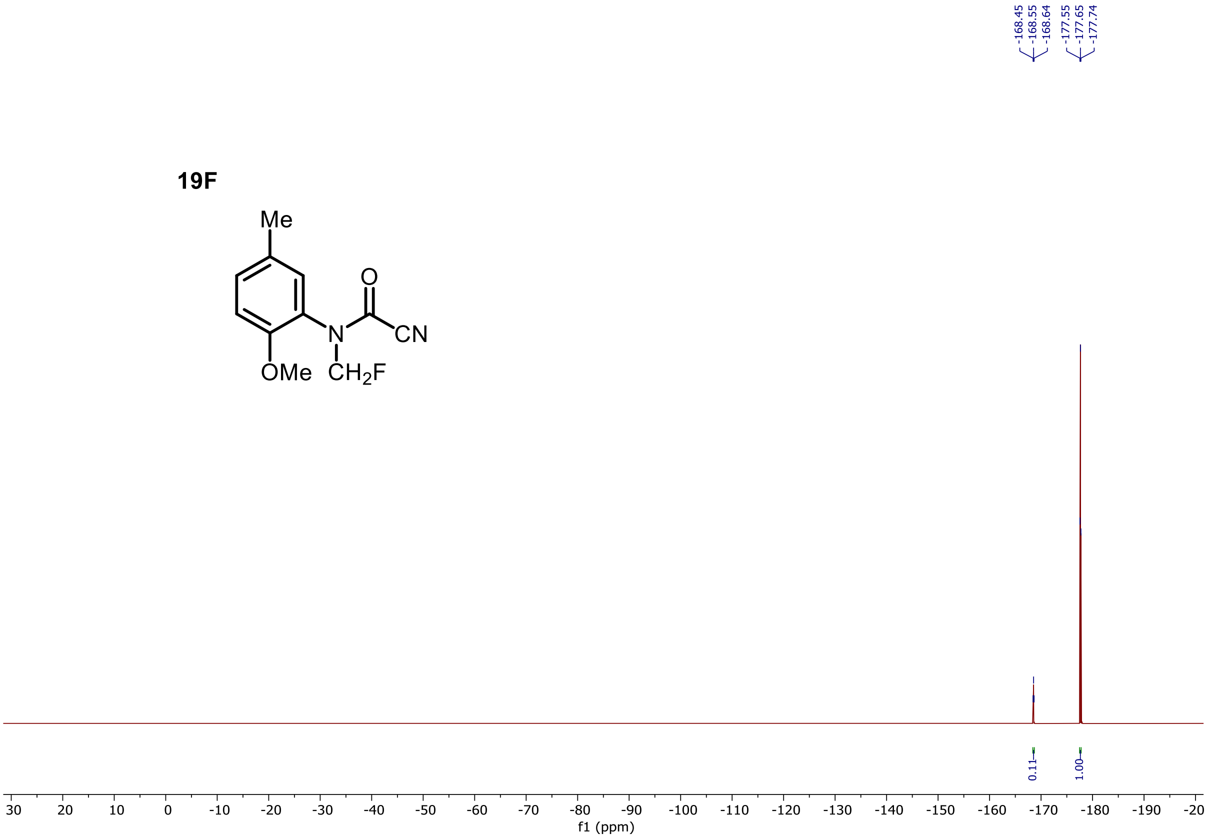

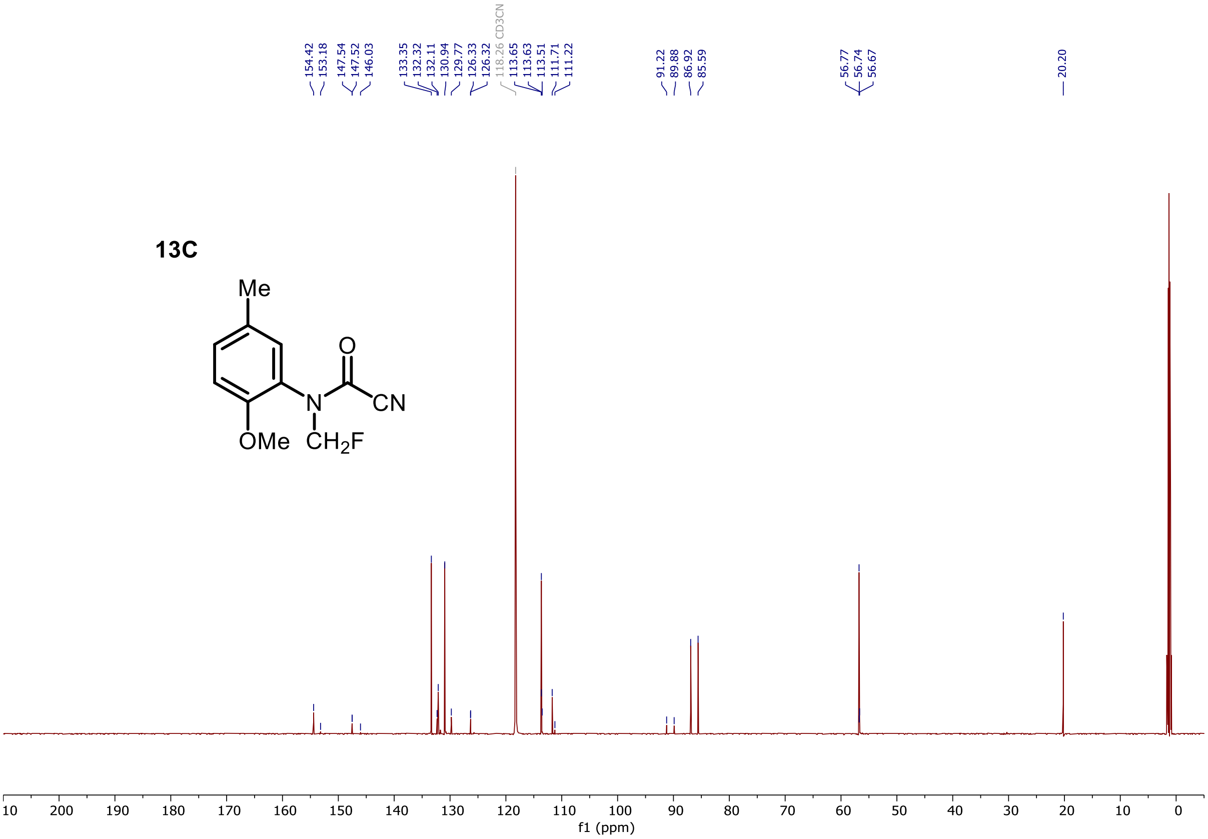

Methyl *N*-(cyanocarbonyl)-*N*-(fluoromethyl)-*L*-phenylalaninate (41)

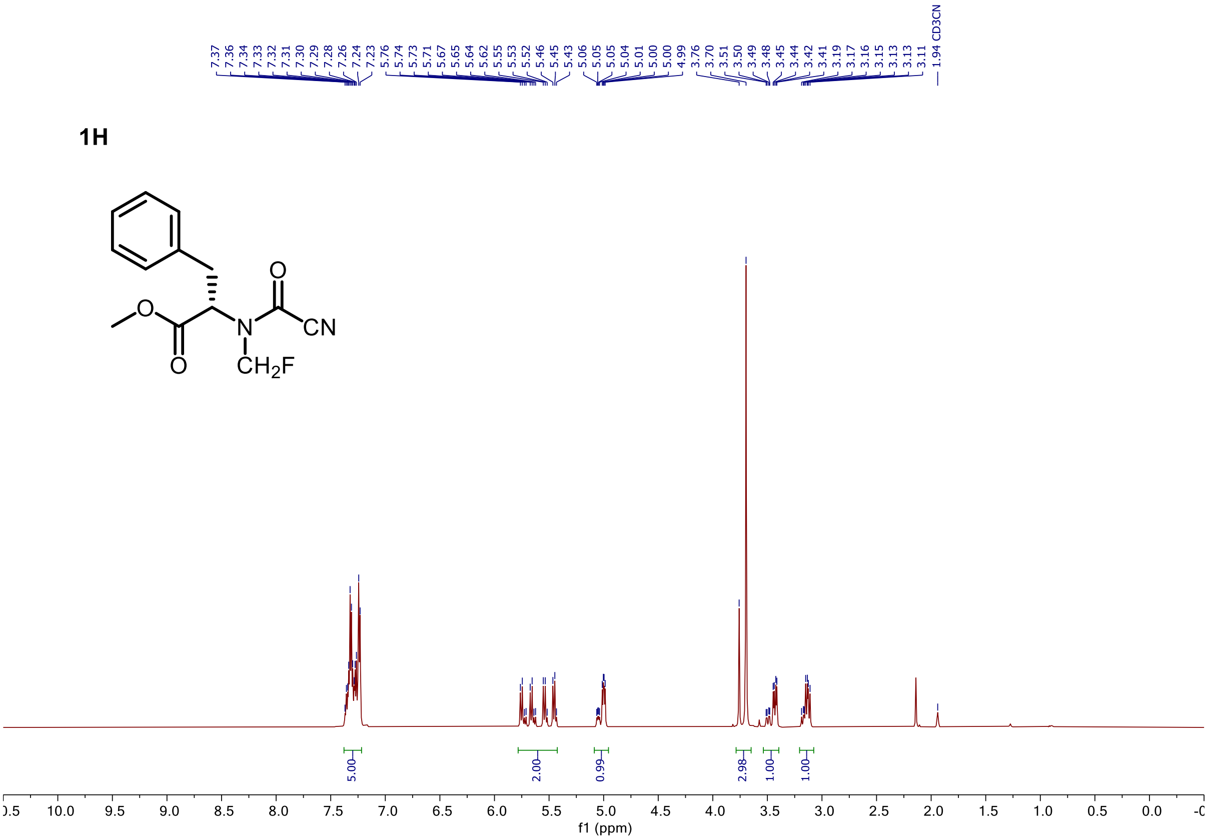

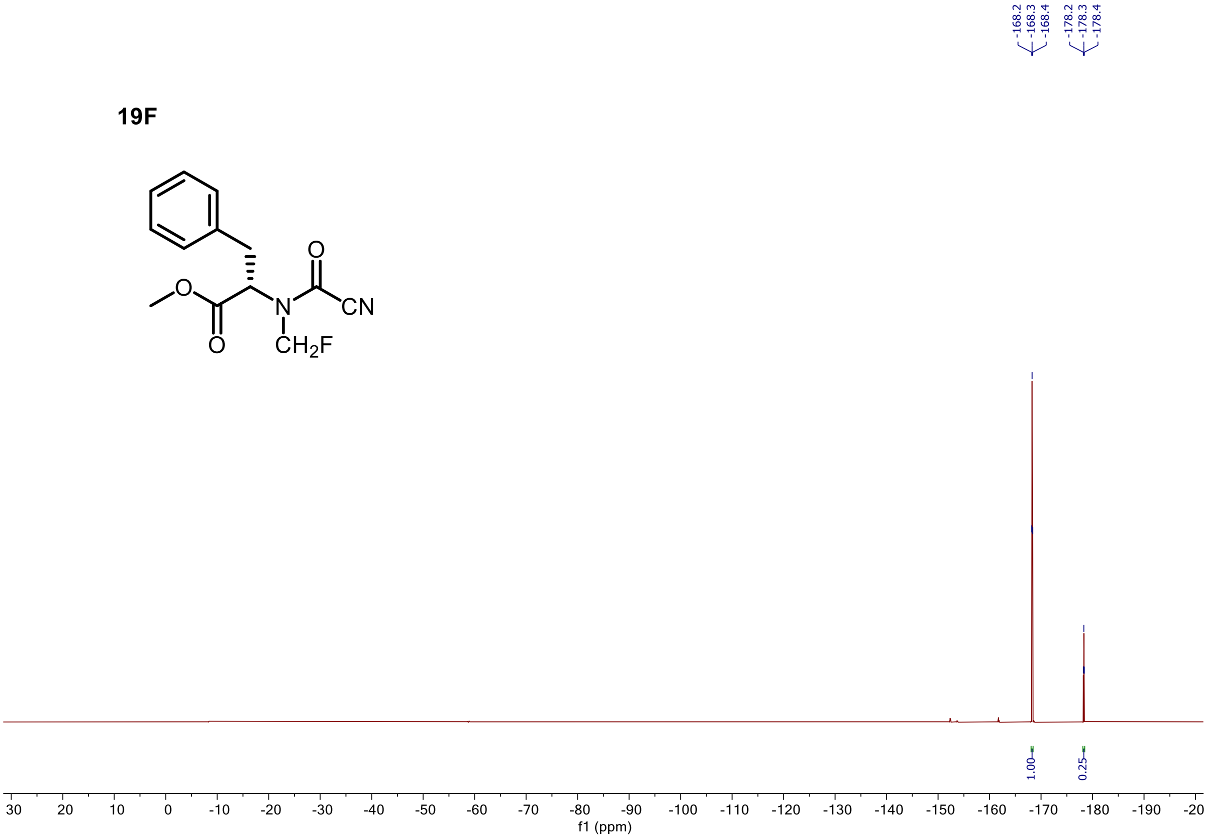

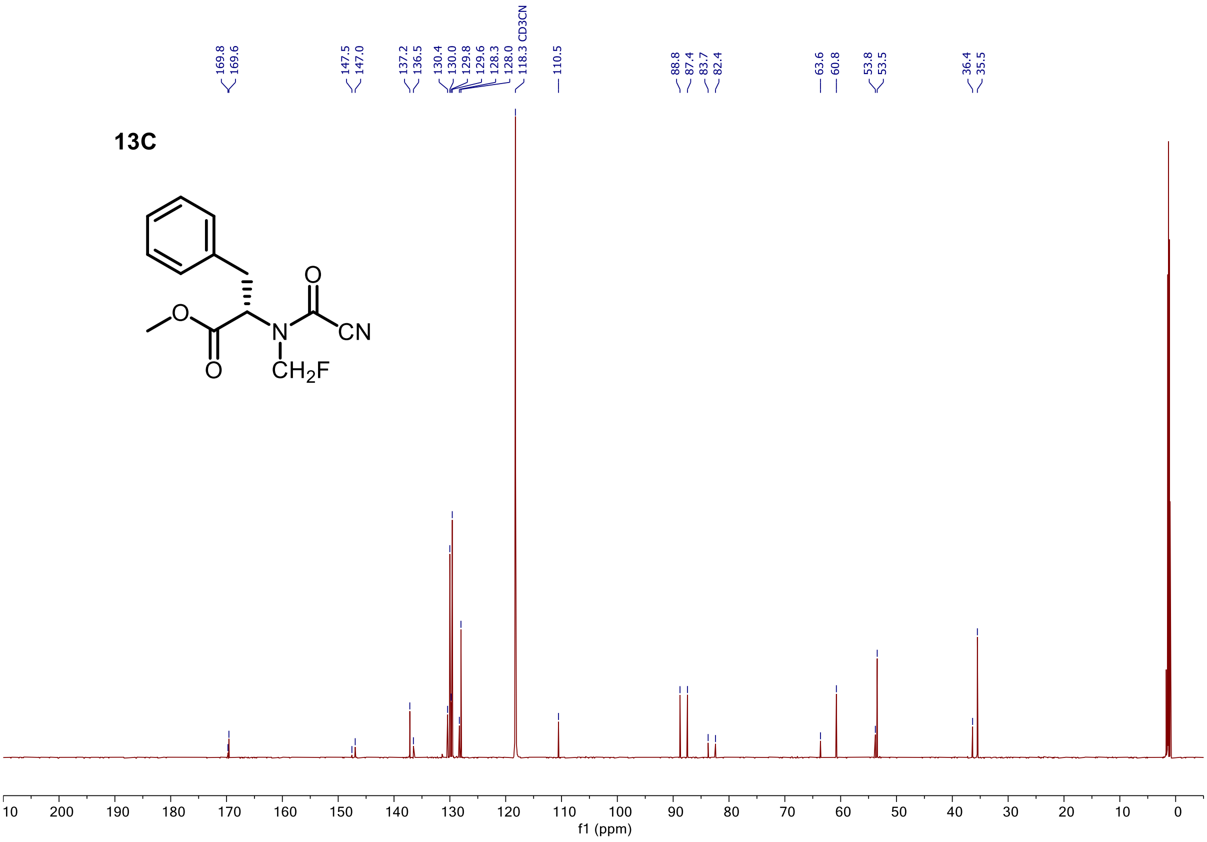

(9-Ethyl-9*H*-carbazol-3-yl)(1-fluoro-2,2-dimethylpent-4-en-1-yl)carbamoyl cyanide (42)

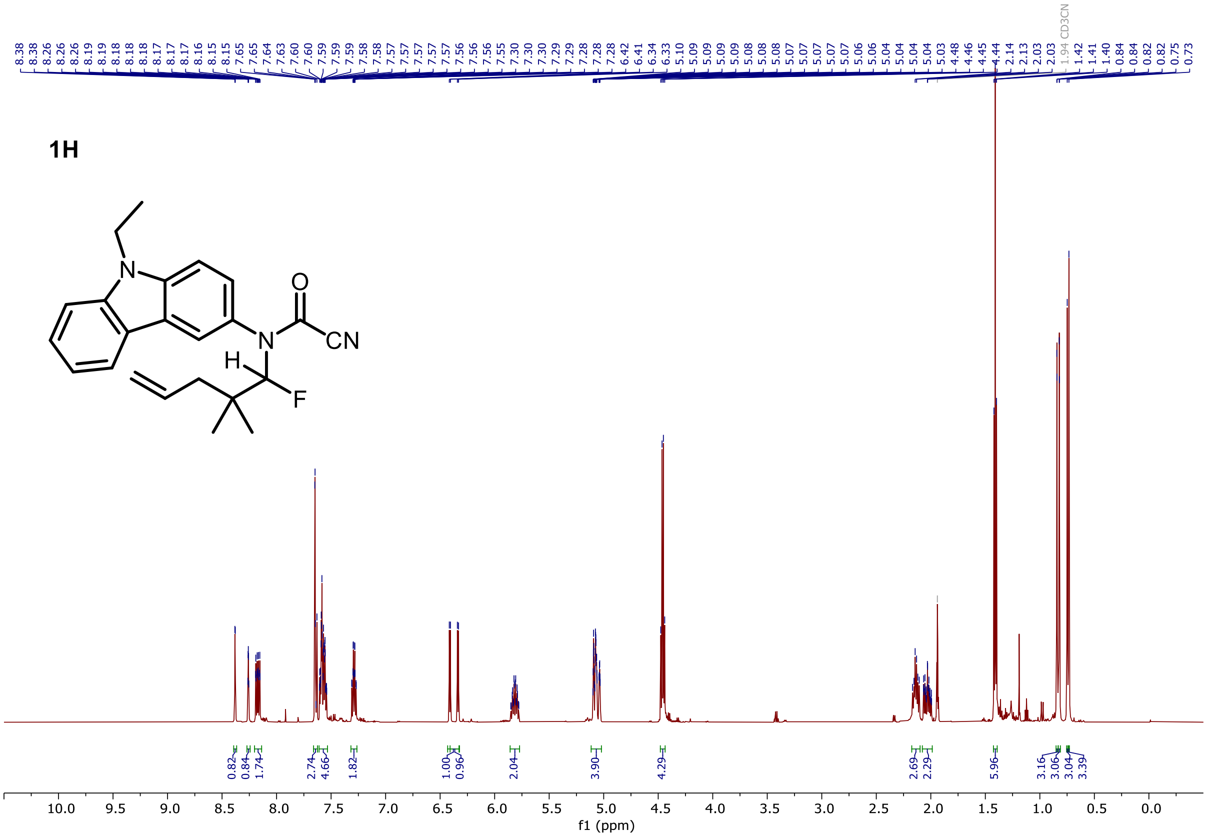
8.38 8.38 8.26 8.26 8.19 8.19 8.18 8.18 8.17 8.17 8.16 8.15 7.65 7.65 7.64 7.63 7.60 7.59 7.59 7.59 7.58 7.58 7.57 7.57 7.57 7.57 7.56 7.56 7.55 7.30 7.30 7.29 7.29 7.28 7.28 6.42 6.41 6.34 6.33 5.10 5.09 5.09 5.09 5.09 5.08 5.08 5.07 5.07 5.07 5.07 5.06 5.04 5.04 5.04 5.03 4.48 4.46 4.45 4.44 2.14 2.13 2.03 1.94 CD3CN 1.42 1.41 1.40 0.84 0.84 0.82 0.75 0.73
1H
N
O
N
CN
H
F
0.82 0.84 1.74 2.74 4.66 1.82 1.00 0.96 2.04 3.90 4.29 2.69 2.29 5.96 3.16 3.06 3.04 3.39
10.0 9.5 9.0 8.5 8.0 7.5 7.0 6.5 6.0 5.5 5.0 4.5 4.0 3.5 3.0 2.5 2.0 1.5 1.0 0.5 0.0
f1 (ppm)

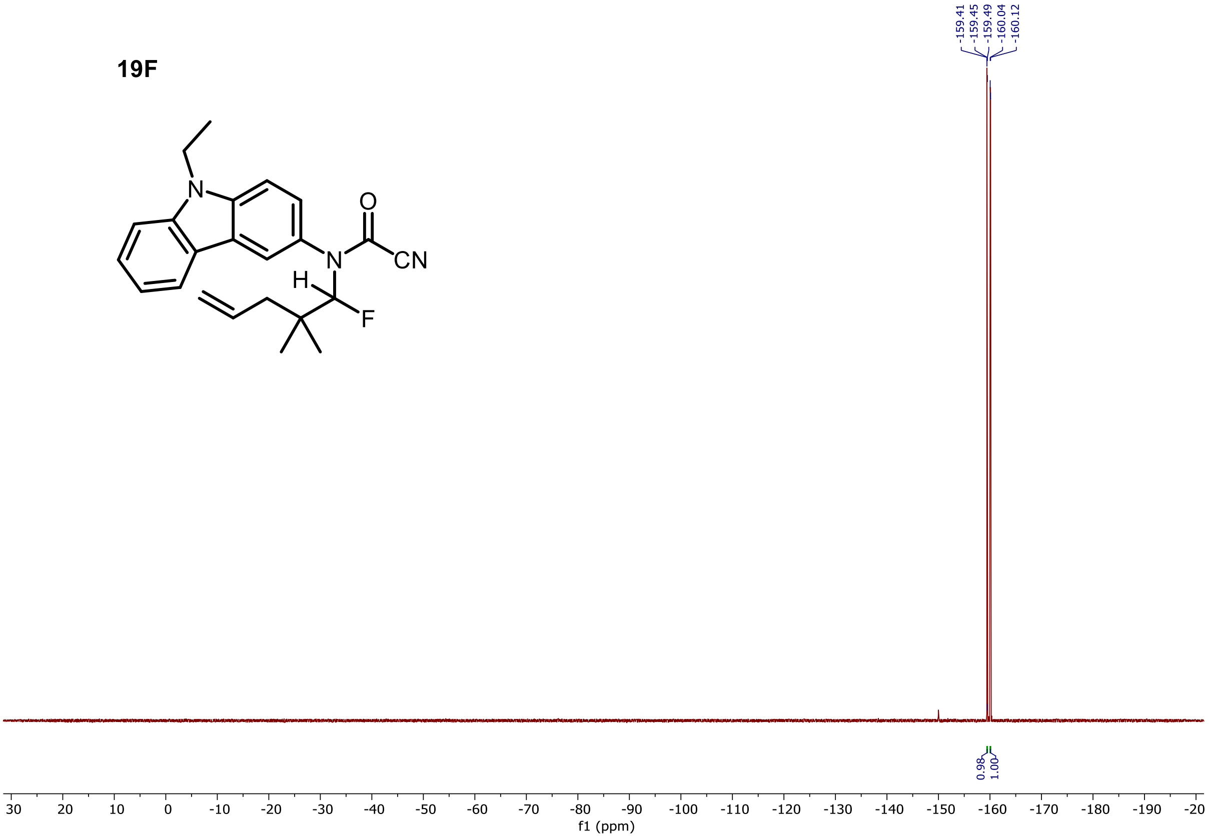
-159.41 -159.45 -159.49 -160.04 -160.12
19F
N
O
N
CN
H
F
0.98 1.00
30 20 10 0 -10 -20 -30 -40 -50 -60 -70 -80 -90 -100 -110 -120 -130 -140 -150 -160 -170 -180 -190 -20
f1 (ppm)

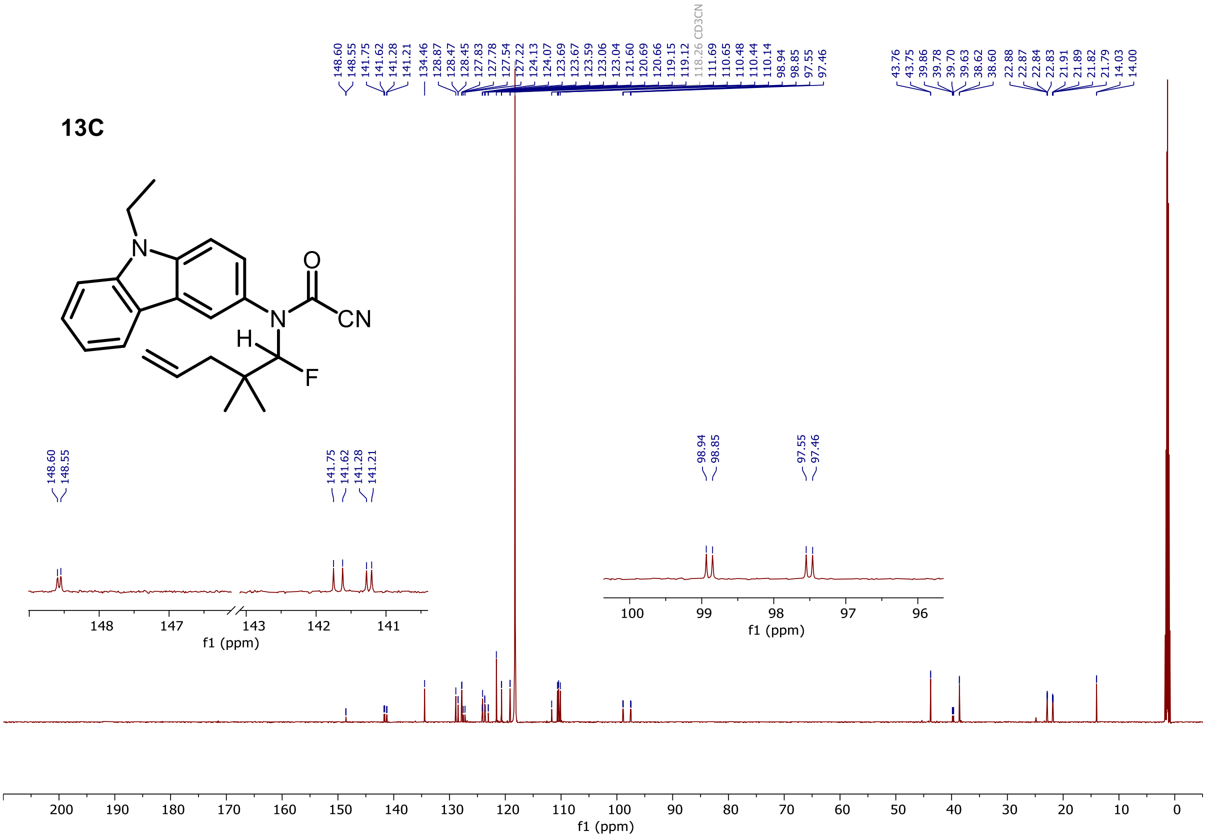

N-([1,1'-Biphenyl]-4-yl)-*N*-(fluoromethyl)-3-phenylpropiolamide (43)

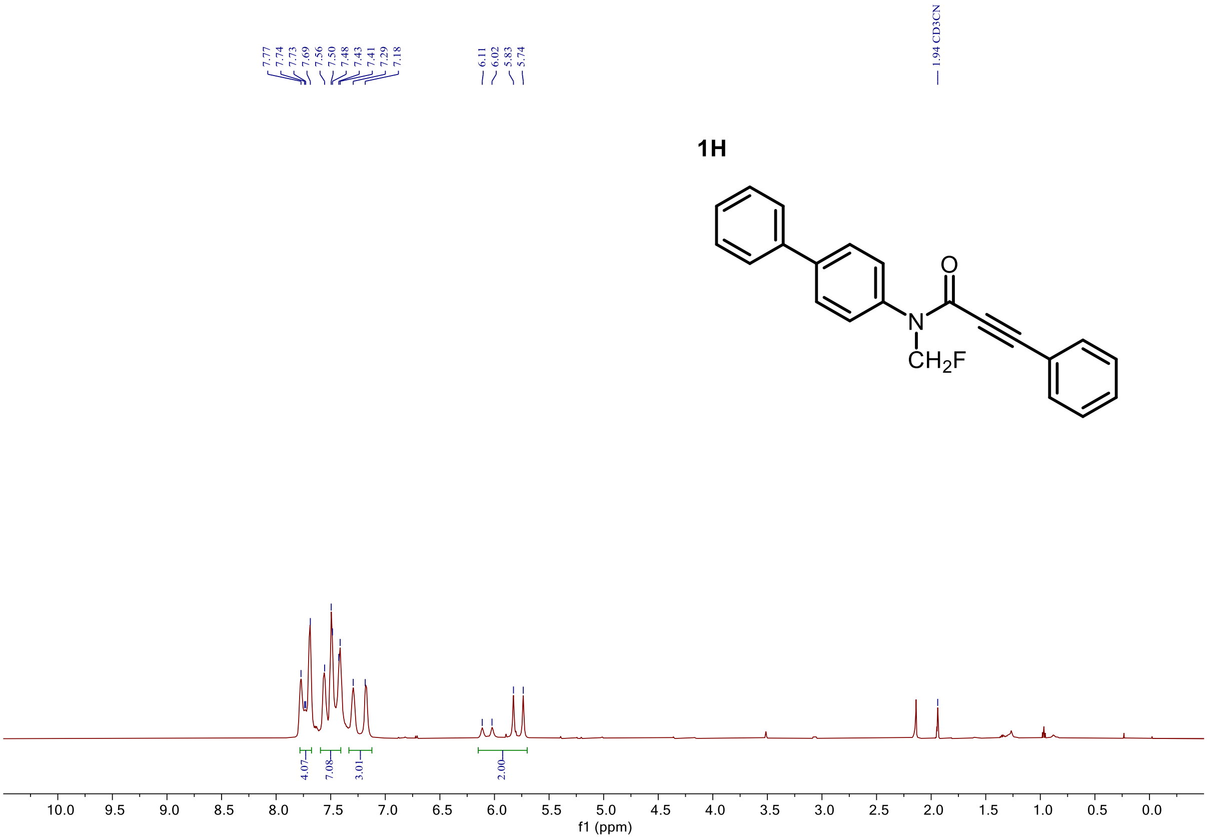

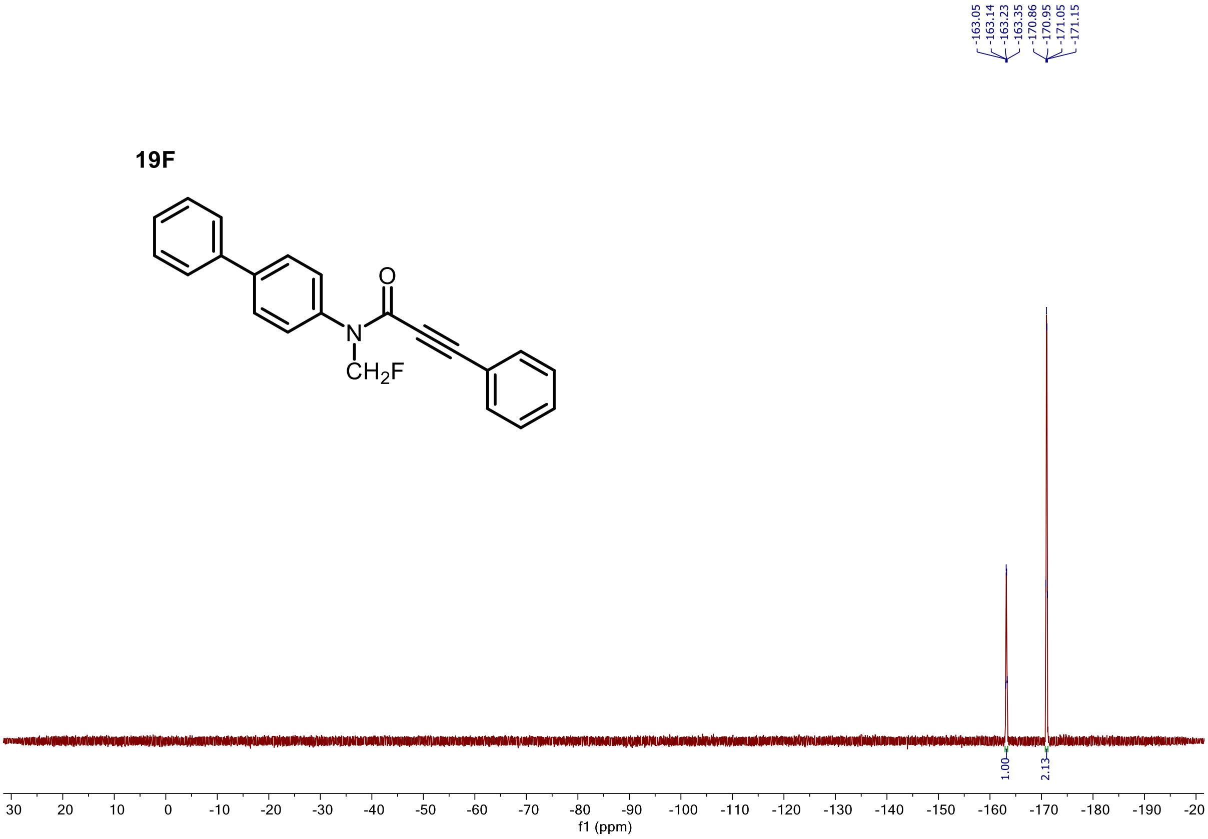

19F
O
N
CH2F
-163.05
-163.14
-163.23
-163.35
-170.86
-170.95
-171.05
-171.15
1.00
2.13
30 20 10 0 -10 -20 -30 -40 -50 -60 -70 -80 -90 -100 -110 -120 -130 -140 -150 -160 -170 -180 -190 -20
f1 (ppm)

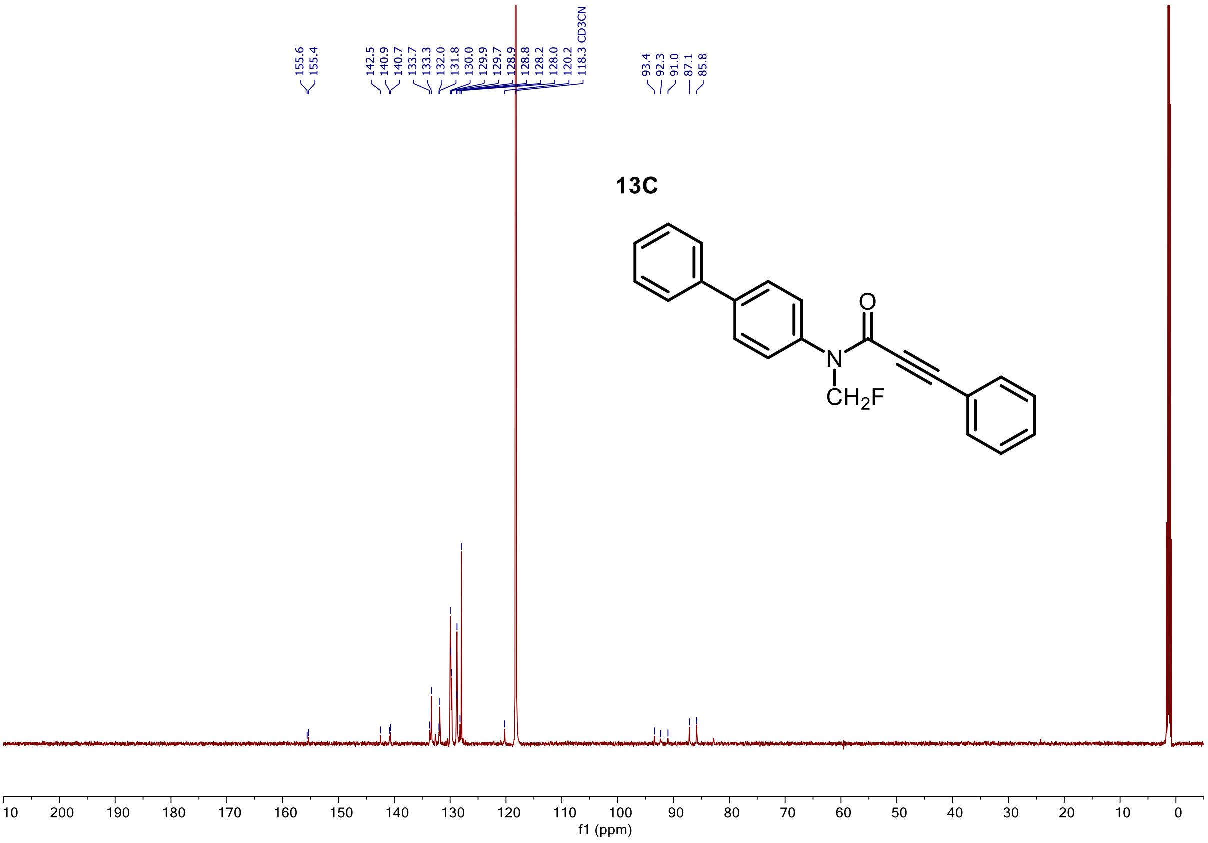

155.6
155.4
142.5
140.9
140.7
133.7
133.3
132.0
131.8
130.0
129.9
129.7
128.9
128.8
128.2
128.0
120.2
118.3 CD3CN
93.4
92.3
91.0
87.1
85.8
13C
O
N
CH2F
10 200 190 180 170 160 150 140 130 120 110 100 90 80 70 60 50 40 30 20 10 0
f1 (ppm)

N-(2-Bromophenethyl)-3-(4-bromophenyl)-*N*-(fluoromethyl)propiolamide (44)

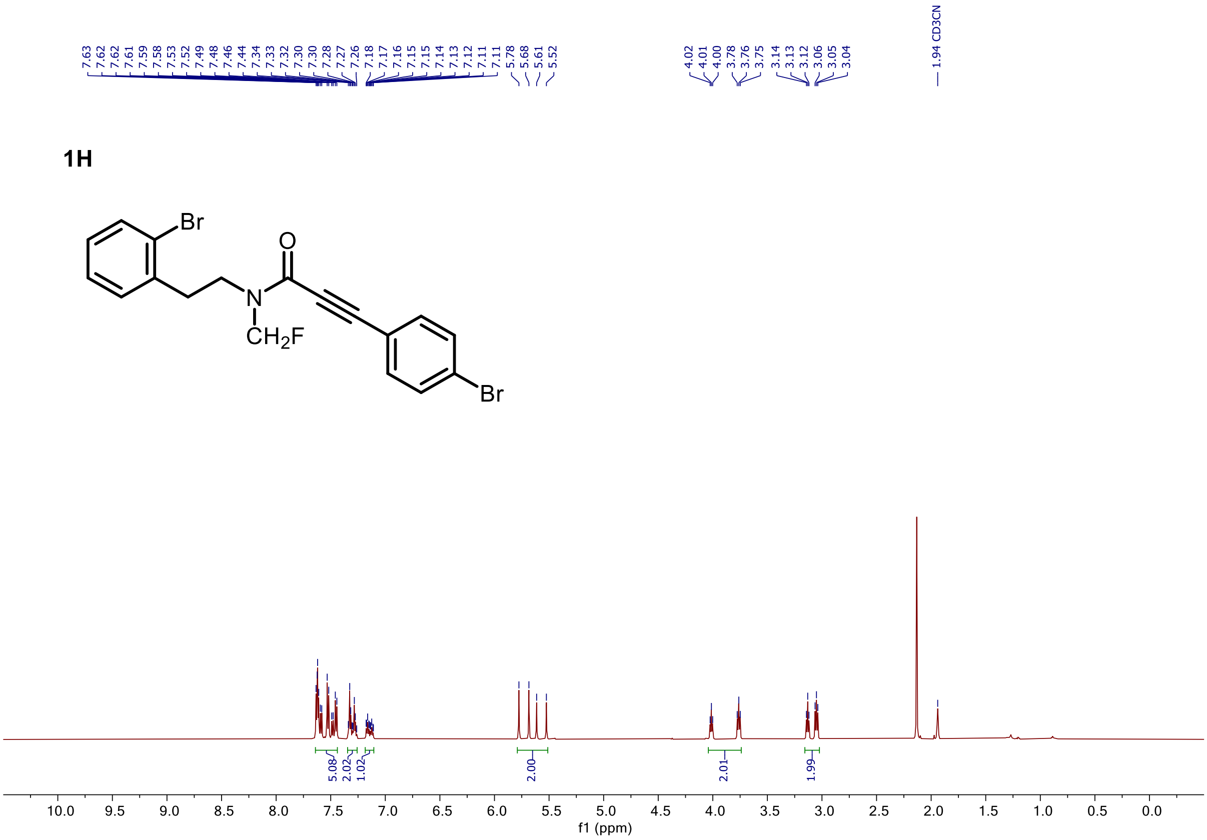

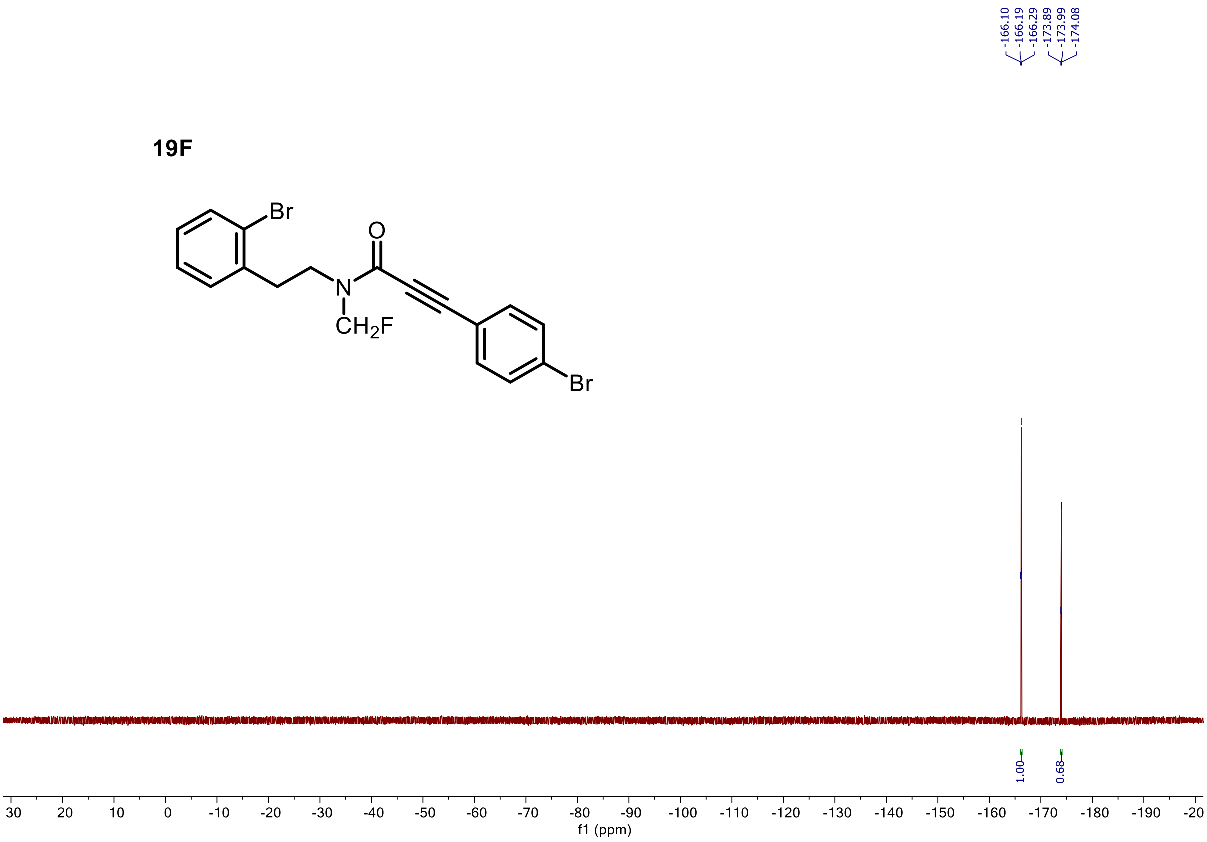

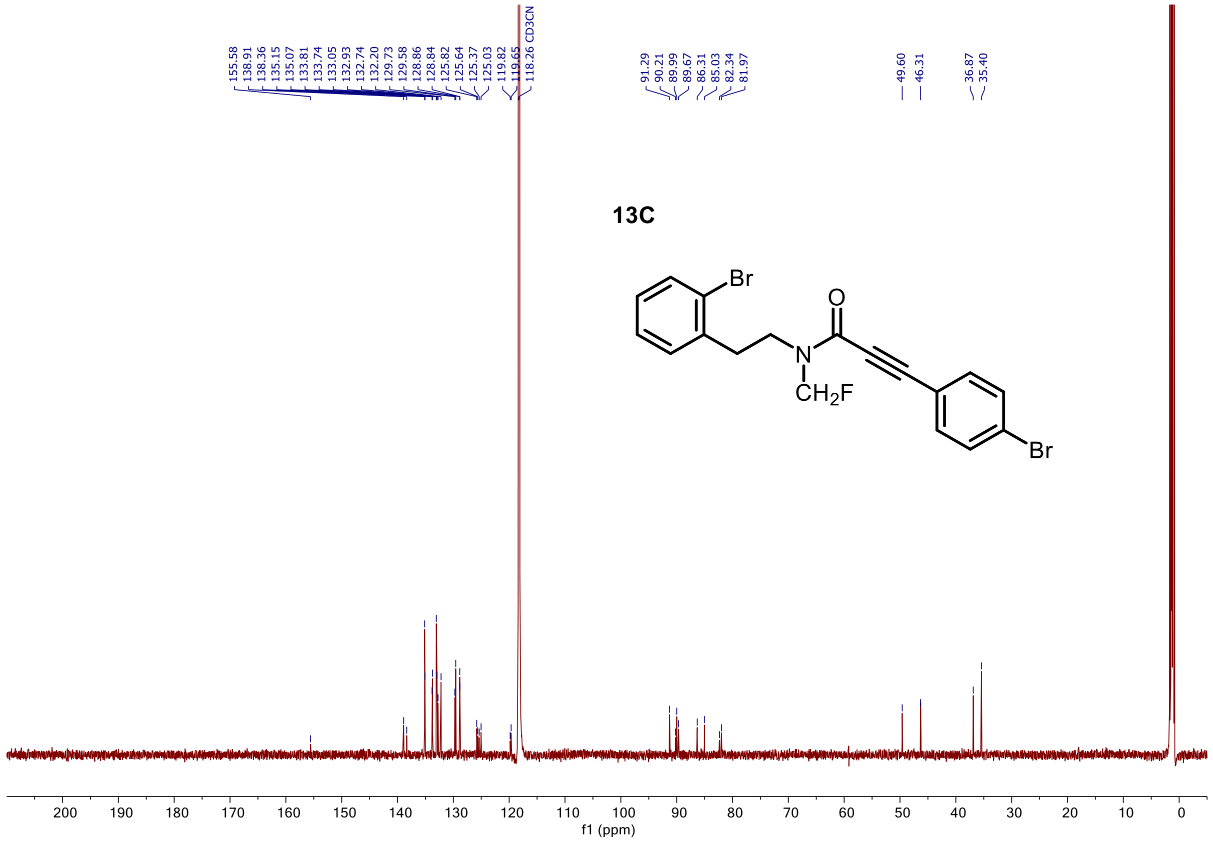

N-(Fluoro(phenyl)methyl)-N-phenyl-3-(3-(trifluoromethyl)phenyl)propiolamide (45)

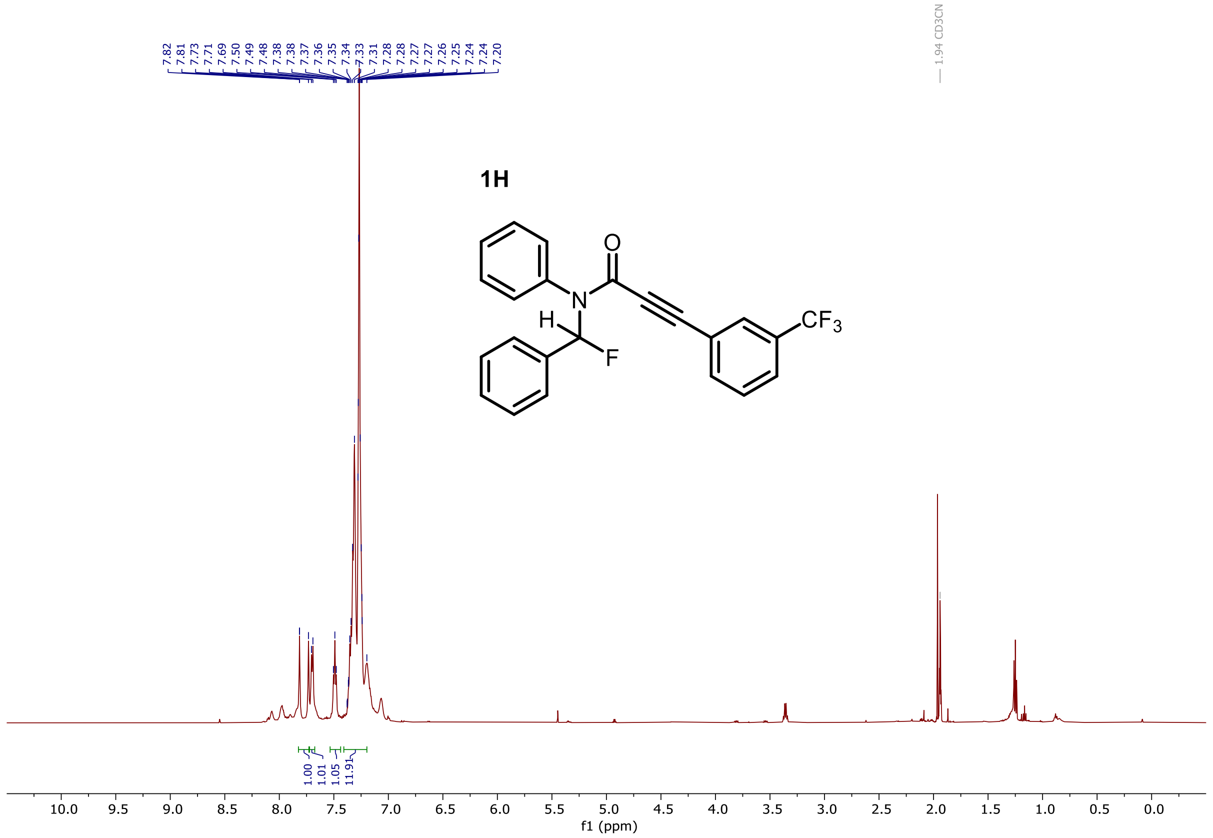

19F

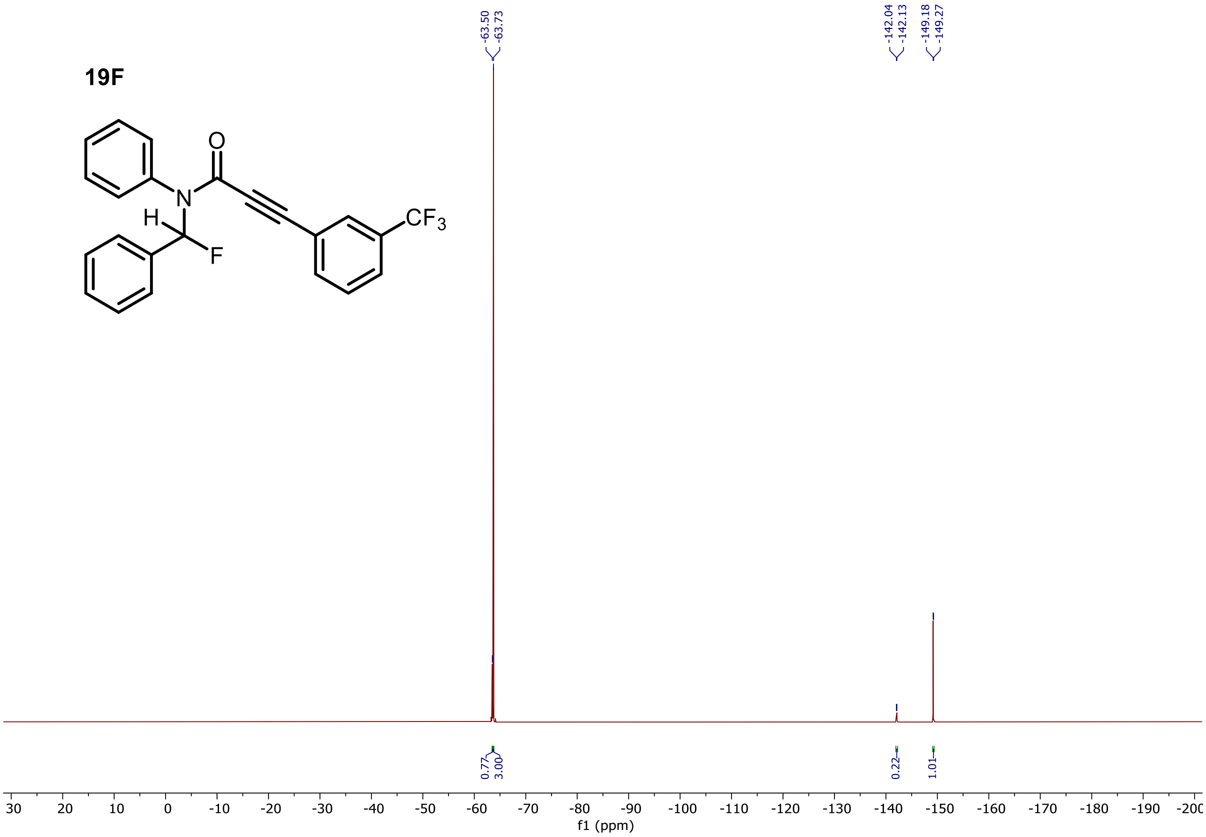
-63.50
-63.73
-142.04
-142.13
-149.18
-149.27
O
N
H
F
CF3
0.77
3.00
0.22
1.01
f1 (ppm)

13C

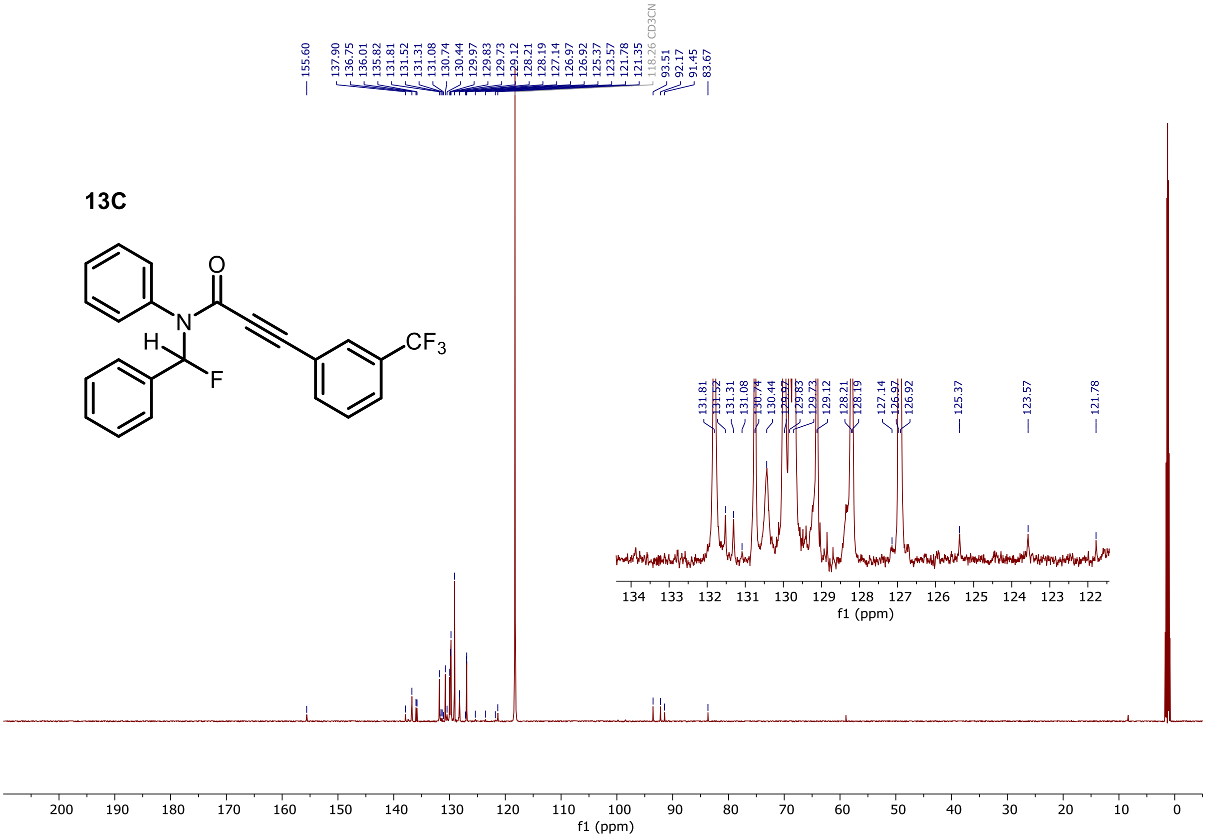
155.60
137.90
136.75
136.01
135.82
131.81
131.52
131.31
131.08
130.74
130.44
129.97
129.83
129.73
129.12
128.21
128.19
127.14
126.97
126.92
125.37
123.57
121.78
121.35
118.26 CD3CN
93.51
92.17
91.45
83.67
O
N
H
F
CF3
f1 (ppm)

***N*-Allyl-*N*-(fluoro(phenyl)methyl)-3-(thiophen-2-yl)propiolamide (46)**

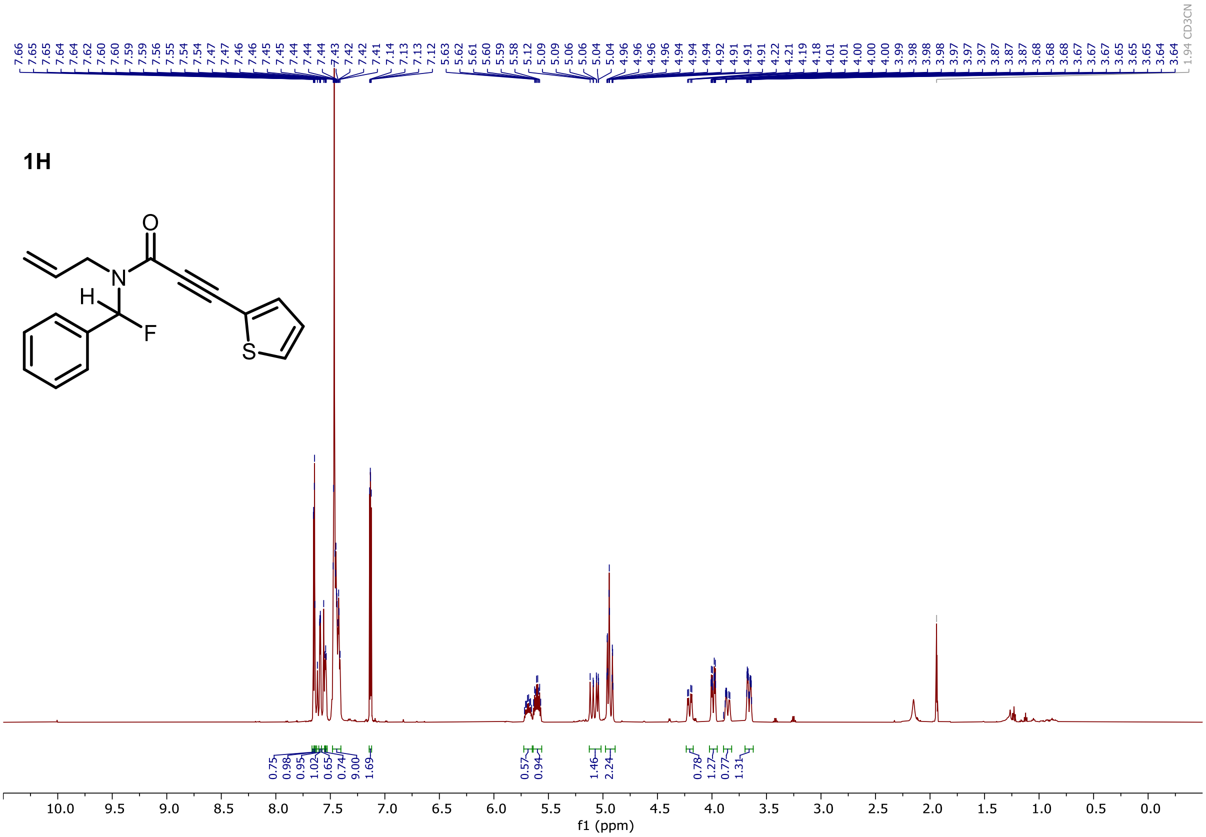

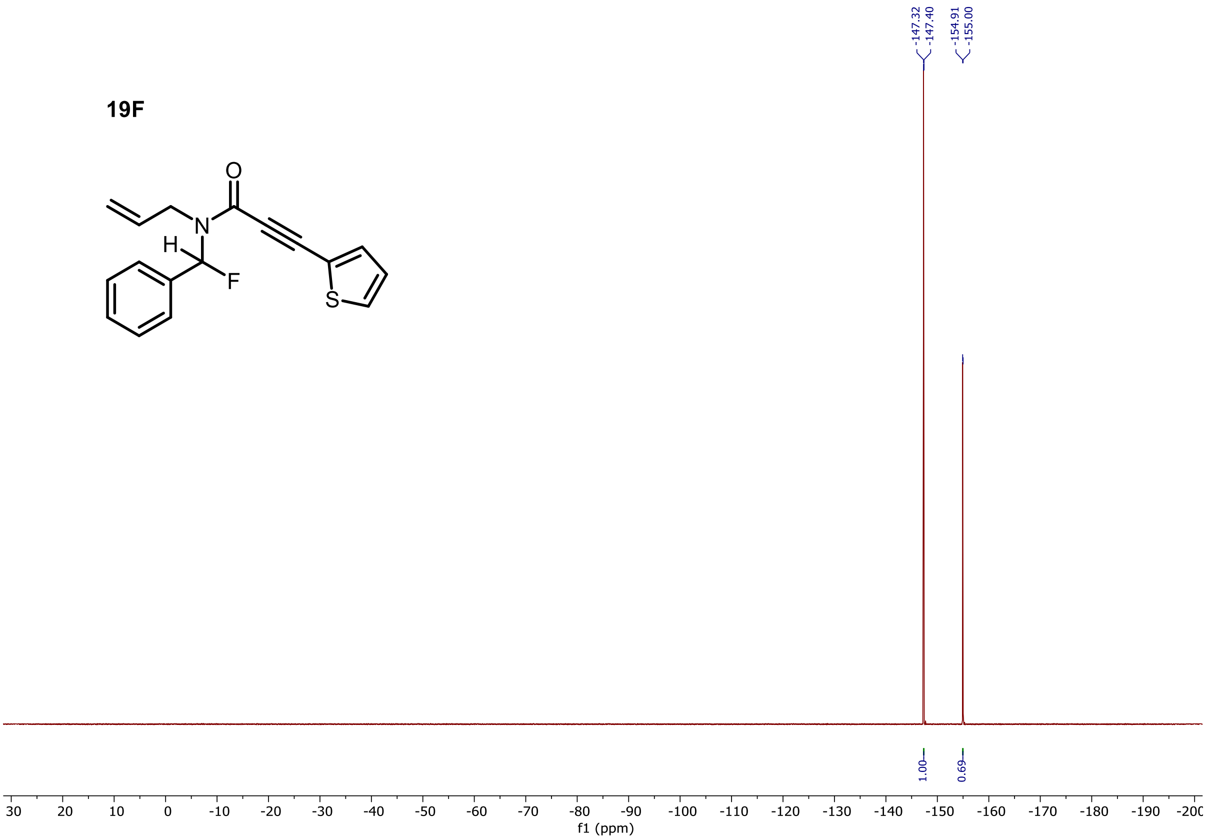

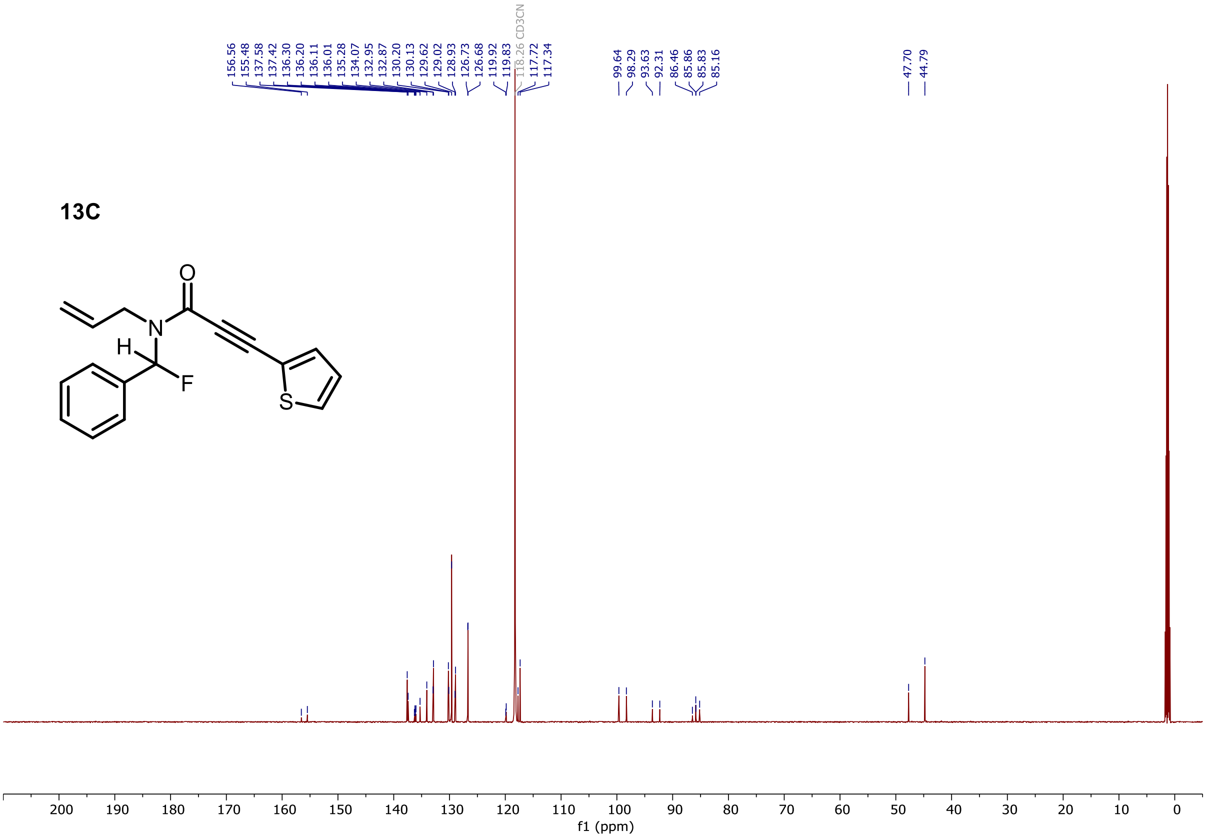
13C
O
N
H
F
S
156.56
155.48
137.58
137.42
136.30
136.20
136.11
136.01
135.28
134.07
132.95
132.87
130.20
130.13
129.62
129.02
128.93
126.73
126.68
119.92
119.83
118.26 CD3CN
117.72
117.34
99.64
98.29
93.63
92.31
86.46
85.86
85.83
85.16
47.70
44.79
200 190 180 170 160 150 140 130 120 110 100 90 80 70 60 50 40 30 20 10 0
f1 (ppm)

10.3. Aldimines

N-Diphenylmethanimine (S1)

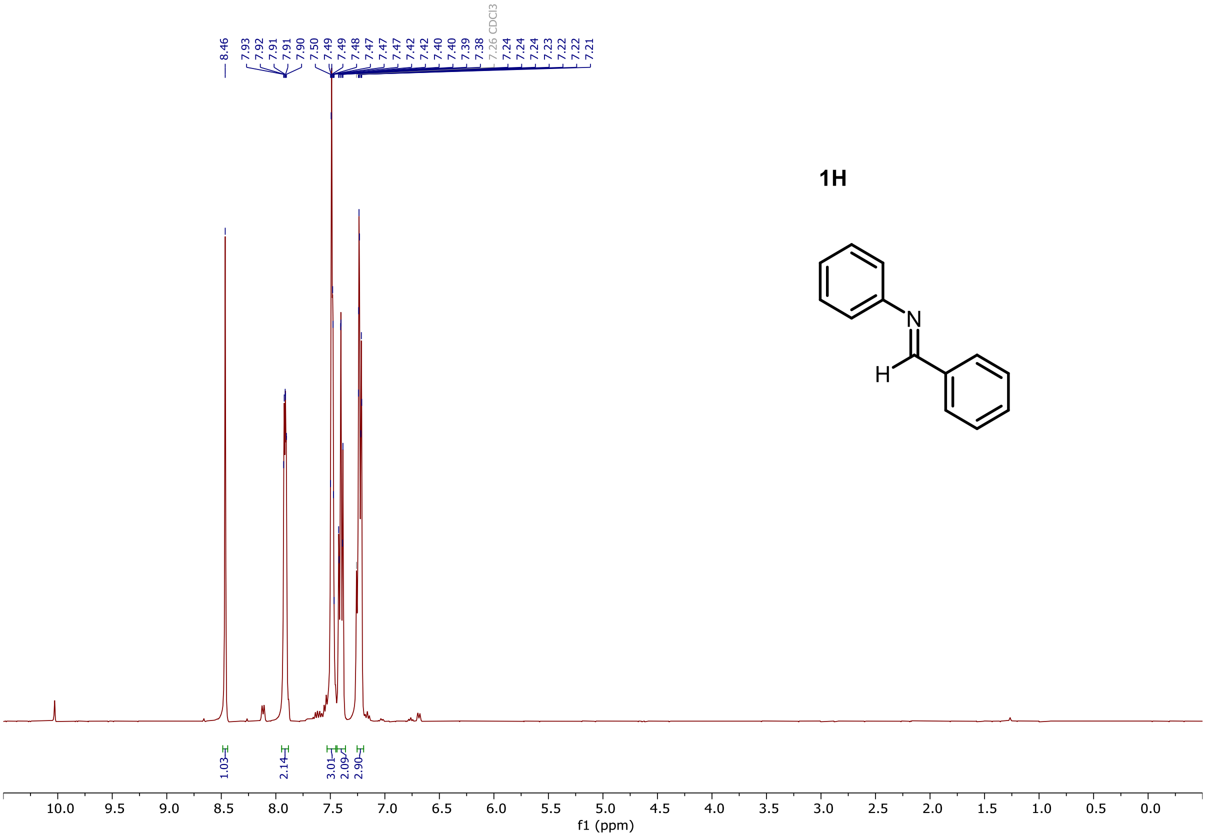

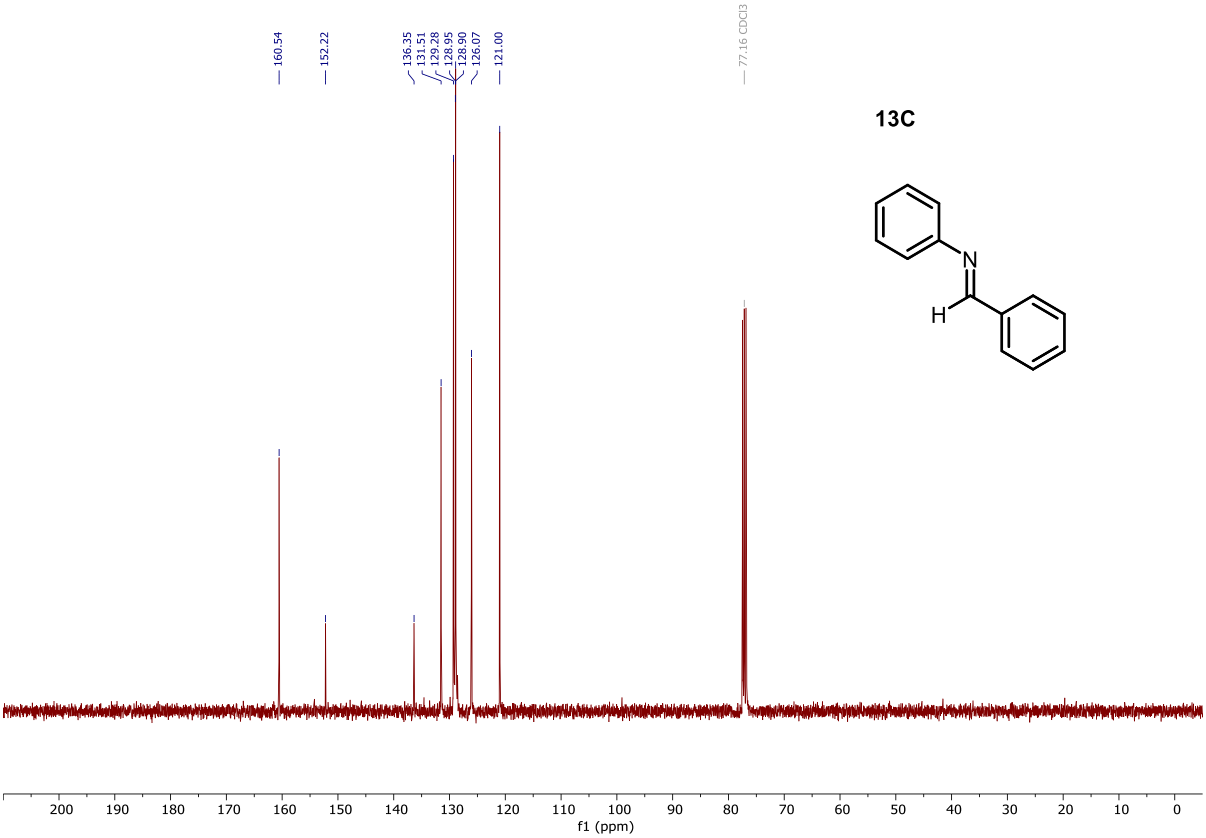

1-Phenyl-*N*-(4-(trifluoromethyl)phenyl)methanimine (*S*2)

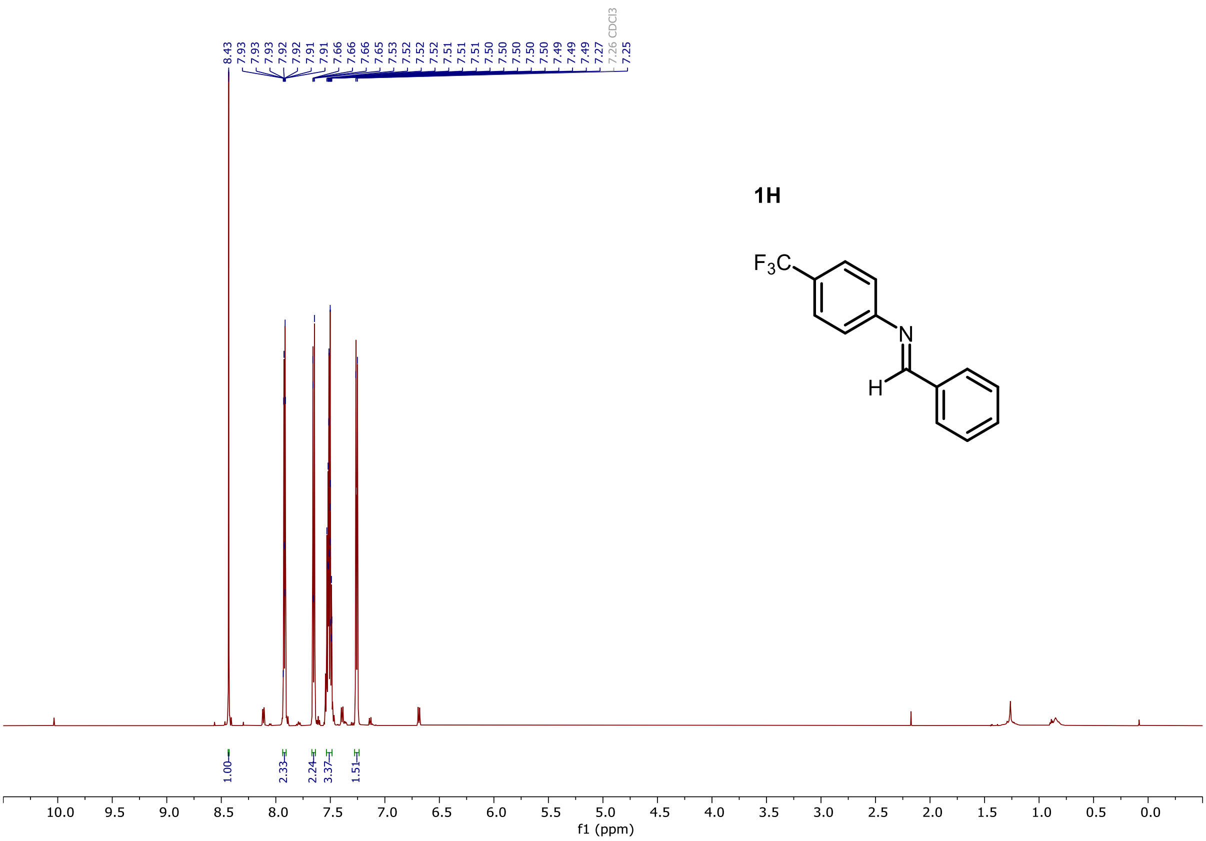

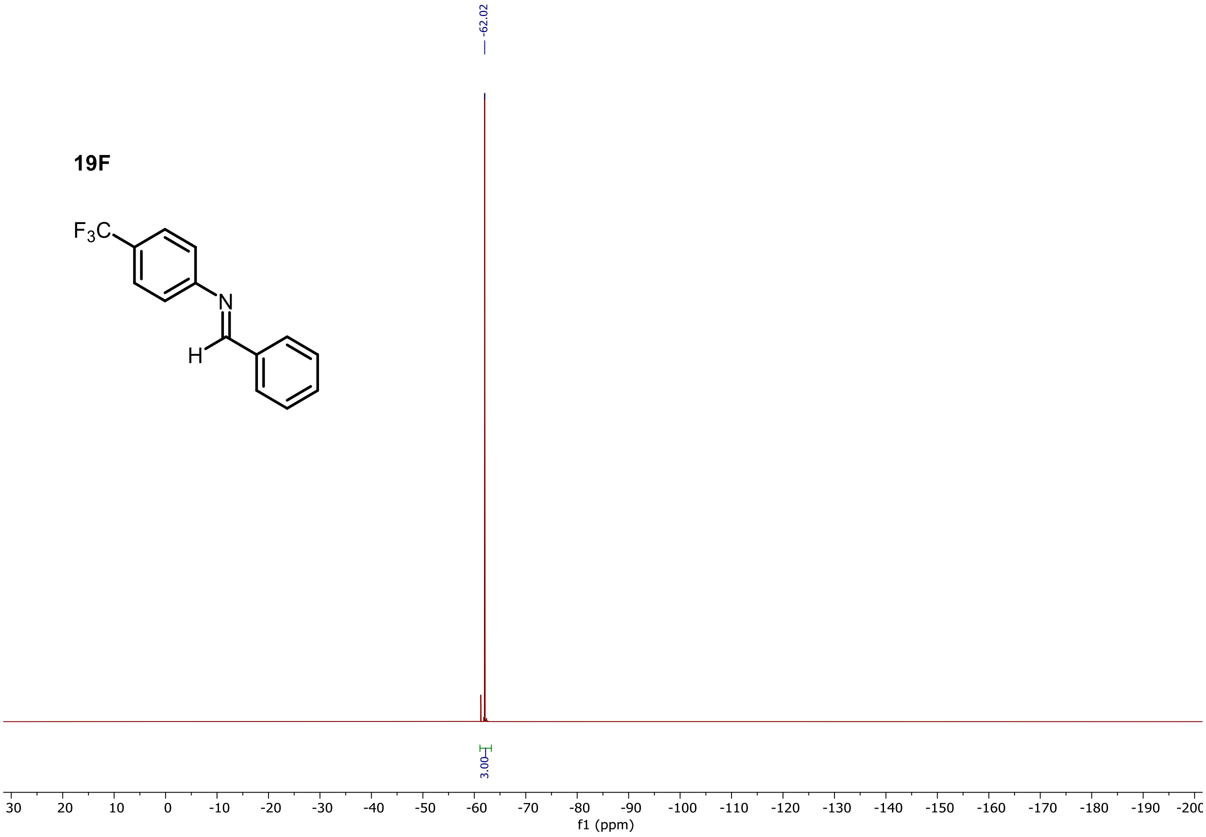

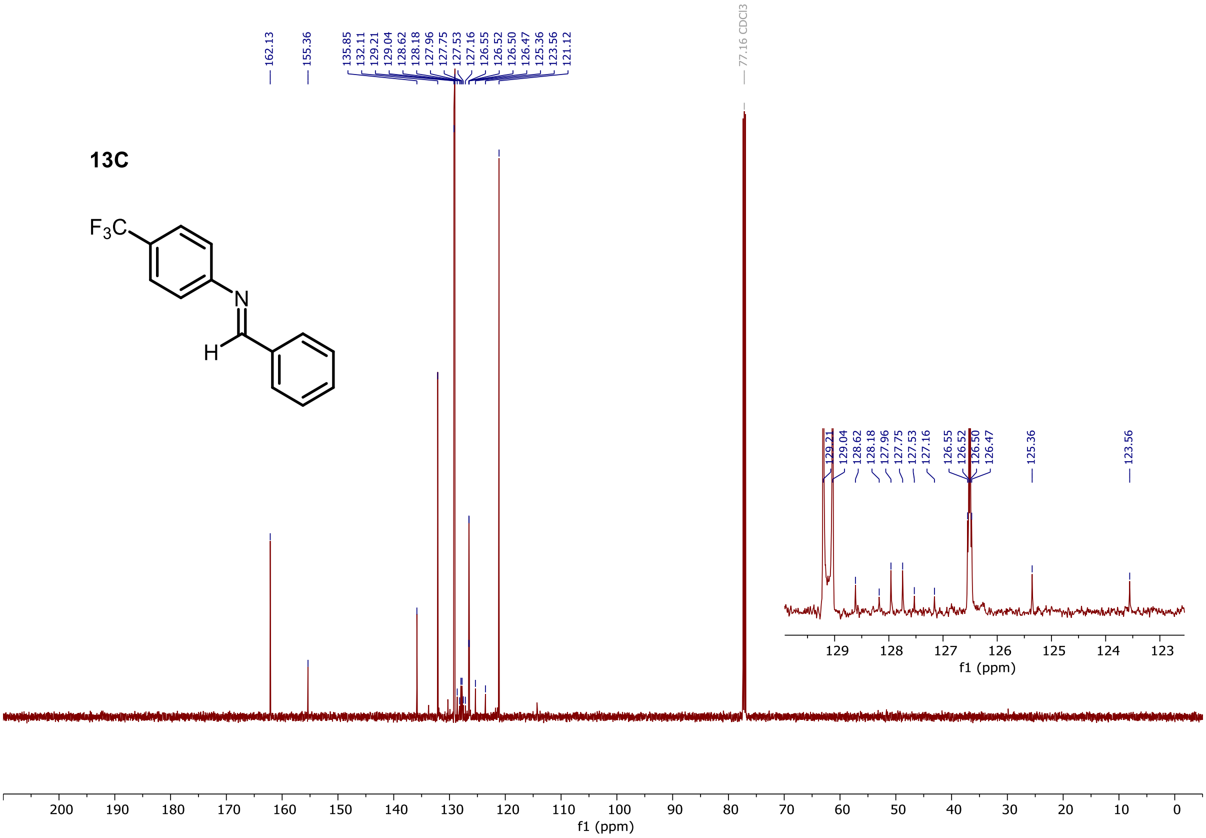

Methyl 4-((2-methylbenzylidene)amino)benzoate (S3)

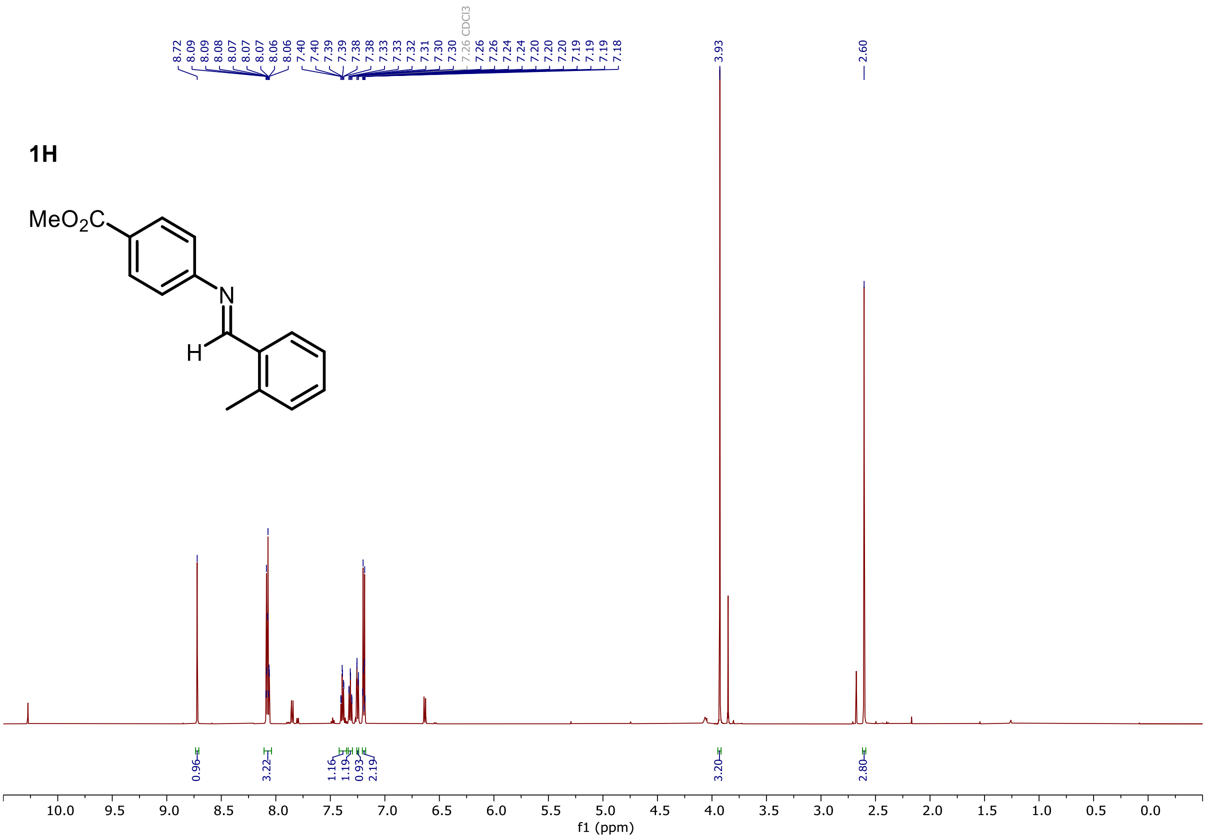

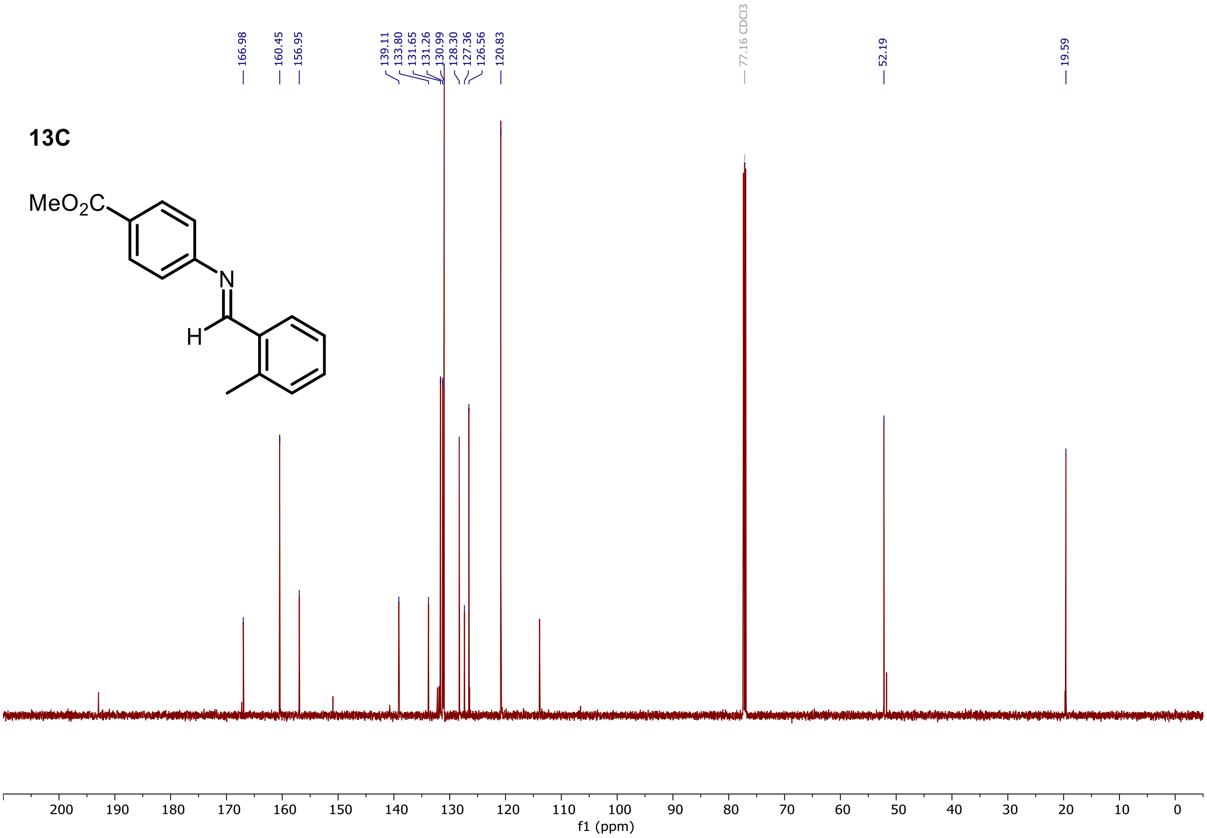

Methyl 4-((2-bromo-5-chlorobenzylidene)amino)thiophene-2-carboxylate (S4)

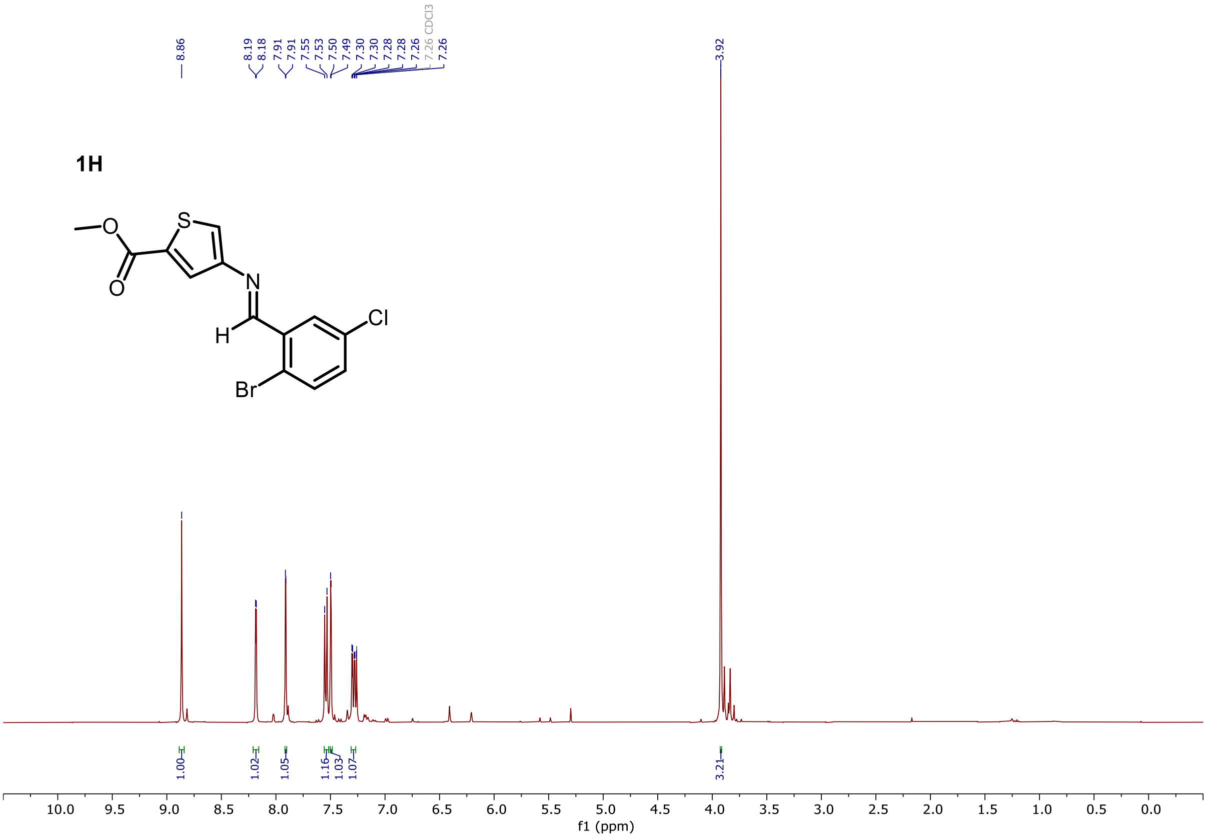

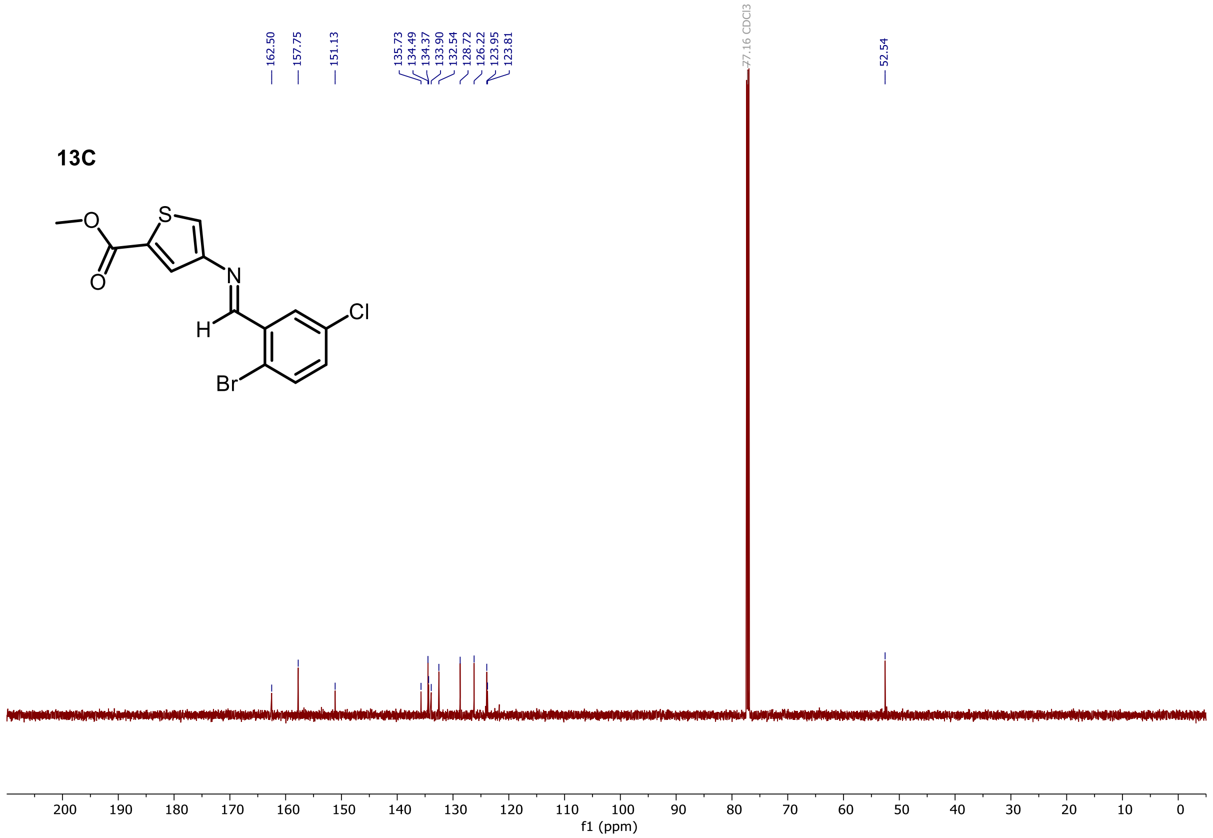

N-(4-Methoxyphenyl)-2-methylbutan-1-imine (S5)

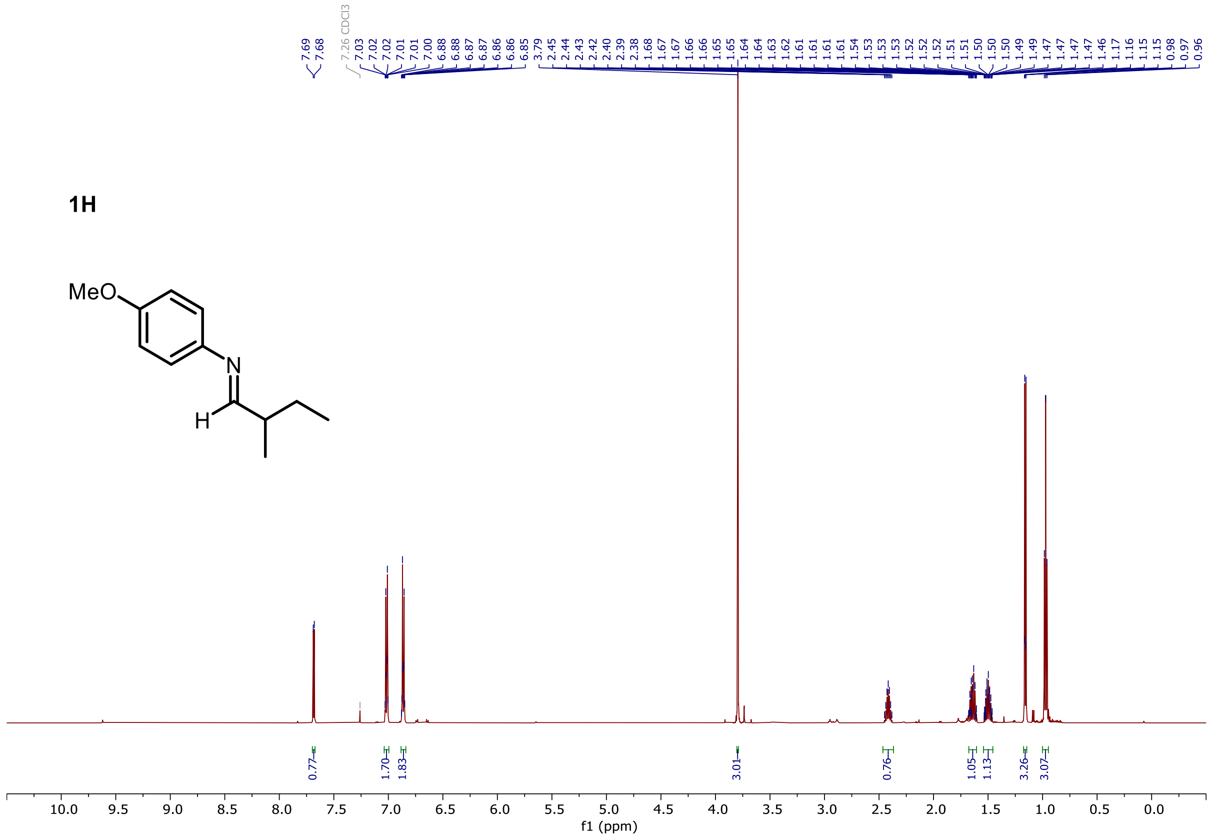

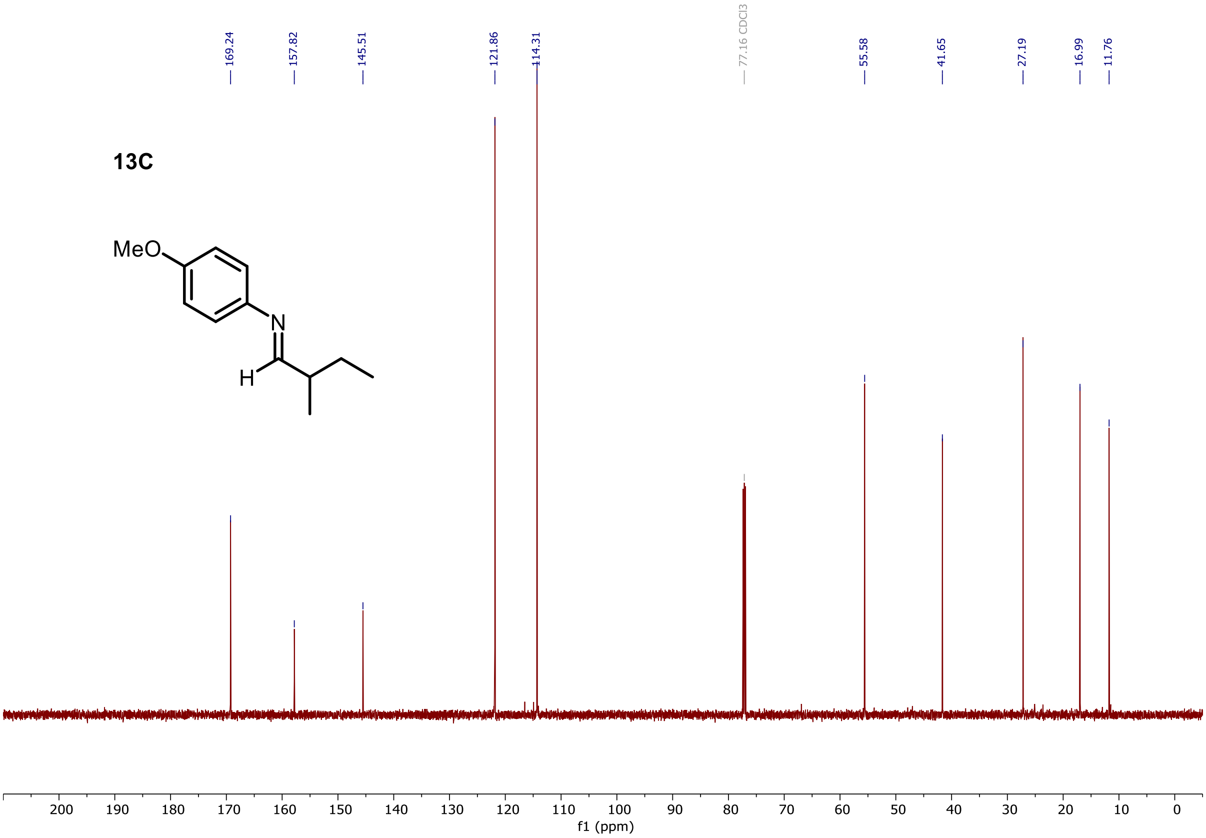

N-(9-Ethyl-9*H*-carbazol-3-yl)-2,2-dimethylpent-4-en-1-imine (S6)

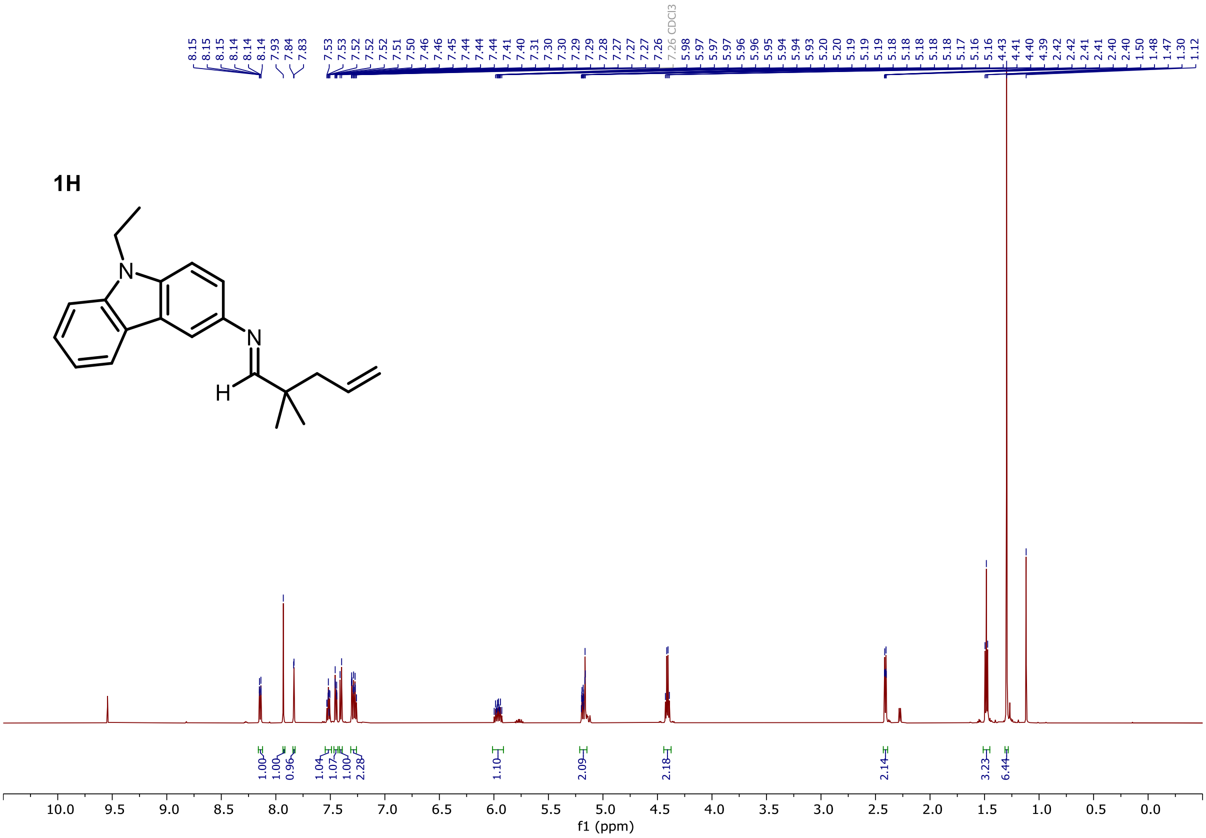

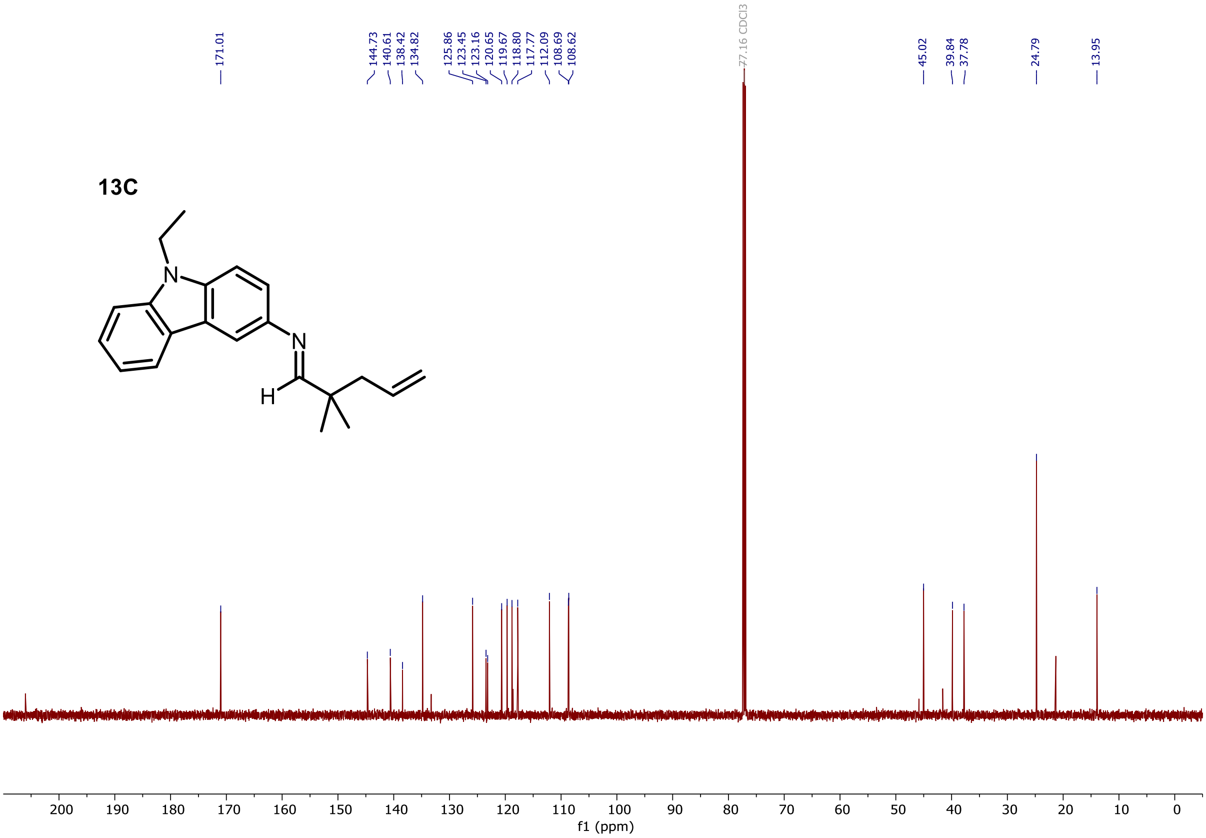

N-(Furan-2-ylmethyl)-1-phenylmethanimine (S7)

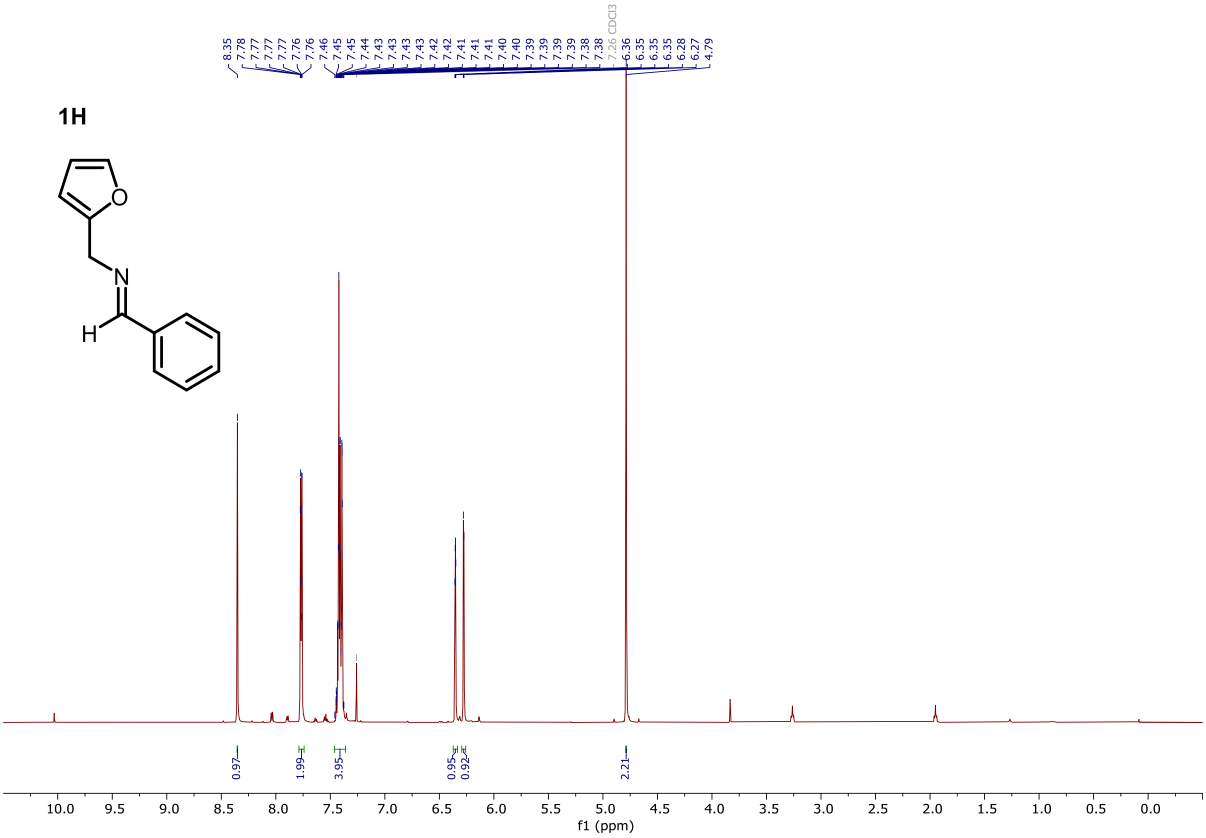

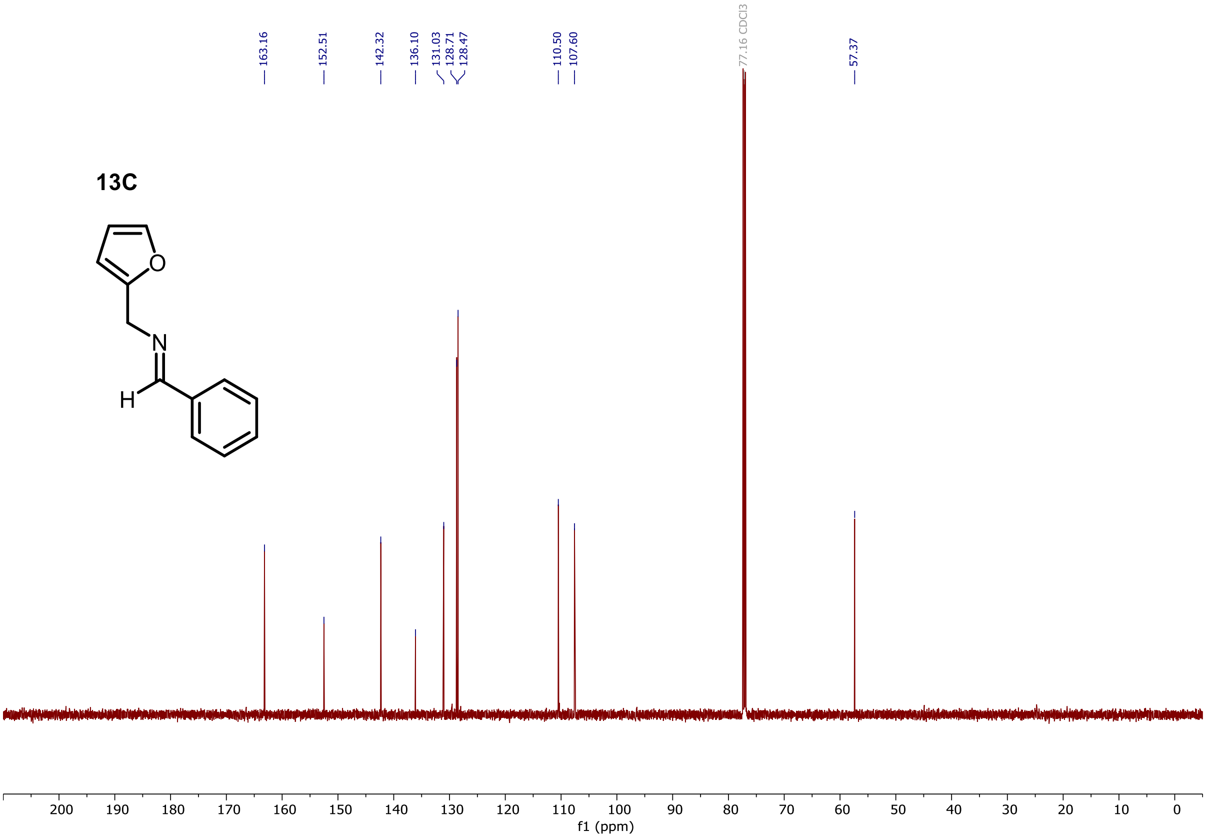

N-Allyl-1-phenylmethanimine (*S8*)

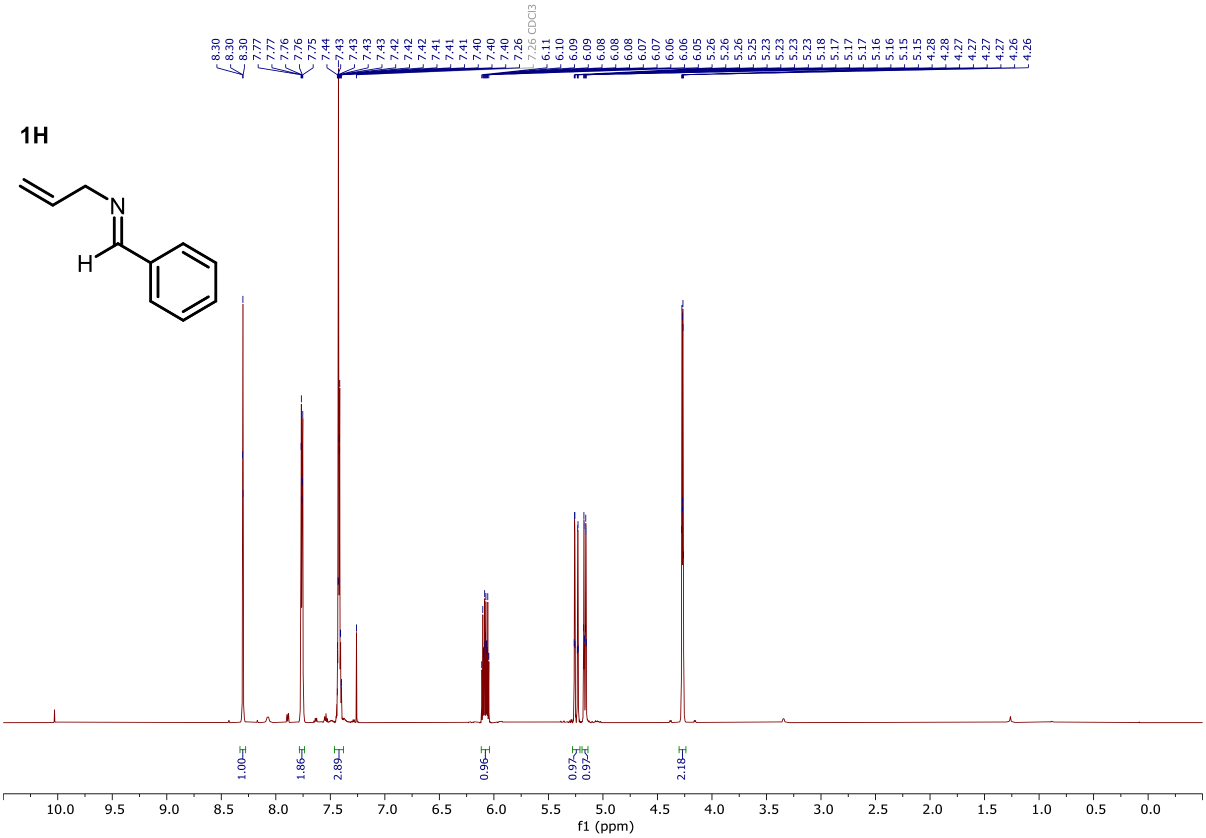

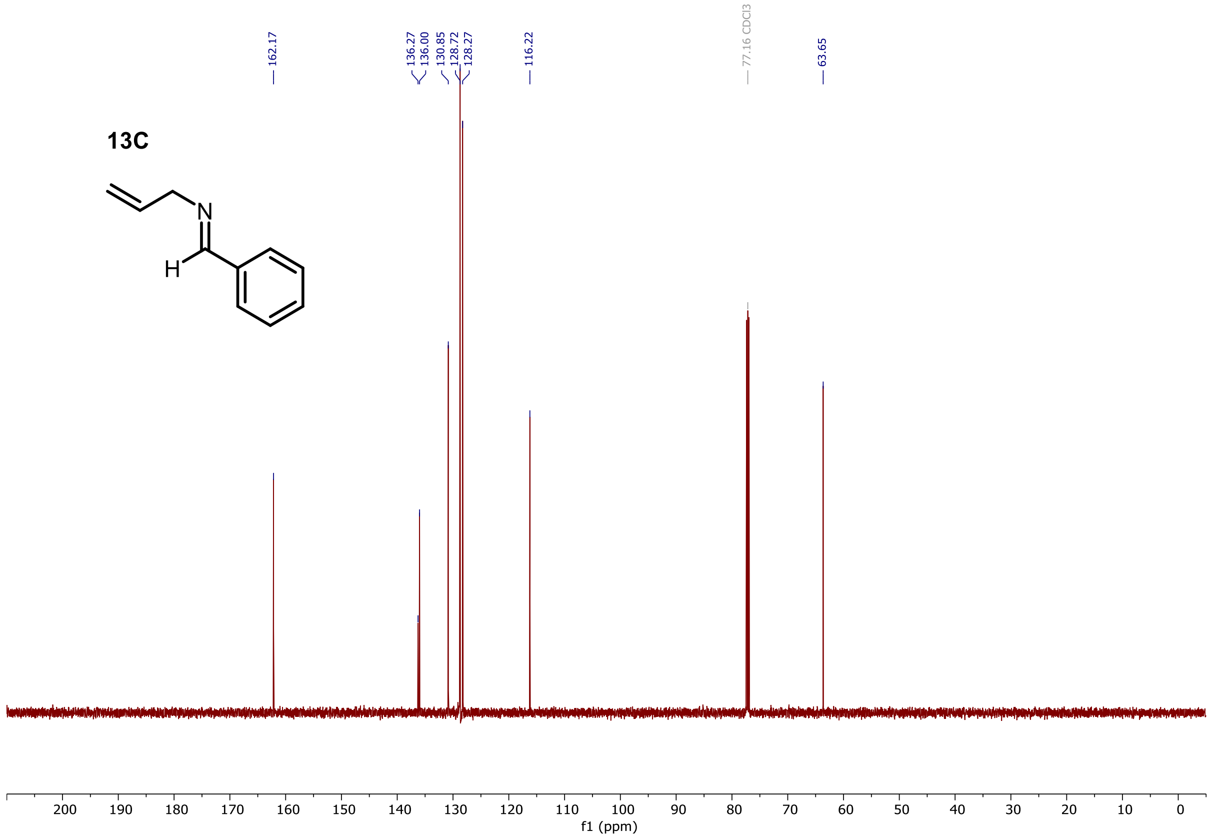

1-(4-Chlorophenyl)-N-cyclohexylmethanimine (S9)

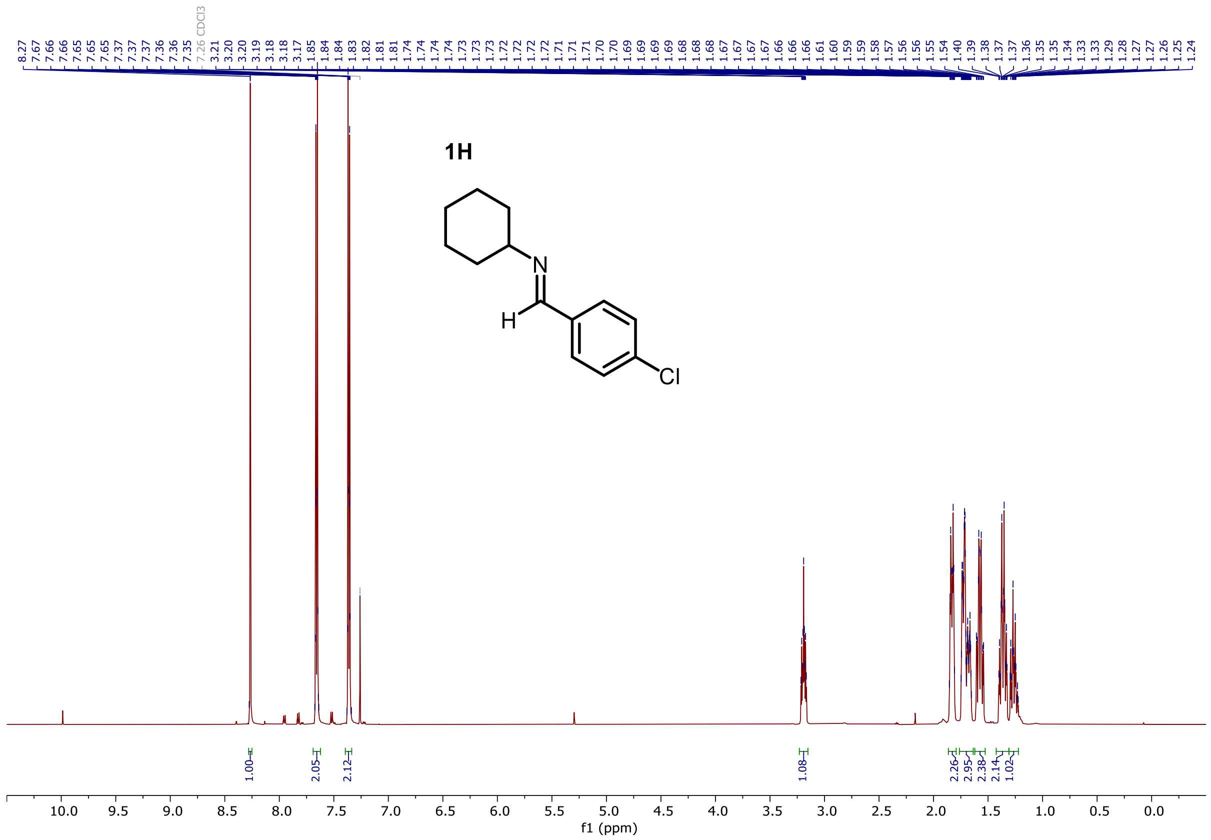

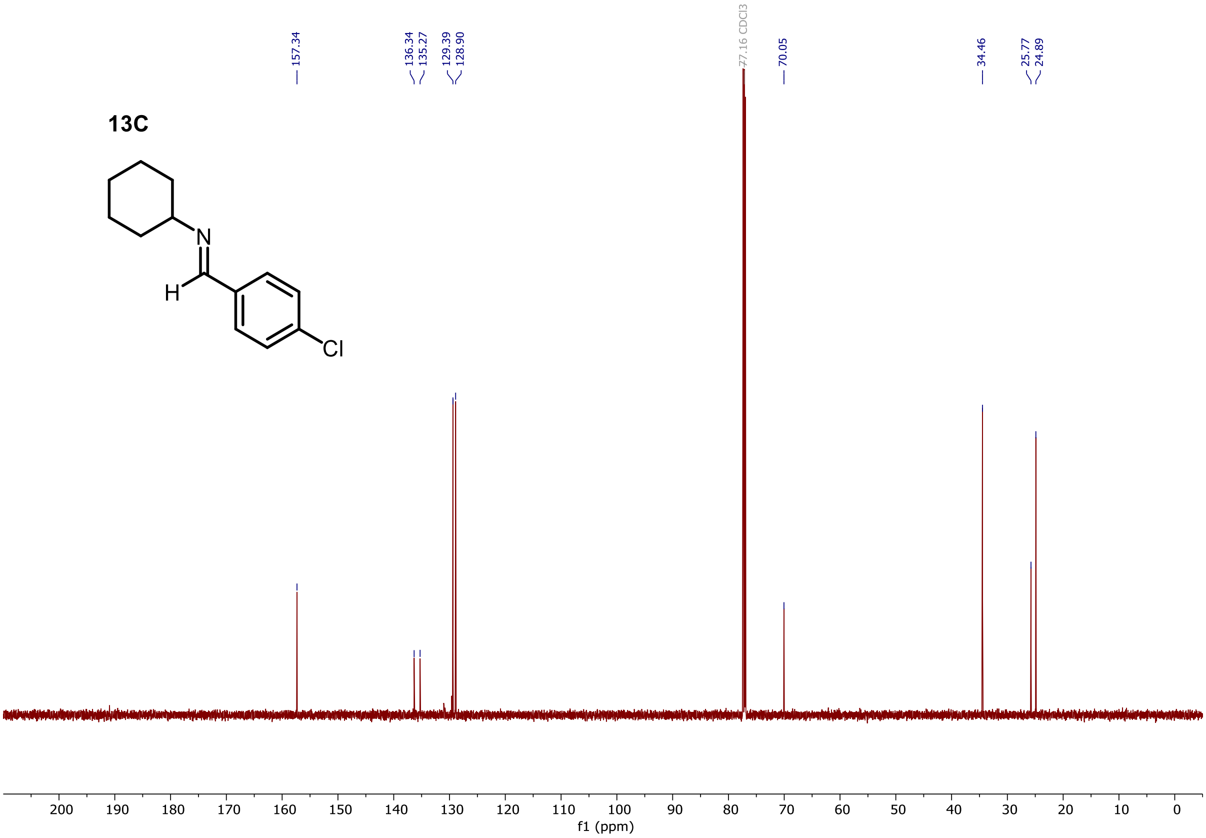

N-(2-Bromophenethyl)-2,2-dimethylpropan-1-imine (S10)

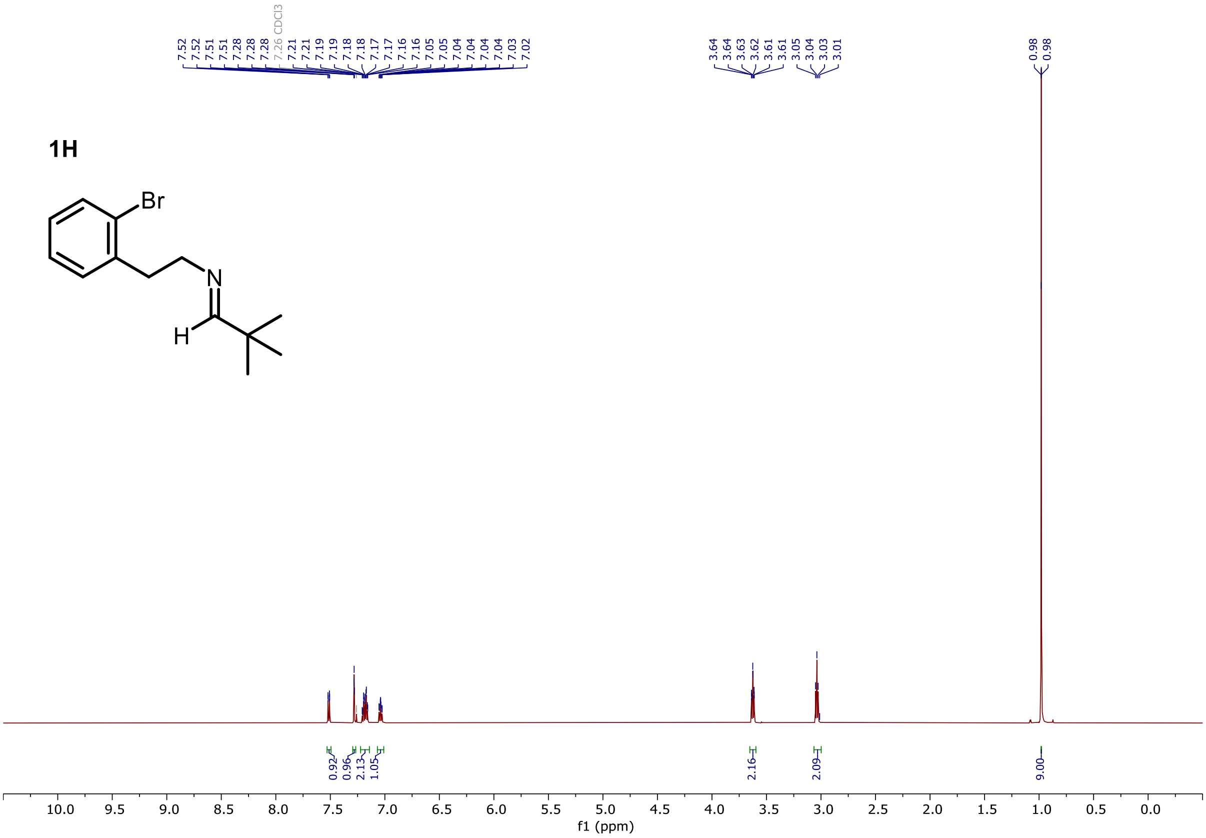

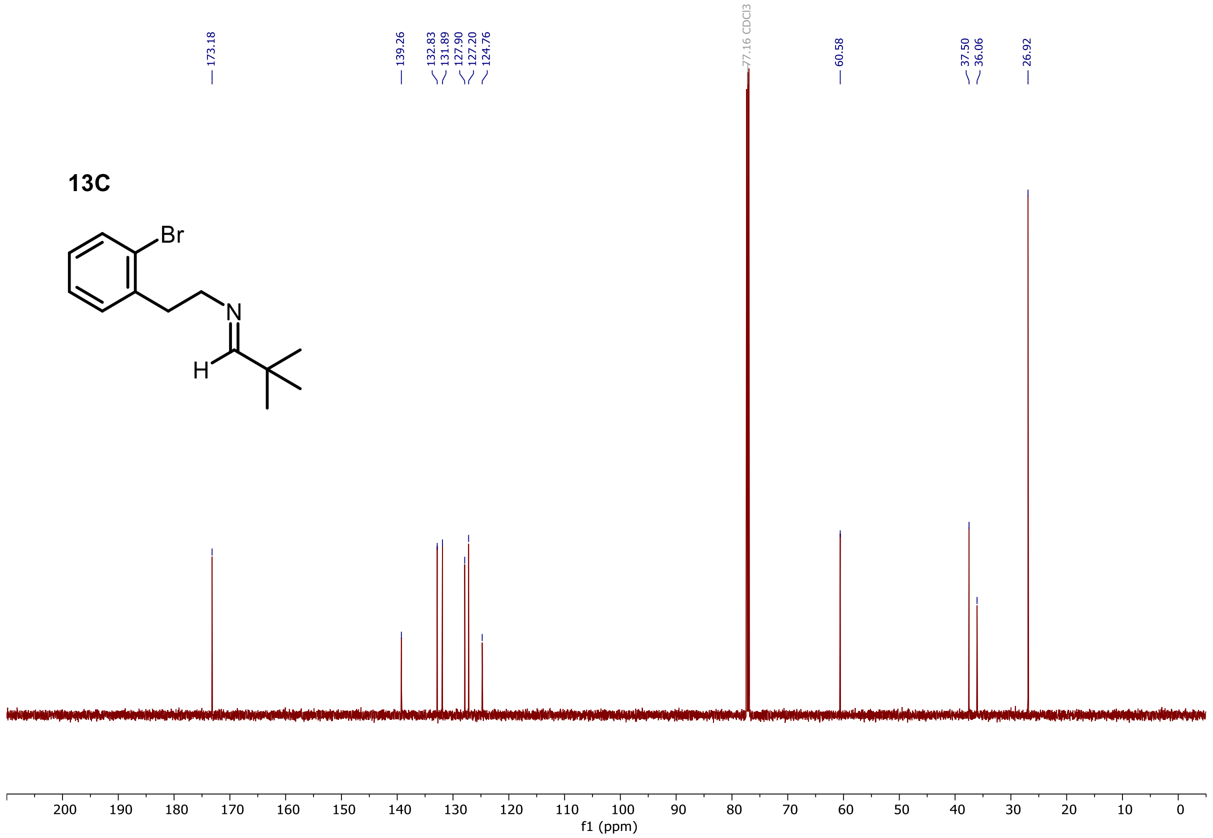
13C
Br
N
H
173.18
139.26
132.83
131.89
127.90
127.20
124.76
77.16 CDCl3
60.58
37.50
36.06
26.92
f1 (ppm)

10.4. TMS-Protected alcohol

1,2:3,4-Bis-*O*-(1-methylethylidene)-6-*O*-(trimethylsilyl)-α-D-galactopyranose (S11)

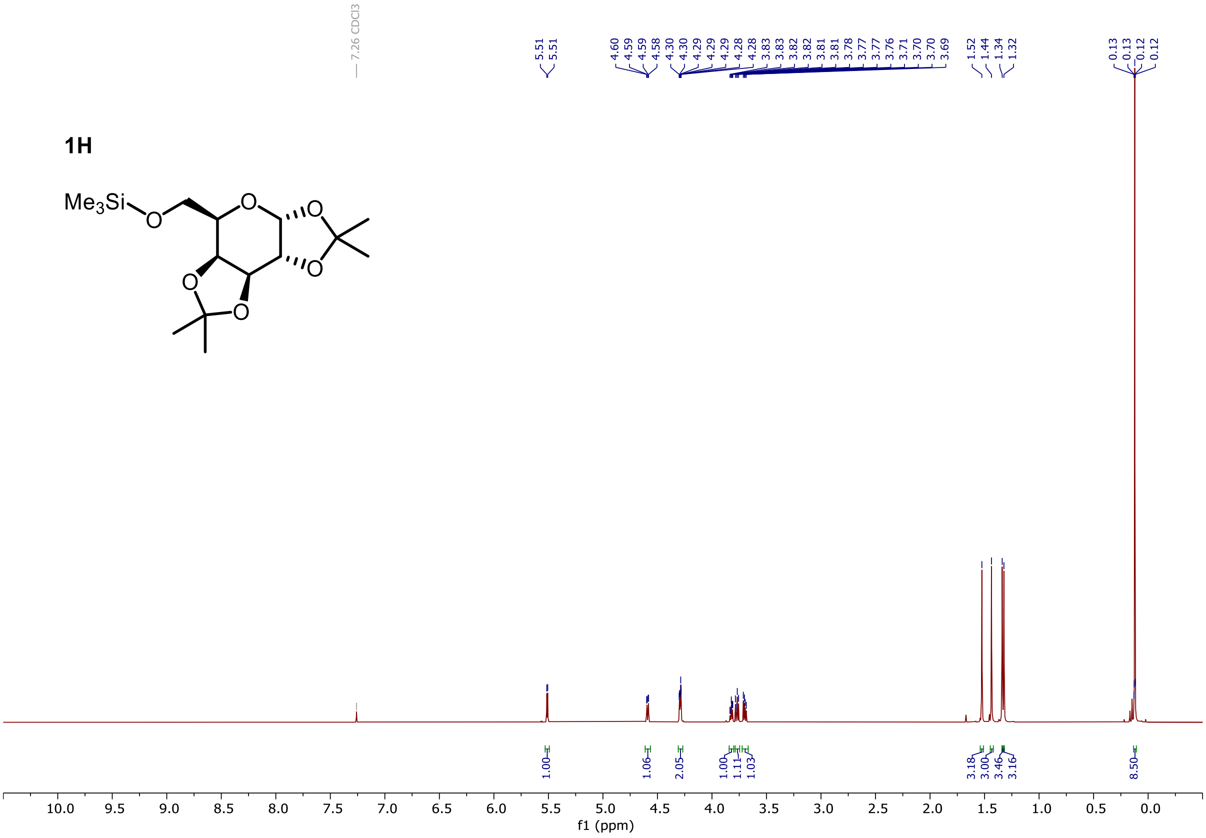

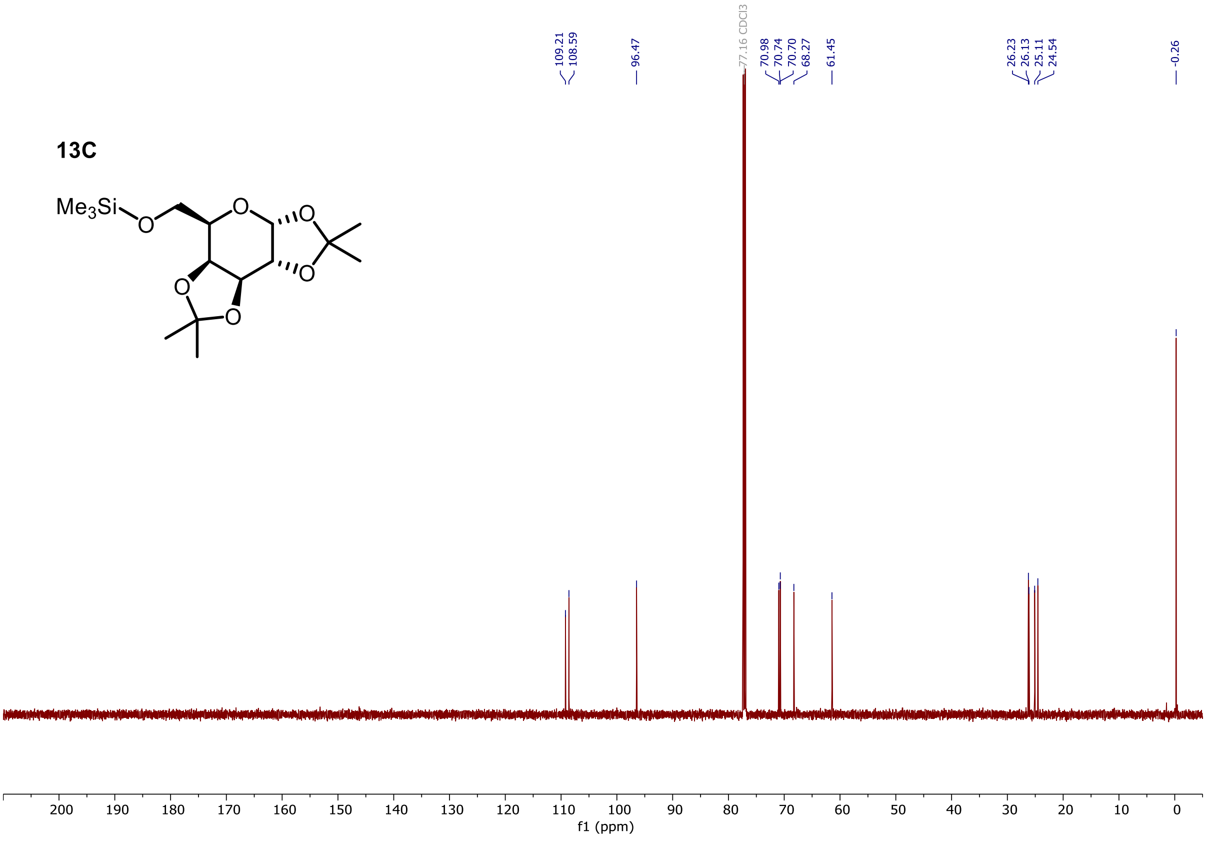

11. References

[1] T. Scattolin, S. Bouayad-Gervais, F. Schoenebeck, *Nature* **2019**, *573*, 102.

[2] A. Turksoy, T. Scattolin, S. Bouayad-Gervais, F. Schoenebeck, *Chem. Eur. J.* **2020**, *26*, 2183.

[3] F. G. Zivkovic, C. D.-T. Nielsen, F. Schoenebeck, *Angew. Chem. Int. Ed.* **2022**, *61*, e202213829.

[4] D. Cadwallader, D. Shevchuk, T. R. Tiburcio, C. M. Le, *Org. Lett.* **2023**, *25*, 7369.

[5] G. Li, G. Yu, C. Wang, T. Morita, X. Zhang, H. Nakamura, *Org. Biomol. Chem.* **2021**, *20*, 113.

[6] S. Morales, F. G. Guijarro, J. L. García Ruano, M. B. Cid, *J. Am. Chem. Soc.* **2014**, *136*, 1082.

[7] B. Karimi, B. Golshani, *J. Org. Chem.* **2000**, *65*, 7228.

[8] a) A. D. Hudwekar, P. K. Verma, J. Kour, S. Balgotra, S. D. Sawant, *Eur. J. Org. Chem.* **2019**, *2019*, 1242; b) R. Ivanov, E. Ivanova, V. Merkulov, M. Zharkov, I. Kuchurov, S. Zlotin, *Eur. J. Org. Chem.* **2023**, *26*.

[9] G. P. Junor, E. A. Romero, X. Chen, R. Jazzar, G. Bertrand, *Angew. Chem. Int. Ed.* **2019**, *58*, 2875.

[10] K. Yeung, R. E. Ruscoe, J. Rae, A. P. Pulis, D. J. Procter, *Angew. Chem. Int. Ed.* **2016**, *55*, 11912.

[11] C. Ouairy, P. Michel, B. Delpech, D. Crich, C. Marazano, *J. Org. Chem.* **2010**, *75*, 4311.

[12] C. Liu, C. Yang, S. Hwang, S. L. Ferraro, J. P. Flynn, J. Niu, *Angew. Chem. Int. Ed.* **2020**, *59*, 18435.

[13] F. Eckert, A. Klamt, *COSMOtherm*, Version C3.0, Release 17.01, COSMOlogic GmbH & Co. KG, available from http://www.cosmologic.de, Leverkusen (Germany), **2016**.

[14] *TURBOMOLE V7.5.1*, a development of University of Karlsruhe and Forschungszentrum Karlsruhe GmbH (1989-2007); TURBOMOLE GmbH (since 2007); available from https://www.turbomole.org, **2021**.

[15] M. J. Frisch, G. W. Trucks, H. B. Schlegel, G. E. Scuseria, M. A. Robb, J. R. Cheeseman, G. Scalmani, V. Barone, G. A. Petersson, H. Nakatsuji et al., *Gaussian 16 Rev. A.03*, Wallingford, CT, **2016**.

[16] F. G. Zivkovic, G. Wycich, L. Liu, F. Schoenebeck, *J. Am. Chem. Soc.* **2024**, *146*, 1276.
